# Photoinduced Halogen-Atom Transfer by *N*-Heterocyclic Carbene-Ligated Boryl Radicals for C(sp^3^)–C(sp^3^) Bond Formation

**DOI:** 10.1021/jacs.2c10444

**Published:** 2022-12-30

**Authors:** Ting Wan, Luca Capaldo, Davide Ravelli, Walter Vitullo, Felix J. de Zwart, Bas de Bruin, Timothy Noël

**Affiliations:** † Flow Chemistry Group, van ’t Hoff Institute for Molecular Sciences (HIMS), University of Amsterdam, Science Park 904, 1098 XH Amsterdam, The Netherlands; ‡ PhotoGreen Lab, Department of Chemistry, University of Pavia, viale Taramelli 12, 27100 Pavia, Italy; § Homogeneous, Supramolecular and Bio-inspired Catalysis Group (HomKat), van’t Hoff Institute for Molecular Sciences (HIMS), Universiteit van Amsterdam (UvA), Science Park 904, 1098 XH, Amsterdam, The Netherlands

## Abstract

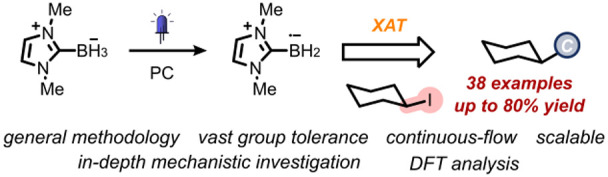

Herein, we present a comprehensive study on the use of *N*-heterocyclic carbene (NHC)-ligated boryl radicals to enable C(sp^3^)–C(sp^3^) bond formation under visible-light irradiation via Halogen-Atom Transfer (XAT). The methodology relies on the use of an acridinium dye to generate the boron-centered radicals from the corresponding NHC-ligated boranes via single-electron transfer (SET) and deprotonation. These boryl radicals subsequently engage with alkyl halides in an XAT step, delivering the desired nucleophilic alkyl radicals. The present XAT strategy is very mild and accommodates a broad scope of alkyl halides, including medicinally relevant compounds and biologically active molecules. The key role of NHC-ligated boryl radicals in the operative reaction mechanism has been elucidated through a combination of experimental, spectroscopic, and computational studies. This methodology stands as a significant advancement in the chemistry of NHC-ligated boryl radicals, which had long been restricted to radical reductions, enabling C–C bond formation under visible-light photoredox conditions.

## Introduction

**Figure 1:**
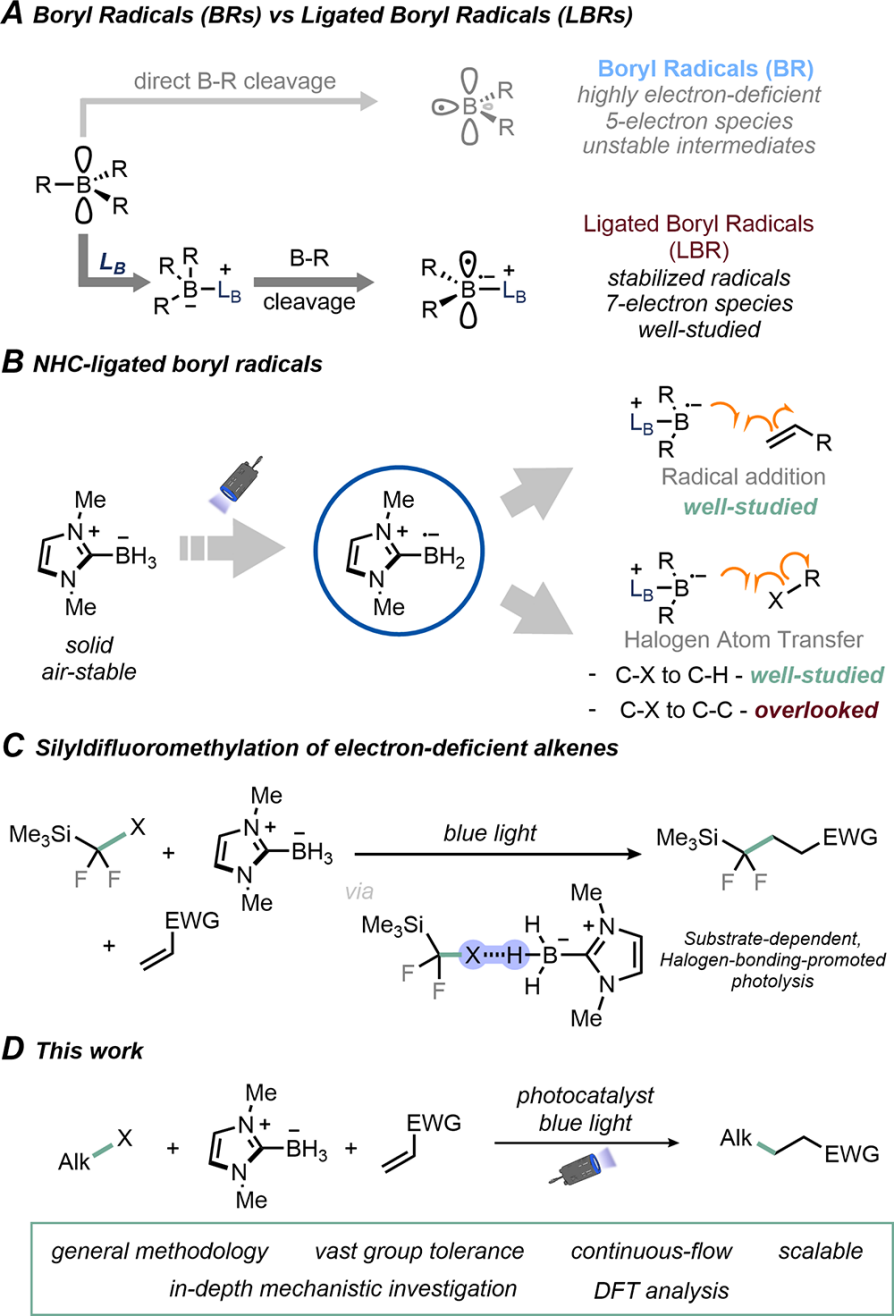
(A) Boryl radicals (BRs) versus ligated boryl radicals (LBRs). (B) NHC boranes as a bench-stable source of LBRs. (C) Silyldifluoromethylation of electron-deficient alkenes. (D) This work. L_B_: Lewis base.

The possibility to exploit photonic energy in organic synthetic endeavors has dramatically impacted the way chemists assemble molecules. In particular, photocatalysis has enabled a convenient entry to open-shell intermediates,^1^ spurring the development of efficient manifolds for the generation of C-,^2^ N-,^3^ and O-^4^ centered radicals, as well as halogen radicals,^5^ which can be subsequently used to forge new chemical bonds.^6^ In contrast, boron-based congeners have long remained in obscurity,^7^ mainly due to the intrinsic difficulties associated with the handling of these highly electron-deficient and unstable intermediates. However, ligated boryl radicals (LBRs),^8^ i.e. boron-centered radicals where the boron atom is coordinated with a Lewis base, are more stable and provide a suitable entry for use in radical chemistry (Figure 1A).^9,10^

In particular, *N*-heterocyclic carbene-based (NHC) boranes are emerging as a convenient source of LBRs: NHC boranes are stable crystalline compounds, and a diverse array of NHCs can be ligated to boranes allowing the LBR properties to be fine-tuned.^11^ Notably, these boranes can be uniquely paired with photocatalysis to generate the targeted boron-centered radicals.^12^ Herein, a photocatalyst absorbs visible light and engages with the ligated borane in a single-electron transfer step which, upon deprotonation, generates the corresponding boryl radical. Although these boron-centered radicals have attracted mainly interest from the synthetic community as nucleophilic radicals,^13,14^ they have also been used in the role of halogen-atom transfer (XAT) agents.

In the latter scenario, the halogen-affinity of the LBR is exploited for the homolytic activation of a C–X bond to yield carbon-centered radicals. However, this manifold has been so far mainly used to reduce C–X bonds into the corresponding C–H bonds via a radical chain mechanism.^11,12,15^ Surprisingly, boryl radicals have been largely overlooked for the construction of C–C bonds (Figure 1B). In an early example, the radical silyldifluoromethylation of electron-deficient alkenes was reported.^16^ Herein, a very specific interaction, based on halogen-bonding between the substrate and an NHC borane, was needed to trigger the desired C–X bond photolysis and subsequently initiate the radical chain mechanism sustained by the LBR (Figure 1C). Inspired by this report, we questioned whether it would be possible to realize a more general strategy to generate the pivotal ligated boryl radical. Such a pathway might allow the engagement of a broader array of substrates, ultimately leading to a general approach for C–C bond formation. Moreover, succeeding in this challenge would provide a cheap, tunable, and sustainable alternative for other XAT-based approaches^17^ using silyl^18^ and α-aminoalkyl radicals.^19^ To this end, we disclose our results regarding the development of a mild and broadly applicable protocol for C(sp^3^)–C(sp^3^) bond formation using photoinduced XAT by NHC-ligated boryl radicals under blue-light irradiation (Figure 1D). The key role of the NHC-ligated boryl radicals in the operative reaction mechanism has been uncovered through a combination of experimental, spectroscopic, and computational studies.

## Results and Discussion

At the outset of our investigations, we recognized the importance of finding the ideal combination of ligated borane and photocatalyst to establish an efficient and competent system to promote the desired reactivity. In detail, the photocatalyst would absorb the visible light and subsequently generate the LBR via a SET followed by deprotonation.^12^ The resulting boryl radical would be ultimately entrusted with the XAT step.

We immediately realized that the success of our plan hinges on (i) the redox potentials of the borane and the photocatalyst and (ii) the halogen-affinity of the resulting LBR. Based on literature and our experimental data (see Section 11 in the Supporting Information), we identified NHC-ligated borane **B1** as an ideal candidate for our purposes: in fact, its oxidation potential (*E*_*pa*_(**B1**^**•+**^/**B1**)) is +0.89 V vs SCE,^20^ which suggests that this approach should be feasible in combination with routinely used photoredox catalysts. Other ligated boranes tested (see Section 11 in the Supporting Information) were found to have an exceedingly high redox potential, thus preventing the formation of the desired ligated boryl radical.

Next, we started our investigation by screening different photoredox catalysts that possess a higher excited state reduction potential (*E*(PC*/PC_red_)) than that of **B1** (Table 1). Our experiments revealed that, when a degassed CH_3_CN solution of **2a** (0.1 M), **1a** (2 equiv), and **B1** (1 equiv) was irradiated with blue light (λ = 456 nm, 12 h, rt) in the presence of 2 mol % of the organic photocatalysts **PC1** or **PC2**, product **3** could be obtained in 43–44% GC yield (Table 1, entries 1 and 2). In contrast, Ru(bpy)_3_(PF_6_)_2_ (**PC3**) gave worse results (Table 1, entry 3).

**Table 1:**
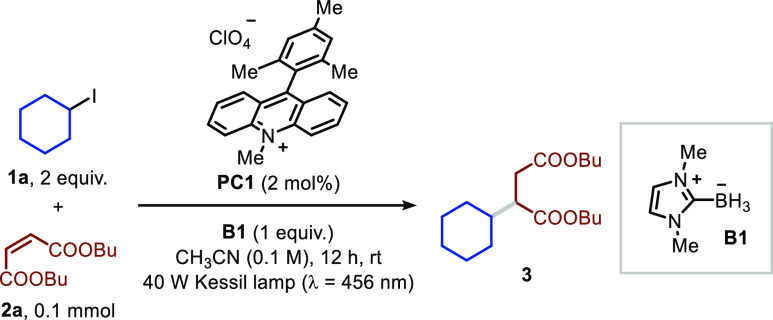

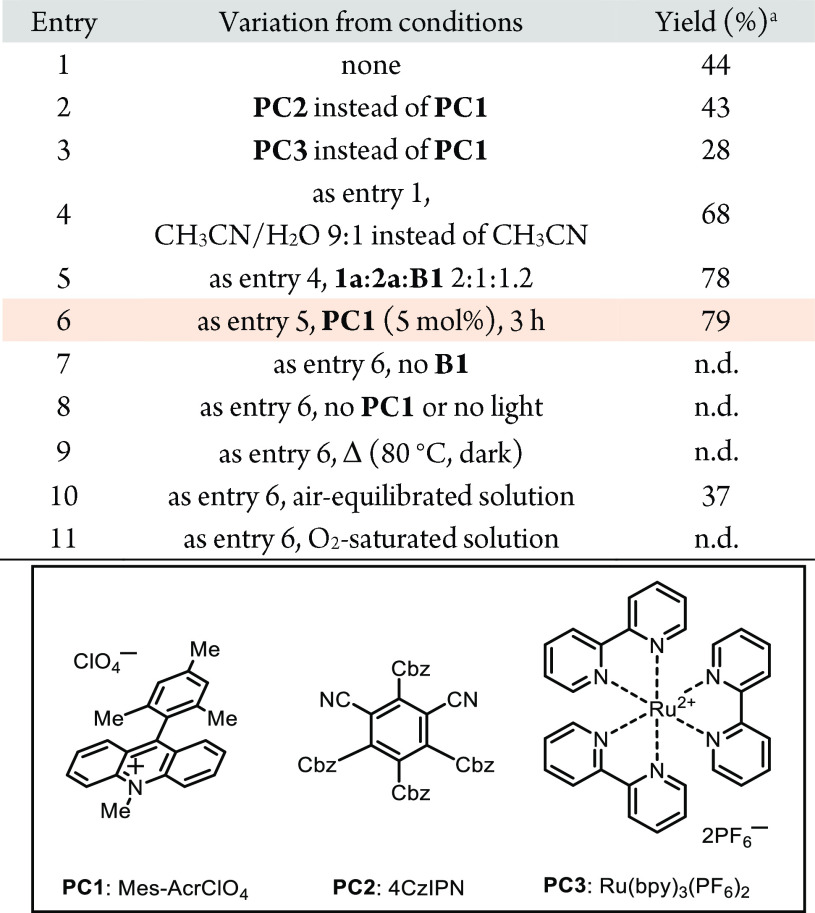
Optimization of Conditions^a^

a GC yields are given using biphenyl as external standard.

Next, we screened the effect of the solvent on the transformation and noticed that protic reaction mixtures boosted the reaction yield (Table 1, entry 4: up to 68% in CH_3_CN/H_2_O 9:1). Fine-tuning the ratio of the reagents, the photocatalyst loading, and reaction time allowed an excellent 79% yield to be obtained for the targeted hydroalkylation (Table 1, entries 5–6). Several control experiments revealed that excluding light, **PC1**, or **B1** did not lead to any product formation (Table 1, entries 7–8). Moreover, **3** was not produced at elevated temperatures either (Table 1, entry 9). The exclusion of molecular oxygen appeared to be crucial as air-equilibrated conditions led to a significantly reduced yield (Table 1, entry 10: 37%), while O_2_-saturation shut down reactivity (Table 1, entry 11). It is important to mention that, when direct UV-A light irradiation (Kessil lamp, λ = 390 nm, full intensity) was used, product **3** was formed in a 75% assay yield, without any photocatalyst added.

**Figure 2:**
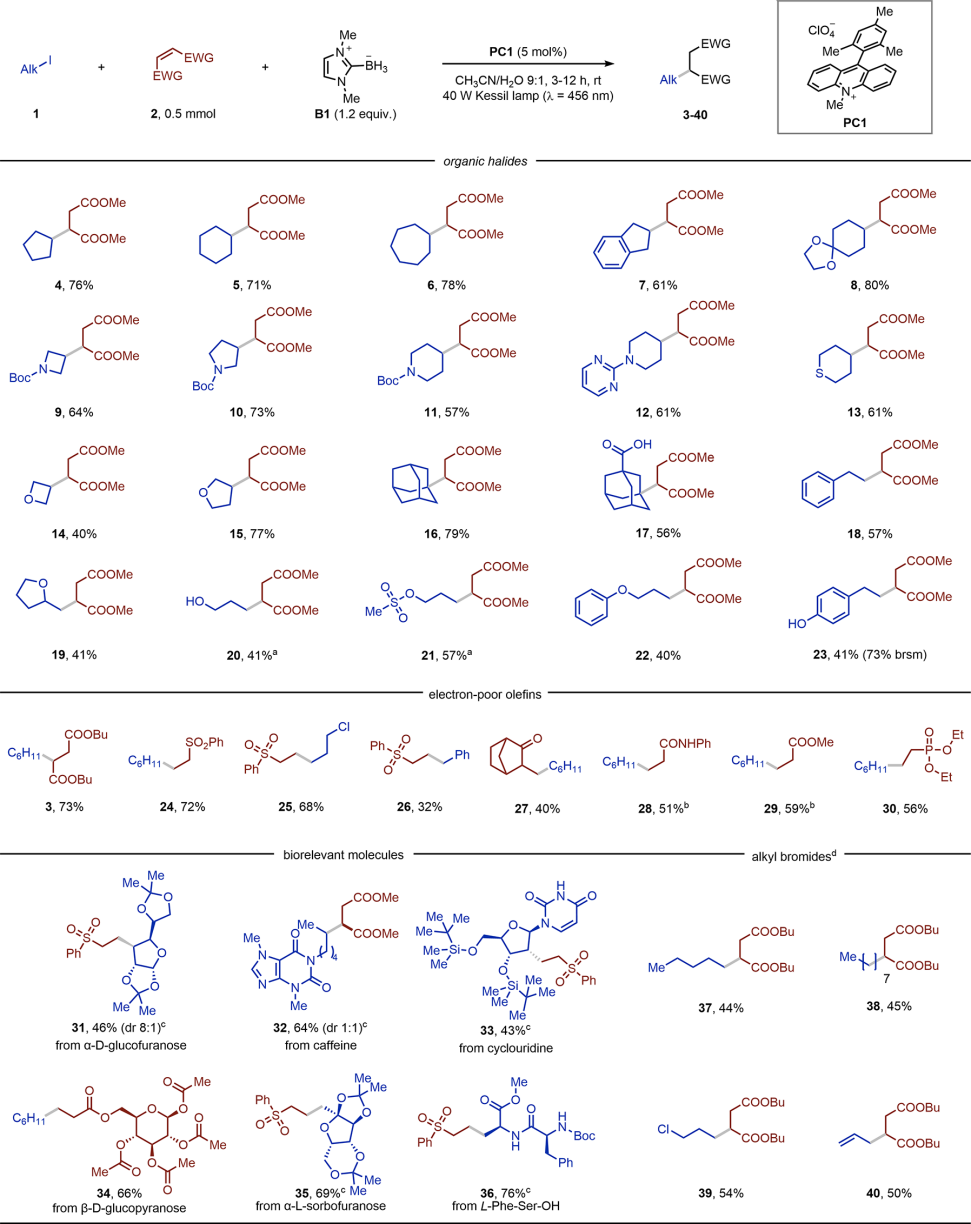
Substrate scope for LBRs-mediated XAT under visible-light irradiation. For secondary and tertiary organic halides: **2** (0.5 mmol), **1** (2 equiv), **B1** (1.2 equiv) in CH_3_CN/H_2_O 9:1 (5 mL) in the presence of **PC1** (5 mol %), 3 h. For primary organic halides: **2** (2 equiv), **1** (0.5 mmol), **B1** (1.2 equiv) in CH_3_CN/H_2_O 9:1 (5 mL) in the presence of **PC1** (5 mol %), 12 h. Solutions were bubbled with N_2_ (5 min) prior to irradiation (λ = 456 nm). ^a^ Reaction time: 18 h. ^b^ Solvent: ethyl acetate. ^c^ See Supporting Information for further details. ^d^ NaI (2 equiv) was added to the reaction mixture. brsm: based on remaining starting material.

Using the optimized set of conditions (Table 1, entry 6), we next evaluated the scope of the visible-light induced hydroalkylation protocol (Figure 2).

Hereto, dimethyl maleate was combined with several alkyl iodides, and we found that the expected products were obtained in all cases (**4**–**23**). Notably, the acid-sensitive acetal function is well tolerated under our optimized conditions (**8**, 80%). Also iodides of medicinally relevant nitrogen-containing scaffolds, including Boc-protected azetidine, pyrrolidine, and piperidine, were competent reaction partners, allowing isolation of the corresponding adducts in good yields (**9**–**12**, 57–73%). In a similar vein, oxygen- and sulfur-containing alkyl iodides could be engaged in the reaction protocol (**13**–**15**, 40–77%). Next, we employed 1-iodoadamantane as a model for tertiary alkyl iodides, and we found that the hydroalkylated product was obtained in very good yield (**16**, 79%). The presence of a free carboxylic acid slightly reduced the reaction efficiency; however, the targeted compound could still be accessed in synthetically useful quantities (**17**, 56%).

Finally, we focused on primary alkyl iodides, which are interesting yet more challenging to engage in the reaction protocol. A slight modification of the reaction conditions (see GP4 in the Supporting Information, including an inverted organic halide/olefin ratio and extended light exposure) resulted in complete conversion and yielded the compounds **18**–**23** in satisfactory yields (40–57%). Of note are the unprotected aliphatic alcohols (**20**) and easily oxidizable phenols (**23**).

With respect to the SOMOphile scope, we found that different olefins could successfully take part in the transformation (**3**, **24**–**30**). The product of our benchmark reaction (**3**) was obtained in 73% yield. Notably, when using phenyl vinyl sulfone (**2c**), we were able to trap stabilized benzyl radicals, while the reaction with dimethyl maleate did not afford the expected product. Also, norbornenone, *N*-phenyl acrylamide, methyl acrylate, and diethyl vinyl phosphonate could be engaged as SOMOphiles in the reaction protocol, showing its tolerance toward a wide variety of functional groups, such as ketones, amides, esters, and phosphonates (**27**–**30**, 40–59% yield). The applicability of our visible-light photocatalytic hydroalkylation process was also demonstrated by the fact that several derivatives of biologically active molecules could be readily modified; these include densely functionalized compounds, such as derivatives of sugars (i.e., α-d-glucofuranose and α-l-sorbofuranose), caffeine, cyclouridine, and even a dipeptide (**31**−**36**, 43–76%).

While alkyl bromides were not reactive under our original reaction conditions, we found that simple addition of NaI (2 equiv) could obviate this issue by generating the corresponding alkyl iodide *in situ* via S_N_2. With this operationally facile approach, we could subject various primary alkyl bromides to the hydroalkylation strategy and obtain the targeted compounds in decent yields (**37**–**40**, 44–50% yield).

**Figure 3:**
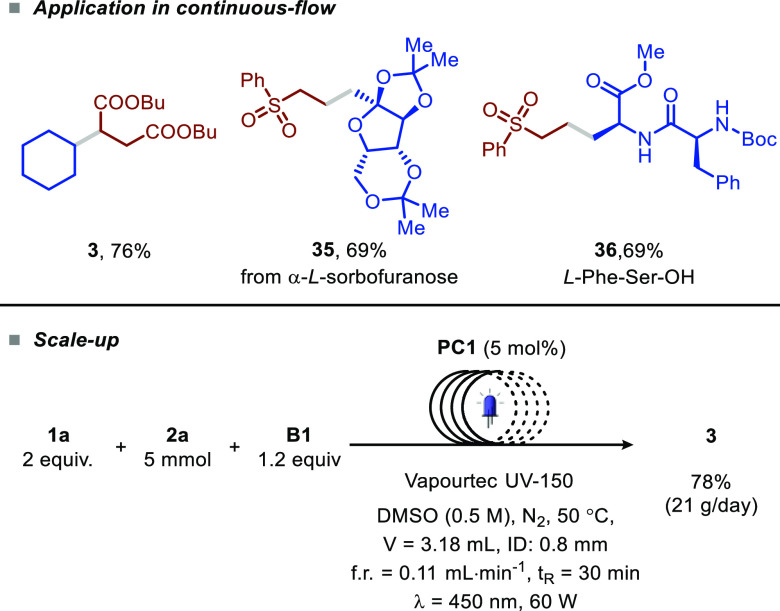
Translation to continuous-flow conditions (see GP6 in the Supporting Information) and scale-up.

Finally, we also successfully translated our batch protocol to a fast and scalable continuous-flow process, which should enable a fast transition between medicinal and process chemistry (see GP6 described in the Supporting Information).^21^ In flow, we were able to prepare compounds **3**, **35**, and **36** in good yields requiring only 30 min of light exposure. We also exploited the continuous-flow technology to scale our benchmark reaction up to 5 mmol (78% yield of the isolated product, Figure 3), corresponding to a productivity of 21 g d^–1^ of compound **3**.

**Figure 4:**
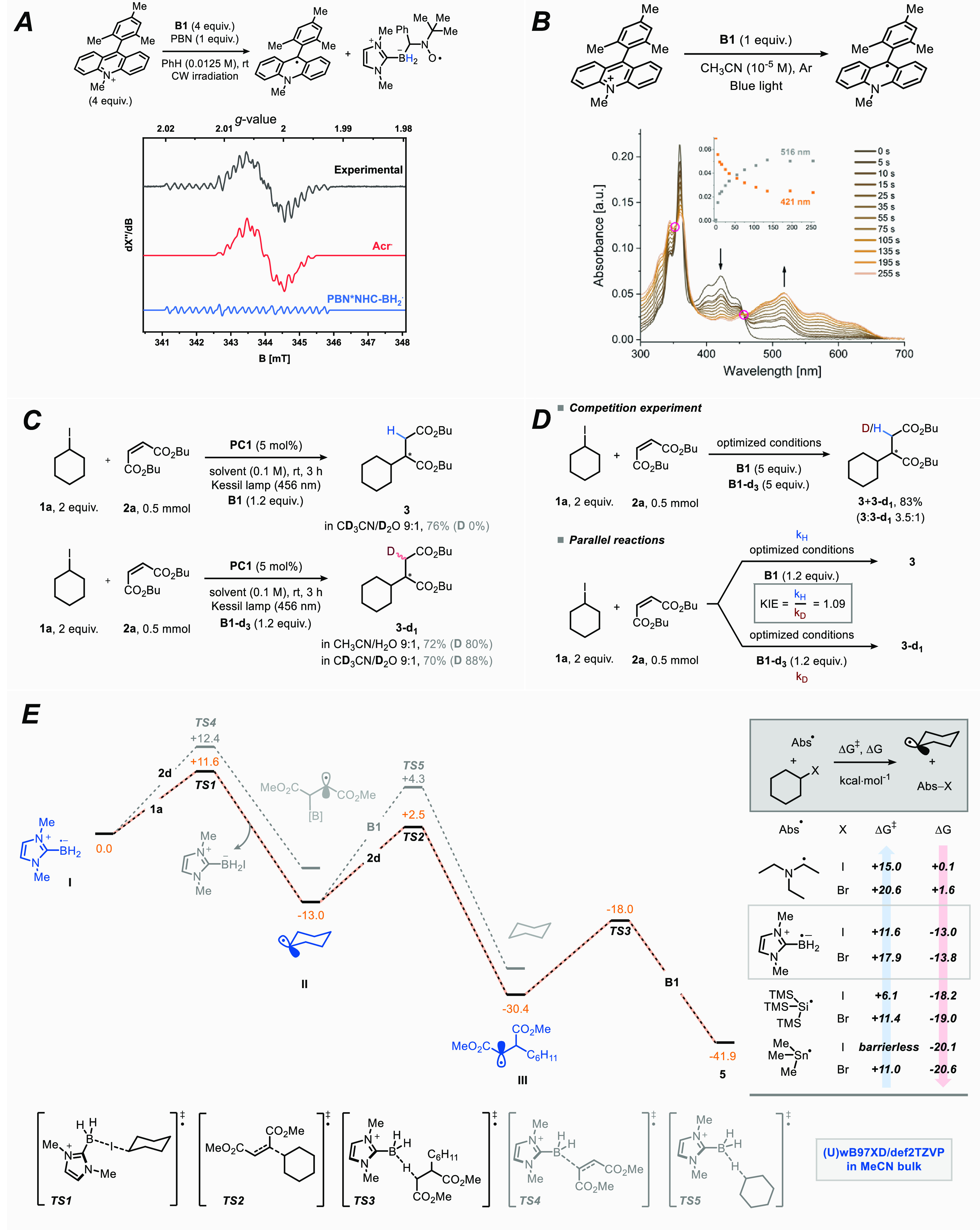
(A) Electron paramagnetic resonance (EPR) spectrum (black) obtained upon irradiation (λ = 460 nm) of a deoxygenated 0.0125 M benzene solution of phenyl *N*-*tert*-butylnitrone containing **PC1** (0.05 M) and **B1** (0.05 M). Simulated profiles for acridine radical generated from **PC1** upon SET (red) and of the PBN*NHCBH_2_^•^ (blue) are also shown (see Section 6.1 in the Supporting Information for experimental and simulation parameters). (B) UV–vis spectra of a deoxygenated CH_3_CN solution of **PC1** and **B1** (both 10^–5^ M) irradiated over 4.25 min. (C) Deuteration experiments. (D) Determination of the kinetic isotope effect (KIE). (E) Computational investigation.

To gain insight into the reaction mechanism, we performed a series of experimental and computational studies. In particular, we identified two crucial steps to be investigated, i.e. (i) the generation of the ligated boryl radical^22^ and (ii) the occurrence of a radical chain process. To reveal the presence of the ligated boryl radical, we recorded an EPR spectrum of a deoxygenated benzene solution of **PC1** (0.05 M) and **B1** (0.05 M) containing phenyl *N*-*tert*-butylnitrone (PBN, 0.0125 M) as a spin trap. Prior to irradiation no signal was observed; however, after continuous irradiation for 15 min (λ = 460 nm) we clearly saw the appearance of two distinct features: one can be attributed to the reduced photocatalyst (acridine radical),^23^ while the second feature is derived from the trapping of the ligated boryl radical with PBN (Figure 4a; for further details, see Section 6.1 in the Supporting Information).^15a^ The assignment is further supported by the observation of the adduct of the LBR with PBN (PBN*NHCBH_2_^•^) in HRMS (Figure S5). These experiments unequivocally show the formation of the desired LBR under photoredox conditions. Direct observation of the ligated boryl radical via EPR was unsuccessful due to its short lifetime.^24^ As further evidence, we also tracked the interaction between **PC1** and **B1** via UV–vis spectroscopy. In particular, when a deoxygenated CH_3_CN solution (10^–5^ M) of **PC1** and **B1** was irradiated at 456 nm, we observed that the former was directly converted into a species absorbing in the 450–700 nm range (two isosbestic points were discerned; see Figure 4b). We propose this species to be the acridine radical generated upon single-electron reduction by **B1**, based on a comparison with the literature for a similar compound.^25^

In order to get some insights into the radical chain mechanism, we conducted experiments with deuterium-labeled substrates, and the results are collected in Figure 4c. The deuterium incorporation was calculated via ^1^H NMR on purified products. Overall, these experiments showed that deuterium incorporation (product **3-***d*_**1**_) was only observed when deuterated borane **B1-***d*_**3**_ was exploited as an XAT agent, while the use of **B1** resulted in the formation of product **3** exclusively.

These results reveal that the hydroalkylated product is obtained upon HAT from another molecule of **B1** rather than the solvent, thus pointing toward a radical chain mechanism. We next wondered if the latter could be the rate-determining step of the transformation; therefore, we set off to evaluate the kinetic isotope effect (KIE) of the reaction (Figure 4d). First, we measured the KIE through a competition experiment. Hereto, we performed our benchmark reaction in the presence of an equimolar mixture of **B1** and **B1-***d*_**3**_ (5 equiv each) and a KIE value of 3.5 was found. However, when we calculated this value with the parallel reactions method by performing independent reactions under optimized conditions, one containing **B1** and one containing **B1-***d*_**3**_, we found a KIE of only 1.09. Taken together, these experiments suggest that the HAT step is not involved in the rate-determining step of the reaction.^26^ Finally, we determined a quantum yield of 2%. Such a modest value is in accordance with a process being supported by either short-lived radical chain propagations, an inefficient initiation process^27^ or decomposition of the photocatalyst (further mechanistic insights are reported in Section 6 of the Supporting Information).^28^

We also performed a computational investigation intended to model the entire reaction profile through the simulation of all the key steps, including some possible parasitic pathways. Thus, we adopted DFT at the ωB97xD/def2TZVP level of theory to optimize the relevant stationary points, also including the effect of the solvent through an implicit model (Figure 4e; see also Section 10 of the Supporting Information for further details). We started by considering LBR **I** reacting with iodocyclohexane **1a** through TS1 (Δ*G*^‡^ = +11.6 kcal·mol^–1^) to afford cyclohexyl radical **II**. This nucleophilic radical (**II**) adds subsequently onto dimethyl maleate **2b** through TS2 (Δ*G*^‡^ = +15.5 kcal·mol^–1^) to deliver radical adduct **III**. Next, the targeted hydroalkylated product **5** is formed through reaction of **III** with NHC-ligated borane **B1** via TS3 (Δ*G*^‡^ = +12.4 kcal·mol^–1^). Notably, all these steps are exergonic in nature, with Δ*G* values in the −11.9 to −17.4 kcal·mol^–1^ range.

In this intricate ballet of fleeting radical intermediates, we realized that a careful balance between the XAT, the radical addition, and the final HAT steps was crucial to avoid parasitic reaction pathways.^11a,11b,15b^ Accordingly, we also evaluated the possibility for these intermediates to undergo competitive, yet nonproductive pathways, including the direct addition of LBR **I** onto dimethyl maleate **2b** with formation of a new B–C bond (through TS4) and the reduction of the cyclohexyl radical **II** to cyclohexane (through TS5). However, our computational analysis revealed that both steps occur with higher activation energies and less negative energy gains compared to those describing the desired process. Intrigued by the lack of reactivity of organic bromides in our reaction, we also computed Δ*G* and Δ*G*^‡^ for the XAT step by LBR **I** for bromocyclohexane.

By comparing the results with those obtained for the iodo analogue **1a**, it seems that the difference in reactivity can be mainly attributed to kinetic factors (Δ*G*^‡^_R-I_ = +11.6 kcal·mol^–1^ vs Δ*G*^‡^_R-Br_ = +17.9 kcal·mol^–1^), as the process shows similar driving forces for both halides (Δ*G*_R-I_ = −13.0 kcal·mol^–1^ vs Δ*G*_R-Br_ = −13.8 kcal·mol^–1^).

Finally, we were interested in comparing quantitatively LBR **I** with other commonly used halogen abstractors, including α-aminoalkyl and (tris(trimethylsilyl)silyl radicals and a conventional tin-based XAT reagent (Me_3_Sn^**•**^). As depicted in Figure 4e, a clear trend emerges. On the one hand, the XAT step shows very low activation energies in the case of metalloidyl radicals (i.e., R_3_Si^•^ and R_3_Sn^•^; for the tin-based abstractor it is barrierless), while LBR **I** and the α-aminoalkyl radical display significant Δ*G*^‡^ values (+11.6 and 15.0 kcal·mol^–1^, respectively). On the other hand, from a thermodynamic point of view, the XAT process is highly exergonic for metalloidyl radicals, moderately exergonic for LBR **I**, and essentially thermoneutral for the α-amino radical. In the latter case, the formation of an iminium ion resulting from the elimination of iodide was invoked as the driving force for the whole process.^19a^

**Figure 5:**
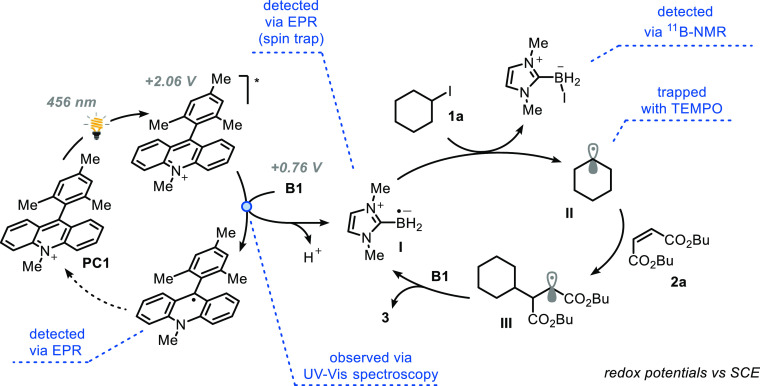
Proposed mechanism for the photoinduced XAT by *N*-heterocarbene boryl radicals for C–C bond formation. Redox potentials are reported vs SCE.

With both experimental and computational insights considered together, a mechanistic scenario is proposed in Figure 5. **PC1** absorbs light resulting in formation of the corresponding highly oxidizing excited state (*E*(PC*/PC_red_) = +2.06 V vs SCE).^29^ This excited state can react with **B1** (*E*_*pa*_(**B1**^**•+**^/**B1**) = +0.89 V vs (SCE) via single-electron transfer to afford LBR **I** upon deprotonation. The latter intermediate is entrusted with the desired XAT step, which is expected to be relatively fast (*k* ≈ 10^8^ M^–1^ s^–1^),^30^ thus yielding the alkyl radical **II**. This radical can be subsequently trapped by the electron-poor olefin to give adduct **III**. The observed inhibition effect of O_2_ (Table 1, entries 10–11) can be explained by taking into account that LBR **I** is known to react even faster with molecular oxygen (*k* > 10^8^ M^–1^ s^–1^),^30^ thus confiscating this crucial intermediate for the XAT event. Alternatively, it has been reported that molecular oxygen can also quench the excited state of **PC1** at diffusion controlled rates to form singlet oxygen (*k* = 2 × 10^9^ M^–1^ s^–1^).^31^ Next, as shown by the deuterium labeling experiments, **III** abstracts a hydrogen atom from **B1** in a polarity-matched step to give product **3** and subsequently kicks off the chain propagation.^32^

## Conclusions

In conclusion, we have shown that *N-*heterocyclic carbene (NHC) borane **B1** is an efficient XAT agent to sustain the radical hydroalkylation of olefins. This is a significant advancement to previous reports where NHC-ligated boryl radicals were mainly used as radical chain carriers for radical reductions. Our method shows remarkable generality, robustness, and versatility, as it does not rely on any interaction between the ligated borane and the organic halide to generate nucleophilic alkyl radicals under visible light irradiation. Due to the mild reaction conditions, it is applicable to a vast array of substrates, including biologically active compounds. And finally, continuous-flow technology can be exploited to accelerate and scale our methodology.

A detailed experimental and spectroscopic mechanistic investigation describes the key role of the NHC-based boryl radicals in the operative reaction mechanism. This is further corroborated by computational analysis, indicating that the described process is the most favorable one with respect to possible competing pathways.

While NHC-ligated boryl radicals have been only recently exploited in radical chemistry, this investigation represents an important step toward the appreciation and the exploitation of the properties and the reactivity of these boryl species. Hence, we are confident that this work will stimulate further research into the use of LBRs for radical-based synthetic transformations.

## Supporting Information Available

Experimental procedures, characterization data of synthesized compounds, copies of NMR spectra, complete mechanistic investigation. The primary NMR FID files for starting materials, compounds **3**–**40**, as well as optimized geometries of species displayed in Figure 4e, IRC and PES profiles are available in the FigShare repository at https://www.doi.org/10.21942/uva.20459517. (PDF)
