# Supplementary material for: Photoinduced Halogen-Atom Transfer by N-Heterocyclic Carbene-Ligated Boryl Radicals for C(sp3)–C(sp3) Bond Formation
Source: J Am Chem Soc. 2022 Dec 30;145(2):991–9. doi: 10.1021/jacs.2c10444 (PMC9853867; doi:10.1021/jacs.2c10444)
Supplement: Supplementary file 1 — ja2c10444_si_001.pdf [file ja2c10444_si_001.pdf]

Supporting Information

Photoinduced Halogen-Atom Transfer by N-heterocyclic carbene-ligated boryl radicals for $C(sp^3)-C(sp^3)$ bond formation

Ting Wan,[†,1] Luca Capaldo,[†,1] Davide Ravelli,[2] Walter Vitullo,[1] Felix J. de Zwart,[3] Bas de Bruin,[3] Timothy Noël*,[1]

[1] Flow Chemistry Group, van 't Hoff Institute for Molecular Sciences (HIMS), University of Amsterdam, Science Park 904, 1098 XH Amsterdam, The Netherlands.

[2] PhotoGreen Lab, Department of Chemistry, University of Pavia, viale Taramelli 12, 27100 Pavia, Italy.

[3] Homogeneous, Supramolecular and Bio-inspired Catalysis Group (HomKat), van 't Hoff Institute for Molecular Sciences (HIMS), Universiteit van Amsterdam (UvA), Science Park 904, 1098 XH, Amsterdam, The Netherlands.

** Email: t.noel@uva.nl*

† These authors contributed equally.

1. General information **S5**

2. Reactor design **S7**

UFO reactor *S7*

Flow reactor *S8*

3. Charts of starting materials **S9**

Organic halides S9

Electron-poor olefins S9

4. Synthesis of starting materials **S10**

Synthesis of **B1** and **B1-*d*$_3$** S10

Synthesis of organic halides S11

Synthesis of electron-poor olefins S16

5. Optimization of reaction conditions **S17**

5.1 Optimization of conditions for reaction under visible-light irradiation . S17

5.1.1 Screening of photocatalysts *S17*

5.1.2 Screening of solvents *S18*

5.1.3 Screening of substrates ratio *S18*

5.1.4 Screening of atmosphere *S18*

5.1.5 Screening of reaction time and photocatalyst loading *S19*

5.1.6 Control experiments *S19*

*5.1.7 Screening of reaction condition with 4CzIPN (**PC2**)* *S19*

5.1.8 Alkyl bromides as radical sources *S20*

5.1.9 Adjustment of reaction conditions for continuous-flow *S20*

6. Mechanistic investigation **S22**

6.1 EPR experiments S22

6.2 UV-Vis spectroscopic analysis S23

Evidence for the absence of an EDA complex *S23*

6.3 Quantum yield measurements S26

6.4 Deuterium labelling experiments S29

6.4.1 Deuterated solvents S30

6.4.2 Deuterated ligated borane S32

6.4.3 Deuterated ligated borane and solvents S33

6.5 Evaluation of kinetic isotopic effect (KIE) S35

6.6 Radical quenching experiment with TEMPO S37

6.7 Competition experiments S38

6.8 Other experiments S38

Identification of by-products S38

Fate of the photocatalyst S40

6.9 Comparison with other XAT agents S42

7. General procedures (GPs) for preparative experiments S43

7.2 GP3: batch conditions, blue light (456 nm) for 3° and 2° alkyl iodides S43

7.3 GP4: batch conditions, blue light (456 nm) for 1° alkyl iodides S43

7.4 GP5: batch conditions, blue light (456 nm) for alkyl bromides S43

7.5 GP6: continuous flow conditions, blue light (450 nm) S43

8. Scale-up for the synthesis of compound 3 in continuous-flow S45

9. Characterization data S46

10. Computational Details S60

10.1 IRC Plots S64

10.2 Relaxed PES Scan S70

10.3 Optimized Structures S71

11. Cyclic voltammetry S97

12. References S98

13. NMR spectra S102

13.1 NMR spectra of starting materials S102

13.2 NMR spectra of products S123

1. General information

Reagents and consumables. All reagents and solvents were bought from Sigma Aldrich, TCI, Flurochem, VWR International and Biosolv and used as received. Disposable syringes were purchased from Laboratory Glass Specialist. Syringe pumps were purchased from Chemix Inc. model Fusion 200 Touch. All capillary tubing, microfluidic fittings and Back Pressure Regulator (BPR) were purchased from IDEX Health & Science. Product isolation was performed manually, using silica (P60, SILICYCLE), or automatically, by a Biotage® Isolation Four, with Biotage® SNAP KP-Sil 20 or 50 g flash chromatography cartridges. TLC analysis was performed using Silica on aluminum foils TLC plates (F254, SILICYCLE) with visualization under ultraviolet light (254 nm and 365 nm) or appropriate TLC staining (potassium permanganate or cerium ammonium molybdate).

NMR spectroscopy. ^{1}H (400 MHz or 300 MHz), ^{13}C (101 MHz or 75 MHz), ^{31}P (121 MHz) and ^{11}B (128 MHz or 96 MHz) spectra were recorded unless stated otherwise on ambient temperature using a Bruker AV400 or a Bruker AV300. ^{1}H NMR spectra are reported in parts per million (ppm) downfield relative to $CDCl_3$ (7.26 ppm) or CD_2Cl_2 (5.32 ppm) and all ^{13}C NMR spectra are reported in ppm relative to $CDCl_3$ (77.2 ppm) or CD_2Cl_2 (53.8 ppm) unless stated otherwise. The multiplicities of signals are designated by the following abbreviations: s (singlet), d (doublet), t (triplet), q (quartet), m (multiplet), dd (doublet of doublets), dt (doublet of triplets), td (triplet of doublets), tt (triplets of triplets), ddd (doublet of doublet of doublets), qd (quartet of doublet). Coupling constants (*J*) are reported in hertz (Hz). NMR data was processed using the MestReNova 14 software package. Known products were characterized by comparing to the corresponding ^{1}H NMR, ^{13}C NMR, ^{31}P NMR and ^{11}B NMR with those available in the literature.

Melting point. Melting points were measured using a Büchi Melting Point M-565 apparatus.

Mass spectrometry. High resolution mass spectra (HRMS) were collected on an AccuTOF LC, JMS-T100LP Mass spectrometer (JEOL, Japen).

UV-Vis spectroscopy. UV-Vis spectra were recorded with a double beam spectrophotometer Shimadzu UV2700 equipped with a deuterium lamp (190-350 nm), a halogen lamp (330-900 nm) and a photomultiplier (Hamamatsu R928). Measurements were performed in a quartz cuvette (optical path: 1 cm). All spectra were recorded in CH_3CN (solvent cutoff: 190 nm) in quartz cuvettes (optical path: 1 cm) with a bandwidth of 5 nm and a data pitch of 1 nm.

EPR measurements. EPR measurements were performed in air-tight J-Young quartz tubes in an atmosphere of purified argon. EPR spectra were recorded on a Bruker EMX-plus CW X-

band spectrometer equipped with a Bruker ER 4112HV-CF100 helium cryostat. The spectra were obtained on freshly prepared solutions of 1–10 mM compound and simulated using EasySpin[1] via the cwEPR[2] GUI. Solutions were irradiated by adopting a Spectra Tune Lab device (Channel 3, centered at 465 nm, 0.94 W, 74 lm, FWHM: 23 nm; see https://ledmotive.com)

2. Reactor design

UFO reactor

For all batch experiments a homemade, 3D-printed reactor was adopted. The reactor was designed to fit reaction vials and to be equipped with a Kessil lamp PR160L series (λ_{em} = 390 or 456 nm). The reactor was designed in Adobe Inventor 2021 with 4 different parts. The lid (100 mm × 12 mm) is designed to host up to 8 reactions vials and holds the Kessil lamp in the center (**Figure S1**A); a fan (SUNON DCLüfter 24 V; 50x50x15 Vapo RoHS) is mounted on the bottom of the reactor for cooling. The box is designed with holes to allow the air flow to escape the reactor and keep the temperature stable around 30−33 °C (**Figure S1**B), as measured by an external thermometer. A reflector is situated underneath the lamp and reflects the photons inside the box to have homogeneous light distribution (**Figure S1**C). Finally, the stirring plate adapter (**Figure S1**D) was added to fix the system on a stirring plate and provide homogeneous stirring (**Figure S1**E). It also spaces the reflector from the plate to ensure a continuous air flow from the top to the bottom of the system. All the inside surfaces were covered with reflective tape. An overview of the assembled reactor is shown in **Figure S2**.

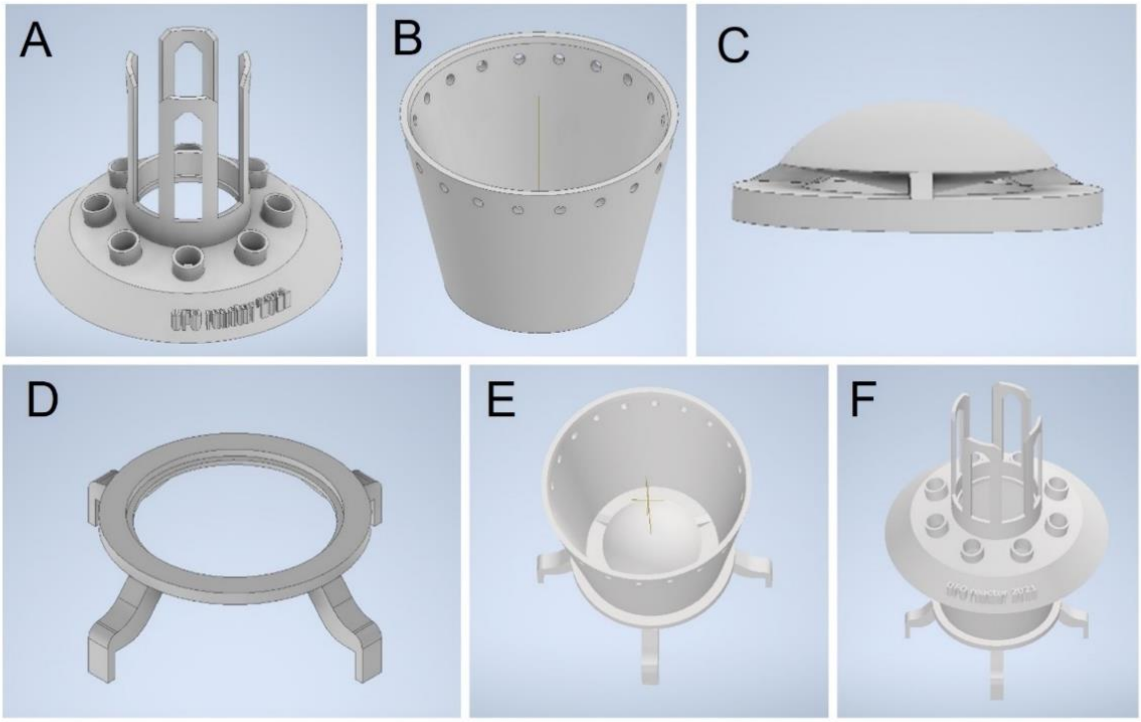

Figure S1: Overview of the 3D-printed reactor: A) lid designed to host up to 8 reactions vials and hold the Kessil lamp in the center; B) body of the reactor; C) light reflector: it is coated with reflective tape; D) adapter for stirring plate; E) inside of the reactor; G) overall reactor.

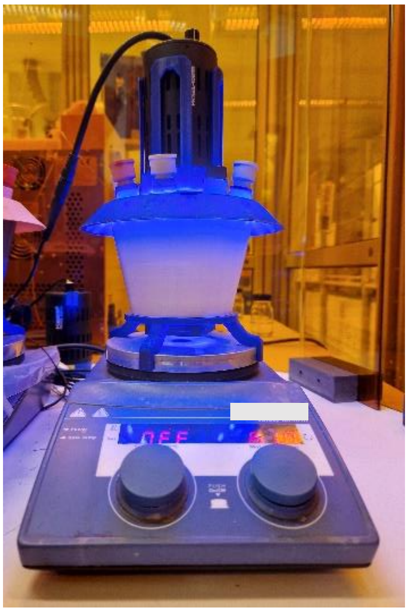

Figure S2: Picture of the assembled reactor equipped with a Kessil lamp (λ_{em} = 456 nm).

Flow reactor

For scale up, a commercially available Vapourtec UV-150 reactor was used (**Figure S3**), equipped with 60 W 450 nm LED.

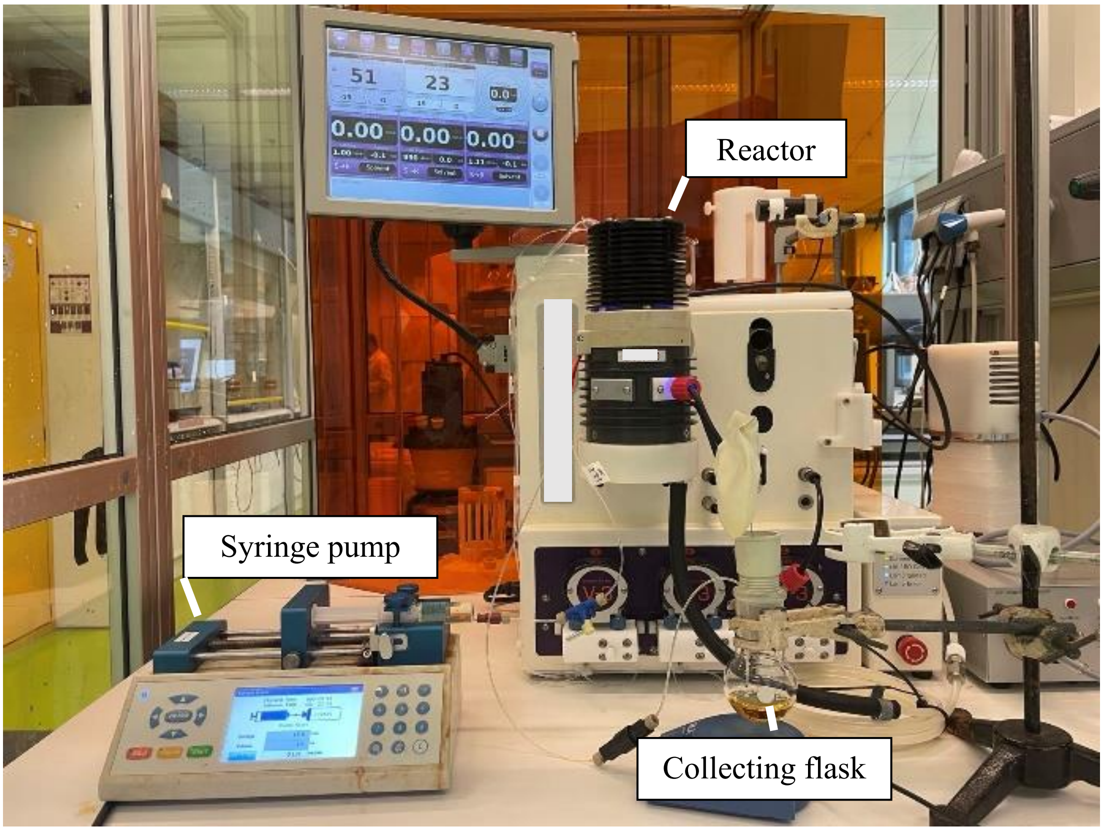

Figure S3: Picture of the Vapourtec UV-150 used in this work.

3. Charts of starting materials

Organic halides

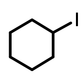
1a

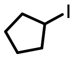
1b

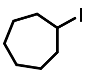
1c*

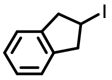
1d*

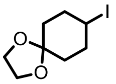
1e*

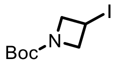

1f

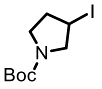

1g*

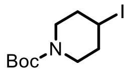

1h

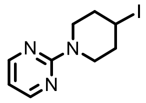
1i*

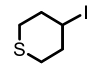
1j*

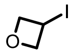
1k

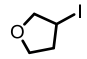
1l

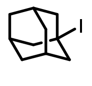
1m

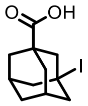
1n*

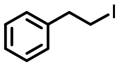
1o

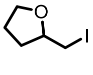
1p*

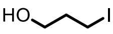

1q

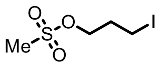

1r*

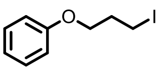
1s*

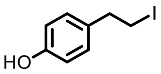

1t*

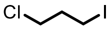

1u

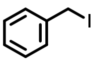
1v*

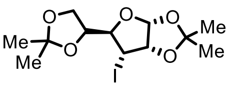

1w*

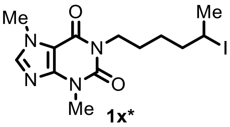

1x*

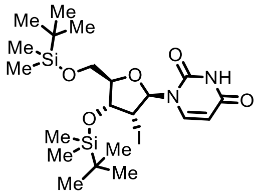

1y*

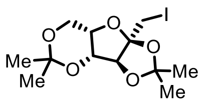

1z*

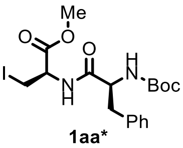

1aa*

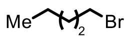

1ab

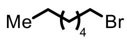

1ac

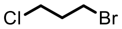

1ad

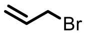

1ae

** Homemade*

Electron-poor olefins

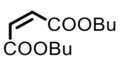

2a

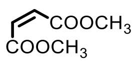

2b

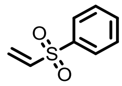
2c

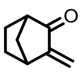
2d

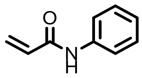
2e

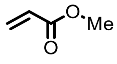

2f

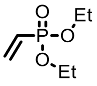

2g

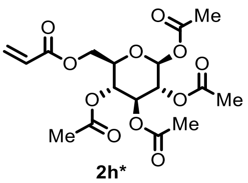

2h*

** Homemade*

4. Synthesis of starting materials

Synthesis of **B1** and **B1-*d*$_3$**

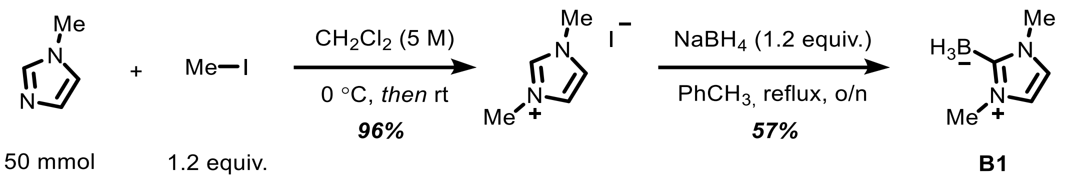

B1 was prepared by following a procedure reported in the literature.[3] At 0 °C, methyl iodide (1.2 equiv) was added dropwise to a CH_2Cl_2 solution of 1-methylimidazole (50 mmol, 5.0 M) over 30 minutes. The ice bath was removed and the reaction mixture was allowed to stir for 1 h at room temperature, after which the mixture was concentrated and dried under vacuum to give 1,3-dimethyl-1H-imidazol-3-ium iodide (10.8 g, 96%). Without purification, 1,3-dimethyl-1H-imidazol-3-ium iodide (10.8 g, 48 mmol) was suspended in toluene (1 mL/mmol) and sodium borohydride (1.2 equiv) was added in one portion. The mixture was refluxed overnight. The hot reaction solvent was decanted from the insoluble mixture, and the remaining residue was extracted with hot toluene (2×1 reaction volume), and concentrated under reduced pressure. Purification by flash chromatography on silica gel (Cyclohexane:Ethyl Acetate 20:80) to give **B1** as a colourless solid (3 g, 57%; 54% over two steps). Spectroscopic data are in accordance with the literature.[3]

^{1}H NMR (300 MHz, $CDCl_3$) δ 6.81 (s, 2H), 3.75 (s, 6H), 1.49 – 0.53 (m, 3H).

^{13}C NMR (75 MHz, $CDCl_3$) δ 120.0, 36.1. The signal of the α-B-carbon was not observed.

^{11}B NMR (96 MHz, $CDCl_3$) δ -37.5 (q, J = 86.3 Hz).

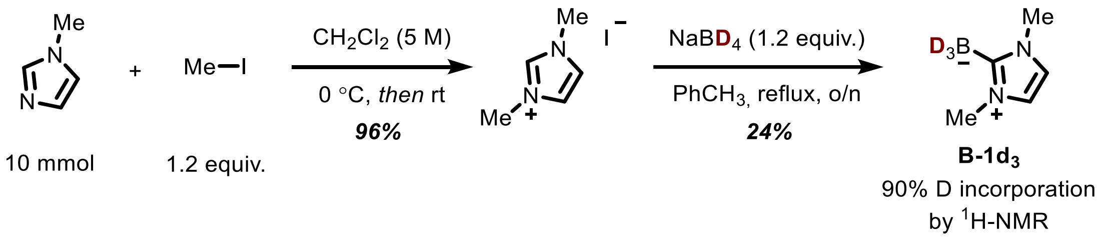

The same procedure described for **B1** was adopted for the synthesis of **B1-d$_3$** on a 10 mmol scale.[3] As far as the second step is concerned, sodium borodeuteride (1.2 equiv) was used instead of sodium borohydride. Purification by flash chromatography on silica gel (Cyclohexane:Ethyl Acetate 20:80) to give colourless solid **B1-*d*$_3$** (271 mg, 24%). A small amount of **B1** was also detected and quantified by ^{1}H-NMR (10%).

^{1}H NMR (400 MHz, $CDCl_3$) δ 6.79 (s, 2H), 3.72 (s, 6H).
^{13}C NMR (101 MHz, $CDCl_3$) δ 120.0, 36.0. The signal of the α-B-carbon was not observed.
^{11}B NMR (128 MHz, $CDCl_3$) δ -36.93 – -38.78 (m).

Synthesis of organic halides

General procedure 1 (GP 1)

Under inert atmosphere (N_2), PPh_3 (1.5 equiv.) and imidazole (1.5 equiv.) were dissolved in CH_2Cl_2 (0.25 mmol/mL) and the mixture was cooled at 0 °C by means of an ice bath. Next, I_2 (1.5 equiv.) was added and the resulting suspension was stirred for 30 min. Alcohol (1 equiv.) was added dropwise to the reaction mixture and the reaction crude was left stirring for 1 h. After the reaction was judged to be complete (via TLC), the reaction was quenched by the addition of H_2O; the aqueous layer was extracted twice with CH_2Cl_2 (2×0.75 reaction volume) and the combined organic layers were washed with an aqueous solution of sodium thiosulphate, dried over $MgSO_4$, filtered and concentrated under reduced pressure. The desired compound was obtained via flash chromatography on silica gel.[4]

General procedure 2 (GP 2)

To a solution of alcohol (1.0 equiv.) in toluene (0.1 mL/mmol) were added imidazole (3.0 equiv.), PPh_3 (2.0 equiv.) and I_2 (1.5 equiv.). The reaction mixture was refluxed for 4 h then quenched with a saturated aqueous solution of $NaHCO_3$ and extracted with Et_2O. I_2 was added to the combined organic phases until a persistent brown color. The organic phase was then washed with a saturated aqueous solution of $Na_2S_2O_3$, dried over Na_2SO_4, filtered and concentrated under reduced pressure. Purification by flash chromatography on silica gel.[5]

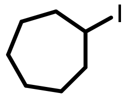

Iodocycloheptane (1c). Following **GP1** from cycloheptanol (0.75 g, 6.6 mmol). Colorless oil (0.9 g, 67%). ^{1}H NMR (300 MHz, $CDCl_3$) δ 4.48 (tt, J_1 = 9 Hz, J_2 = 4 Hz, 1H), 2.37 – 2.08 (m, 4H), 1.70 – 1.55 (m, 6H), 1.52 – 1.34 (m, 2H). ^{13}C NMR (75 MHz, $CDCl_3$) δ 42.2, 36.5, 27.5, 27.2. Spectroscopic data are in accordance with the literature.[6]

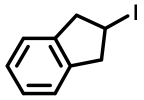

2-Iodo-2,3-dihydro-1H-indene (1d). Following **GP1** from 2,3-dihydro-1H-inden-2-ol (1.3 g, 10 mmol). Colourless

solid (2.0 g, 82%). ^{1}H NMR (400 MHz, $CDCl_3$) δ 7.34 – 7.21 (m, 4H), 4.74 (tt, J_1 = 6 Hz, J_2 = 5 Hz, 1H), 3.47 (qd, J_1 = 17 Hz, J_2 = 6 Hz, 4H). ^{13}C NMR (101 MHz, $CDCl_3$) δ 141.5, 127.0, 124.4, 46.7, 24.0. Spectroscopic data are in accordance with the literature.[7]

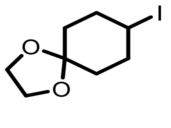

8-Iodo-1,4-dioxaspiro[4.5]decane (1e). Following **GP1** from 1,4-dioxaspiro[4.5]decan-8-ol (1.6 g, 10 mmol). Colorless oil (1.9 g, 73%). ^{1}H NMR (400 MHz, $CDCl_3$) δ 4.49 – 4.33 (m, 1H), 3.99 – 3.87 (m, 4H), 2.21 – 2.02 (m, 4H), 1.86 – 1.56 (m, 2H), 1.60 (ddd, J_1 = 13 Hz, J_2 = 8 Hz, J_3 = 4 Hz, 2H). ^{13}C NMR (101 MHz, $CDCl_3$) δ 107.6, 64.5, 64.4, 36.4, 34.9. Spectroscopic data are in accordance with the literature.[4]

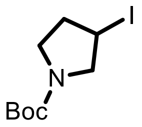

***tert*-Butyl 3-iodopyrrolidine-1-carboxylate (1g)**. Following **GP2** from *tert*-butyl 3-hydroxypyrrolidine-1-carboxylate (2.0 g, 10.7 mmol). Colorless oil (2.6 g, 80%). ^{1}H NMR (400 MHz, $CDCl_3$) δ 4.42 – 4.26 (m, 1H), 3.89 – 3.64 (m, 2H), 3.63 – 3.51 (m, 1H), 3.49 – 3.34 (m, 1H), 2.32 – 2.15 (m, 2H), 1.50 – 1.43 (m, 9H). ^{13}C NMR (101 MHz, $CDCl_3$) δ 154.3, 79.9, 57.5, 57.2, 45.2, 44.9, 38.5, 37.7, 28.6, 20.1. Spectroscopic data are in accordance with the literature.[5]

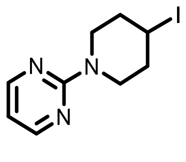

2-(4-Iodopiperidin-1-yl)pyrimidine (1i). Following **GP1** from 1-(pyrimidin-2-yl)piperidin-4-ol (1.5 g, 8.4 mmol). Yellow oil (1.9 g, 79%). ^{1}H NMR (300 MHz, $CDCl_3$) δ 8.30 (d, J = 5 Hz, 2H), 6.48 (t, J = 5 Hz, 1H), 4.63 – 4.48 (m, 1H), 4.15 – 4.01 (m, 2H), 3.68 – 3.55 (m, 2H), 2.20 – 2.05 (m, 4H). ^{13}C NMR (75 MHz, $CDCl_3$) δ 161.7, 157.9, 110.0, 44.3, 37.6, 28.7. Spectroscopic data are in accordance with the literature.[4]

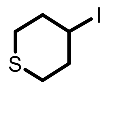

4-Iodotetrahydro-2H-thiopyran (1j). Following **GP1** from tetrahydro-2H-thiopyran-4-ol (1 g, 8.5 mmol). Yellow oil (1.14 g, 59%). ^{1}H NMR (300 MHz, $CDCl_3$) δ 4.55 – 4.42 (m,

1H), 2.86 – 2.74 (m, 2H), 2.63 – 2.49 (m, 2H), 2.42 – 2.22 (m, 4H). ^{13}C NMR (75 MHz, $CDCl_3$) δ 38.8, 31.0, 28.1. Spectroscopic data are in accordance with the literature.[8]

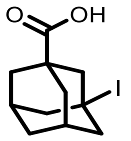

(1r,3s,5R,7S)-3-Iodoadamantane-1-carboxylic acid (1n). Prepared according a procedure reported in the literature. Spectroscopic data are in accordance with the literature.[9] ^{1}H NMR (400 MHz, $CDCl_3$) δ 2.74 (s, 2H), 2.63 – 2.50 (m, 4H), 2.09 – 2.03 (m, 2H), 2.02 – 1.92 (m, 4H), 1.82 – 1.74 (m, 2H). ^{13}C NMR (101 MHz, $CDCl_3$) δ 181.7, 52.3, 51.1 (2C), 45.7, 44.7, 37.0 (2C), 34.6, 32.0 (2C).

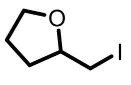

2-(Iodomethyl)tetrahydrofuran (1p). Following **GP1** from (tetrahydrofuran-2-yl)methanol (1 g, 9.8 mmol). Light yellow oil (0.9 g, 43%). ^{1}H NMR (400 MHz, CD_2Cl_2) δ 4.04 – 3.90 (m, 2H), 3.82 (td, J_1 = 8 Hz, J_2 = 6 Hz, 1H), 3.22 (qd, J_1 = 10 Hz, J_2 = 6 Hz, 2H), 2.17 – 2.05 (m, 1H), 2.04 – 1.85 (m, 2H), 1.72 – 1.59 (m, 1H). ^{13}C NMR (101 MHz, CD_2Cl_2) δ 78.6, 69.1, 32.0, 26.2, 10.6. Spectroscopic data are in accordance with the literature.[10]

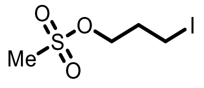

3-iodopropyl methanesulfonate (1r). Prepared by adapting a procedure reported in the literature[11]. Triethylamine (2 eq) and methanesulfonyl chloride (2 eq) were added dropwise to a solution of 3-iodopropan-1-ol (0.4 g, 2 mmol) in dry DCM (20 ml) under inert atmosphere (N_2). The reaction mixture was stirred overnight at room temperature and then quenched with sat. NH_4Cl (20 ml) solution and extracted with DCM (2 × 20 ml). The combined organic layers were dried over Na_2SO_4 and filtered. The solvent was evaporated to give **1ab** as a yellowish oil (456 mg, 90%), which was used without any further purification.

^{1}H NMR (300 MHz, $CDCl_3$) δ 4.31 (t, J = 6 Hz, 1H), 3.27 (t, J = 7 Hz, 1H), 3.04 (s, 2H), 2.22 (ddd, J_1 = 12 Hz, J_2 = 7 Hz, J_1 = 6 Hz, 1H). ^{13}C NMR (75 MHz, $CDCl_3$) δ 69.4, 37.5,

32.5, 0.7. HRMS (FI) m/z calcd for $C_4H_9IO_3S^+$: $[M]^+$ 263.9314; found: 263.9317.

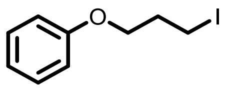

(3-Iodopropoxy)benzene (1s). Following **GP1** from 3-phenoxypropan-1-ol (1.5 g, 9.9 mmol). Colorless oil (2.1 g, 81%). ^{1}H NMR (400 MHz, $CDCl_3$) δ 7.37 – 7.29 (m, 2H), 7.04 – 6.97 (m, 1H), 6.97 – 6.90 (m, 2H), 4.06 (t, *J* = 6 Hz, 2H), 3.40 (t, *J* = 7 Hz, 2H), 2.35 – 2.25 (m, 2H). ^{13}C NMR (101 MHz, $CDCl_3$) δ 158.7, 129.6, 121.0, 114.6, 67.2, 33.1, 2.7. Spectroscopic data are in accordance with the literature.[12]

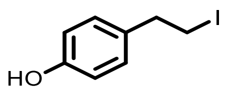

4-(2-Iodoethyl)phenol (1t). Following **GP1** from 4-(2-hydroxyethyl)phenol (1.4 g, 10 mmol). Colourless solid (1.8 g, 73%). ^{1}H NMR (300 MHz, CD_3CN) δ 7.12 – 7.01 (m, 2H), 6.82 (s, 1H), 6.78 – 6.70 (m, 2H), 3.37 (t, *J* = 8 Hz, 2H), 3.05 (t, *J* = 8 Hz, 2H). ^{13}C NMR (75 MHz, CD_3CN) δ 156.6, 133.2, 130.5, 116.1, 39.9, 8.4. Spectroscopic data are in accordance with the literature.[13]

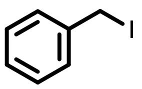

(Iodomethyl)benzene (1v). Following **GP1** from benzyl alcohol (1 g, 9.2 mmol). Colorless oil (0.9 g, 44%). ^{1}H NMR (300 MHz, $CDCl_3$) δ 7.47 – 7.39 (m, 2H), 7.38 – 7.24 (m, 3H), 4.50 (s, 2H). ^{13}C NMR (75 MHz, $CDCl_3$) δ 139.4, 128.9, 128.9, 128.9, 5.9. Spectroscopic data are in accordance with the literature.[14]

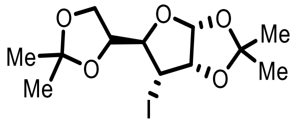

(3aR,5R,6R,6aS)-5-((R)-2,2-dimethyl-1,3-dioxolan-4-yl)-6-iodo-2,2-dimethyltetrahydrofuro[2,3-*d*][1,3]dioxole (1w). Following **GP2** from 1,2:5,6-di-*O*-isopropylidene-α-D-glucofuranose (2.0 g, 7.7 mmol) gave colorless oil (0.8 g, 28%). ^{1}H NMR (400 MHz, $CDCl_3$) δ 5.79 (d, *J* = 4 Hz, 1H), 4.58 (t, *J* = 4 Hz, 1H), 4.33 – 4.27 (m, 1H), 4.23 (dd, J_1 = 10 Hz, J_2 = 4 Hz, 1H), 4.10 (dd, J_1 = 8 Hz, J_2 = 6 Hz, 1H), 4.04 (dd, J_1 = 8 Hz, J_2 = 7 Hz, 1H), 3.74 (dd, J_1 = 10 Hz, J_2 = 4 Hz, 1H), 1.54 (s, 3H), 1.47 (s, 3H), 1.35 (s, 6H). ^{13}C NMR (101 MHz, $CDCl_3$) δ 111.8, 110.1, 103.2, 81.8, 81.6, 75.6,

65.9, 26.7, 26.7, 26.5, 25.3, 19.3. Spectroscopic data are in accordance with the literature.[15]

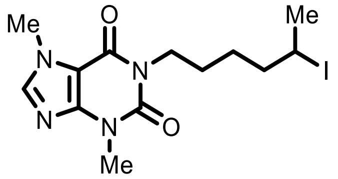

1-(5-Iodohexyl)-3,7-dimethyl-3,7-dihydro-1H-purine-2,6-dione (1x). Following **GP1** from 1-(5-hydroxyhexyl)-3,7-dimethyl-3,7-dihydro-1H-purine-2,6-dione (1 g, 3.6 mmol) gave a colourless deliquescent solid (1.1 g, 78%). ^{1}H NMR (400 MHz, $CDCl_3$) δ 7.49 (s, 1H), 4.24 – 4.11 (m, 1H), 4.03 – 3.94 (m, 2H), 3.98 (s, 3H), 3.56 (s, 3H), 1.90 (d, J = 7 Hz, 3H), 1.93 – 1.82 (m, 1H), 1.71 – 1.38 (m, 5H). ^{13}C NMR (101 MHz, $CDCl_3$) δ 155.4, 151.6, 148.9, 141.5, 107.8, 42.5, 41.2, 33.7, 30.2, 29.8, 29.0, 27.2 (2C). HRMS (ESI) m/z calcd for $C_{13}H_{19}IN_4O_2^+$: $[M+H]^+$ 391.0625; found: 391.0629.

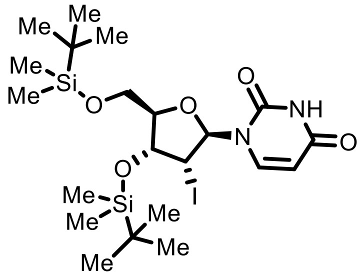

1-((2R,3R,4R,5R)-4-((tert-butyldimethylsilyl)oxy)-5-(((tert-butyldimethylsilyl)oxy)methyl)-3-iodotetrahydrofuran-2-yl)pyrimidine-2,4(1H,3H)-dione (1y). Prepared by following a procedure reported in the literature.[16] ^{1}H NMR (300 MHz, $CDCl_3$) δ 8.26 (s, 1H), 7.84 (d, J = 8 Hz, 1H), 6.38 (d, J = 6 Hz, 1H), 5.70 (dd, J_1 = 8 Hz, J_2 = 2 Hz, 1H), 4.29 (dd, J_1 = 6 Hz, J_2 = 5 Hz, 1H), 4.12 (dt, J_1 = 4 Hz, J_2 = 2 Hz, 1H), 3.95 (dd, J_1 = 12 Hz, J_2 = 2 Hz, 1H), 3.85 (dd, J_1 = 5 Hz, J_2 = 4 Hz, 1H), 3.76 (dd, J_1 = 12 Hz, J_2 = 2 Hz, 1H), 0.95 – 0.92 (m, 18H), 0.22 – 0.07 (m, 12H). ^{13}C NMR (75 MHz, $CDCl_3$) δ 162.7, 150.2, 139.5, 102.8, 91.3, 86.1, 71.5, 62.2, 32.2, 26.1, 25.9, 18.5, 18.3, -4.2, -4.4, -5.4, -5.4.

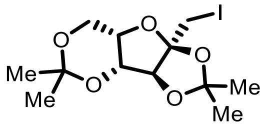

(3aS,3bR,7aS,8aR)-8a-(Iodomethyl)-2,2,5,5-tetramethyltetrahydro-7H-[1,3]dioxolo[4',5':4,5]furo[3,2-d][1,3]dioxine (1z). Prepared by following a procedure reported in the literature.[17] ^{1}H NMR (300 MHz, $CDCl_3$) δ 4.49 (s, 1H), 4.34 (dd, J_1 = 3 Hz, J_2 = 1 Hz, 1H), 4.17 (q, J = 2 Hz, 1H), 4.08 – 3.96 (m, 2H), 3.66 (d, J = 11 Hz, 1H), 3.54 (d, J = 11 Hz, 1H), 1.50 (s, 3H), 1.46 (s, 3H), 1.42 (s, 3H), 1.38 (s, 3H). ^{13}C

NMR (75 MHz, $CDCl_3$) δ 112.9, 112.7, 97.6, 85.7, 73.7, 73.2, 60.6, 29.0, 27.9, 27.0, 18.9, 7.0. Spectroscopic data are in accordance with the literature.[18]

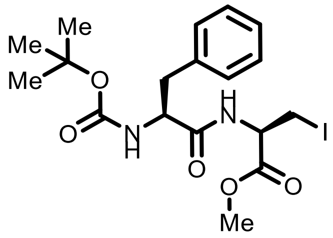

Methyl (R)-2-((S)-2-((tert-butoxycarbonyl)amino)-3-phenylpropanamido)-3-iodopropanoate (1aa). Following **GP1** from methyl (*tert*-butoxycarbonyl)-*D*-phenylalanyl-*L*-serinate[19] (2.2 g, 6 mmol) gave colorless oil (1.8 g, 62%). ^1H NMR (300 MHz, $CDCl_3$) δ 7.41 – 7.15 (m, 5H), 6.93 – 6.71 (m, 1H), 5.09 – 4.84 (m, 1H), 4.73 (dt, J_1 = 7 Hz, J_2 = 4 Hz, 1H), 4.54 – 4.33 (m, 1H), 3.80 (s, 3H), 3.66 – 3.51 (m, 2H), 3.21 – 3.02 (m, 2H), 1.44 (s, 9H). ^{13}C NMR (75 MHz, $CDCl_3$) δ 171.3, 169.5, 155.4, 136.4, 129.4, 128.9, 127.2, 80.6, 55.8, 53.2, 52.7, 38.1, 28.4, 6.7. Spectroscopic data are in accordance with the literature.[19]

Synthesis of electron-poor olefins

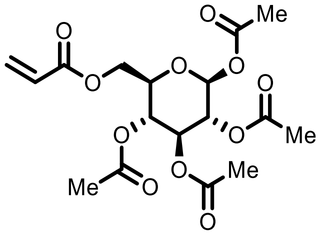

(2S,3R,4S,5R,6R)-6-((acryloyloxy)methyl)tetrahydro-2H-pyran-2,3,4,5-tetrayl tetraacetate (2h). Prepared by following a procedure reported in the literature.[20] ^1H NMR (300 MHz, $CDCl_3$) δ 6.44 (dd, J_1 = 17 Hz, J_2 = 1 Hz, 1H), 6.14 (dd, J_1 = 17 Hz, J_2 = 10 Hz, 1H), 5.88 (dd, J_1 = 10 Hz, J_2 = 1 Hz, 1H), 5.72 (d, J = 8 Hz, 1H), 5.32 – 5.20 (m, 1H), 5.20 – 5.07 (m, 2H), 4.35 – 4.20 (m, 2H), 3.95 – 3.83 (m, 1H), 2.11 (s, 3H), 2.03 (s, 6H), 2.01 (s, 3H). ^{13}C NMR (75 MHz, $CDCl_3$) δ 170.1, 169.3, 169.2, 168.9, 165.6, 131.8, 127.6, 91.7, 72.8, 72.7, 70.2, 67.9, 61.7, 20.8, 20.5 (3C).

5. Optimization of reaction conditions

5.1 Optimization of conditions for reaction under visible-light irradiation

The optimization of the reaction conditions was carried out by studying the radical addition of iodocyclohexane (**1a**) onto dibutyl maleate (**2a**) to give dibutyl 2-cyclohexylsuccinate (**3**) on a 0.1 mmol scale (see Table S1-13).

In a 7 mL vial equipped with a screw cap **2a** (0.1 mmol), **1a** (*n* equiv.), **B1** (*n* equiv.) and the chosen photocatalyst (*n* mol%) were dissolved in 1.0 mL of the chosen solvent. The mixture was bubbled with N_2 (1 min) and irradiated for the indicated time with a 40 W Kessil lamp (λ = 456 nm, full intensity) for the required time in the UFO reactor. After irradiation, 1.0 mL of a standard solution of biphenyl (0.1 M in EtOAc) was added to the reaction crude and 100 μl of the obtained mixture were added to a GC vial and further diluted with 900 μL of EtOAc. The resulting solution was filtered through a short silica plug in a Pasteur pipette and the filtrate analyzed via GC-MS. Yields were calculated by means of calibration curves built with authentic samples.

5.1.1 Screening of photocatalysts

Table S1: Screening of photocatalysts.

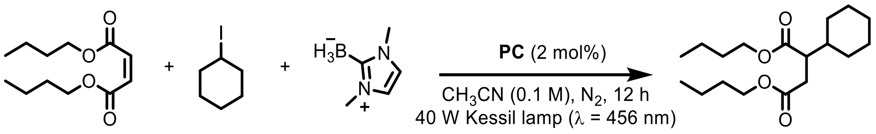

2a 0.1 mmol | **1a** 2 equiv. | **B1** 1 equiv. | **3**

Entry	Photocatalyst	Yield (%)[a]
1	**PC1**	44
2	**PC2**	43
3	**PC3**	28
4	**PC4**	25

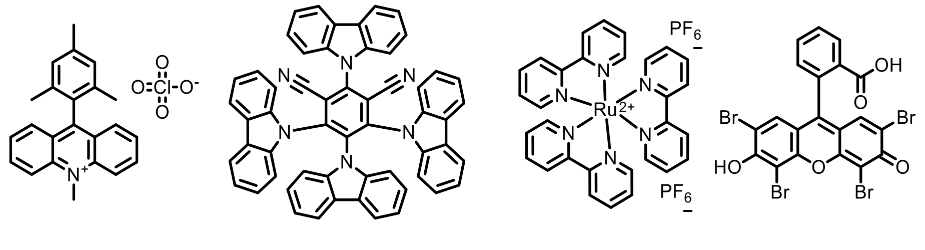

PC1: Mes-AcrClO$_4$ **PC2**: 4CzIPN **PC3**: Ru(bpy)$_3$(PF$_6$)$_2$ **PC4**: Eosin Y

[a] Yields determined by GC-MS, biphenyl as external standard.

5.1.2 Screening of solvents

Table S2: Screening of solvents.

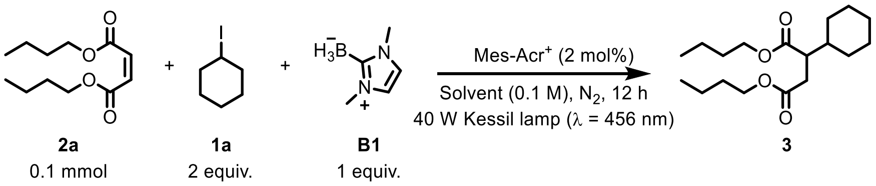

Entry	Solvent	Yield (%)[a]
1	CH_3CN	44
2	CH_3CN/H_2O (9:1)	68
3	CH_3OH	61
4	*t*BuOH	62
5	THF	62
6	DCM	39
7	Toluene	60

[a] Yields determined by GC-MS, biphenyl as external standard.

5.1.3 Screening of substrates ratio

Table S3: Screening of substrates ratio.

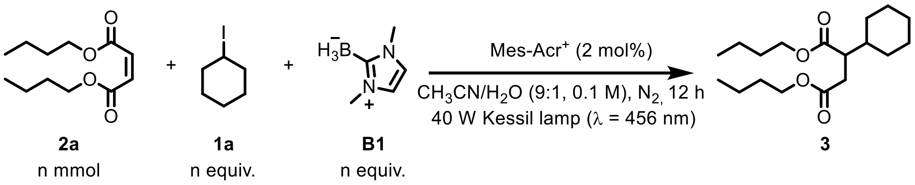

Entry	Substrates ratio (**2a**:**1a**:**B1**)	Yield (%)[a]
1	1:2:1	68
2	1:1.5:1	63
3	1:1:1	40
4	1.2:1:1	53
5	1:2:0.5	39
6	1:2:1.2	78

[a] Yields determined by GC-MS, biphenyl as external standard.

5.1.4 Screening of atmosphere

Table S4: Screening of atmosphere.

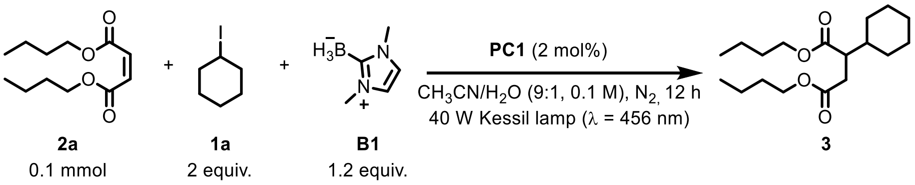

Entry	Atmosphere	Yield (%)[a]
1	N_2	78
2	Air	37
3	O_2	n.d.[b]

[a] Yields determined by GC-MS, biphenyl as external standard. [b] conversion of **2a**: 35%.

5.1.5 Screening of reaction time and photocatalyst loading

Table S5: Screening of reaction time and photocatalyst loading.

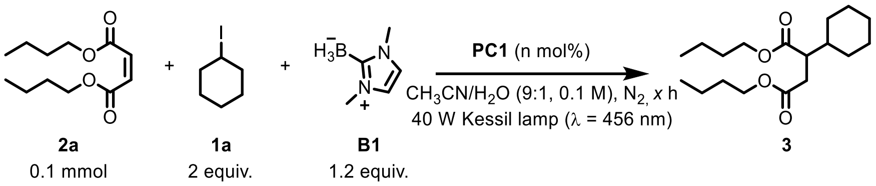

Entry	Variation from conditions	Yield (%)[a]
1	**PC1** (2 mol%), 12 h	78
2	**PC1** (2 mol%), 3 h	65
3	**PC1** (5 mol%), 3 h	79

[a] Yields determined by GC-MS, biphenyl as external standard.

5.1.6 Control experiments

Table S6: Control experiments.

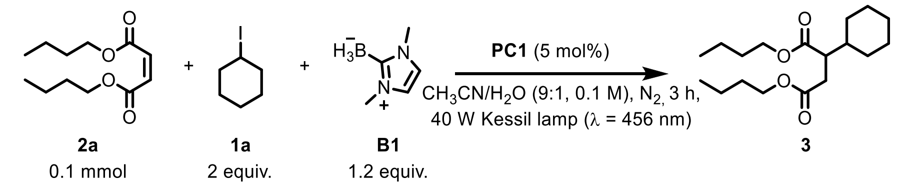

Entry	Variation from conditions	Yield (%)[a]
1	Without light	n.d.
2	Without **PC1**	n.d.
3	Without **B1**	n.d.
4	Heating at 80 °C in the dark	n.d.
5	Pyridine-BH_3	n.d.
6	Me_3N-BH_3	n.d.
7	*t*$BuMe_2P$-BH_3	n.d.

[a] Yields determined by GC-MS, biphenyl as external standard.

*5.1.7 Screening of reaction condition with 4CzIPN (**PC2**)*

Table S7: Screening of solvents with **PC2**.

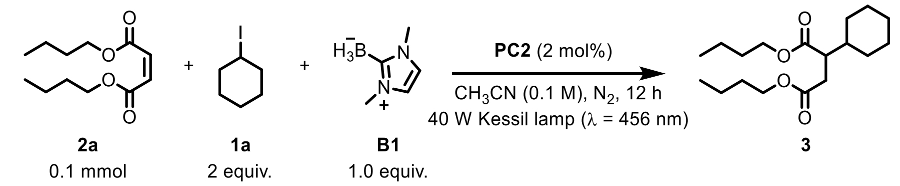

Entry	Variation from conditions	Yield (%)[a]
1	-	43
2	DMSO	72
3	CH_3CN/H_2O 9:1	75

4	CH_2Cl_2	26
5	EtOAc	67
6	As entry 3, 5 mol% **PC2**	68

[a] Yields determined by GC-MS, biphenyl as external standard.

5.1.8 Alkyl bromides as radical sources

Table S8: Use of alkyl bromides in optimized conditions.

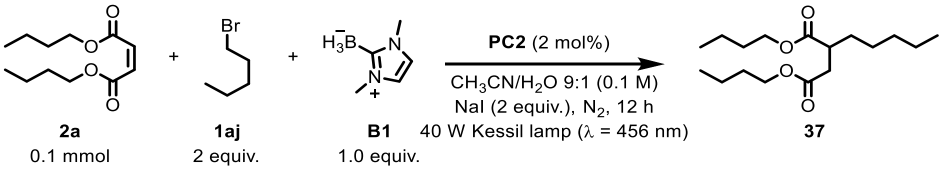

Entry	Conditions	Yield (%)[a]
1	**PC1** (5 mol%)	37
2	**PC2** (2 mol%), **B1** (1.2 equiv.)	52

[a] Yields determined by ^{1}H-NMR, CH_2Br_2 as external standard.

5.1.9 Adjustment of reaction conditions for continuous-flow

A solution of **2a** (*n* mmol), **1a** (2 equiv.), **B1** (1.2 equiv.) and **PC1** (5 mol%) in 1.0 mL of the indicated solvent was prepared and bubbled with N_2 (1 min). The liquid was taken up with a syringe and mounted on a syringe pump. The syringe was connected to a 3.18 mL reactor (PFA capillary tubing, 0.8 mm inner diameter) and the mixture was pumped into the flow reactor (Vapourtec UV-150, λ = 450 nm, 60 W) at 0.106 mL/min (corresponding to 30 minutes residence time). When the syringe was fully empty, pure CH_3CN was loaded into a syringe and used to push the reaction mixture all the way through the reactor. As during the reaction we observed the generation of gas, a back pressure regulator (BPR, 2.8 bar) was connected at the outlet to have a stable residence time. The crude was collected at the end of the reactor directly in a flask, external standard biphenyl (0.1 mmol) was added in reaction solution. Solution was processed as in Section 5.1 for quantitative analysis.

Table S9: Screening of reaction conditions in continuous-flow.

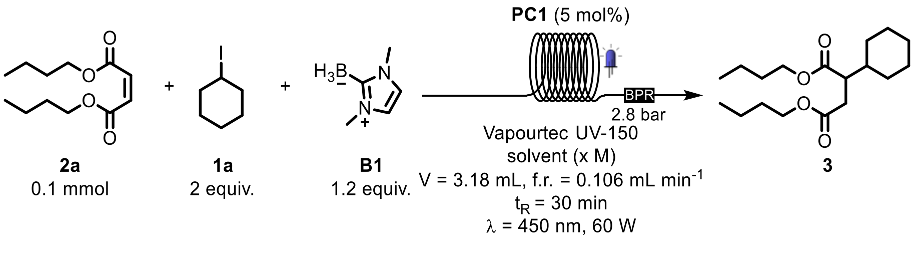

Entry	Condition	Yield (%)[a]
1	0.1 M, rt, 30 min	56
2	0.1 M, 48~50 °C, 30 min	65
3	0.5 M, 48~50 °C, 30 min	64

4	DMSO (0.5 M), 48~50 ºC, 30 min	83

[a] Yields determined by GC-MS, biphenyl as external standard.

6. Mechanistic investigation

6.1 EPR experiments

In order to prove the generation of the ligated boryl radical, we performed a spin trapping experiment (**Figure S4**).

Thus, in a nitrogen filled glovebox, **PC1** (0.05 mmol), **B1** (0.05 mmol), and phenyl *N-tert*-butylnitrone (PBN, 0.0125 mmol) were dissolved in benzene (1 mL) and filtered into a J. Young valve EPR tube. Prior to irradiation, this provided no EPR signal. At room temperature the EPR sample was irradiated with 460 nm light for 15 minutes in the EPR cavity using an optical fiber and measured to provide the following spectrum (MW freq. = 9.6441, MW power = 6.325 mW, Mod. amp. = 0.1 G):

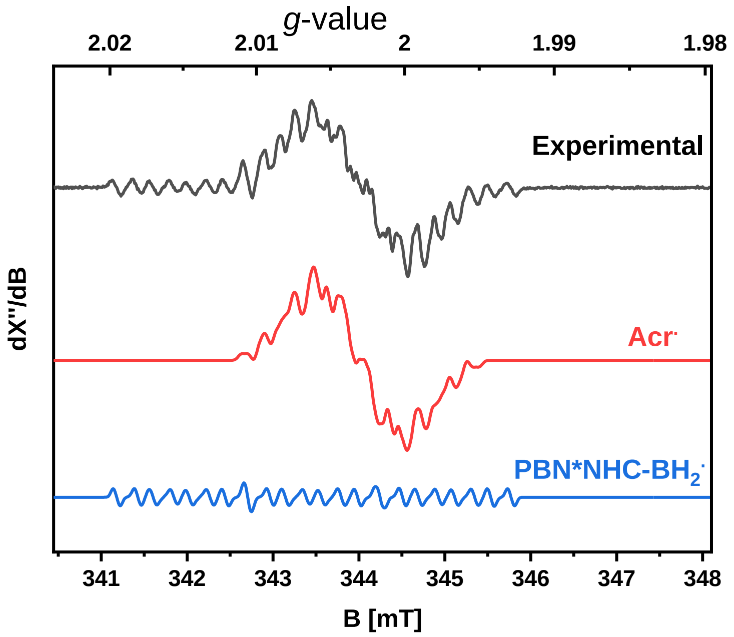

Figure S4: Experimental EPR spectrum (top) obtained upon irradiation (λ = 460 nm) of a mixture of **PC1**, **B1** and PBN (4:4:1) in benzene. The simulated spectra for the acridinyl radical·(red) and PBN*NHC-$BH_2^•$ adduct (blue) are also reported.

Simulation of this spectrum, using EasySpin via the cwEPR GUI, provided a satisfactory fit to two components. One component can be assigned to the neutral acridinium radical, based on the hyperfine coupling to four sets inequivalent protons (A_{H1} = 9.55 MHz, A_{H345} = 5.6 MHz, A_{H67} = 10.7 MHz, A_{H89} = 2.1 MHz) and one nitrogen hyperfine coupling (A_{N1} = 7.0 MHz) which is in accordance with literature.[21] The other component can be assigned to the PBN adduct of an NHC-$BH_2^•$ radical, based on a hyperfine interaction with the nitrogen and hydrogen of the spin trap (A_N = 43.3 MHz, A_H = 6.9 MHz) and a borane coupling of 11.8 MHz,

strongly indicating the formation of a boryl radical as this is in accordance with literature.[22] The assignment is further supported by the observation of the PBN*NHC-$BH_2^{\bullet}$ adduct in HRMS (**Figure S5**).

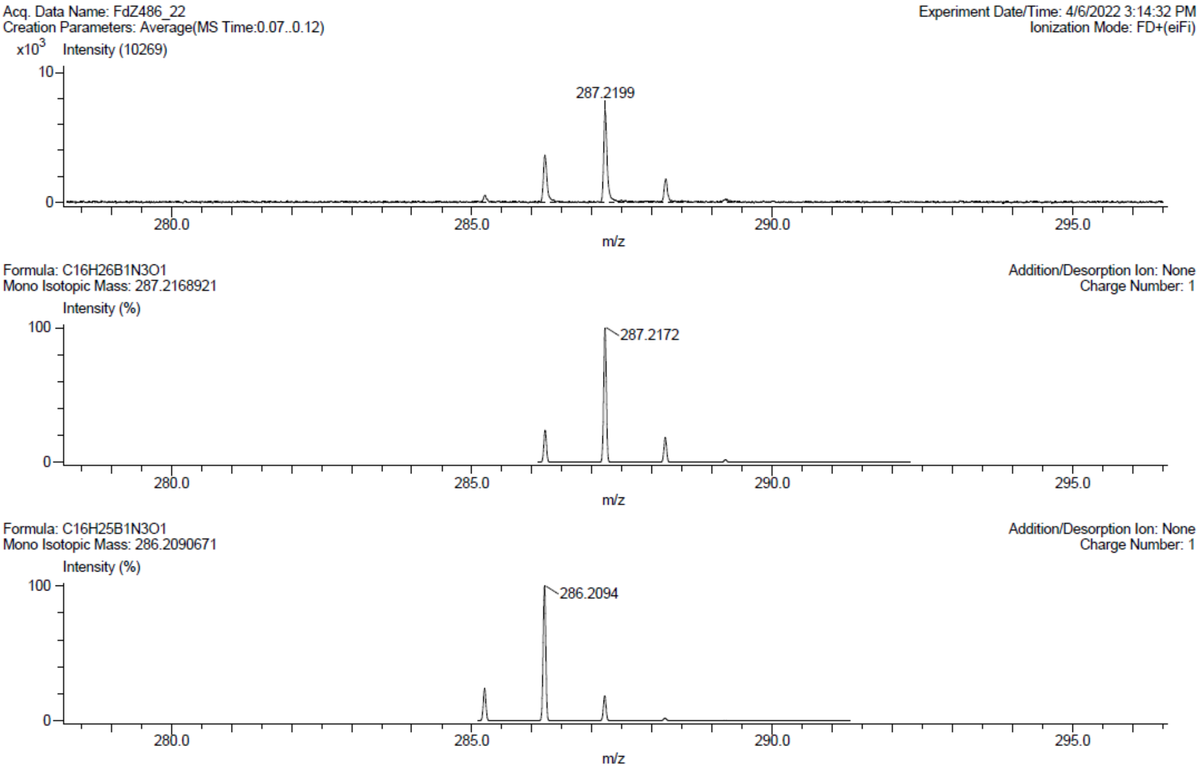

Figure S5: Top: acquired HRMS (FD) spectrum of the crude obtained after the spin trapping experiment; middle: simulated spectrum for a [PBNH * $NHCBH_2]^{\bullet}$; bottom: simulated spectrum for a [PBN * $NHCBH_2]^{\bullet}$ adduct (bottom).

6.2 UV-Vis spectroscopic analysis

Evidence for the absence of an EDA complex

Prompted by Dilman's report on the radical silyldifluoromethylation of electron-deficient alkenes,[23] where a halogen bonding responsible for weakening the C−I bond was claimed, we decided to investigate ground state interactions between the reactants in our mixture. In particular, we started by recording an absorption spectrum of single components (**1a**, **2b**, **B1**) and that of a mixture of **1a** and **B1** to seek for the formation of, e.g., an EDA complex (**Figure S6**).

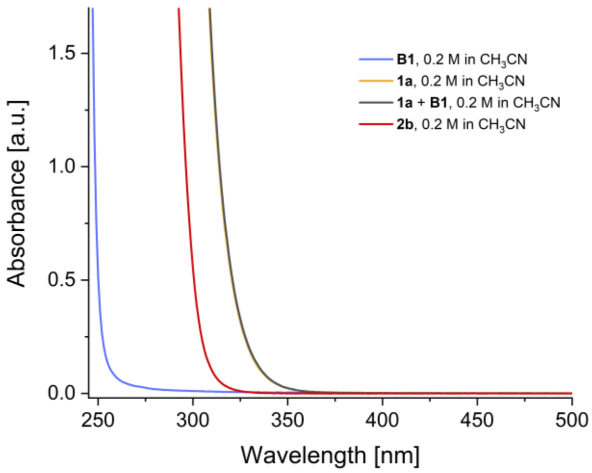

Figure S6: Absorption spectra of borane **B1**, iodocyclohexane (**1a**), dimethyl maleate (**2b**) and a mixture of **1a** and **B1**. All spectra were recorded 0.2 M in CH_3CN (solvent cutoff: 190 nm) in quartz cuvettes (optical path: 1 cm) with a bandwidth of 5 nm and a data pitch of 1 nm. Scan rate: medium.

In our experiments we could not find any evidence for the formation of an EDA complex able to justify any reactivity under visible light irradiation (456 nm, cfr Table S6, entry 2).

Next, we recorded absorption spectra of reaction components to see if **PC1** could form and EDA complex with **B1**.

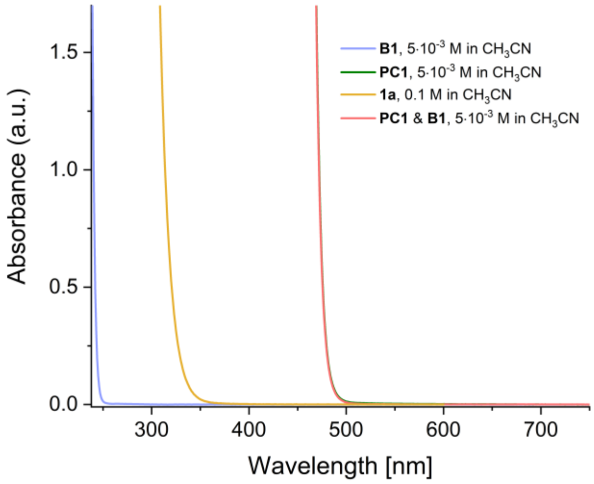

Figure S7: Absorption spectra of borane **B1**, iodocyclohexane (**1a**), mesityl acridinium perchlorate (**PC1**) and a mixture of **PC1** and **B1**. All spectra were recorded in quartz cuvettes (optical path: 1 cm) with a bandwidth of 5 nm and a data pitch of 1 nm. Scan rate: medium.

Besides proving that **PC1** is the only light absorbing species when irradiating at 456 nm, these results prove that an EDA complex between electron-deficient **PC1** and electron-rich **B1** is not formed (**Figure S7**).

Next, we recorded an absorption spectrum of an Ar-bubbled (10 min) solution of **PC1** ($5\cdot10^{-3}$ M in CH_3CN) before and after irradiation with a 40 W Kessil lamp PR160L (456 nm, full intensity, 5 cm away from cuvette) to check the photostability of the photocatalyst alone.

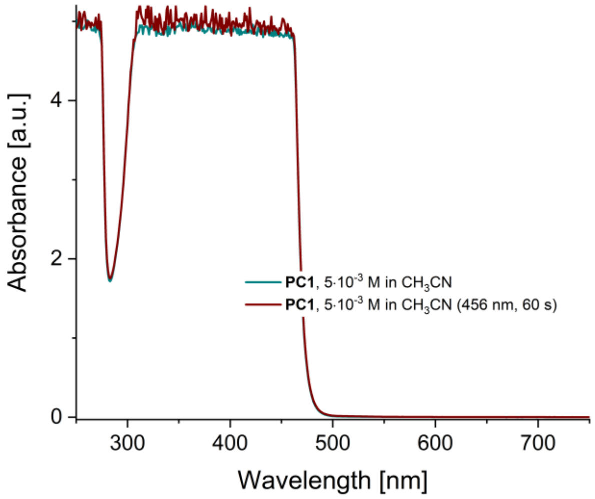

Figure S8: Absorption spectra of an Ar-bubbled solution of **PC1** ($5\cdot10^{-3}$ M in CH_3CN) before and after irradiation (60 s) with a Kessil lamp PR160L (full intensity, 5 cm away from cuvette).

No appreciable changes were observed after 1 minute of continuous irradiation, which shows that **PC1** is stable under operating conditions when **B1** is not in solution (**Figure S8**).

Next we performed the same experiment, but in the presence of **B1**; however, in order to better appreciate the features of the spectral bands, we recorded the spectrum under diluted conditions (**PC1** and **B1**, both 10^{-5} M in CH_3CN). In detail, an Ar-bubbled (10 min) solution of **B1** and **PC1** was placed in a quartz cuvette and irradiated with a 40 W Kessil lamp PR160L (456 nm, full intensity, 5 cm away from cuvette). Absorption spectra were recorded at regular time intervals (**Figure S9a**).

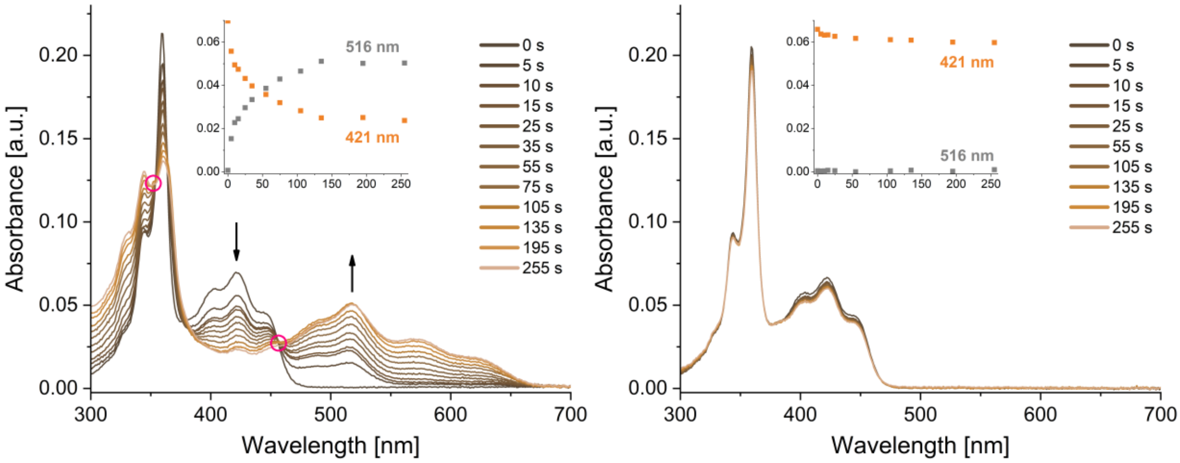

Figure S9: a) Absorption spectra of an Ar-bubbled solution of **B1** and **PC1** (both 10^{-5} M in CH_3CN) irradiated with a 40 W Kessil lamp PR160L (full intensity, 5 cm away from cuvette) taken at regular time intervals; b) Absorption spectra of an Ar-bubbled solution of **PC1, 1a** and **2b** (all 10^{-5} M in CH_3CN) irradiated in the same conditions.

From data shown in **Figure S9a**, it is evident that **PC1** is converted to another species absorbing above 450 nm when irradiated in the presence of **B1**, which does not occur in the absence of irradiation (cfr. **Figure S8**). The conversion is direct (no intermediacy of other species) as shown by the presence of isosbestic points. Based on results obtained in EPR measurements and comparison with the literature, we propose this latter species to be the acridine radical generated upon single-electron reduction by **B1**.[24]

In stark contrast, when a solution containing **PC1**, **1a** and **2b** (all 10^{-5} M in CH_3CN), without **B1**, was irradiated under otherwise identical conditions, the red-light-absorbing species could not be observed (**Figure S9b**).

6.3 Quantum yield measurements

In a similar manner, we also determined the quantum yield for the process under visible-light irradiation.

In a dark room equipped with a red light, a 0.15 M solution of ferrioxalate was prepared by dissolving 736 mg of $K_3[Fe(C_2O_4)_3]*3H_2O$ in 10 mL of 0.05 M H_2SO_4. A buffered solution of phenanthroline was prepared by dissolving 50 mg of phenanthroline and 11.25 g of NaOAc in 50 mL of 0.5 M H_2SO_4.

Four 7-mL vials were charged with 1 mL of the ferrioxalate solution and irradiated with the setup shown in **Figure S1** for the indicated time (see Table S10). 20 μL of the irradiated solutions were added to 2 mL of the phenanthroline solution (1:101 dilution); the resulting mixture was left equilibrating in the dark for 1 h. Next, the obtained solutions were further

diluted by taking 50 µL and adding 3 mL of distilled water (1:61 dilution). The final solution was analyzed via UV-Vis spectroscopy. The experiment was repeated twice by two different operators (operator 1 and operator 2), with two different ferrioxalate batches and different irradiation times.

Table S10: Results obtained for the ferrioxalate actinometry.

OPERATOR 1				OPERATOR 2			
Entry	t_{irr} (s)	$A_{510\ nm}$	mol Fe^{II}	Entry	t_{irr} (s)	$A_{510\ nm}$	mol Fe^{II}
Blank	0	0	0	Blank	0	0	0
1	5	0.013	$7.2\cdot10^{-6}$	1	3	0.008	$4.5\cdot10^{-6}$
2	10	0.023	$1.3\cdot10^{-5}$	2	6	0.016	$8.7\cdot10^{-6}$
3	15	0.032	$1.8\cdot10^{-5}$	3	9	0.017	$9.4\cdot10^{-6}$
4	20	0.043	$2.4\cdot10^{-5}$	4	12	0.024	$1.3\cdot10^{-5}$

Given that

$$photon\ flux\ (F) = \frac{mol_{Fe^{2+}}}{t \times \Phi \times f}$$

from which

$$mol_{Fe^{2+}} = (F \times \Phi \times f) \times t$$

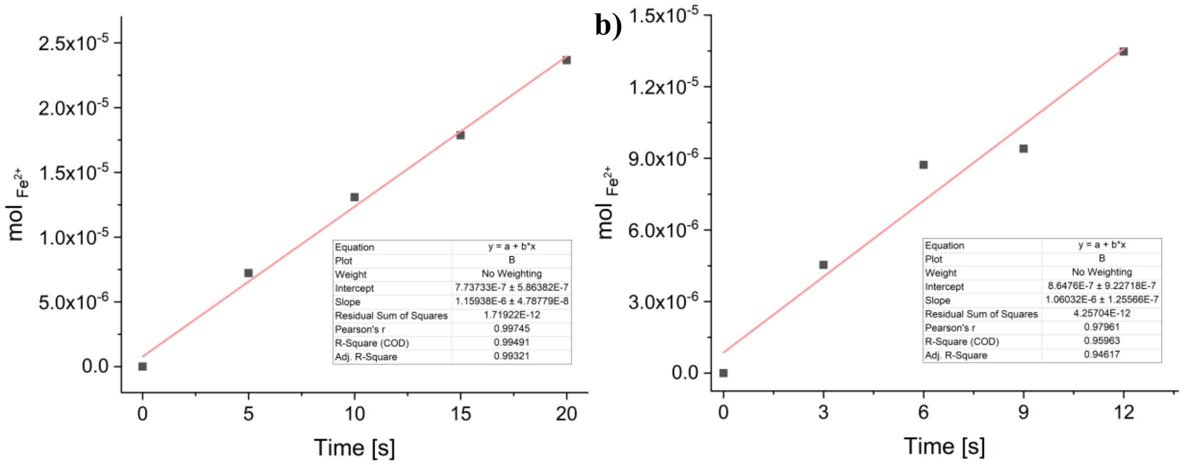

Figure S10: Plot of the moles of Fe^{2+} vs time (s) shows a linear correlation: a) results obtained by operator 1; b) results obtained by operator 2.

By dividing the slopes of the graphs shown in **Figure S10** by the quantum yield and fraction of light absorbed by ferrioxalate at the considered wavelength (data were available for $\lambda = 464$ nm: $\Phi = 0.98$ and $f = 0.93$),[25] an average photon flux of $1.41\cdot10^{-6}$ E s^{-1} can be estimated.

Considering that the yield for compound **3** after 10 min (600 s) in the same setup is 19%, the quantum yield for the reaction is calculated to be ~2%.

As it is known that the apparent quantum yield can depend on the light intensity,[26] we also measured the quantum yield in a different reactor equipped with much less powerful blue LEDs.
Reactor design: The reactor consists of a 3D-printed (PLA) reactor (height: 10 cm, inner diameter: 12.5 cm) that has been internally coated with LED strips (456 nm LEDs, 18 W, 24 V; purchased from 123-LED). The reactor was capped with a 3D-printed (PLA) lid with 8 holes serving as vials holder. Distance of the vial from LED: 2 cm.
The same procedure described above was adopted for determining the photon flux.

Table S11: Results obtained for the ferrioxalate actinometry.

Entry	t_{irr} (s)	$A_{510\ nm}$	mol Fe^{II}
Blank	0	0	0
1	30	0.005	$2.8 \cdot 10^{-6}$
2	60	0.009	$5.1 \cdot 10^{-6}$
3	90	0.012	$6.5 \cdot 10^{-6}$
4	120	0.019	$1.0 \cdot 10^{-5}$

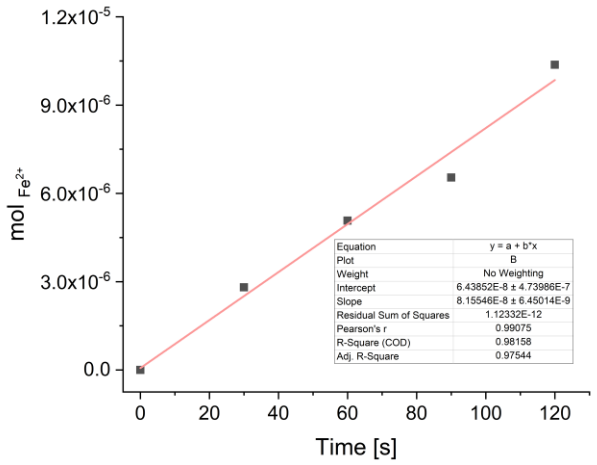

Figure S11: Plot of the moles of Fe^{2+} vs time (s) shows a linear correlation. Results obtained with low intensity blue LEDs.

By dividing the slope of the graphs shown in **Figure S11** by the quantum yield and fraction of light absorbed by ferrioxalate at the considered wavelength (data were available for λ = 464 nm: Φ = 0.98 and f = 0.93)[25] a photon flux of $9.25 \cdot 10^{-8}$ E s^{-1} can be estimated.
Considering that the yield for compound **3** after 160 min (9600 s) in the same setup is 16%, the quantum yield for the reaction is calculated to be 2%.

All in all, a QY of 2% has been measured by two different operators, with different batches of the actinometer, with different light sources and reactors. Although such a modest value is in accordance with a process being supported by either short-lived radical chain propagations or an inefficient initiation process,[27] we believe that the premature decomposition of the photocatalyst is responsible for this result.

6.4 Deuterium labelling experiments

Reactions in this section were performed as detailed in GP3 (*vide infra*). In particular, a solution of **2a** (0.5 mmol), **1a** (2.0 equiv.), **B1** or **B1-*d*$_3$** (1.2 equiv.) and **PC1** (5 mol%) in CH_3CN/H_2O 9:1 or CD_3CN/D_2O 9:1 (0.1 M) was prepared in a 7 mL vial equipped with a screw cap and a stirring bar. The solution was bubbled with N_2 (5 min) and irradiated with a 40 W Kessil lamp (λ = 456 nm, full intensity) for 3 h in the UFO reactor (see **Figure S1**). The reaction mixture was then collected, solvent was removed under reduced pressure and the crude was purified via column chromatography on silica gel (pentane/ethyl acetate 95:5) to afford product **3**, **3-*d*$_1$** or a mixture of them.

Characterization data for **3**:

^{1}H NMR (300 MHz, $CDCl_3$) δ 4.15 – 4.0 (m, 4H), 2.77 – 2.60 (m, 2H), 2.49 – 2.35 (m, 1H), 1.79 – 1.52 (m, 10H), 1.45 – 1.29 (m, 4H), 1.28 – 0.98 (m, 5H), 0.92 (td, J_1 = 7 Hz, J_2 = 3 Hz, 6H). ^{13}C NMR (75 MHz, $CDCl_3$) δ 174.7, 172.8, 64.6, 64.4, 47.2, 40.2, 33.6, 30.8, 30.8, 30.7, 30.2, 26.5 (2C), 26.3, 19.3, 19.2, 13.8 (2C).

Characterization data for **3-*d*$_1$** *(from experiment in section 6.4.3)*:

^{1}H NMR (300 MHz, $CDCl_3$) δ 4.11 – 3.91 (m, 4H), 2.72 – 2.55 (m, 1.4H), 2.44 – 2.29 (m, 0.7H), 1.74 – 1.45 (m, 10H), 1.40 – 1.22 (m, 4H), 1.22 – 0.91 (m, 5H), 0.86 (td, J_1 = 7 Hz, J_1 = 3 Hz, 6H). ^{13}C NMR (101 MHz, $CDCl_3$) δ 174.6, 172.7, 64.5, 64.4, 47.2 (m), 40.1, 33.3 (t, J = 20 Hz), 30.8, 30.7, 30.7, 30.2, 26.4 (2C), 26.3, 19.3, 19.2, 13.8 (2C).

6.4.1 Deuterated solvents

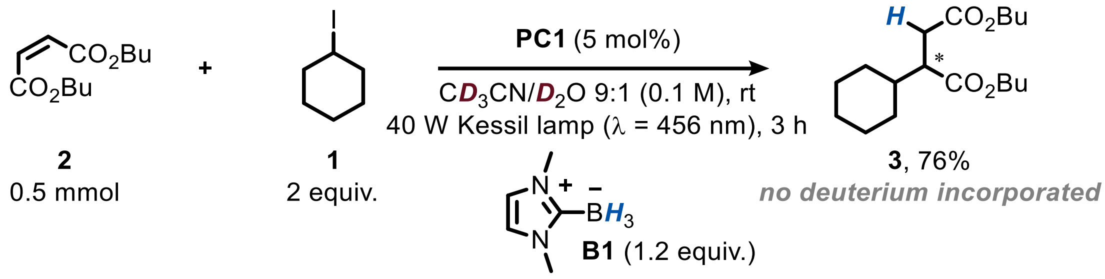

Reaction performed as described above: **B1** (1.2 equiv.) and CD_3CN/D_2O 9:1 (0.1 M) were used. Product **3** was isolated in 76% yield; no deuterium incorporation was observed.

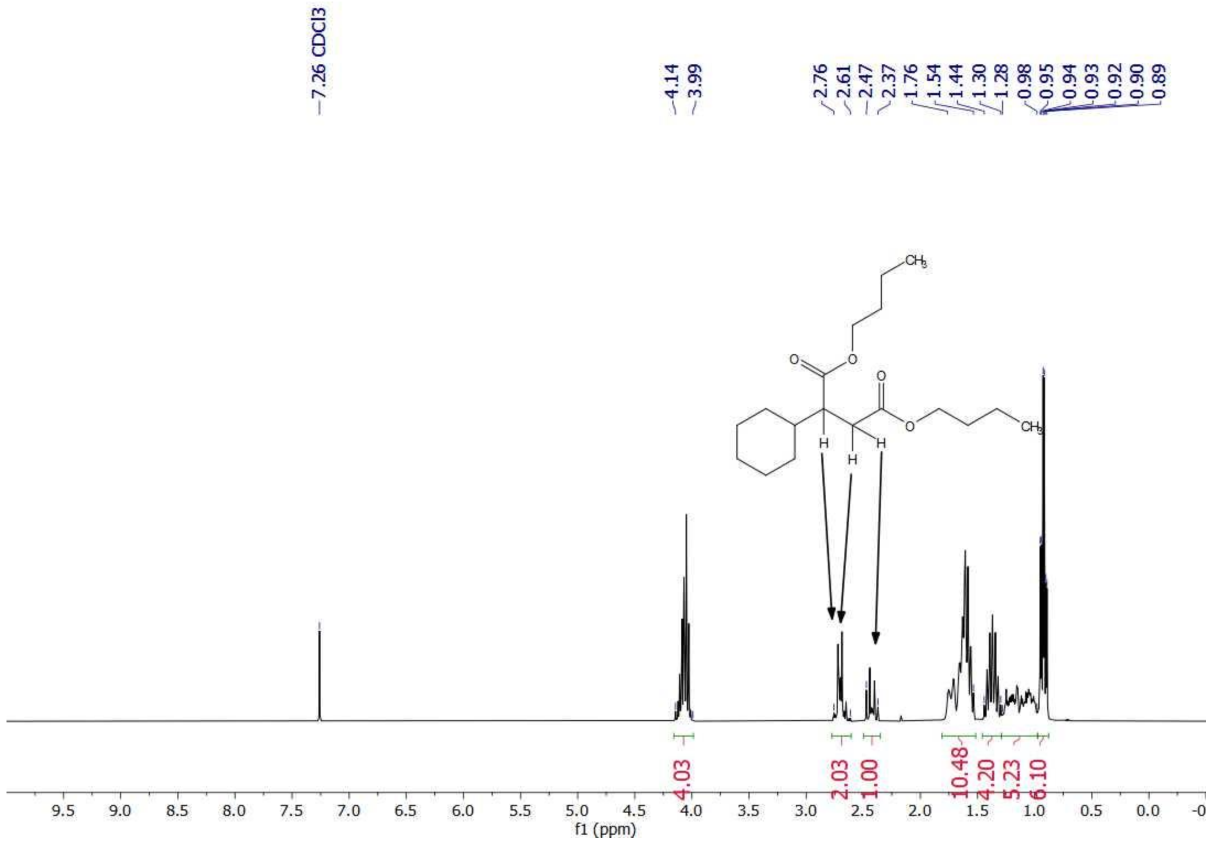
7.26 CDCl3
4.14
3.99
2.76
2.61
2.47
2.37
1.76
1.54
1.44
1.30
1.28
0.98
0.95
0.94
0.93
0.92
0.90
0.89
CH3
O
O
O
O
H
H
H
4.03
2.03
1.00
10.48
4.20
5.23
6.10
f1 (ppm)

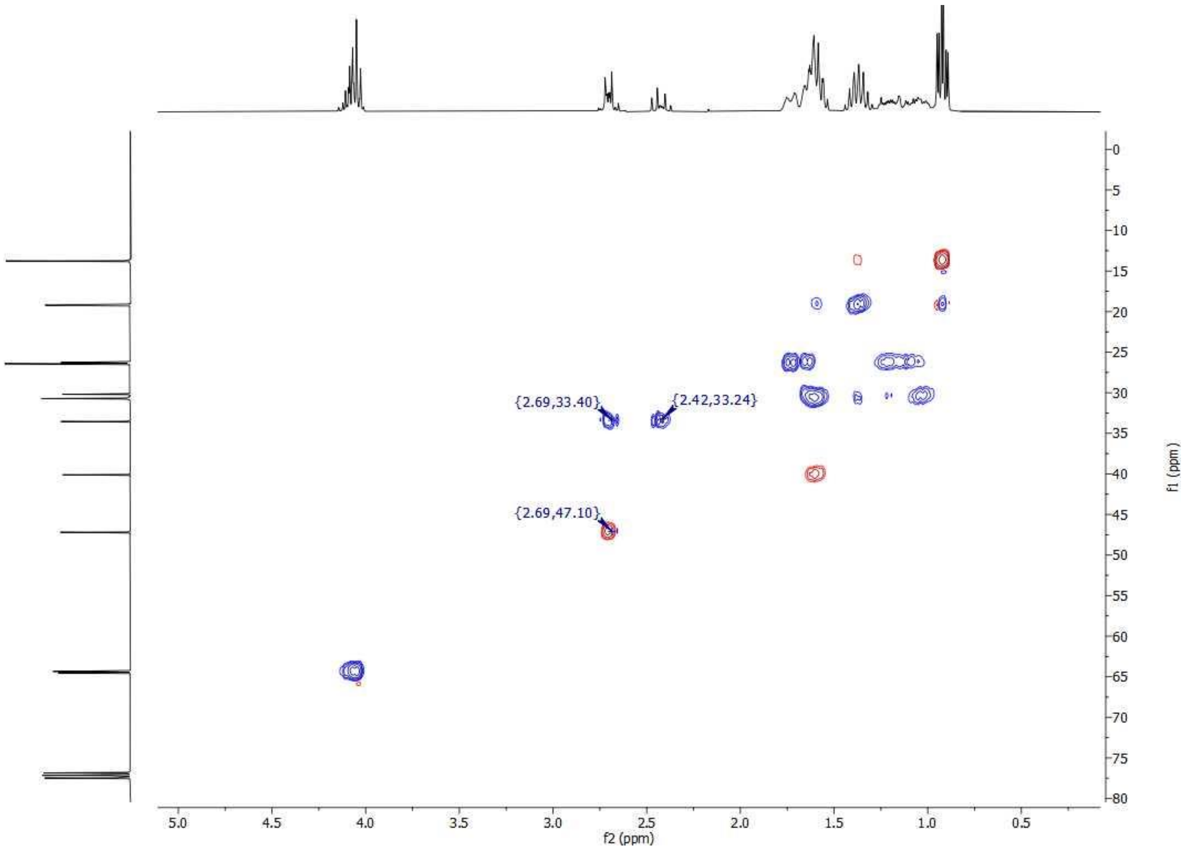
{2.69,33.40}
{2.42,33.24}
{2.69,47.10}
f2 (ppm)
f1 (ppm)

6.4.2 Deuterated ligated borane

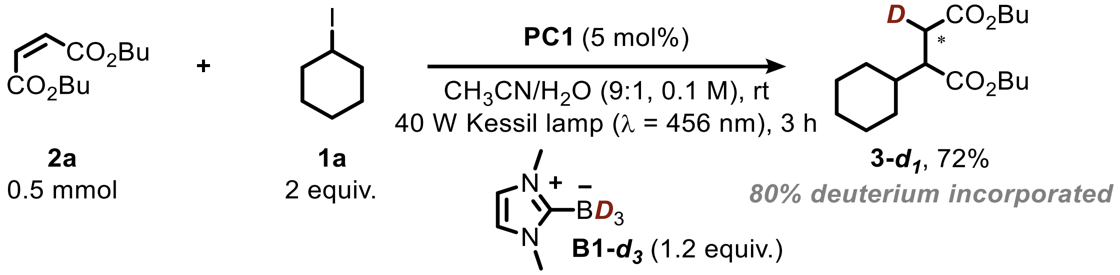

Reaction performed as described above: **B1-*d*₃** (1.2 equiv.) and CH_3CN/H_2O 9:1 (0.1 M) were used. A mixture of product **3** (14%) and **3-*d*₁** (58%, d.r. 1.8:1) was isolated (72% yield overall); 80% of deuterium incorporation was observed on the α-to-ester methylene.

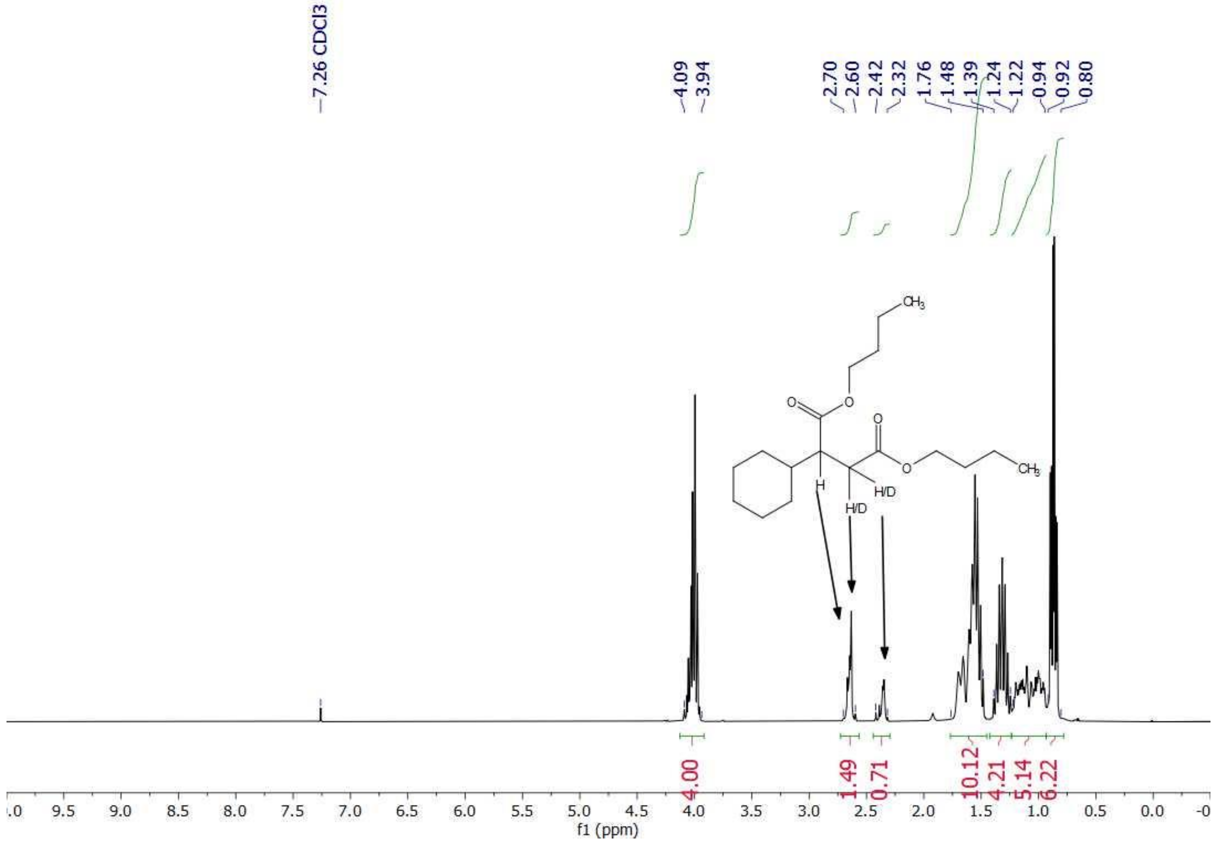

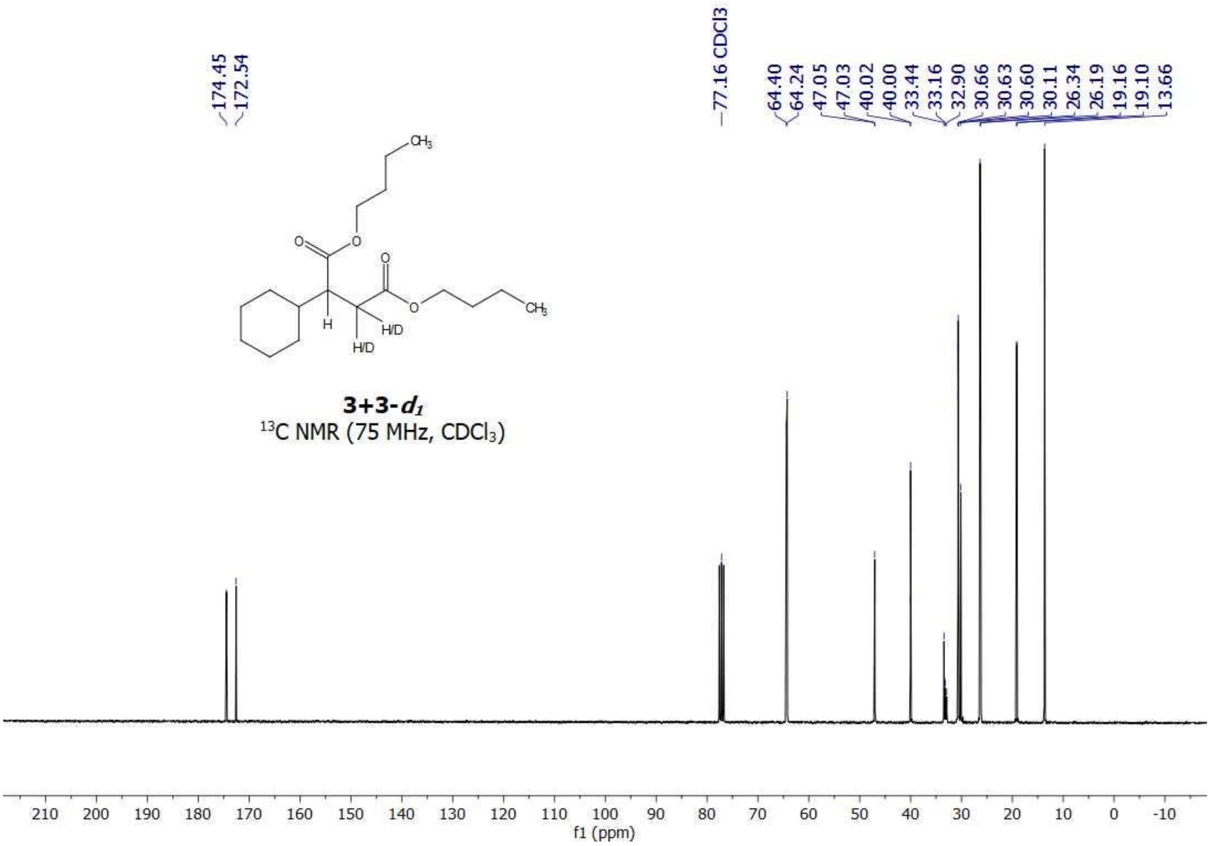

6.4.3 Deuterated ligated borane and solvents

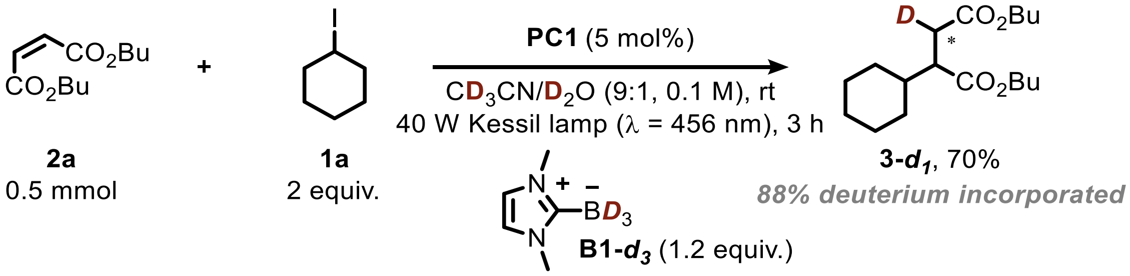

Reaction performed as described above: **B1-*d₃*** (1.2 equiv.) and CD_3CN/D_2O 9:1 (0.1 M) were used. A mixture of product **3** (8%) and **3-*d₁*** (62%, d.r. 1.8:1) was isolated (70% yield overall); 88% of deuterium incorporation was observed on the α-to-ester methylene.

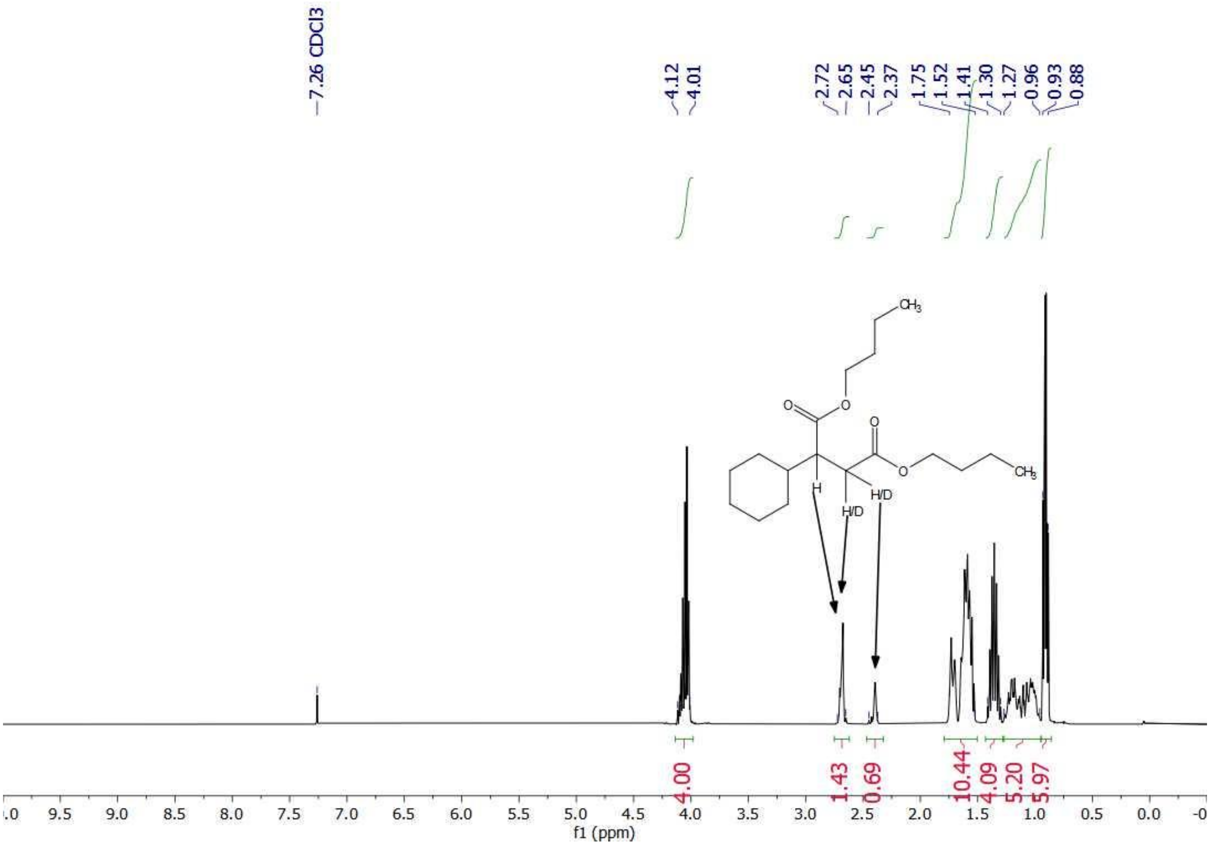

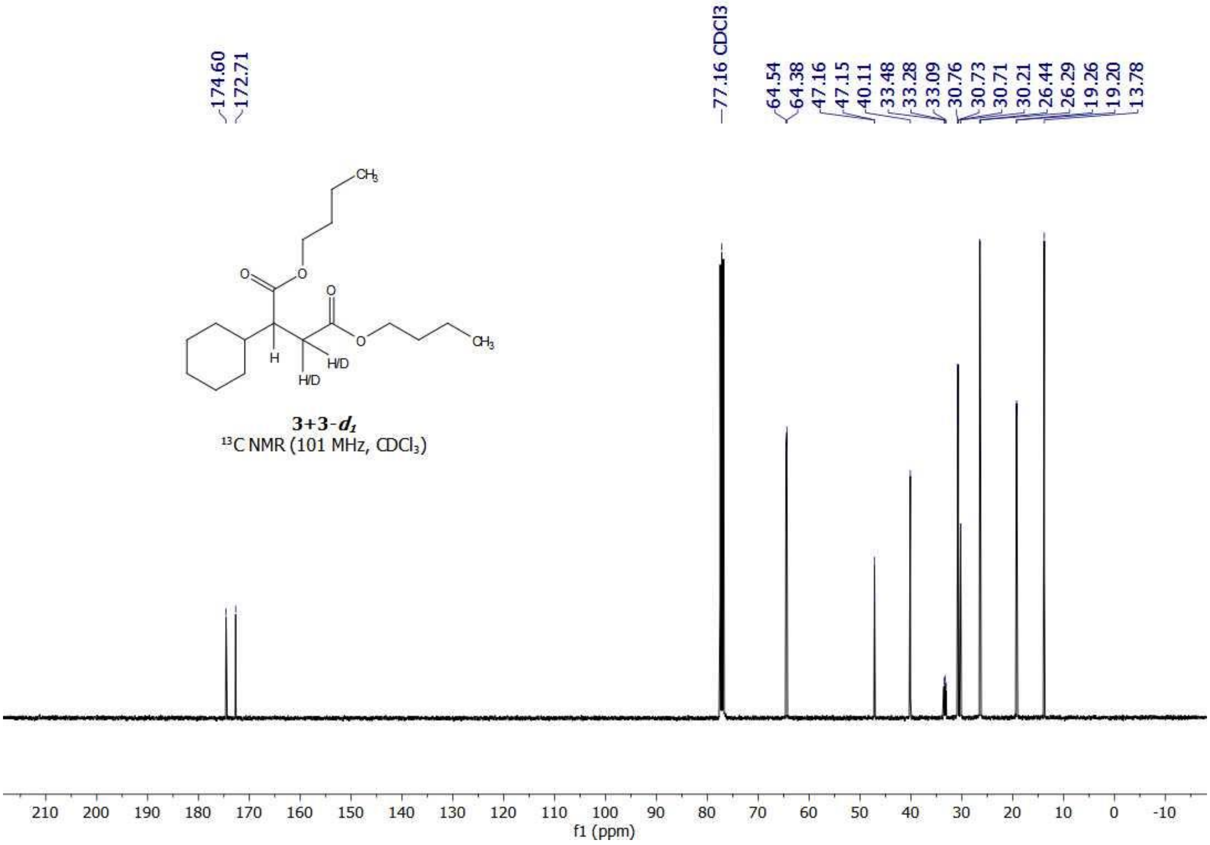

The experiments above support the fact that a radical chain mechanism is the major pathway for our reaction.

6.5 Evaluation of kinetic isotopic effect (KIE)

We evaluated the KIE via the parallel reaction method (**Figure S12**). In detail, we measured the rates of two independent reactions under optimized conditions (Table S5, entry 3), one containing **B1** and one containing **B1-*d*₃** (in both cases 1.2 equiv.). After 3, 6, 9 minutes of irradiation, 1 mL of a standard solution of biphenyl (0.1 M in EtOAc) was added, 70 μL of the resulting solution were withdrawn and further diluted with 1 mL of EtOAc. The resulting mixture was analyzed via GC-MS and yields were calculated by means of calibration curves built with authentic samples.

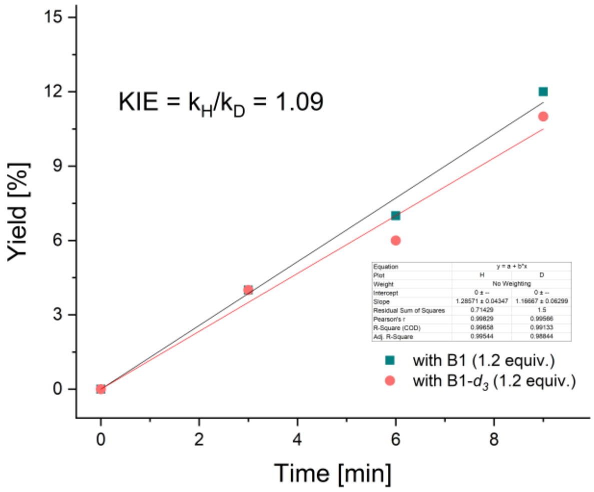

Figure S12: Evaluation of the KIE with the parallel reactions method by using **B1** and **B1-*d*₃**.

A KIE of 1.09 was calculated, thus we conclude that a HAT step might not be involved in the rate-determining step of the reaction.

KIE was also evaluated according to the competition reaction method, where the reaction was run under optimized conditions in the presence of an equimolar amount of **B1** and **B1-*d*₃** (5 equiv. each). The reaction was performed according to GP3 (*vide infra*) and a 3.5:1 mixture of product **3** (65%) and **3-*d*₁** (18%, d.r. 2:1) was isolated (83% yield overall). KIE was also evaluated in the presence of **B1** and **B1-*d*₃** 1.2 equiv. each and a 3:1 mixture of product **3** (60%) and **3-*d*₁** (20%, d.r. 2:1) was isolated (80% yield overall).

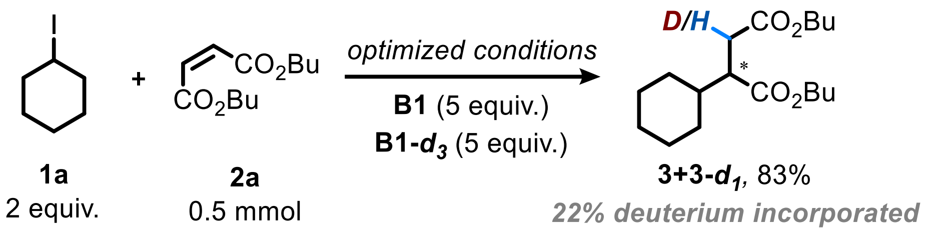

1a
2 equiv.
2a
0.5 mmol
optimized conditions
B1 (5 equiv.)
B1-d3 (5 equiv.)
D/H
CO2Bu
CO2Bu
3+3-d1, 83%
22% deuterium incorporated

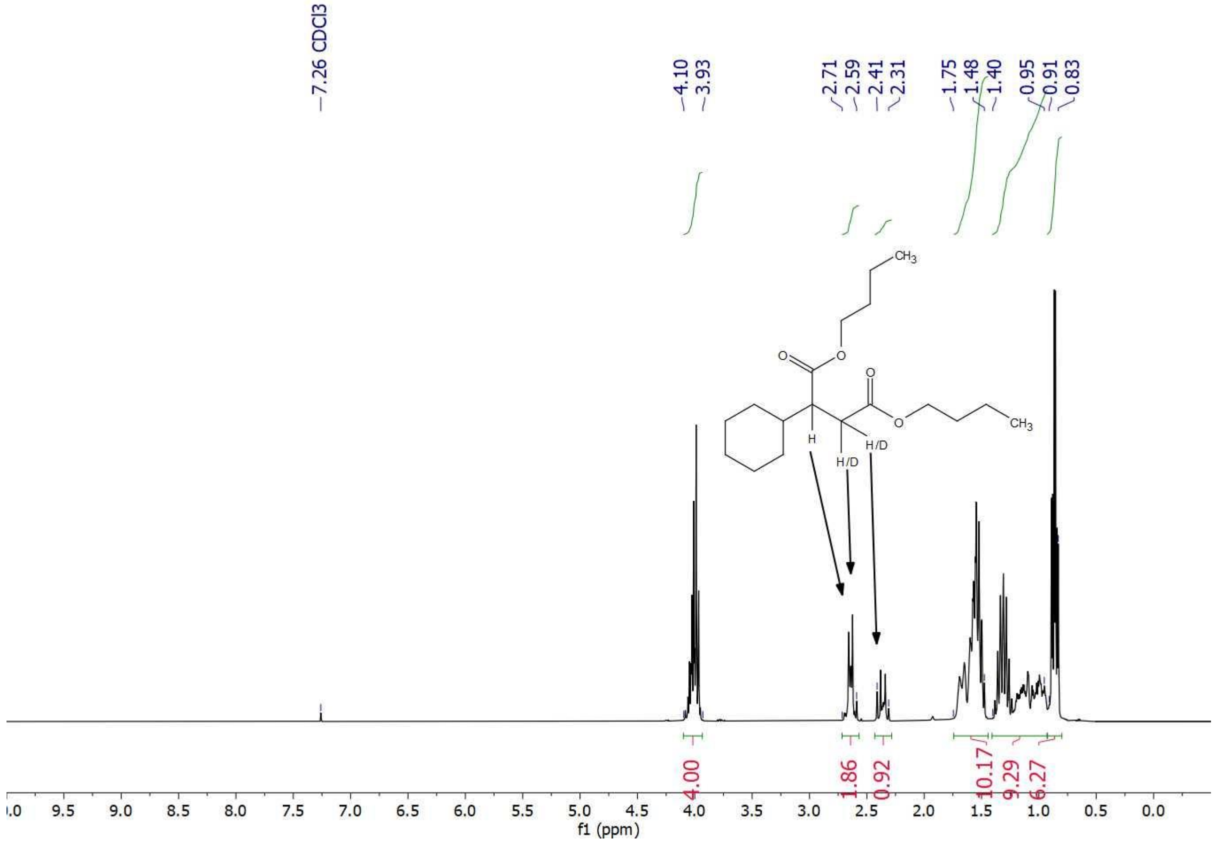

7.26 CDCl3
4.10
3.93
2.71
2.59
2.41
2.31
1.75
1.48
1.40
0.95
0.91
0.83
H
H/D
H/D
4.00
1.86
0.92
10.17
9.29
6.27
f1 (ppm)

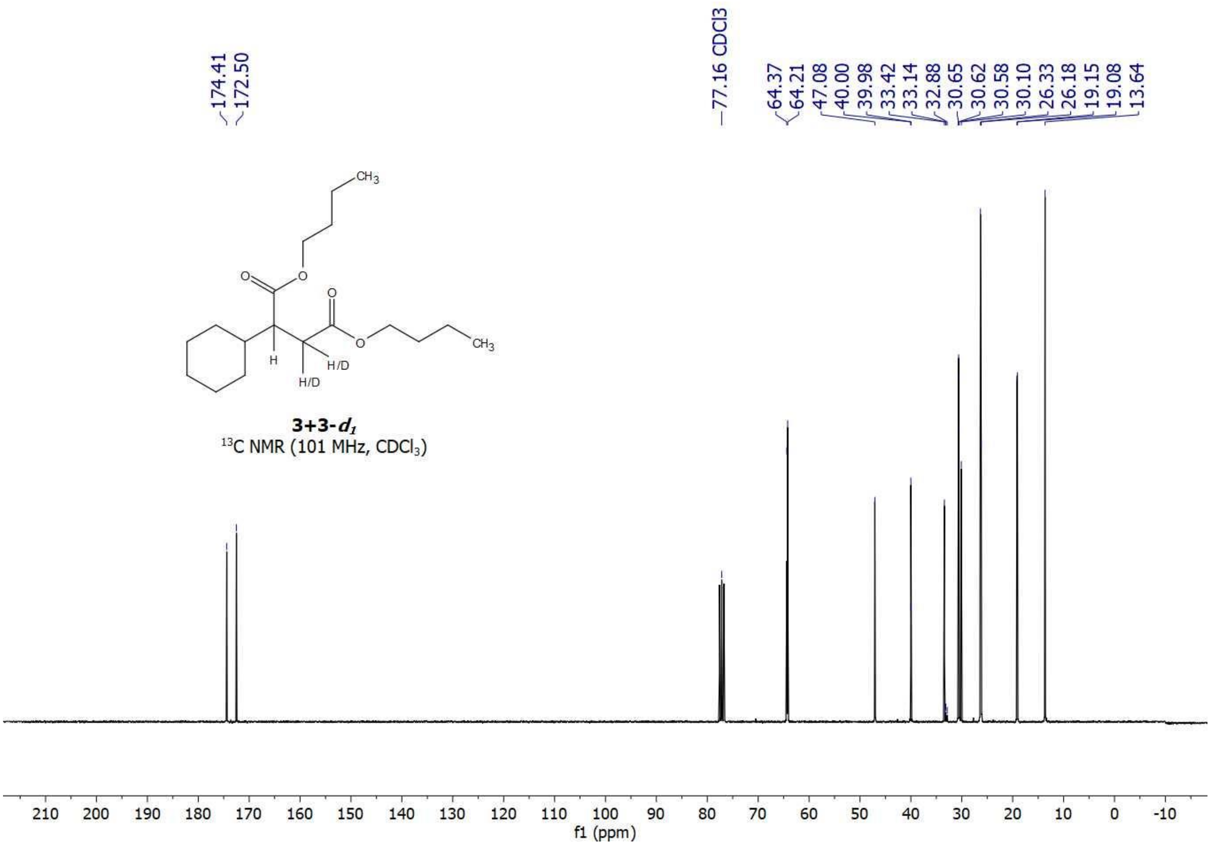

6.6 Radical quenching experiment with TEMPO

We performed a chemical quenching experiment with TEMPO, a well-known radical scavenger. In particular, we performed the model reaction in optimized conditions (Table S5, entry 3) in the presence of TEMPO (2.5 equiv.). The solution was bubbled with N_2 (1 min) and irradiated with a 40 W Kessil lamp ($\lambda = 456$ nm, full intensity) for 3 h in the UFO reactor (see Figure S1). The crude was analyzed via GC-MS and we found that the desired reactivity was completely shut down. Instead, the cyclohexyl-TEMPO adduct was detected.

Method: T_i = 50 °C, rate = 25 °C/min, T_f = 300 °C; hold time: 10 min. Column: SH-Rtx-5 Amine (thickness: 0.25 μm, diameter 0.25 mm, length: 30 m). Column flow: 3.3 mL/min

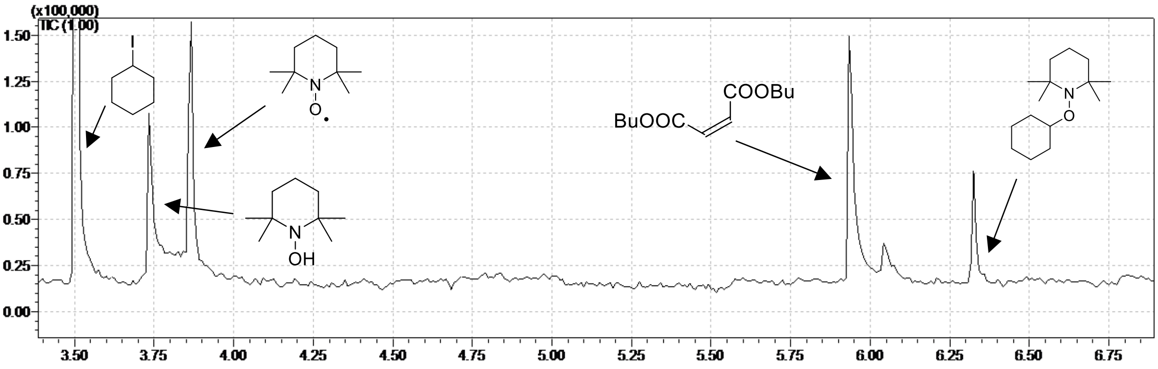

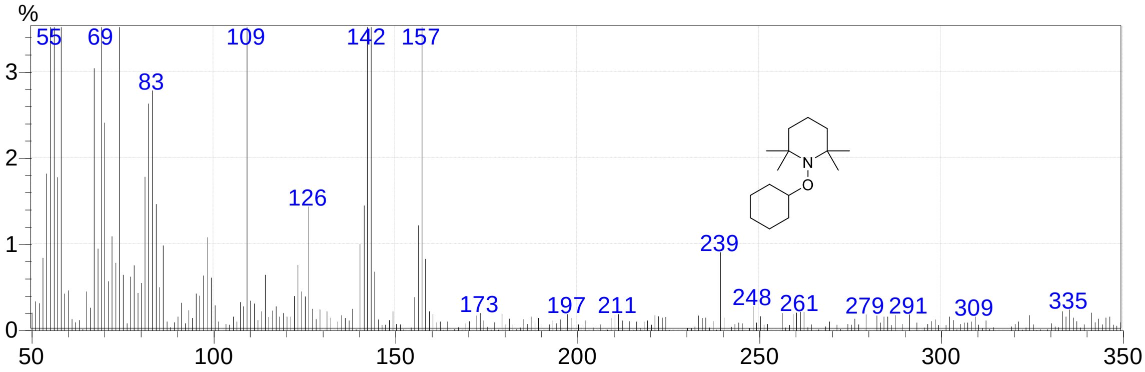

6.7 Competition experiments

We also performed a competition experiment where a mixture of a primary (iodobutane), secondary (**1a**) and tertiary (**1m**) was reacted in optimized conditions for both manifolds and the yields of the corresponding products were calculated by GC-MS by means of calibration curves built with authentic samples. Results are reported in Figure S13:

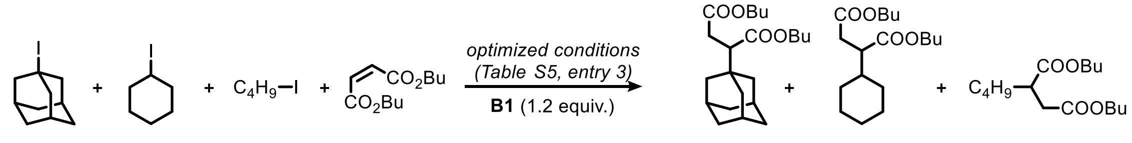

Figure S13: Competition reactions to prove the intermediacy of the same ligated boryl radical.

6.8 Other experiments

Identification of by-products

According to our proposed mechanistic scenario, NHC-BH_2I should be formed upon the XAT event. In fact, as reported by Curran and co-workers,[15] NHC-BH_2I is quite sensitive to aqueous environments and readily decomposes to give the corresponding imidazolium and boric acid, both soluble in water. These species were detected in the reaction crude of our experiments (see, e.g., Figure S14-S16). Therefore, in order to prove that NHC-BH_2I is actually formed during the reaction, we decided to perform our model reaction in deuterated benzene (C_6D_6) and analyze the mixture right after irradiation via ^{11}B-NMR. The characteristic triplet at -31.5 ppm was observed, thus confirming Curran's observation and our hypothesis (see Figure S16).

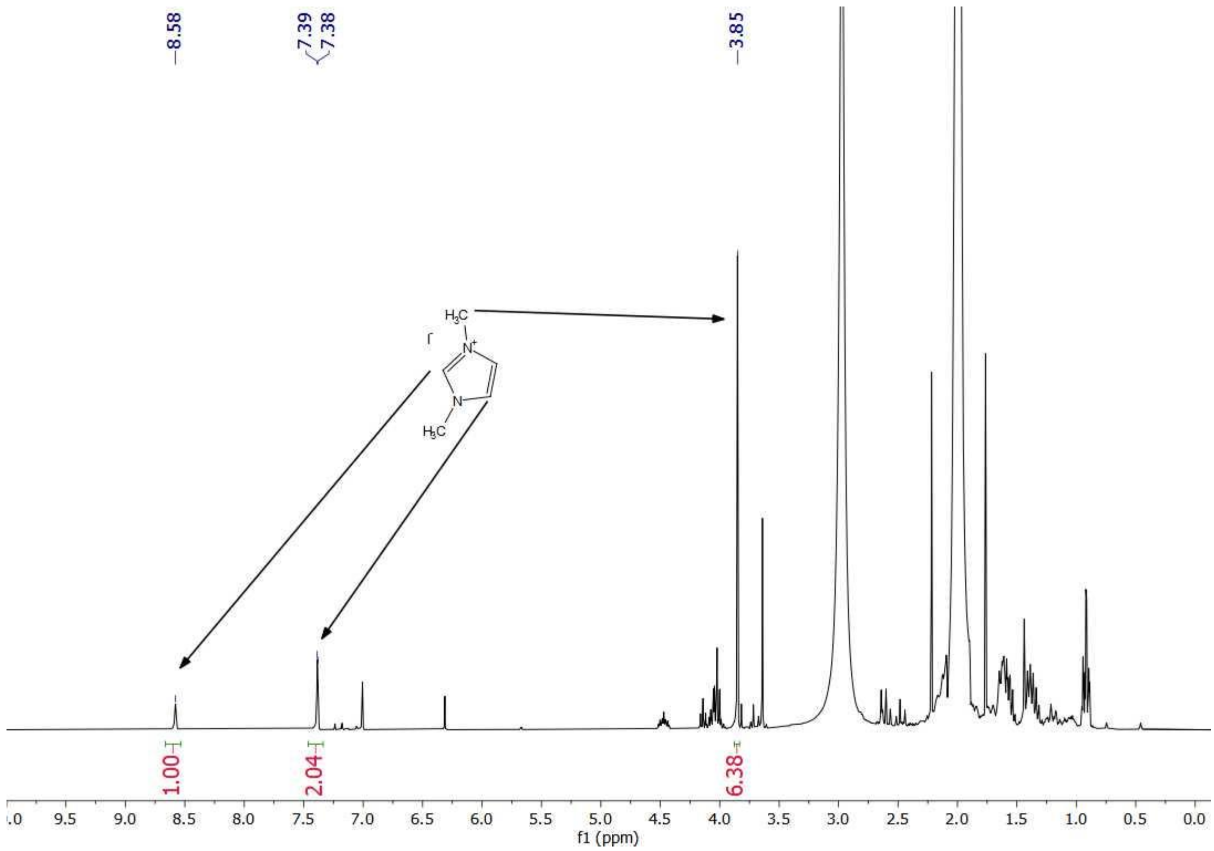

Figure S14: ^{1}H NMR spectrum of the reaction crude. Signals of *N,N*-dimethylimidazolium iodide highlighted (solvent: CD_3CN).

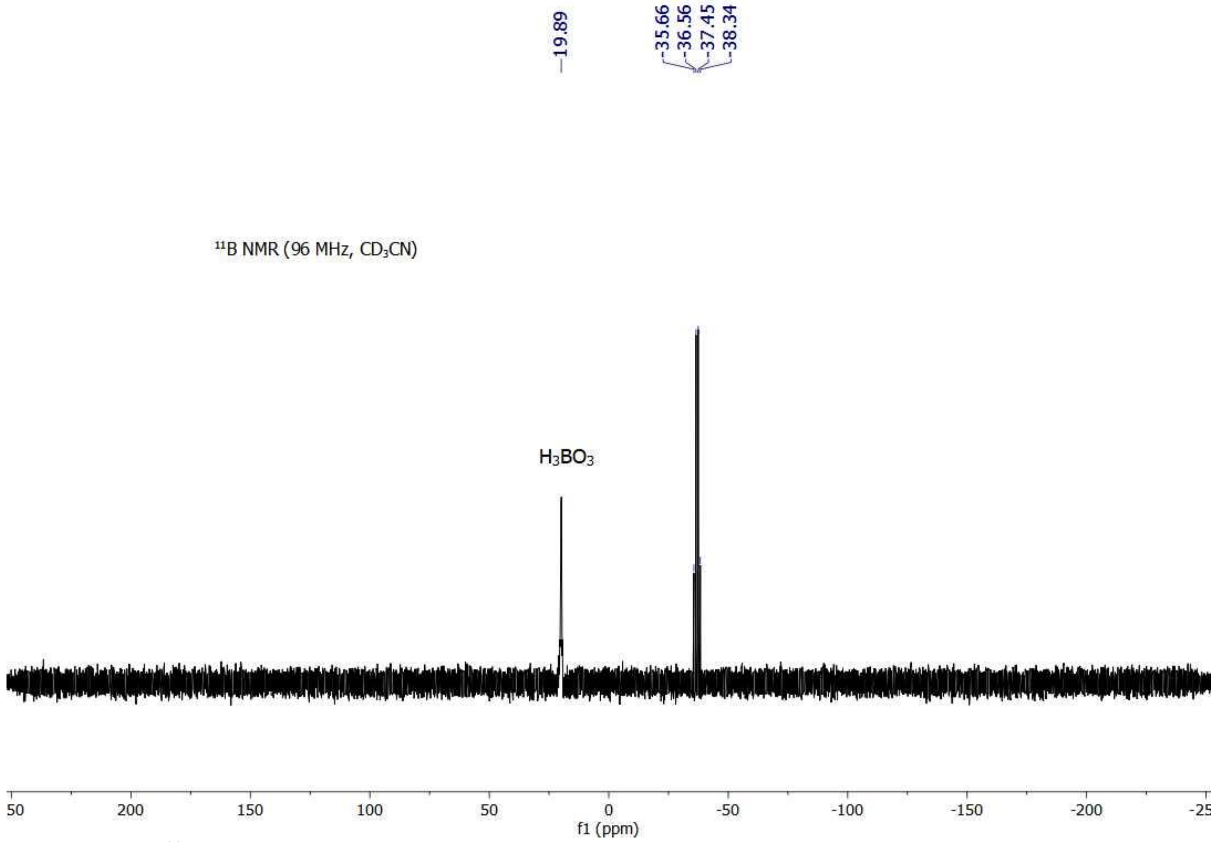

Figure S15: ^{11}B NMR spectrum of the reaction crude. Signal of boric acid highlighted (solvent: CD_3CN).[28]

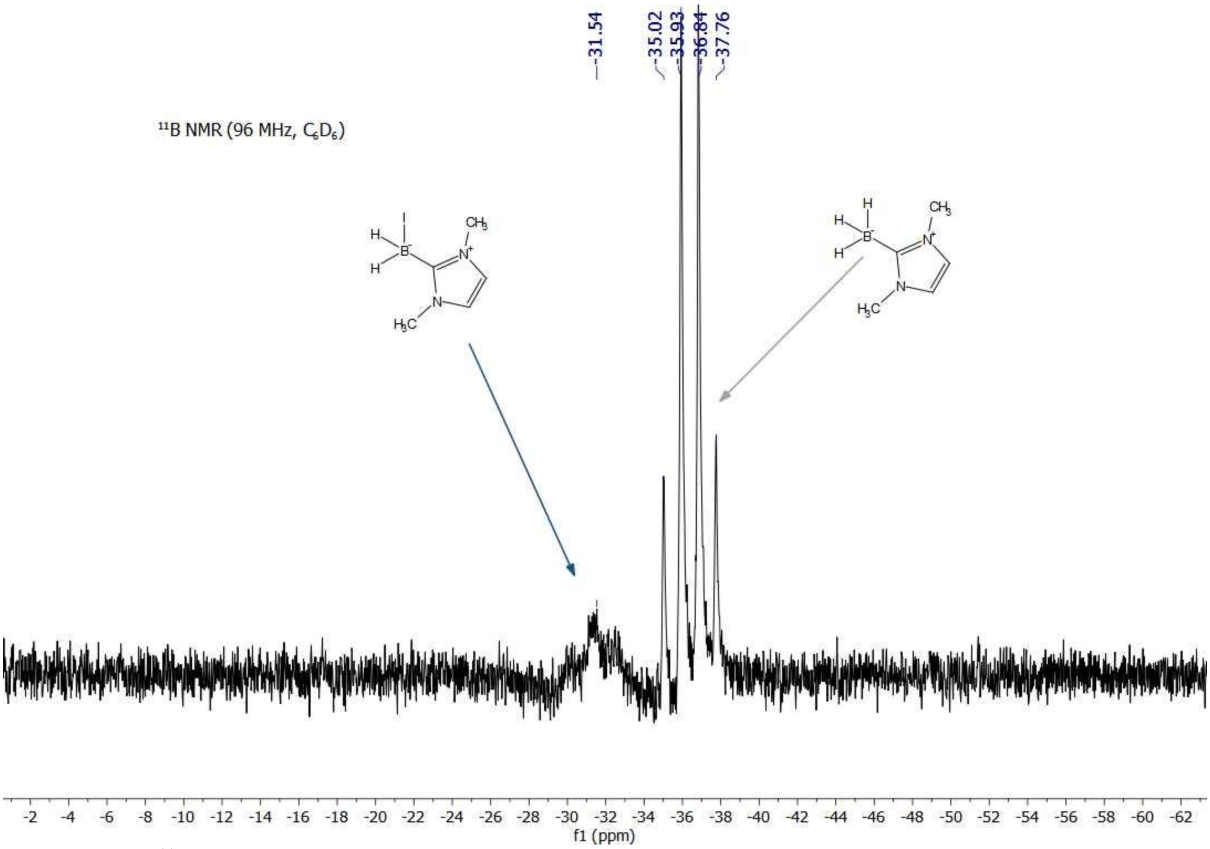

Figure S16: ^{11}B NMR spectrum of the reaction crude. Signals of NHC-BH_2I highlighted (solvent: C_6D_6).[22]

During the reaction we also observed the formation of a gas, which we identified as hydrogen gas by adopting a H_2-leak controller (Model LC10 from Leak Control Benelux). We propose that the formation of H_2 is due to the decomposition of NHC-BH_2I. Moreover, given the fragility of NHC-BH_2I, we propose its participation to the radical chain mechanism to be unlikely.

Fate of the photocatalyst

Intrigued by the results obtained in the above sections, we decided to get more insights in the doom of the photocatalyst. In particular, after the model reaction was performed as in our optimized conditions (Table S9, entry 3), an aliquot was withdrawn and diluted with neat CH_3CN to have [**PC1**] = 10^{-5} M (1:500 dilution). An absorption spectrum of the so-obtained solution was recorded (**Figure S17**).

Although macroscopically speaking the solution was still yellow after irradiation, the UV-Vis spectrum showed clear signs of the decomposition of the photocatalyst.

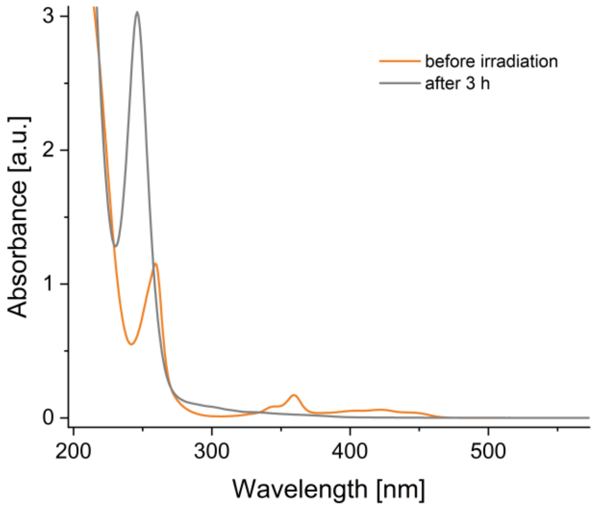

Figure S17: Absorption spectra of the reaction crude diluted 500 times before and after irradiation. Spectra recorded in quartz cuvettes (optical path: 1 cm) with a bandwidth of 5 nm and a data pitch of 1 nm. Scan rate: medium.

In view of the above, we propose that our reaction is a photoinitiated transformation.

6.9 Comparison with other XAT agents

In analogy to our methodology, α-aminoalkyl radicals are typically generated under photoredox conditions upon the reductive quenching of the excited state of a photocatalyst (and subsequent deprotonation). Although this activation manifold has been applied to a plethora of alkyl and aryl halides, a major challenge can still be identified: the use of strongly alkylating agents, e.g. CH_3I, as starting materials. In fact, the alkylating agent would alkylate the amine giving the corresponding ammonium ion, thus hampering the required photoredox chemistry; therefore, we became interested in knowing if CH_3I could survive our photoredox conditions. Thus, we performed the reaction as indicated in Table S5, entry 3 (C_6D_6 as the solvent, as the presence of water caused the hydrolysis of methyl iodide) and detected the expected product in 46% ^{1}H-NMR yield. In stark contrast, when a degassed CH_3CN/H_2O 10:1 solution (0.1 M) of CH_3I (10 equiv.), **2a** (0.1 mmol), triethylamine (2 equiv.), 4CzIPN (5 mol%) was irradiated with a 40 W Kessil lamp (λ = 456 nm, full power),[29] no product was detected by ^{1}H-NMR.

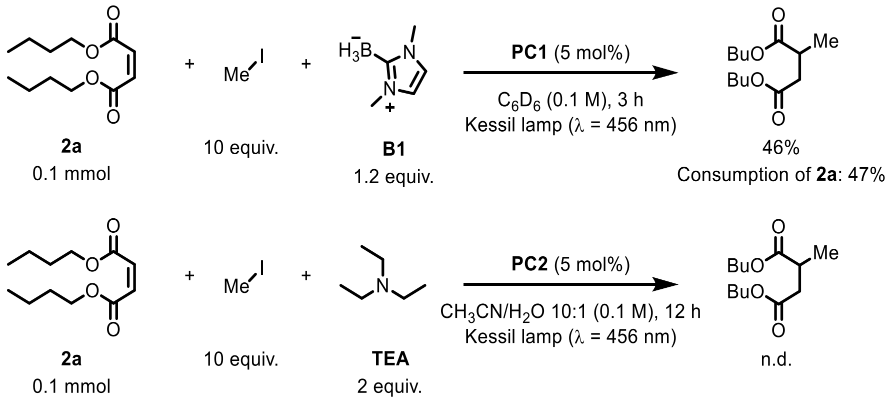

7. General procedures (GPs) for preparative experiments

7.2 GP3: batch conditions, blue light (456 nm) for 3° and 2° alkyl iodides

A CH_3CN/H_2O 9:1 (0.1 M) solution containing the electron-poor olefin **2** (0.5 mmol), organic halide **1** (2.0 equiv.), **B1** (1.2 equiv.) and **PC1** (5 mol%) was prepared in a 7 mL vial equipped with a screw cap and a stirring bar. The solution was bubbled with N_2 (5 min) and then irradiated by adopting the setup shown in **Figure S1** equipped with a 40 W Kessil lamp (λ = 456 nm, full intensity) for 3 h. The solutions were collected, solvent was removed under reduced pressure and the crude was purified via column chromatography on silica gel to provide the expected product.

7.3 GP4: batch conditions, blue light (456 nm) for 1° alkyl iodides

A CH_3CN/H_2O 9:1 (0.1 M) solution containing the electron-poor olefin **2** (2.0 equiv.), organic halide **1** (0.5 mmol), **B1** (1.2 equiv.) and **PC1** (5 mol%) was prepared in a 7 mL vial equipped with a screw cap and a stirring bar. The solution was bubbled with N_2 (5 min) and then irradiated by adopting the setup shown in **Figure S1** equipped with a 40 W Kessil lamp (λ = 456 nm, full intensity) for 12 h. The solutions were collected, solvent was removed under reduced pressure and the crude was purified via column chromatography on silica gel to provide the expected product.

7.4 GP5: batch conditions, blue light (456 nm) for alkyl bromides

A stock solution containing alkyl bromide **1** (0.5 mmol), olefin **2** (2 equiv.), **B1** (1.0 equiv.), NaI (2 equiv.), **PC2** (2 mol%) in CH_3CN/H_2O (9:1, 0.1 M) were mixed in a 7 mL vial equipped with screw cap with septum and stirring bar under N_2, then irradiated by adopting the setup shown in **Figure S1** equipped with a 40 W Kessil lamp (λ = 456 nm, full intensity) for 12 h. The solutions were collected, solvent was removed under reduced pressure and the crude was purified via column chromatography on silica gel to provide the product.

7.5 GP6: continuous flow conditions, blue light (450 nm)

1 (0.5 mmol), **2** (2 equiv.), **B1** (1.2 equiv.), **PC1** (5 mol%) in DMSO (0.5 M) were mixed in a 7 mL flash and the flask was swirled to achieve homogeneity. The liquid was taken up with a

syringe and mounted on a syringe pump. The syringe was connected to a 3.18 mL reactor (PFA capillary tubing, 0.8 mm inner diameter) at the outlet connected a back pressure regulator (BPR, 2.8 bar) at 50 ºC. The liquid feed was pumped into the flow reactor at 0.106 mL/min (corresponding to 30 minutes residence time). When the syringe was fully empty, again CH_3CN was loaded into a syringe and injected to collect all product at the end of the reactor in a flask. Vapourtec reactor light (λ = 450 nm, 60 W) was used. The solutions were collected, then diluted with H_2O and EtOAc. The layers were separated and the aqueous layer was extracted with EtOAc (×2). The combined organic layers was washed with brine, dried with $MgSO_4$, filter and evaporated. The crude was purified via column chromatography on silica gel to afford the product.

8. Scale-up for the synthesis of compound 3 in continuous-flow

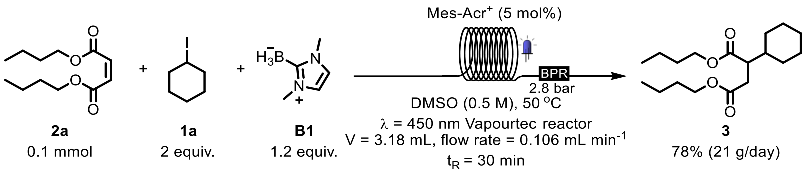

2a (5 mmol, 1.14 mL), **1a** (2 equiv., 1.28 mL), **B1** (1.2 equiv., 660 mg), **PC1** (5 mol%, 103 mg) in DMSO (0.5 M) were mixed in a 25 mL flash and the flask was swirled to achieve homogeneity. The liquid was taken up with a syringe and mounted on a syringe pump. The syringe was connected to a 3.18 mL reactor (PFA capillary tubing, 0.8 mm inner diameter) at the outlet connected a back pressure regulator (BPR, 2.8 bar) at 50 ºC. The liquid feed was pumped into the flow reactor at 0.106 mL/min (corresponding to 30 minutes residence time). When the syringe was fully empty, again CH_3CN was loaded into a syringe and injected to collect all product at the end of the reactor in a flask. Vapourtec reactor light (λ = 450 nm, 60 W) was used. The solutions were collected, then diluted with H_2O and EtOAc. The layers were separated and the aqueous layer was extracted with EtOAc (×2). The combined organic layers was washed with brine, dried with $MgSO_4$, filter and evaporated. The crude was purified via column chromatography on silica gel (Cyclohexane:Ethyl Acetate 90:10) to afford the product as yellow oil **3** (1.22 g, 78%).

9. Characterization data

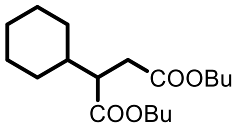

Dibutyl 2-cyclohexylsuccinate (3). Prepared according to GP3. Purified by flash column chromatography on silica gel (Cyclohexane:Ethyl Acetate 90:10) to afford the product as yellow oil (114 mg, 73%). ^{1}H NMR (300 MHz, $CDCl_3$) δ 4.15 – 4.0 (m, 4H), 2.77 – 2.60 (m, 2H), 2.49 – 2.35 (m, 1H), 1.79 – 1.52 (m, 10H), 1.45 – 1.29 (m, 4H), 1.28 – 0.98 (m, 5H), 0.92 (td, J_1 = 7 Hz, J_2 = 3 Hz, 6H). ^{13}C NMR (75 MHz, $CDCl_3$) δ 174.7, 172.8, 64.6, 64.4, 47.2, 40.2, 33.6, 30.8, 30.8, 30.7, 30.2, 26.5 (2C), 26.3, 19.3, 19.2, 13.8 (2C). Spectroscopic data are in accordance with the literature.[30]

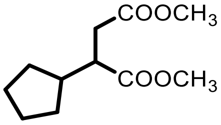

Dimethyl 2-cyclopentylsuccinate (4). Prepared according to GP3. Purified by flash column chromatography on silica gel (Cyclohexane:Ethyl Acetate 80:20) to afford the product as light yellow oil (81 mg, 76%). ^{1}H NMR (300 MHz, $CDCl_3$) δ 3.67 (s, 3H), 3.64 (s, 3H), 2.79 – 2.60 (m, 2H), 2.52 – 2.42 (m, 1H), 2.02 – 1.86 (m, 1H), 1.81 – 1.44 (m, 6H), 1.33 – 1.08 (m, 2H). ^{13}C NMR (75 MHz, $CDCl_3$) δ 175.5, 172.8, 51.8, 51.7, 46.3, 42.5, 35.5, 30.6, 30.5, 25.0, 25.0. Spectroscopic data are in accordance with the literature.[30]

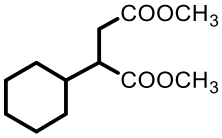

Dimethyl 2-cyclohexylsuccinate (5). Prepared according to GP3. Purified by flash column chromatography on silica gel (Cyclohexane:Ethyl Acetate 90:10) to afford the product as light yellow oil (81 mg, 71%). ^{1}H NMR (400 MHz, $CDCl_3$) δ 3.65 (s, 3H), 3.62 (s, 3H), 2.74 – 2.63 (m, 2H), 2.42 (dt, J_1 = 13 Hz, J_2 = 9 Hz, 1H), 1.76 – 1.66 (m, 2H), 1.65 – 1.51 (m, 4H), 1.26 – 0.90 (m, 5H). ^{13}C NMR (101 MHz, $CDCl_3$) δ 175.1, 173.0, 51.8, 51.7, 47.1, 40.0, 33.3, 30.7, 30.2, 26.3 (2C), 26.2. Spectroscopic data are in accordance with the literature.[30]

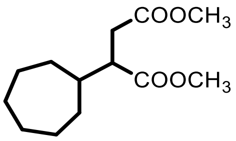

Dimethyl 2-cycloheptylsuccinate (6). Prepared according to GP3. Purified by flash column chromatography on silica gel (Cyclohexane:Ethyl Acetate 75:25) to afford the product as light yellow oil (94 mg, 78%). ^{1}H NMR (400 MHz, $CDCl_3$) δ 3.64 (s, 3H), 3.62 (s, 3H), 2.82 – 2.76 (m, 1H), 2.70 (dd, J_1 = 16 Hz, J_2 = 11 Hz, 1H), 2.36 (dd, J_1 = 16 Hz, J_2 = 3 Hz, 1H), 1.87 – 1.76 (m, 1H), 1.68 – 1.48 (m, 6H), 1.47 – 1.29 (m, 4H), 1.29 – 1.27 (m, 2H). ^{13}C (101 MHz, $CDCl_3$) δ 174.9, 173.1, 51.8, 51.7, 47.5, 41.2, 32.7, 32.2, 31.2, 28.0, 27.9, 26.8, 26.7. HRMS (FI) m/z calcd for $C_{13}H_{23}O_4^+$: 243.1596 $[M+H]^+$; found: 243.1598.

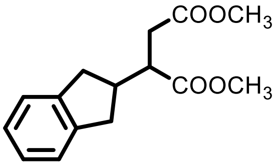

Dimethyl 2-(2,3-dihydro-1H-inden-2-yl)succinate (7). Prepared according to GP3. Purified by flash column chromatography on silica gel (Cyclohexane:Ethyl Acetate 90:10) to afford the product as light yellow oil (80 mg, 61%). ^{1}H NMR (300 MHz, $CDCl_3$) δ 7.22 – 7.08 (m, 4H), 3.70 (s, 3H), 3.68 (s, 3H), 3.11 – 2.92 (m, 3H), 2.89 – 2.61 (m, 4H), 2.54 (dd, J_1 = 16 Hz, J_2 = 4 Hz, 1H). ^{13}C NMR (75 MHz, $CDCl_3$) δ 174.7, 172.5, 142.4, 142.1, 126.5, 126.5, 124.4, 124.3, 51.9, 51.9, 45.9, 42.0, 37.1, 36.9, 35.0. HRMS (FI) m/z calcd for $C_{15}H_{18}O_4$: 262.1205; found: 262.1212.

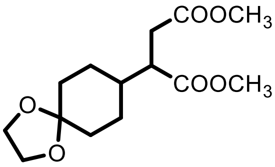

Dimethyl 2-(1,4-dioxaspiro[4.5]decan-8-yl)succinate (8). Prepared according to GP3. Purified by flash column chromatography on silica gel (Cyclohexane:Ethyl Acetate 75:25) to afford the product as yellow oil (114 mg, 80%). ^{1}H NMR (400 MHz, $CDCl_3$) δ 3.88 – 3.78 (m, 4H), 3.62 (s, 3H), 3.58 (s, 3H), 2.75 – 2.59 (m, 2H), 2.46 – 2.32 (m, 1H), 1.72 – 1.63 (m, 2H), 1.62 – 1.49 (m, 3H), 1.48 – 1.37 (m, 2H), 1.37 – 1.21 (m, 2H). ^{13}C NMR (101 MHz, $CDCl_3$) δ 174.7, 172.7, 108.2, 64.2, 64.2, 51.7, 51.7, 46.0, 38.5, 34.5, 34.4, 33.2, 27.7, 27.0. HRMS (FI) m/z calcd for $C_{14}H_{22}O_6$: 286.1416; found: 286.1418.

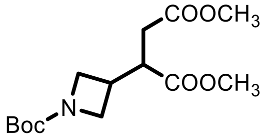

Dimethyl 2-(1-(*tert*-butoxycarbonyl)azetidin-3-yl)succinate (9). Prepared according to GP3. Purified by flash column chromatography on silica gel (Cyclohexane:Ethyl Acetate 75:25 →

0:100) to afford the product as brown oil (96 mg, 64%). ^{1}H NMR (400 MHz, $CDCl_3$) δ 3.97 (q, J = 9 Hz, 2H), 3.76 (dd, J_1 = 9 Hz, J_2 = 6 Hz, 1H), 3.68 (s, 3H), 3.66 (s, 3H), 3.62 (dd, J_1 = 9 Hz, J_2 = 5.9 Hz, 1H), 3.00 (td, J_1 = 10 Hz, J_2 = 4 Hz, 1H), 2.78 – 2.68 (m, 1H), 2.63 (dd, J_1 = 17 Hz, J_2=9 Hz, 1H), 2.41 (dd, J_1 = 17 Hz, J_2 = 4 Hz, 1H), 1.40 (s, 9H). ^{13}C NMR (101 MHz, $CDCl_3$) δ 173.2, 171.7, 156.1, 79.5, 52.9 (bs), 52.2, 52.0, 44.5, 33.5, 30.3, 28.4. HRMS (FI) m/z calcd for $C_{14}H_{23}NO_6$: 301.1525; found: 301.1538.

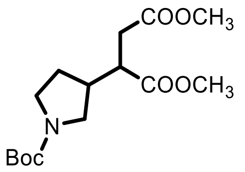

Dimethyl 2-(1-(*tert*-butoxycarbonyl)pyrrolidin-3-yl)succinate (10). Prepared according to GP3. Purified by flash column chromatography on silica gel (Cyclohexane:Ethyl Acetate 75:25 → 0:100) to afford the product as mixture of diastereomers as a brown oil (115 mg, 73%, dr 3:1). ^{1}H NMR (400 MHz, $CDCl_3$) δ 3.61 – 3.45 (m, 6H), 3.45 – 3.22 (m, 2H), 3.12 – 2.99 (m, 1H), 2.94 – 2.72 (m, 1H), 2.66 – 2.54 (m, 2H), 2.41 – 2.22 (m, 1H), 2.22 – 2.05 (m, 1H), 1.88 – 1.70 (m, 1H), 1.57 – 1.35 (m, 1H), 1.27 (d, J = 1.3 Hz, 9H). ^{13}C NMR (101 MHz, $CDCl_3$) δ 174.0, 173.9, 171.9, 171.8, 154.3, 154.2, 79.2, 79.2, 51.9, 51.9, 51.8, 49.4, 49.3, 49.2, 45.5, 45.4, 45.2, 45.1, 44.1, 43.9, 40.8, 40.5, 39.9, 39.9, 35.0, 34.6, 29.9, 29.8, 29.1, 29.0, 28.4. HRMS (FI) m/z calcd for $C_{15}H_{25}NO_6$: 315.1682; found: 315.1676 (dr 1) and 315.1669 (dr 2).

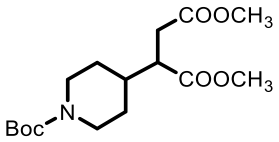

Dimethyl 2-(1-(*tert*-butoxycarbonyl)piperidin-4-yl)succinate (11). Prepared according to GP3. Purified by flash column chromatography on silica gel (Cyclohexane:Ethyl Acetate 50:50) to afford the product as yellow oil (94 mg, 57%). ^{1}H NMR (300 MHz, $CDCl_3$) δ 4.09 (d, J = 14.4 Hz, 2H), 3.67 (s, 3H), 3.63 (s, 3H), 2.82 – 2.52 (m, 4H), 2.50 – 2.34 (m, 1H), 1.80 – 1.64 (m, 1H), 1.63 – 1.46 (m, 2H), 1.41 (s, 9H), 1.29 – 1.10 (m, 2H). ^{13}C NMR (75 MHz, $CDCl_3$) δ 174.3, 172.5, 154.7, 79.5, 51.9, 51.8, 46.2, 43.8, 38.3, 33.2, 29.6, 29.3, 28.4. HRMS (FI) m/z calcd for $C_{16}H_{27}NO_6$: 329.1838; found: 329.1838.

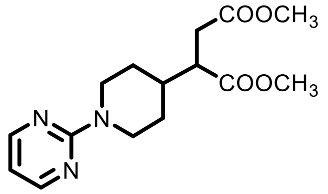

Dimethyl 2-(1-(pyrimidin-2-yl)piperidin-4-yl)succinate (12). Prepared according to GP3. Purified by flash column chromatography on silica gel (Cyclohexane:Ethyl Acetate 70:30) to afford the product as brown oil (94 mg, 61%). ^{1}H NMR (300 MHz, $CDCl_3$) δ 8.21 (s, 1H), 8.19 (s, 1H), 6.37 (t, J = 4.7 Hz, 1H), 4.80 – 4.68 (m, 2H), 3.63 (s, 3H), 3.59 (s, 3H), 2.81 – 2.62 (m, 4H), 2.50 – 2.34 (m, 1H), 1.89 – 1.74 (m, 1H), 1.69 – 1.53 (m, 2H), 1.32 – 1.14 (m, 2H). ^{13}C NMR (75 MHz, $CDCl_3$) δ 174.4, 172.5, 161.4, 157.7 (2C), 109.5, 51.8, 51.8, 46.3, 43.9, 43.8, 38.6, 33.2, 29.5, 29.2. HRMS (ESI) m/z calcd for $C_{15}H_{22}N_3O_4^+$: 308.1605 $[M+H]^+$; found: 308.1599.

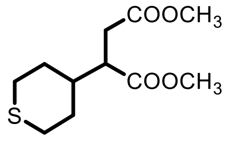

Dimethyl 2-(tetrahydro-2H-thiopyran-4-yl)succinate (13). Prepared according to GP3. Purified by flash column chromatography on silica gel (Pentane:Ethyl Acetate 94:6) to afford the product as yellowish oil (75 mg, 61%). ^{1}H NMR (300 MHz, $CDCl_3$) δ 3.67 (s, 3H), 3.63 (s, 3H), 2.80 – 2.50 (m, 6H), 2.47 – 2.32 (m, 1H), 1.96 – 1.84 (m, 2H), 1.73 – 1.60 (m, 1H), 1.54 – 1.36 (m, 2H). ^{13}C NMR (75 MHz, $CDCl_3$) δ 174.2, 172.7, 51.9, 47.0, 39.5, 32.6, 31.7, 31.3, 28.9. HRMS (FI) m/z calcd for $C_{11}H_{18}O_4S$: 246.0926; found: 246.0925.

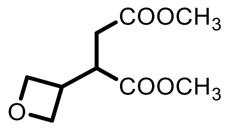

Dimethyl 2-(oxetan-3-yl)succinate (14). Prepared according to GP3. Purified by flash column chromatography on silica gel (Cyclohexane:Ethyl Acetate 80:20) to afford the product as colorless oil (40 mg, 40%). ^{1}H NMR (400 MHz, $CDCl_3$) δ 4.77 – 4.67 (m, 2H), 4.56 (t, J = 6 Hz, 1H), 4.43 (t, J = 6 Hz, 1H), 3.67 (s, 3H), 3.66 (s, 3H), 3.28 – 3.15 (m, 2H), 2.66 – 2.53 (m, 1H), 2.40 (dd, J_1 = 17 Hz, J_2 = 4 Hz, 1H). ^{13}C NMR (101 MHz, $CDCl_3$) δ 173.3, 171.8, 75.9, 75.1, 52.2, 52.1, 44.2, 36.6, 33.4. HRMS (FI) m/z calcd for $C_9H_{14}O_5$: 202.0841; found: 202.0854.

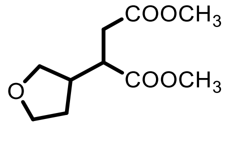

Dimethyl 2-(tetrahydrofuran-3-yl)succinate (15). Prepared according to GP3. Purified by flash column chromatography on silica gel (Cyclohexane:Ethyl Acetate 50:50 → 0:100) to afford the product as mixture of diastereomers as a yellow oil (83 mg, 77%, dr 1:1). ^{1}H NMR (300 MHz, $CDCl_3$) δ 3.96 – 3.81 (m, 4H), 3.78 – 3.63 (m, 14H), 3.56 – 3.41 (m, 2H), 2.85 –

2.70 (m, 4H), 2.60 – 2.33 (m, 4H), 2.09 – 1.91 (m, 2H), 1.79 – 1.54 (m, 2H). ^{13}C NMR (75 MHz, $CDCl_3$) δ 174.5, 174.4, 172.3, 172.1, 71.5, 71.1, 68.1, 68.0, 52.1, 52.1, 52.0 (2C), 44.2, 44.1, 41.3, 41.1, 35.3, 35.0, 30.4, 30.2. HRMS (FI) m/z calcd for $C_{10}H_{16}O_5$: 216.0998; found: 216.0981.

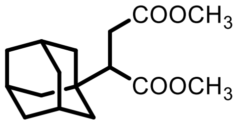

Dimethyl 2-((3r,5r,7r)-adamantan-1-yl)succinate (16). Prepared according to GP3. Purified by flash column chromatography on silica gel (Cyclohexane:Ethyl Acetate 80:20) to afford the product as light yellow solid (111 mg, 79%), m.p. 56.6~57.4 °C ^{1}H NMR (300 MHz, $CDCl_3$) δ 3.64 (s, 3H), 3.60 (s, 3H), 2.71 (dd, J_1 = 17 Hz, J_2 = 12 Hz, 1H), 2.52 – 2.39 (m, 2H), 1.99 – 1.88 (m, 3H), 1.70 – 1.51 (m, 9H), 1.48 – 1.33 (m, 3H). ^{13}C NMR (75 MHz, $CDCl_3$) δ 174.3, 173.4, 52.3, 51.8, 51.3, 40.0, 36.8, 34.4, 31.0, 28.5. Spectroscopic data are in accordance with the literature.[31]

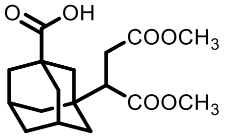

(1s,3r,5R,7S)-3-(1,4-Dimethoxy-1,4-dioxobutan-2-yl)adamantane-1-carboxylic acid (17). Prepared according to GP3, **2b** (0.5 mmol), **1y** (1.2 equiv.), **B1** (1.2 equiv.) for 3 h. Purified by flash column chromatography on silica gel (CH_2Cl_2→Cyclohexane:Ethyl Acetate 75:25→50:50) to afford the product as light yellow oil (91 mg, 56%). ^{1}H NMR (300 MHz, $CDCl_3$) δ 3.69 (s, 3H), 3.64 (s, 3H), 2.83 – 2.69 (m, 1H), 2.60 – 2.44 (m, 2H), 2.17 – 2.04 (m, 2H), 1.92 – 1.71 (m, 5H), 1.70 – 1.52 (m, 5H), 1.49 – 1.38 (m, 2H). ^{13}C NMR (75 MHz, $CDCl_3$) δ 183.5, 174.1, 173.2, 52.0, 51.9, 51.6, 41.3, 40.9, 39.1, 38.8, 38.0, 37.9, 35.6, 34.8, 31.1, 28.2 (2C). HRMS (ESI$^-$) m/z calcd for $C_{17}H_{23}O_6^-$: 323.1500 [M-H]$^-$; found: 323.1484.

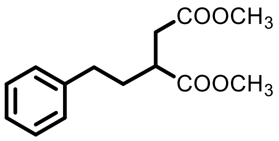

Dimethyl 2-phenethylsuccinate (18). Prepared according to GP4. Purified by flash column chromatography on silica gel (Cyclohexane:Ethyl Acetate 85:15) to afford the product as colorless oil (71 mg, 57%). ^{1}H NMR (400 MHz, CD_2Cl_2) δ 7.34 – 7.25 (m, 2H), 7.24 – 7.14 (m, 3H), 3.69 (s, 3H), 3.65 (s, 3H), 2.90 – 2.81 (m, 1H), 2.74 (dd, J_1 = 16 Hz, J_2 = 9 Hz, 1H), 2.68 – 2.57 (m, 2H), 2.49 (dd, J_1 = 16 Hz, J_2 = 5 Hz, 1H), 2.03 – 1.92 (m, 1H), 1.88 – 1.77 (m,

1H). ^{13}C NMR (101 MHz, CD_2Cl_2) δ 175.3, 172.5, 141.8, 128.8 (2C), 126.4, 52.1, 52.0, 41.2, 36.2, 34.0, 33.5. Spectroscopic data are in accordance with the literature.[32]

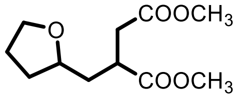

Dimethyl 2-((tetrahydrofuran-2-yl)methyl)succinate (19). Prepared according to GP4. Purified by flash column chromatography on silica gel (Cyclohexane:Ethyl Acetate 70:30) to afford the product as mixture of diastereomers as a yellow oil (47 mg, 41%, dr: 3:2). ^{1}H NMR (400 MHz, CD_2Cl_2) δ 3.85 – 3.72 (m, 4H), 3.69 – 3.60 (m, 14H), 2.99 – 2.87 (m, 2H), 2.71 (dd, J_1 = 9 Hz, J_2 = 5 Hz, 1H), 2.66 (dd, J_1 = 9 Hz, J_2 = 5 Hz, 1H), 2.56 (dd, J_1 = 7 Hz, J_2 = 5 Hz, 1 H), 2.52 (dd, J_1 = 8 Hz, J_2 = 5 Hz, 1H), 2.04 – 1.92 (m, 2H), 1.91 – 1.77 (m, 6H), 1.67 – 1.58 (m, 2H), 1.49 – 1.36 (m, 2H). ^{13}C NMR (101 MHz, CD_2Cl_2) δ 175.6 (major), 175.5 (minor), 172.6 (major), 172.6 (minor), 77.4 (minor), 76.8 (major), 68.0 (minor), 68.0 (major), 52.1 (major), 52.0 (minor), 51.9 (major), 51.9 (minor), 39.6 (minor), 39.3 (major), 38.1 (minor), 37.8 (major), 36.6 (minor), 35.8 (major), 32.1 (minor), 31.9 (major), 26.0 (major), 25.9 (minor). HRMS (FI) m/z calcd for $C_{11}H_{18}O_5$: 230.1154; found: 230.1156.

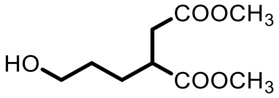

Dimethyl 2-(3-hydroxypropyl)succinate (20). Prepared according to GP4. Reaction time: 18 h. Purified by flash column chromatography on silica gel (Cyclohexane:Ethyl Acetate 50:50; second column: Ethyl Acetate:Cyclohexane 70:30) to afford the product as yellow oil (42 mg, 41%).^{1}H NMR (300 MHz, $CDCl_3$) δ 3.68 (s, 3H), 3.65 (s, 3H), 3.61 (t, J = 6.0 Hz, 2H), 2.92 – 2.79 (m, 1H), 2.71 (dd, J_1 = 16 Hz, J_2 = 9 Hz, 1H), 2.44 (dd, J_1 = 16 Hz, J_2 = 5 Hz, 1H), 1.97 – 1.80 (m, 1H), 1.77 – 1.47 (m, 4H). ^{13}C NMR (75 MHz, $CDCl_3$) δ 175.4, 172.5, 62.3, 52.0, 51.9, 40.9, 36.0, 30.0, 28.2. HRMS (FI) m/z calcd for $C_9H_{16}O_5$: 204.0998; found: 204.0986.

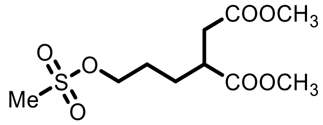

Dimethyl 2-(3-((methylsulfonyl)oxy)propyl)succinate (21). Prepared according to GP4. Reaction time: 18 h. Purified by flash column chromatography on silica gel (Cyclohexane:Ethyl Acetate 80:20 → 60:40) to afford the product as yellow oil (80 mg, 57%). ^{1}H NMR (400 MHz, $CDCl_3$) δ 4.19 (t, J = 6 Hz, 2H), 3.67 (s, 3H), 3.64 (s, 3H), 2.98 (s, 3H), 2.89 – 2.78 (m, 1H), 2.71 (dd, J_1 = 17 Hz, J_2 = 9 Hz, 1H), 2.42 (dd, J_1 = 17 Hz, J_2 = 6 Hz, 1H),

1.84 – 1.57 (m, 4H). ^{13}C NMR (101 MHz, $CDCl_3$) δ 174.7, 172.1, 69.3, 52.1, 51.9, 40.6, 37.4, 35.9, 27.8, 26.7. HRMS (FI) m/z calcd for $C_{10}H_{18}O_7S$: 282.0773; found: 282.0761.

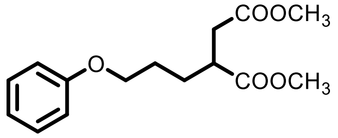

Dimethyl 2-(3-phenoxypropyl)succinate (22). Prepared according to GP4. Purified by flash column chromatography on silica gel (Cyclohexane:Ethyl Acetate 80:20) to afford the product as light yellow oil (56 mg, 40%). ^{1}H NMR (400 MHz, CD_2Cl_2) δ 7.32 – 7.22 (m, 2H), 6.97 – 6.85 (m, 3H), 3.98 – 3.92 (t, J = 6 Hz, 2H), 3.68 (s, 3H), 3.65 (s, 3H), 2.94 – 2.84 (m, 1H), 2.73 (dd, J_1 = 16 Hz, J_2 = 9 Hz, 1H), 2.48 (dd, J_1 = 16 Hz, J_2 = 5 Hz, 1H), 1.88 – 1.68 (m, 4H). ^{13}C NMR (101 MHz, CD_2Cl_2) δ 175.4, 172.6, 159.4, 129.8, 121.0, 114.8, 67.7, 52.1, 52.0, 41.3, 36.2, 28.9, 27.2. HRMS (FI) m/z calcd for $C_{15}H_{20}O_5$: 280.1311; found: 280.1314.

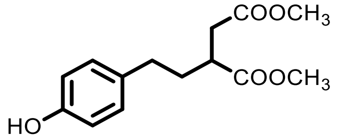

Dimethyl 2-(4-hydroxyphenethyl)succinate (23). Prepared according to GP4, **2b** (0.5 mmol), **1aj** (1 equiv.). Purified by flash column chromatography on silica gel (Cyclohexane:Ethyl Acetate 65:35) to afford the product as light yellow oil (55 mg, 41%). Consumption of **2b**: 56%. ^{1}H NMR (400 MHz, $CDCl_3$) δ 7.02 (d, J = 8 Hz, 2H), 6.75 (d, J = 8 Hz, 2H), 5.36 (s, 1H), 3.70 (s, 3H), 3.67 (s, 3H), 2.92 – 2.83 (m, 1H), 2.75 (dd, J_1 = 17 Hz, J_2 = 9 Hz, 1H), 2.58 – 2.52 (m, 2H), 2.48 (dd, J_1 = 17 Hz, J_2 = 5 Hz, 1H), 2.02 – 1.88 (m, 1H), 1.82 – 1.73 (m, 1H). ^{13}C NMR (101 MHz, $CDCl_3$) δ 175.5, 172.6, 154.2, 133.1, 129.6 (2C), 115.4 (2C), 52.1, 52.0, 40.9, 36.0, 33.9, 32.4. HRMS (EI) m/z calcd for $C_{14}H_{18}O_5$: 266.1154; found: 266.1164.

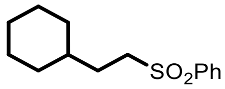

((2-Cyclohexylethyl)sulfonyl)benzene (24). Prepared according to GP3. Purified by flash column chromatography on silica gel (Cyclohexane:Ethyl Acetate 75:25) to afford the product as light yellow oil (91 mg, 72%). ^{1}H NMR (300 MHz, $CDCl_3$) δ 7.96 – 7.84 (m, 2H), 7.70 – 7.61 (m, 1H), 7.60 – 7.50 (m, 2H), 3.14 – 3.03 (m, 2H), 1.75 – 1.52 (m, 7H), 1.36 – 0.98 (m, 4H), 0.94 – 0.75 (m, 2H). ^{13}C NMR (75 MHz, $CDCl_3$) δ 139.2, 133.6, 129.2, 128.0, 54.3, 36.5, 32.7, 29.6, 26.2, 25.9. Spectroscopic data are in accordance with the literature.[33]

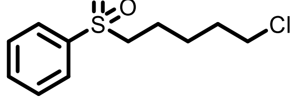

((5-Chloropentyl)sulfonyl)benzene (25). Prepared according to GP4. Purified by flash column chromatography on silica gel (Cyclohexane:Ethyl Acetate 85:15) to afford the product as colorless oil (84 mg, 68%). ^{1}H NMR (400 MHz, $CDCl_3$) δ 7.94 – 7.87 (m, 2H), 7.71 – 7.62 (m, 1H), 7.62 – 7.53 (m, 2H), 3.49 (t, J = 6 Hz, 2H), 3.17 – 3.05 (m, 2H), 1.82 – 1.70 (m, 4H), 1.58 – 1.48 (m, 2H). ^{13}C NMR (101 MHz, $CDCl_3$) δ 139.3, 133.9, 129.5, 128.2, 56.2, 44.5, 32.0, 25.7, 22.2. HRMS (FI) m/z calcd for $C_{11}H_{15}ClO_2S$: 246.0481; found: 246.0486.

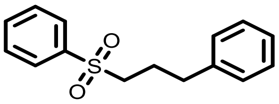

((3-Phenylpropyl)sulfonyl)benzene (26). Prepared according to GP4. Purified by flash column chromatography on silica gel (Cyclohexane:Ethyl Acetate 80:20) to afford the product as solid (42 mg, 32%), m.p. 79.0~81.4 °C ^{1}H NMR (300 MHz, CD_2Cl_2) δ 7.92 – 7.84 (m, 2H), 7.72 – 7.64 (m, 1H), 7.62 – 7.53 (m, 2H), 7.31 – 7.23 (m, 2H), 7.22 – 7.15 (m, 1H), 7.14 – 7.06 (m, 2H), 3.13 – 3.03 (m, 2H), 2.68 (t, J = 7 Hz, 2H), 2.07 – 1.93 (m, 2H). ^{13}C NMR (75 MHz, CD_2Cl_2) δ 140.6, 139.6, 134.0, 129.7, 128.9, 128.8, 128.4, 126.7, 55.8, 34.4, 24.8. Spectroscopic data are in accordance with the literature.[34]

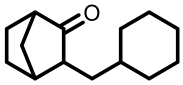

3-(Cyclohexylmethyl)bicyclo[2.2.1]heptan-2-one (27). Prepared according to GP3. Purified by flash column chromatography on silica gel (Cyclohexane:Ethyl Acetate 90:10) to afford the product as yellow oil (41 mg, 40%). ^{1}H NMR (400 MHz, $CDCl_3$) δ 2.61 – 2.54 (m, 2H), 2.08 (dt, J_1 = 10 Hz, J_2 = 4 Hz, 1H), 1.86 – 1.58 (m, 8H), 1.56 – 1.46 (m, 2H), 1.42 – 1.32 (m, 1H), 1.31 – 1.05 (m, 6H), 0.99 – 0.76 (m, 2H). ^{13}C NMR (101 MHz, $CDCl_3$) δ 221.0, 51.3, 50.6, 38.6, 37.2, 35.9, 34.3, 33.8, 32.3, 26.7, 26.4, 26.3, 25.5, 21.4. Spectroscopic data are in accordance with the literature.[35]

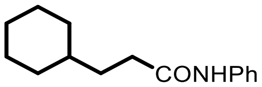

3-Cyclohexyl-N-phenylpropanamide (28). Prepared according to GP3. Solvent: ethyl acetate. Purified by flash column chromatography on silica gel (Cyclohexane:Ethyl Acetate 75:25) to afford the product as yellow solid (59 mg, 51%), m.p. 91.9~95.4 °C ^{1}H NMR (300 MHz, CD_2Cl_2) δ 7.64 – 7.40 (m, 3H), 7.39 – 7.24 (m, 2H), 7.14 – 7.03 (m, 1H), 2.40 – 2.29 (m, 2H), 1.84 – 1.53 (m, 7H), 1.38 – 1.07 (m, 4H), 1.05 – 0.84 (m, 2H). ^{13}C NMR (75 MHz, CD_2Cl_3) δ 172.1, 138.8, 129.2, 124.3, 120.1, 37.8, 35.6, 33.5, 33.4, 27.0, 26.7. Spectroscopic data are in accordance with the literature.[36]

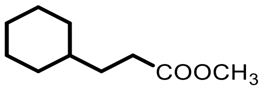

Methyl 3-cyclohexylpropanoate (29). Prepared according to GP3, ethyl acetate instead of acetonitrile as solvent. Purified by flash column chromatography on silica gel (Cyclohexane:Ethyl Acetate 95:5) to afford the product as yellow oil (50 mg, 59%). ^{1}H NMR (400 MHz, $CDCl_3$) δ 3.64 (s, 3H), 2.34 – 2.25 (m, 2H), 1.74 – 1.58 (m, 5H), 1.55 – 1.45 (m, 2H), 1.28 – 1.05 (m, 4H), 0.94 – 0.79 (m, 2H). ^{13}C NMR (101 MHz, $CDCl_3$) δ 174.7, 51.6, 37.3, 33.1, 32.5, 31.8, 26.6, 26.3. Spectroscopic data are in accordance with the literature.[37]

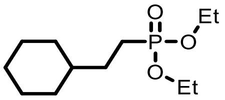

Diethyl (2-cyclohexylethyl)phosphonate (30). Prepared according to GP3. Purified by flash column chromatography on silica gel (Cyclohexane:Acetone 66:33) to afford the product as yellow oil (69 mg, 56%). ^{1}H NMR (300 MHz, $CDCl_3$) δ 4.14 – 3.93 (m, 4H), 1.74 – 1.54 (m, 7H), 1.49 – 1.36 (m, 2H), 1.26 (t, J = 7 Hz, 6H), 1.23 – 0.99 (m, 4H), 0.91 – 0.74 (m, 2H). ^{13}C NMR (75 MHz, $CDCl_3$) δ 61.4 (d, J = 6 Hz), 38.3 (d, J = 17 Hz), 32.8, 29.6 (d, J = 5 Hz), 26.5, 26.2, 23.2 (d, J = 140 Hz), 16.5 (d, J = 6 Hz). ^{31}P NMR (121 MHz, $CDCl_3$) δ 33.3. Spectroscopic data are in accordance with the literature.[38]

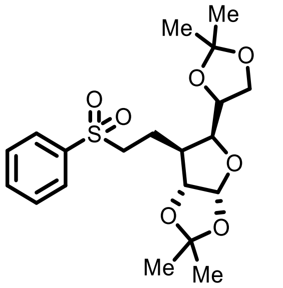

Major, 31

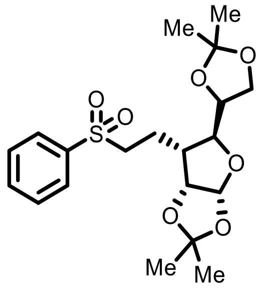

Minor, 31'

(3aR,5S,6S,6aR)-5-((R)-2,2-dimethyl-1,3-dioxolan-4-yl)-2,2-dimethyl-6-(2-(phenylsulfonyl)ethyl)tetrahydrofuro[2,3-d][1,3]dioxole (31) and (3aR,5S,6R,6aR)-5-((R)-2,2-dimethyl-1,3-dioxolan-4-yl)-2,2-dimethyl-6-(2-(phenylsulfonyl)ethyl)tetrahydrofuro[2,3-d][1,3]dioxole (31'). Prepared according to GP4. Reaction time: 3 h. Purified by flash column chromatography on silica gel to afford the product as mixture of diastereomers as a colorless oil (95 mg, 46% dr: 8:1), along with the reduction product (49 mg, 40%).[39]

Major Product **31**: purified by flash column chromatography on silica gel (Cyclohexane:Ethyl Acetate 75:25→50:50) to afford the product (85 mg, 41%). ^{1}H NMR (300 MHz, $CDCl_3$) δ 7.94 – 7.82 (m, 2H), 7.69 – 7.61 (m, 1H), 7.60 – 7.50 (m, 2H), 5.68 (d, J = 4 Hz, 1H), 4.34 (d, J =

4 Hz, 1H), 4.11 – 3.95 (m, 2H), 3.90 – 3.78 (m, 2H), 3.36 – 3.10 (m, 2H), 2.30 – 2.2 (m, 1H), 2.05 – 1.90 (m, 1H), 1.63 – 1.48 (m, 1H), 1.44 (s, 3H), 1.36 – 1.30 (m, 3H), 1.25 (s, 3H), 1.24 (s, 3H). ^{13}C NMR (75 MHz, $CDCl_3$) δ 138.9, 133.9, 129.4 (2C), 128.1 (2C), 111.5, 109.5, 104.6, 84.2, 80.4, 72.9, 68.6, 54.3, 45.1, 26.8, 26.6, 26.1, 25.3, 18.7. HRMS (ESI) m/z calcd for $C_{20}H_{29}O_7S^+$: 413.1629 $[M+H]^+$; found: 413.1639.

Minor Product **31'**: Purified by flash column chromatography on silica gel (Cyclohexane:Ethyl Acetate 75:25→50:50 second column: DCM:CH_3OH 99:1), to afford the product (10 mg, 5%). ^{1}H NMR (400 MHz, $CDCl_3$) δ 7.96 – 7.90 (m, 2H), 7.67 (t, *J* = 7 Hz, 1H), 7.57 (t, *J* = 8 Hz, 2H), 5.74 (d, *J* = 4 Hz, 1H), 4.60 (t, *J* = 4 Hz, 1H), 4.11 – 4.03 (m, 1H), 3.95 – 3.83 (m, 2H), 3.63 (dd, J_1 = 9 Hz, J_2 = 7 Hz, 1H), 3.36 – 3.19 (m, 2H), 2.21 – 2.10 (m, 1H), 2.09 – 1.99 (m, 1H), 1.99 – 1.87 (m, 1H), 1.42 (s, 3H), 1.32 (s, 3H), 1.30 (s, 3H), 1.28 (s, 3H). ^{13}C NMR (101 MHz, $CDCl_3$) δ 139.1, 133.8, 129.3 (2C), 128.3 (2C), 112.2, 109.7, 105.2, 82.2, 81.2, 77.8, 68.0, 54.4, 46.9, 26.8, 26.7, 26.5, 25.4, 19.6.

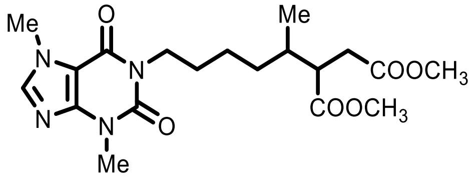

Dimethyl 2-(6-(3,7-dimethyl-2,6-dioxo-2,3,6,7-tetrahydro-1H-purin-1-yl)hexan-2-yl)succinate (32). Prepared according to GP4. Reaction time: 3 h. Purified by flash column chromatography on silica gel (Cyclohexane:Ethyl Acetate 30:70 → 0:100) to afford the product as mixture of diastereomers as a colorless oil (131 mg, 64%, dr: 1:1). ^{1}H NMR (300 MHz, $CDCl_3$) δ 7.47 (d, *J* = 1 Hz, 1H), 4.00 – 3.88 (m, 5H), 3.62 (s, 3H), 3.60 (s, 3H), 3.50 (s, 3H), 2.85 – 2.74 (m, 1H), 2.74 – 2.58 (m, 1H), 2.38 – 2.24 (m, 1H), 1.94 – 1.66 (m, 1H), 1.65 – 1.47 (m, 2H), 1.43 – 1.11 (m, 4H), 0.82 (dd, J_1 = 14 Hz, J_2 = 7 Hz, 3H). ^{13}C NMR (75 MHz, $CDCl_3$) δ 175.0, 174.6, 173.1, 172.9, 155.3, 151.5, 148.8, 141.5, 107.7, 51.8, 51.7, 51.6, 46.0, 45.7, 41.2, 41.2, 35.0, 34.6, 34.0, 33.6, 33.3, 31.4, 29.7, 28.1, 24.7, 24.6, 16.9, 16.2. HRMS (ESI) m/z calcd for $C_{19}H_{29}N_4O_6^+$: 409.2082 $[M+H]^+$; found: 409.2083.

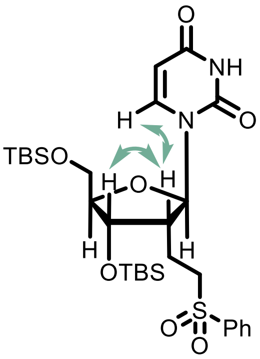

1-((2R,3S,4S,5R)-4-((tert-butyldimethylsilyl)oxy)-5-(((tert-butyldimethylsilyl)oxy)methyl)-3-(2-(phenylsulfonyl)ethyl)tetrahydrofuran-2-yl)pyrimidine-2,4(1H,3H)-dione (33). Prepared according to GP4. Reaction time: 3 h. Purified by flash column chromatography on silica gel (DCM → Cyclohexane:Ethyl Acetate 50:50, second column with reverse phase C_{18}, H_2O:CH_3CN 30:70 → 10:90) to afford the product as a colorless oil (134 mg, 43%), along with the reduction product **33'** (68 mg, 30%). ^1H NMR (400 MHz, $CDCl_3$) δ 9.75 (d, J = 2 Hz, 1H), 7.89 – 7.79 (m, 3H), 7.64 – 7.57 (m, 1H), 7.55 – 7.47 (m, 2H), 5.92 (d, J = 7 Hz, 1H), 5.72 (dd, J_1 = 8 Hz, J_2 = 2.0 Hz, 1H), 4.27 (dd, J_1 = 6 Hz, J_2 = 3 Hz, 1H), 3.93 – 3.88 (m, 1H), 3.84 (dd, J_1 = 11 Hz, J_2 = 3 Hz, 1H), 3.71 (dd, J_1 = 11 Hz, J_2 = 2 Hz, 1H), 3.24 – 3.05 (m, 2H), 2.40 – 2.28 (m, 1H), 2.16 – 2.01 (m, 1H), 1.80 – 1.67 (m, 1H), 0.90 (s, 9H), 0.80 (s, 9H), 0.11 – 0.07 (m, 6H), 0.02 (s, 3H), -0.01 (s, 3H). ^{13}C NMR (101 MHz, $CDCl_3$) δ 163.6, 150.8, 140.2, 138.6, 133.9, 129.4 (2C), 128.1 (2C), 102.8, 88.2, 86.8, 72.5, 62.9, 53.7, 48.2, 26.0 (3C), 25.7 (3C), 18.6, 18.5, 17.9, -4.5, -4.9, -5.5, -5.5. HRMS (ESI) m/z calcd for $C_{29}H_{49}N_2O_7SSi_2^+$: 625.2794 [M+H]$^+$; found: 629.2791.

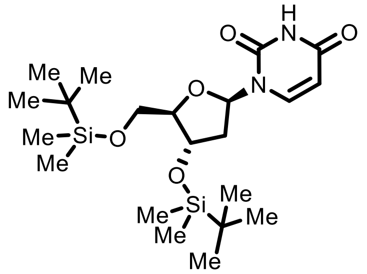

1-((2R,4S,5R)-4-((tert-butyldimethylsilyl)oxy)-5-(((tert-butyldimethylsilyl)oxy)methyl)tetrahydrofuran-2-yl)pyrimidine-2,4(1H,3H)-dione (33'-side product). Purified by flash column chromatography on silica gel (Cyclohexane:Ethyl Acetate 90:10 → 75:25) to afford the product as a colorless oil (68 mg, 30%). ^1H NMR (300 MHz, $CDCl_3$) δ 9.73 (s, 1H), 7.87 (d, J = 8 Hz, 1H), 6.27 (t, J = 6 Hz, 1H), 5.67 (dd, J_1 = 8 Hz, J_2 = 2 Hz, 1H), 4.39 (dt, J_1 = 6 Hz, J_2 = 4 Hz, 1H), 3.93 – 3.82 (m, 2H), 3.79 – 3.68 (m, 1H), 2.34 – 2.26 (m, 1H), 2.13 – 1.97 (m, 1H), 0.89 (s, 9H), 0.86 (s, 9H), 0.08 (s, 6H), 0.05 (s, 3H), 0.05 (s, 3H). ^{13}C NMR (75 MHz, $CDCl_3$) δ 163.9, 150.6, 140.2, 102.3, 87.8, 85.2, 71.1, 62.4, 41.9, 25.9 (3C), 25.8 (3C), 18.4, 18.0, -4.5, -4.8, -5.4, -5.5.

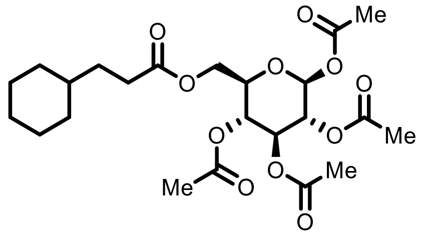

(2S,3R,4S,5R,6R)-6-(((3-cyclohexylpropanoyl)oxy)methyl)tetrahydro-2H-pyran-2,3,4,5-tetrayl tetraacetate (34). Prepared according to GP3. Purified by flash column chromatography on silica gel (Cyclohexane:Ethyl Acetate 50:50) to afford the product as colourless solid (160 mg, 66%), m.p. 105.6~106.5 ºC ^{1}H NMR (300 MHz, $CDCl_3$) δ 5.68 (d, J = 8 Hz, 1H), 5.22 (t, J = 9 Hz, 1H), 5.15 – 5.02 (m, 2H), 4.23 (dd, J_1 = 12 Hz, J_2 = 5 Hz, 1H), 4.15 – 4.03 (m, 1H), 3.81 (ddd, J_1 = 10 Hz, J_2 = 5 Hz, J_3 = 2 Hz, 1H), 2.37 – 2.26 (m, 2H), 2.08 (s, 3H), 2.05 – 1.94 (m, 9H), 1.75 – 1.55 (m, 5H), 1.54 – 1.41 (m, 2H), 1.29 – 1.03 (m, 4H), 0.96 – 0.77 (m, 2H). ^{13}C NMR (75 MHz, $CDCl_3$) δ 173.7, 170.1, 169.3, 169.3, 168.9, 91.7, 72.9, 72.8, 70.3, 67.9, 61.3, 37.2, 33.0 (2C), 32.1, 31.6, 26.6, 26.3 (2C), 20.8, 20.6 (2C), 20.6. HRMS (EI) m/z calcd for $C_{21}H_{31}O_9^+$: 427.1968 [M-CH_3COO^-]$^+$; found: 427.1978.

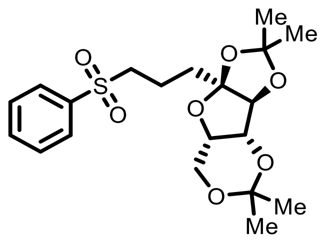

(3aS,3bR,7aS,8aS)-2,2,5,5-Tetramethyl-8a-(3-(phenylsulfonyl)propyl)tetrahydro-7H-[1,3]dioxolo[4',5':4,5]furo[3,2-d][1,3]dioxine (35). Purified by flash column chromatography on silica gel (CH_2Cl_2 → Cyclohexane:Ethyl Acetate 50:50) to afford the product as light yellow oil (GP4: 142 mg, 69%; GP6: 142 mg, 69%). ^{1}H NMR (300 MHz, $CDCl_3$) δ 7.92 – 7.82 (m, 2H), 7.64 – 7.56 (m, 1H), 7.55 – 7.46 (m, 2H), 4.18 (d, J = 2 Hz, 1H), 4.15 (s, 1H), 4.04 – 3.83 (m, 3H), 3.28 – 3.07 (m, 2H), 2.10 – 1.84 (m, 4H), 1.39 (s, 3H), 1.35 (s, 3H), 1.30 (s, 3H), 1.24 (s, 3H). ^{13}C NMR (75 MHz, $CDCl_3$) δ 139.1, 133.6, 129.2, 128.1 114.7, 111.1, 97.3, 86.6, 73.5, 72.0, 60.3, 56.2, 36.2, 28.8, 27.3, 26.6, 18.7, 17.8. HRMS (FD) m/z calcd for $C_{20}H_{29}O_7S^+$: 413.1629 [M+H]$^+$; found: 413.1632.

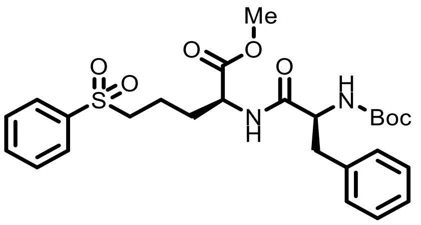

Methyl (S)-2-((S)-2-((tert-butoxycarbonyl)amino)-3-phenylpropanamido)-5-(phenylsulfonyl)pentanoate (36). Purified by flash column chromatography on silica gel (Cyclohexane:Ethyl Acetate 50:50) to afford the product as colourless solid (GP4: 196 mg, 76%; GP6 [CH_3CN/H_2O 9:1 as solvent]: 179 mg, 69%). m.p. 113.3~116.4 ºC. ^{1}H NMR (300 MHz, $CDCl_3$) δ 7.92 – 7.82 (m, 2H), 7.70 – 7.60 (m, 1H), 7.60 – 7.47 (m, 2H), 7.31 – 7.13 (m,

5H), 6.54 (d, $J = 8$ Hz, 1H), 4.95 (d, $J = 8$ Hz, 1H), 4.47 (td, $J_1 = 7$ Hz, $J_2 = 5$ Hz, 1H), 4.31 (d, $J = 7$ Hz, 1H), 3.66 (s, 3H), 3.21 – 2.90 (m, 4H), 1.98 – 1.80 (m, 1H), 1.79 – 1.63 (m, 3H), 1.39 (s, 9H). ^{13}C NMR (75 MHz, $CDCl_3$) δ 171.68, 171.36, 155.39, 139.14, 136.50, 133.86, 129.43, 129.35, 128.78, 128.14, 127.10, 80.45, 55.90, 55.36, 52.66, 51.44, 38.22, 31.20, 28.33, 18.80. HRMS (ESI) m/z calcd for $C_{26}H_{35}N_2O_7S^+$: 519.2159 $[M+H]^+$; found: 519.2155.

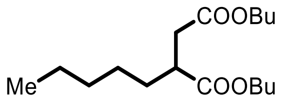

Dibutyl 2-pentylsuccinate (37). Prepared according to GP5. Purified by flash column chromatography on silica gel (Pentane:Ethyl Acetate 92:8) to afford the product as colorless oil (66 mg, 44%). ^{1}H NMR (300 MHz, $CDCl_3$) δ 4.12 – 3.99 (m, 4H), 2.84 – 2.75 (m, 1H), 2.67 (dd, $J_1 = 16$ Hz, $J_2 = 9$ Hz, 1H), 2.38 (dd, $J_1 = 16$ Hz, $J_2 = 5$ Hz, 1H), 1.63 – 1.19 (m, 16H), 0.94 – 0.79 (m, 9H). ^{13}C NMR (75 MHz, $CDCl_3$) δ 175.1, 172.2, 64.5, 64.5, 41.4, 36.2, 32.0, 31.6, 30.7, 30.7, 26.6, 22.5, 19.2, 19.2, 14.0, 13.7 (2C). HRMS (FI) m/z calcd for $C_{17}H_{32}O_4$: 300.2301; found: 300.2310.

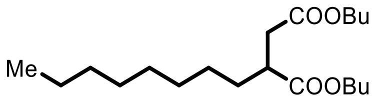

Dibutyl 2-octylsuccinate (38). Prepared according to GP5. Purified by flash column chromatography on silica gel (Pentane:Ethyl Acetate 92:8) to afford the product as colorless oil (77 mg, 45%). ^{1}H NMR (300 MHz, $CDCl_3$) δ 4.14 – 4.00 (m, 4H), 2.86 – 2.74 (m, 1H), 2.68 (dd, $J_1 = 16$ Hz, $J_2 = 9$ Hz, 1H), 2.39 (dd, $J_1 = 16$, $J_2 = 5$ Hz, 1H), 1.64 – 1.17 (m, 22H), 0.94 – 0.80 (m, 9H). ^{13}C NMR (75 MHz, $CDCl_3$) δ 175.2, 172.1, 64.5, 64.5, 41.4, 36.2, 32.1, 31.9, 30.8, 30.7, 29.5, 29.5, 29.3, 27.0, 22.7, 19.2, 19.2, 14.2, 13.8 (2C). HRMS (FI) m/z calcd for $C_{20}H_{38}O_4$: 342.2770; found: 342.2765.

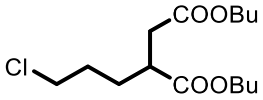

Dibutyl 2-(3-chloropropyl)succinate (39). Prepared according to GP5. Purified by flash column chromatography on silica gel (Cyclohexane:Ethyl Acetate 90:10) to afford the product as colorless oil (83 mg, 54%). ^{1}H NMR (300 MHz, $CDCl_3$) δ 4.17 – 4.01 (m, 4H), 3.61 – 3.47 (m, 2H), 2.92 – 2.80 (m, 1H), 2.73 (dd, $J_1 = 16$ Hz, $J_2 = 9$ Hz, 1H), 2.43 (dd, $J_1 = 16$ Hz, $J_2 = 5$ Hz, 1H), 1.91 – 1.67 (m, 4H), 1.67 – 1.53 (m, 4H), 1.47 – 1.30 (m, 4H), 0.93 (td, $J_1 = 7.3$ Hz, $J_2 = 2.1$ Hz, 6H). ^{13}C NMR (75 MHz, $CDCl_3$) δ 174.6, 171.9, 64.8, 64.8, 44.6, 40.8, 36.3, 30.8, 30.8, 30.1, 29.3, 19.3, 19.2, 13.8 (2C). HRMS (CI) m/z calcd for $C_{15}H_{28}ClO_4^+$: 307.1671 $[M+H]^+$; found: 307.1682.

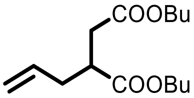

Dibutyl 2-allylsuccinate (40). Prepared according to GP5. Purified by flash column chromatography on silica gel (Pentane:Ethyl Acetate 95:5) to afford the product as colorless oil (68 mg, 50%). ^{1}H NMR (300 MHz, $CDCl_3$) δ 5.78 – 5.61 (m, 1H), 5.12 – 5.00 (m, 2H), 4.13 – 3.99 (m, 4H), 2.96 – 2.84 (m, 1H), 2.67 (dd, J_1 = 17 Hz, J_2 = 9 Hz, 1H), 2.47 – 2.35 (m, 2H), 2.32 – 2.20 (m, 1H), 1.64 – 1.52 (m, 4H), 1.42 – 1.28 (m, 4H), 0.90 (td, J_1 = 7 Hz, J_2 = 2 Hz, 6H). ^{13}C NMR (75 MHz, $CDCl_3$) δ 174.3, 172.1, 134.6, 117.8, 64.7, 64.6, 41.0, 36.1, 35.3, 30.7, 30.7, 19.2, 19.2, 13.8 (2C). HRMS (FI) m/z calcd for $C_{15}H_{26}O_4$: 270.1831; found: 270.1831.

10. Computational Details

All the calculations were carried out using the Gaussian 16 program package, revision C.01 installed on the HPC infrastructure Galileo100 at CINECA facility (Italy).[42] In our investigation, the level of theory chosen for the optimization of the reported stationary points was DFT (Density Functional Theory) by using the ωB97xD functional and the def2TZVP basis set in the gas phase. When appropriate, an unrestricted formalism (U prefix) has been adopted via the UωB97xD/def2TZVP keyword. No symmetry constraint was applied to the structures investigated and a thorough conformers search has been performed to locate the absolute minimum for each species. The structures and data used for this work correspond to those of the absolute minimum. Frequency calculations were performed in the gas phase to check that minima and transition states (TS) had 0 or 1 imaginary frequencies, respectively.

Solvent effect was included by single-point calculations at the same level of theory (ωB97xD/def2TZVP) adopting the standard implicit solvent model implemented in Gaussian 16 via the keyword SCRF=(SOLVENT=ACETONITRILE) on the optimized geometries obtained in vacuo.

The DFT Gibbs free energies reported in the text (see Figure 5E and Table S12Table S13) have been calculated by means of Eq. S1 reported below:

$$G_{DFT} = E_{0(DFT,MeCN)} + \Delta G_{CORR(vacuo)} \qquad (S1)$$

Where:

- $E_{0(DFT,MeCN)}$ is the total electronic energy calculated at the SCRF-ωB97xD/def2TZVP level (acetonitrile bulk);
- $\Delta G_{CORR(vacuo)}$ is the unscaled thermal correction to Gibbs Free Energy as from the output of the frequency calculation in vacuo, also including the zero-point vibrational energy (ZPVE).

The two terms from Eq. S1 have been reported in blue color for all the stationary points reported below.

As for TSs, Intrinsic Reaction Coordinate (IRC) calculations were performed in both directions (20 steps each) at the same level of theory adopted for optimizations (ωB97xD/def2TZVP in the gas phase) in order to investigate the process in detail and to confirm the nature of the TS itself. When indicated, the "LQA" option for the IRC keyword has been specified, in order to adopt the local quadratic approximation for the predictor step.

When structures not corresponding to stationary points have been considered (e.g. when stretching a bond or along IRC) energies have been expressed simply by considering the first term of Eq. S1, that is $E_{0(DFT,MeOH)}$, since frequency calculations (needed for determining $\Delta G_{CORR(vacuo)}$) were carried out exclusively on stationary points.

Optimized geometry listed in cartesian format (coordinates are given in Å), minimum energies and thermochemical data (in Hartree; the default options were adopted in the latter case, *viz.* temperature: 298.150 K and pressure: 1.00000 atm) are reported below.

The conversion factor adopted between Hartree and kcal mol^{-1} is: 1 Hartree = 627.509 kcal mol^{-1}.

Reaction profile reported in Figure 5E

SPECIES	G_{DFT} [Hartree]	SPECIES	G_{DFT} [Hartree]
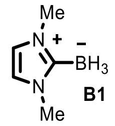 B1	-331.398913	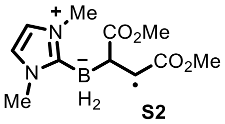 S2	-865.071356
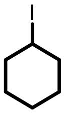 1a	-532.956177	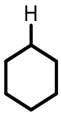 S3	-235.751370
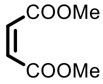 2b	-534.287989	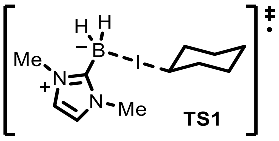 TS1	-863.706597
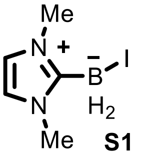 S1	-628.647677	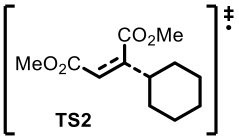 TS2	-769.361443
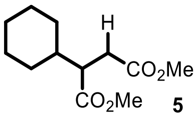 5	-770.062835	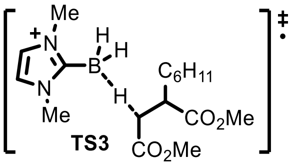 TS3	-1100.792971
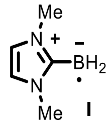 I	-330.768912	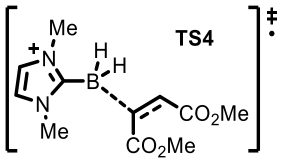 TS4	-865.037139
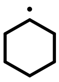 II	-235.098148	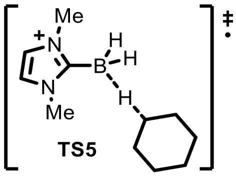 TS5	-566.469443
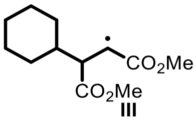 III	-769.413862		

Table S12: Gibbs free energies (shown in Hartree) of the species optimized to model the reaction profile gathered in Figure 5E, as determined from ωB97xD/def2TZVP calculations in bulk acetonitrile.

Comparison between different halogen abstractors shown in Figure 5E

SPECIES	G_{DFT} [Hartree]	SPECIES	G_{DFT} [Hartree]
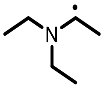 **S4**	-291.622791	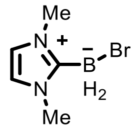 **S11**	-2905.080380
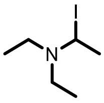 **S5**	-589.480638	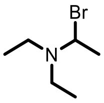 **S12**	-2865.909710
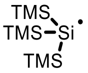 **S6**	-1517.069513	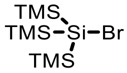 **S13**	-4091.389282
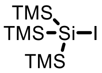 **S7**	-1814.956484	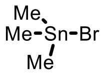 **S14**	-2908.332218
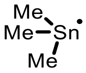 **S8**	-334.009945	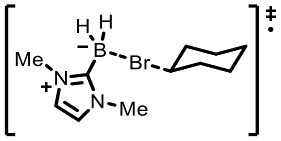 **TS8**	-3140.127994
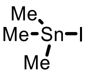 **S9**	-631.900065	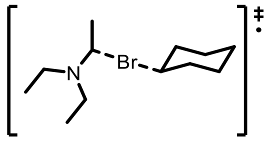 **TS9**	-3100.977702
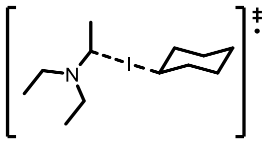 **TS6**	-824.555101	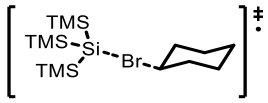 **TS10**	-4326.439008
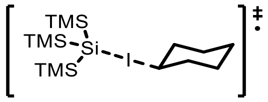 **TS7**	-2050.015998	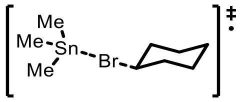 **TS11**	-3143.380054
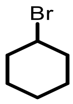 **S10**	-2809.387668		

Table S13: Gibbs free energies (shown in Hartree) of the species required to model selected examples of XAT steps between different halogen donors and halogen abstractors, as shown in Figure 5E. Values as determined from ωB97xD/def2TZVP calculations in bulk acetonitrile.

10.1 IRC Plots

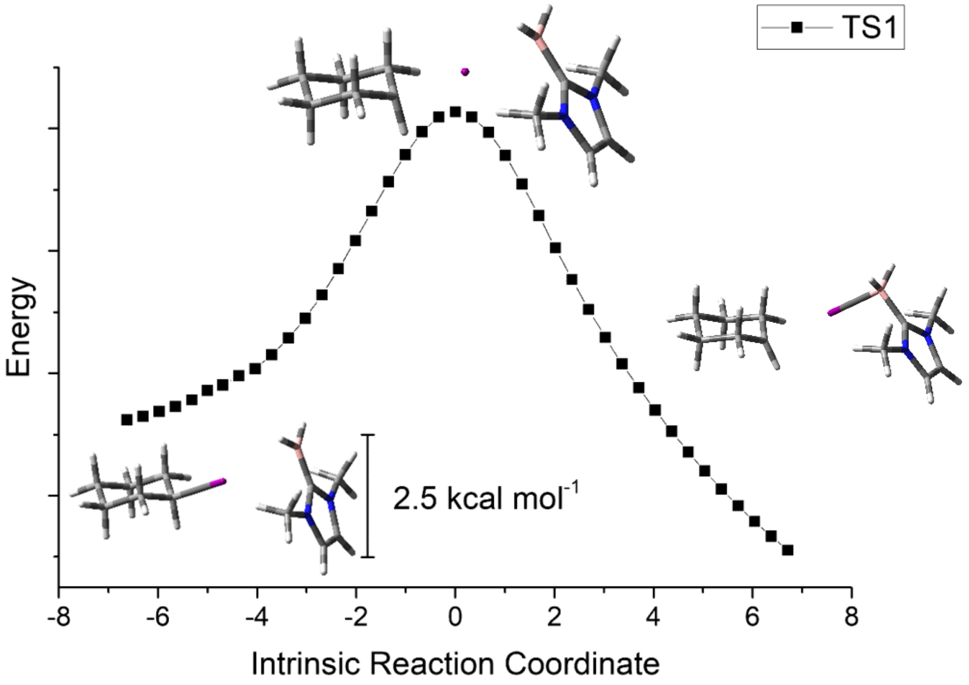

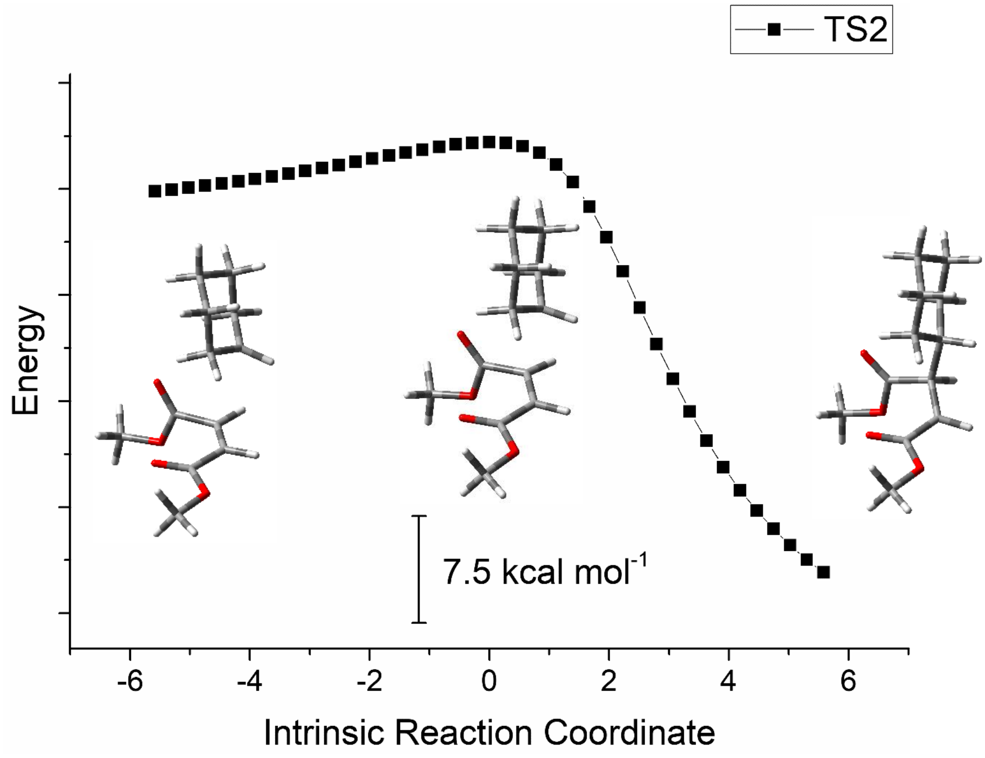

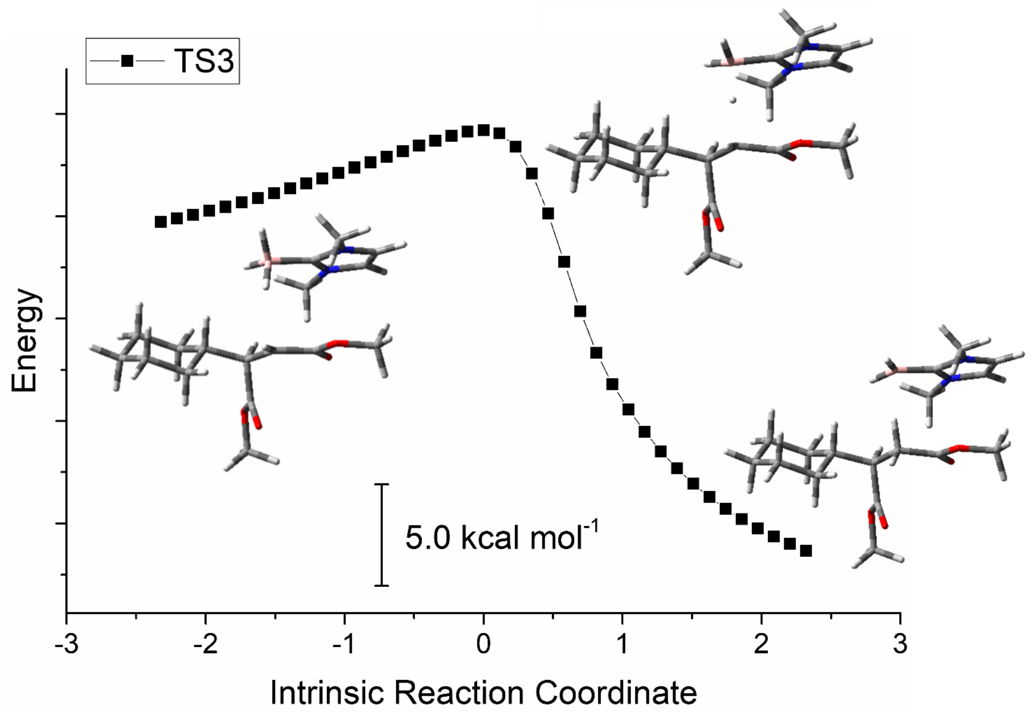

TS3
Energy
5.0 kcal mol-1
-3
-2
-1
0
1
2
3
Intrinsic Reaction Coordinate

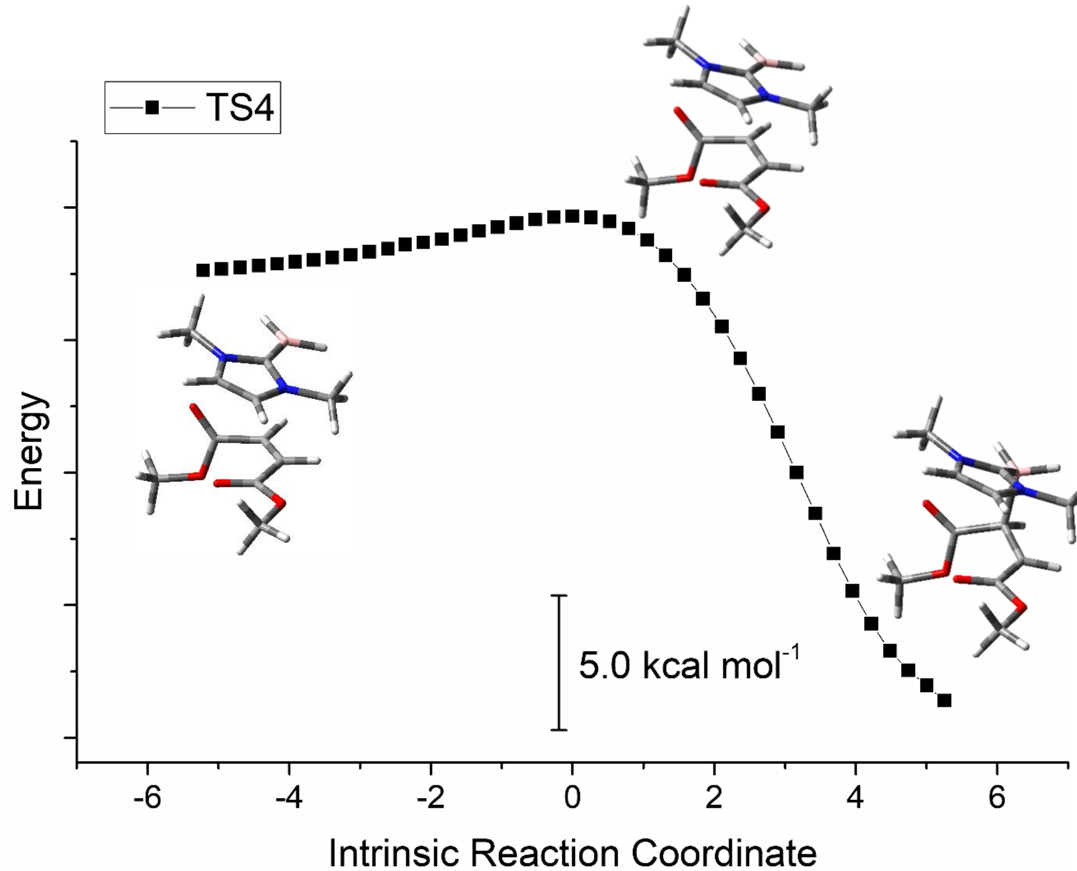

TS4
Energy
5.0 kcal mol-1
-6
-4
-2
0
2
4
6
Intrinsic Reaction Coordinate

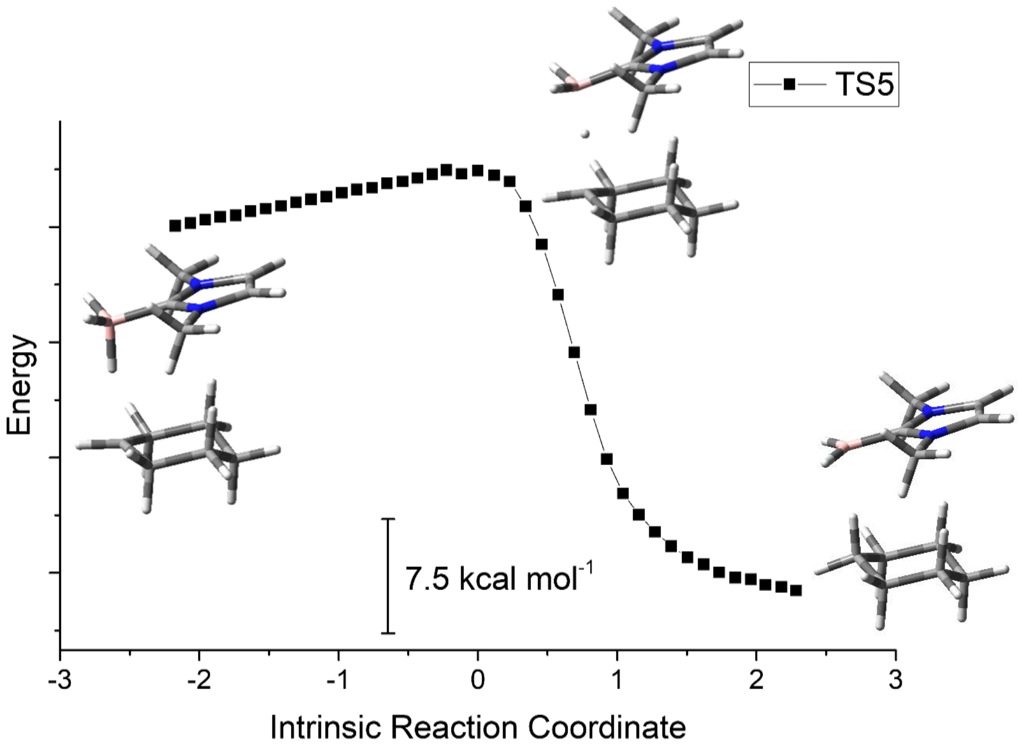

Figure S18: IRC plots of the transition states **TS1-5** describing the reaction profile reported in Figure 5E, as from calculations at the ωB97xD/def2TZVP level of theory in the gas phase (total electronic energy values have been reported). The three structures reported in each graph refer, respectively, to those of the first point (left), the transition state (center) and the last point (right) along the reaction coordinate. The LQA option has been adopted for **TS1,5**.

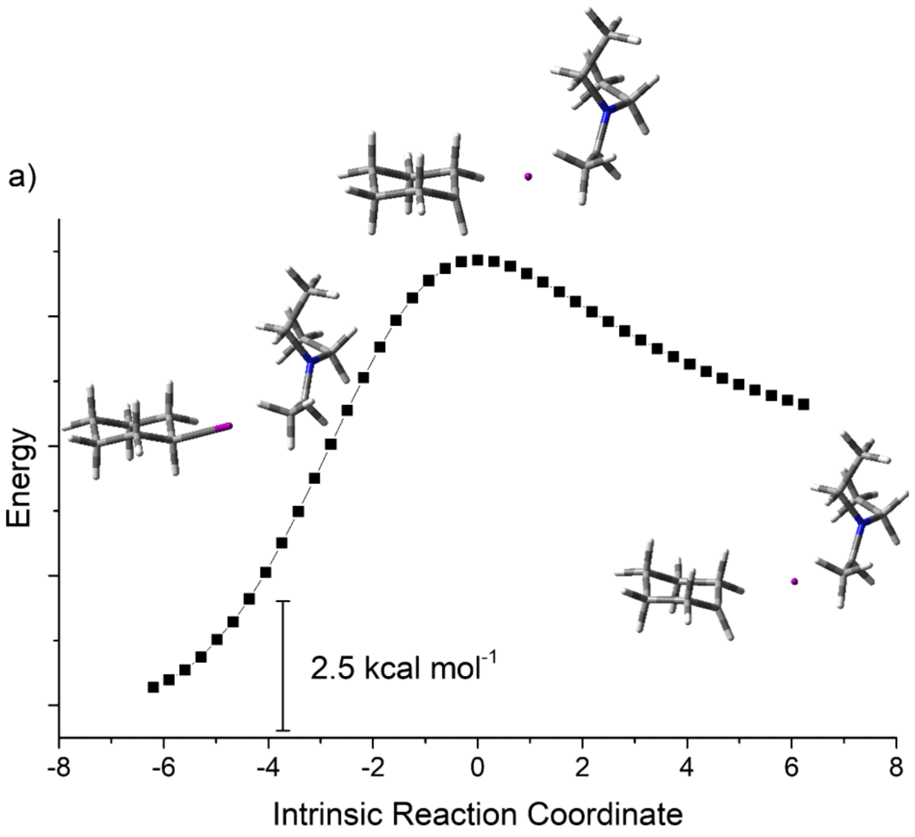
a)
Energy
2.5 kcal mol-1
-8
-6
-4
-2
0
2
4
6
8
Intrinsic Reaction Coordinate

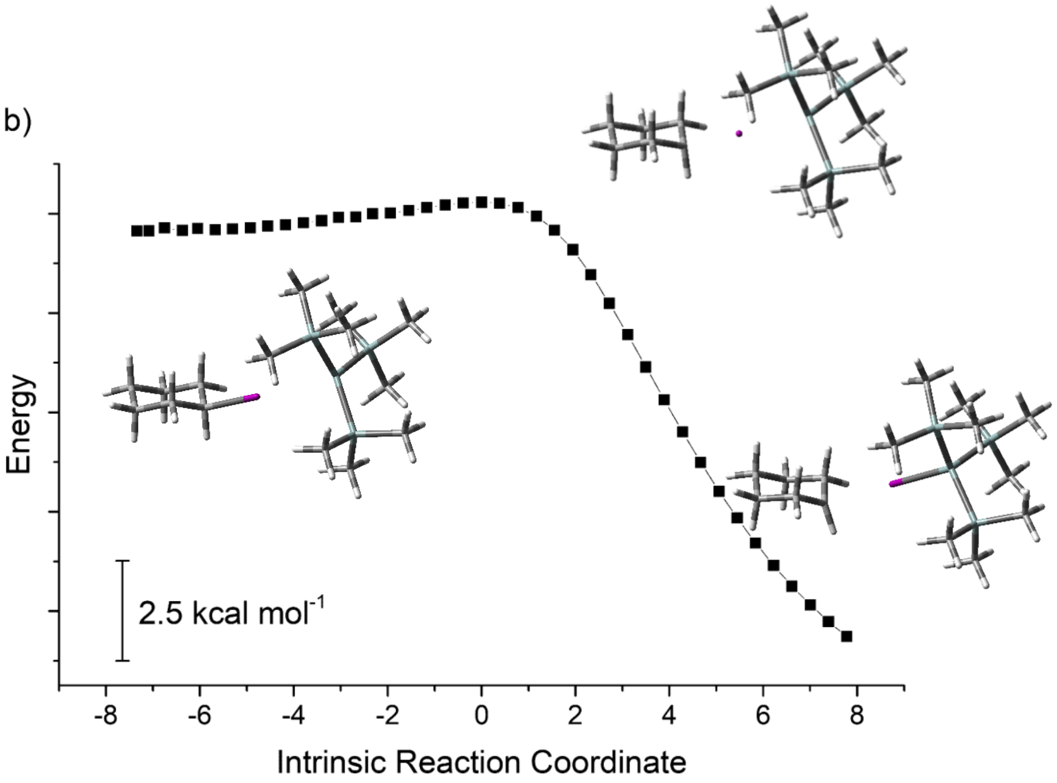
b)
Energy
2.5 kcal mol-1
-8
-6
-4
-2
0
2
4
6
8
Intrinsic Reaction Coordinate

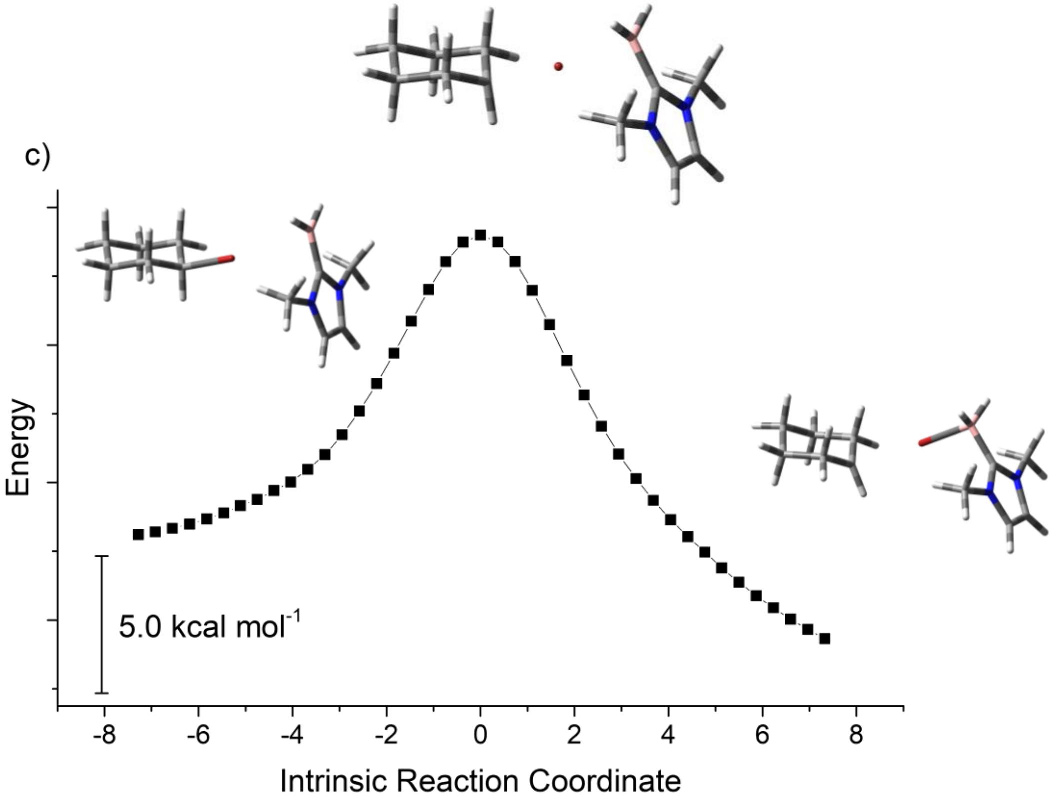
c)
Energy
5.0 kcal mol-1
-8
-6
-4
-2
0
2
4
6
8
Intrinsic Reaction Coordinate

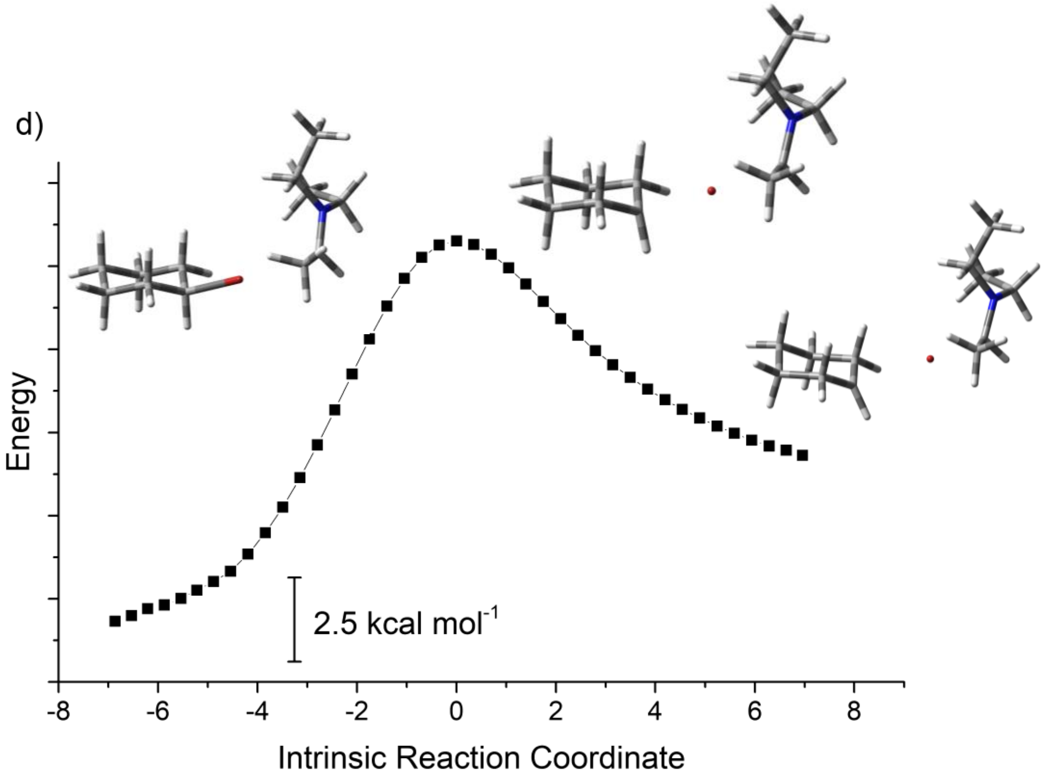
d)
Energy
2.5 kcal mol-1
-8
-6
-4
-2
0
2
4
6
8
Intrinsic Reaction Coordinate

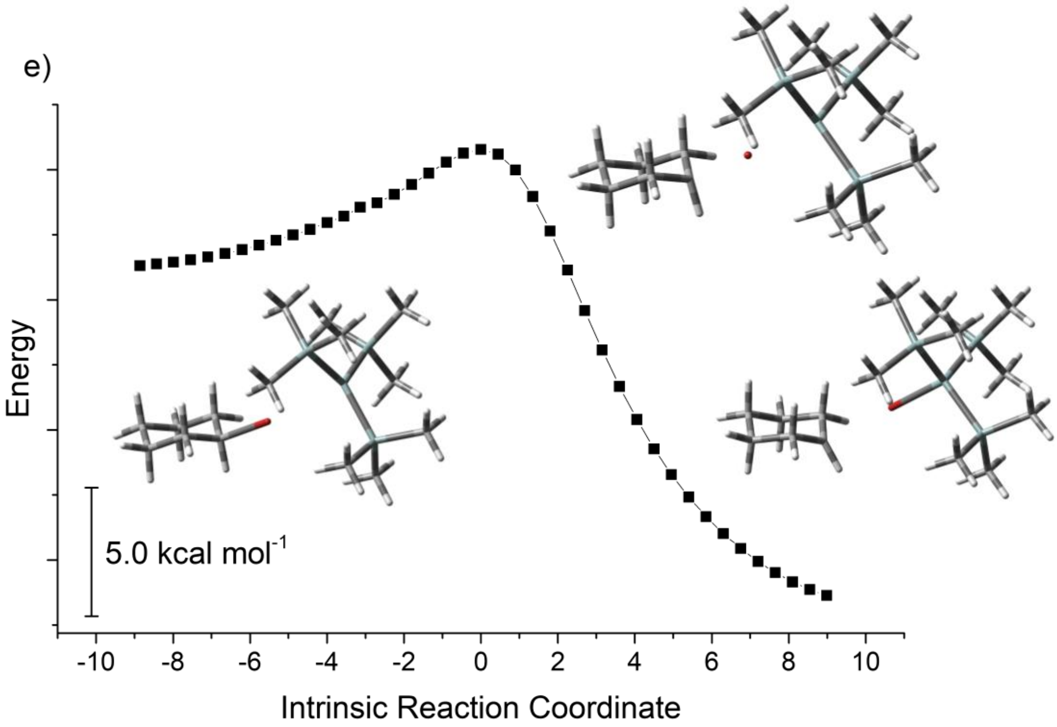

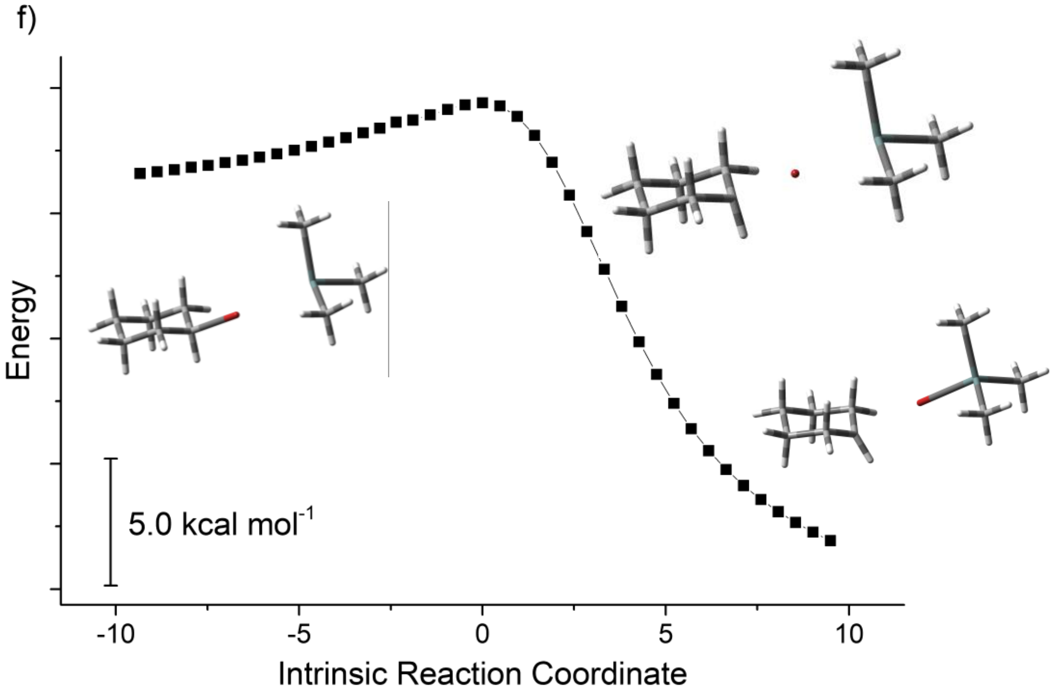

Figure S19: IRC plots of the transition states describing the XAT step between: a) α-amino radical **S4** and cyclohexyl iodide **1a**, b) silyl radical **S6** and cyclohexyl iodide **1a**; c) ligated boryl radical **I** and cyclohexyl bromide **S10**; d) α-amino radical **S4** and cyclohexyl bromide **S10**, e) silyl radical **S6** and cyclohexyl bromide **S10**; f) stannyl radical **S8** and cyclohexyl bromide **S10** (see Table in Figure 5E), as from calculations at the ωB97xD/def2TZVP level of theory in the gas phase (total electronic energy values have been reported). The three structures reported in each graph refer, respectively, to those of the first point (left), the transition state (center) and the last point (right) along the reaction coordinate. In all cases, the LQA option has been adopted.

10.2 Relaxed PES Scan

All the attempts to locate a transition state describing the XAT step from cyclohexyl iodide (**1a**) when using $Me_3Sn^•$ (**S8**) as the halogen abstractor failed. For this reason, we performed a relaxed potential energy surface (PES) scan by elongating the C–I bond in the model substrate **1a** in the presence of named abstractor. Thus, we adopted the "OPT = MODREDUNDANT" approach by freezing the C–I bond at the equilibrium length found in **1a** alone (2.16 Å) and then elongating it with a 0.1 Å step size for 15 steps. As apparent from Figure S20, the total electronic energy values follow a decreasing profile upon C–I elongation (black symbols, left axis). In the same graphic, the Sn–I bond length (not involved in any constraint) is also plotted, showing that this value decreases finally reaching a plateau (red symbols, right axis), which corresponds to the same Sn–I bond length found in the Me_3Sn–I species alone (2.72 Å).

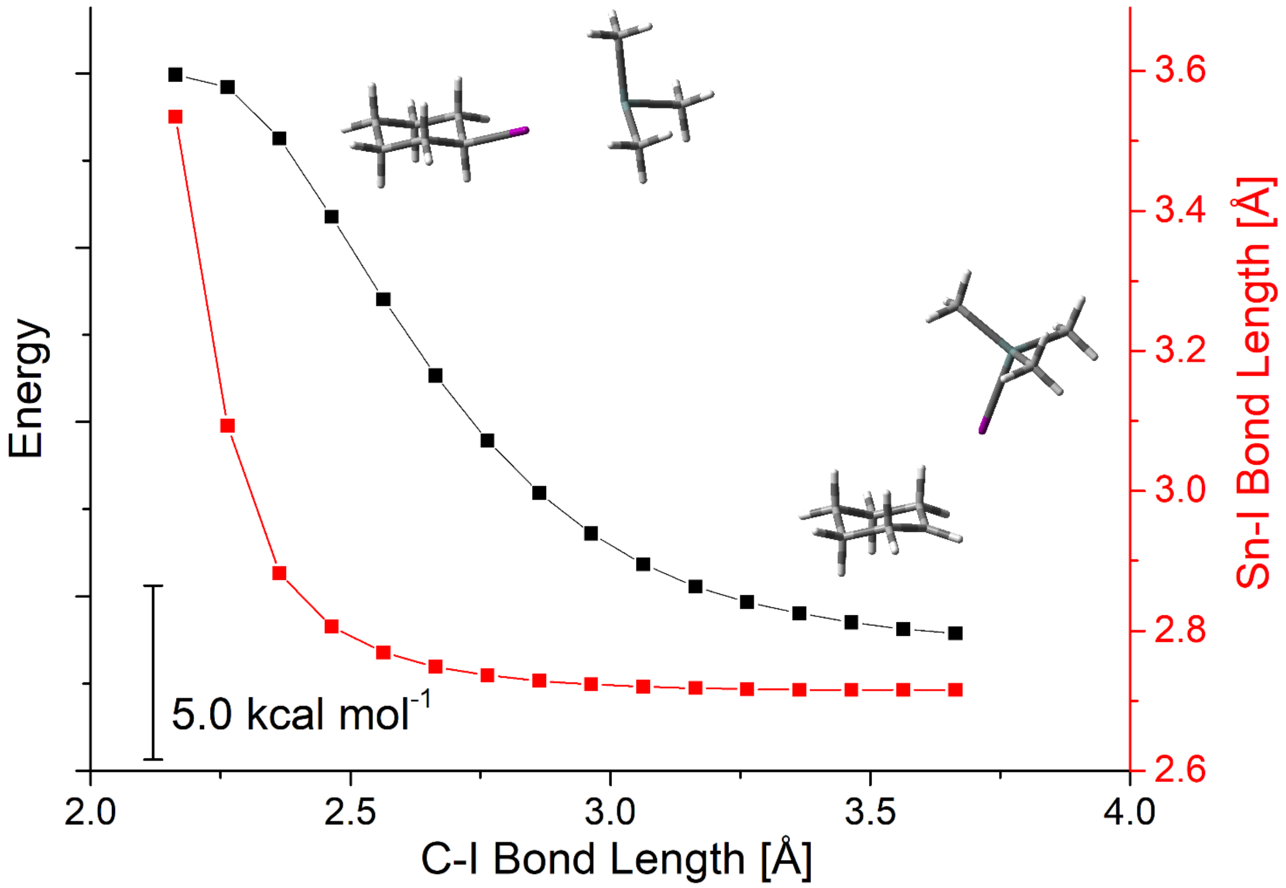

Figure S20: Relaxed PES scan describing the XAT step in cyclohexyl iodide (**1a**) promoted by the $Me_3Sn^•$ abstractor (**S8**, see Table in Figure 5E), as from calculations at the ωB97xD/def2TZVP level of theory in the gas phase (total electronic energy values have been reported). The two structures reported in the graph refer, respectively, to those of the first (left) and last (right) points of the scan.

10.3 Optimized Structures

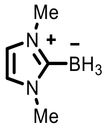

B1

C	0.77146100	-1.51291600	0.00045100
C	-0.57300700	-1.58835500	-0.00069400
C	-0.02002600	0.58658100	0.00016700
N	1.09372500	-0.17191800	0.00108600
H	1.52093100	-2.28389900	0.00055100
H	-1.23024800	-2.43936000	-0.00143300
N	-1.04024700	-0.29470000	-0.00124600
B	-0.20596900	2.16894000	-0.00018800
H	-0.87338000	2.44226200	0.98584500
H	0.86229500	2.74239200	0.01366300
H	-0.84723000	2.44391000	-1.00337800
C	2.45286900	0.32989200	-0.00006500
H	2.41369700	1.41469500	0.00879700
H	2.98040700	-0.02503200	0.88524200
H	2.97402200	-0.01029400	-0.89492900
C	-2.43232600	0.10375900	0.00087500
H	-3.05355400	-0.78845400	-0.03763100
H	-2.65203800	0.67404700	0.90217100
H	-2.63323000	0.73759700	-0.86124400

E(UwB97XD, vacuo)	-331.510016
Zero-point correction=	0.160452
Thermal correction to Energy=	0.169544
Thermal correction to Enthalpy=	0.170489
Thermal correction to Gibbs Free Energy=	**0.125942**
E(UwB97XD, MeCN)	**-331.524855**

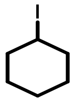

1a

C	2.59021200	-1.25759000	0.18929600
C	1.09405300	-1.26191200	-0.13397300
C	0.43757500	0.00000000	0.40512400
C	1.09405300	1.26191200	-0.13397400
C	2.59021100	1.25759100	0.18929800
C	3.27269100	0.00000000	-0.33980000
H	0.95464900	-1.30377800	-1.21858300
H	0.61626500	-2.14827200	0.28534500
H	2.72534600	-1.31395200	1.27489200
H	3.05450500	-2.15206200	-0.23105100
H	0.95465200	1.30377400	-1.21858500
H	0.61626200	2.14827300	0.28533900
H	3.05450400	2.15206400	-0.23104600
H	2.72534300	1.31395000	1.27489400

H	3.22926500	0.00000100	-1.43435400
H	4.32988700	-0.00000100	-0.06638500
H	0.46873400	0.00000100	1.49503800
I	-1.68306000	0.00000000	-0.02398700

E(UwB97XD, vacuo)	-533.081999
Zero-point correction=	0.161571
Thermal correction to Energy=	0.168598
Thermal correction to Enthalpy=	0.169542
Thermal correction to Gibbs Free Energy=	**0.128715**
E(UwB97XD, MeCN)	**-533.084892**

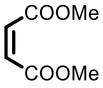

2b

C	0.66278500	-1.55002100	-0.30836200
C	-0.65369300	-1.41160300	-0.36207400
H	1.11638600	-2.50056500	-0.56478400
H	-1.28276300	-2.23309300	-0.67778000
C	1.62452100	-0.50736700	0.16763500
O	2.14315300	-0.55055400	1.24765300
O	1.88150900	0.39836800	-0.77005700
C	-1.33894600	-0.15341600	0.01739200
O	-0.80661100	0.82041200	0.48041900
O	-2.65268300	-0.24264200	-0.21663700
C	-3.41973100	0.91071300	0.11902800
H	-3.32328400	1.13775100	1.18062200
H	-3.08685000	1.77251500	-0.45928300
H	-4.44887400	0.66298200	-0.12567800
C	2.73714400	1.46877600	-0.37185000
H	2.27556000	2.02735600	0.44193400
H	3.70490600	1.08800900	-0.04629500
H	2.84949800	2.09788000	-1.25036900

E(UwB97XD, vacuo)	-534.379468
Zero-point correction=	0.139721
Thermal correction to Energy=	0.150563
Thermal correction to Enthalpy=	0.151507
Thermal correction to Gibbs Free Energy=	**0.101849**
E(UwB97XD, MeCN)	**-534.389838**

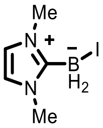

S1

C	2.88802900	-0.67473800	-0.67385100
C	2.88795500	0.67473200	-0.67395900
C	1.14896400	0.00000200	0.55017400

N	1.81553600	-1.07089300	0.08802800
H	3.55138800	-1.37776000	-1.14430700
H	3.55125900	1.37775000	-1.14449900
N	1.81554400	1.07089300	0.08803200
B	-0.18660100	0.00000600	1.40843800
H	-0.30841600	-1.01183400	2.04384100
H	-0.30841800	1.01185200	2.04383100
C	1.39349200	-2.44343100	0.30183700
H	1.43794700	-2.68904100	1.36065100
H	0.36953200	-2.56497400	-0.04904500
H	2.05563200	-3.10035600	-0.25711000
C	1.39351600	2.44343200	0.30186100
H	2.05561600	3.10035300	-0.25713800
H	0.36953400	2.56496300	-0.04896000
H	1.43803300	2.68905800	1.36066900
I	-1.82959200	0.00000000	-0.20790400

E(UwB97XD, vacuo) -628.747854
Zero-point correction= 0.154203
Thermal correction to Energy= 0.164246
Thermal correction to Enthalpy= 0.165190
Thermal correction to Gibbs Free Energy= **0.116429**

E(UwB97XD, MeCN) **-628.764106**

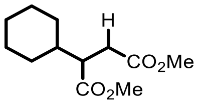

5

C	-1.20126400	-1.07618700	0.00229900
C	-0.16232300	-0.06758100	-0.46796500
H	-1.11649300	-1.25267700	1.07546400
C	-2.61446700	-0.62694100	-0.26685000
O	-2.92985700	0.41528800	-0.77506300
O	-3.49626300	-1.54726000	0.13767300
C	-0.27310400	1.19638400	0.36199900
O	-0.52167200	1.22348900	1.53914600
O	-0.03526600	2.29200600	-0.36222000
C	-0.11337800	3.52835800	0.33982400
H	-1.11058900	3.66521500	0.75732500
H	0.61724600	3.55959300	1.14848100
H	0.09976400	4.29992000	-0.39510700
C	-4.86972300	-1.21707200	-0.05006300
H	-5.12442100	-0.31226600	0.50156600
H	-5.08652200	-1.06033700	-1.10673700
H	-5.43261400	-2.06484400	0.33112900
C	1.26808200	-0.65459000	-0.43615200
C	2.29367300	0.28090700	-1.08076700
C	1.74281400	-1.08115200	0.95542700
H	1.22147900	-1.55853600	-1.05791500
C	3.67590100	-0.36171700	-1.14560300
H	2.35787900	1.20611500	-0.49921300
H	1.95787500	0.56841300	-2.08019500
C	3.12935200	-1.71863300	0.89936900

H	1.76805100	-0.20674400	1.61319600
H	1.03695300	-1.78301100	1.40380800
C	4.14811000	-0.79559500	0.23859300
H	4.39000600	0.33470600	-1.59062000
H	3.63806800	-1.23646900	-1.80460400
H	3.45517700	-1.98440000	1.90736800
H	3.07234800	-2.65440800	0.33166900
H	5.12023600	-1.28926100	0.17169800
H	4.28741800	0.09264300	0.86481400
H	-0.37963000	0.21116600	-1.50005600
H	-1.04980900	-2.04008900	-0.48903400

E(UwB97XD, vacuo)	-770.322039
Zero-point correction=	0.315646
Thermal correction to Energy=	0.332740
Thermal correction to Enthalpy=	0.333684
Thermal correction to Gibbs Free Energy=	**0.268986**
E(UwB97XD, MeCN)	**-770.331821**

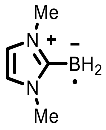

I

C	0.67454600	-1.50605400	-0.00002000
C	-0.67456500	-1.50603300	0.00007100
C	0.00002400	0.65105800	-0.00009200
N	1.08697100	-0.19663200	-0.00014000
H	1.36908300	-2.32721100	0.00003800
H	-1.36911100	-2.32718500	0.00002400
N	-1.08696700	-0.19660900	-0.00004900
B	0.00000400	2.14939900	0.00001800
H	1.04312000	2.73736000	-0.00002400
H	-1.04317200	2.73726300	0.00007200
C	2.45297000	0.25824700	0.00008800
H	2.65096700	0.86672200	-0.88357900
H	2.65098300	0.86595700	0.88428500
H	3.11395600	-0.60634300	-0.00030400
C	-2.45296800	0.25824900	0.00004200
H	-3.11393500	-0.60635500	-0.00045300
H	-2.65109500	0.86591000	0.88425000
H	-2.65088400	0.86676700	-0.88361200

E(UwB97XD, vacuo)	-330.877078
Zero-point correction=	0.150417
Thermal correction to Energy=	0.158964
Thermal correction to Enthalpy=	0.159908
Thermal correction to Gibbs Free Energy=	**0.117135**
E(UwB97XD, MeCN)	**-330.886047**

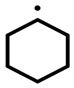

II

C	-1.25912300	0.70835400	-0.24284900
C	-1.28175500	-0.77199900	0.15670500
C	-0.00000100	-1.45638700	-0.16566900
C	1.28175400	-0.77200000	0.15670500
C	1.25912400	0.70835300	-0.24285000
C	0.00000100	1.40149400	0.27025000
H	-1.46240700	-0.82685200	1.24414500
H	-2.12427500	-1.28343700	-0.31342900
H	-1.28871600	0.78497000	-1.33440800
H	-2.15270900	1.20945200	0.13619000
H	1.46240500	-0.82685200	1.24414600
H	2.12427400	-1.28344000	-0.31342700
H	2.15271000	1.20945000	0.13618700
H	1.28871500	0.78496800	-1.33440800
H	0.00000100	1.38781000	1.36655100
H	0.00000100	2.45171700	-0.03055900
H	-0.00000100	-2.51467200	-0.39473900

E(UwB97XD, vacuo)	-235.224466
Zero-point correction=	0.156606
Thermal correction to Energy=	0.162638
Thermal correction to Enthalpy=	0.163582
Thermal correction to Gibbs Free Energy=	**0.126870**
E(UwB97XD, MeCN)	**-235.225018**

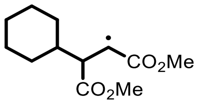

III

C	1.19957000	-1.02406400	0.24052000
C	0.14300700	-0.02961300	0.56026900
H	0.94904800	-2.04888200	0.00328500
C	2.59379900	-0.64162800	0.28016000
O	2.99114600	0.46340500	0.57962700
O	3.40919400	-1.65883100	-0.04788900
C	0.28873000	1.20445600	-0.32435200
O	0.49548600	1.17822600	-1.50694600
O	0.11638500	2.32809800	0.37399300
C	0.23639000	3.53897100	-0.36705200
H	1.23003300	3.61434900	-0.80775000
H	-0.50978100	3.58027800	-1.16078900
H	0.07566700	4.34058200	0.34867600
C	4.79874100	-1.35523100	-0.04527300
H	5.01879300	-0.55719500	-0.75485800
H	5.12349900	-1.04238500	0.94760800
H	5.30350000	-2.27227900	-0.33752800
C	-1.27696700	-0.63215700	0.48095500
C	-2.33730400	0.31349700	1.04763300
C	-1.67653300	-1.09604200	-0.92158500

H	-1.25086100	-1.51818900	1.12871800
C	-3.71647600	-0.33961500	1.05957800
H	-2.37558200	1.22222600	0.43749800
H	-2.05640800	0.62948900	2.05551700
C	-3.05216900	-1.75768200	-0.91103800
H	-1.69052000	-0.23561500	-1.59645300
H	-0.93142100	-1.78523100	-1.32556200
C	-4.11311100	-0.82711300	-0.33084300
H	-4.45858200	0.36452600	1.44201000
H	-3.70583000	-1.19019700	1.75032300
H	-3.32582900	-2.05949600	-1.92423300
H	-3.00974600	-2.67393300	-0.31115800
H	-5.08136800	-1.33123400	-0.29361300
H	-4.23238400	0.03701000	-0.99371700
H	0.30401800	0.31631200	1.58791900

E(UwB97XD, vacuo)	-769.658463
Zero-point correction=	0.301940
Thermal correction to Energy=	0.318989
Thermal correction to Enthalpy=	0.319933
Thermal correction to Gibbs Free Energy=	**0.254921**
E(UwB97XD, MeCN)	**-769.668783**

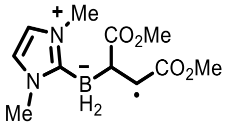

S2

C	1.37510700	-0.48369800	1.31152600
C	0.72528800	0.82451900	1.43619500
H	1.66959400	-1.02363000	2.20130400
C	1.51961000	-1.15557300	0.05051700
O	1.11659200	-0.74522000	-1.02152000
O	2.14773200	-2.35140700	0.17458800
C	1.09015600	1.77648900	0.31898200
O	0.34735700	2.51356800	-0.27578300
O	2.41301900	1.77022200	0.08727500
C	2.85813300	2.58646500	-0.98656900
H	2.39035600	2.27142300	-1.91977000
H	2.61911400	3.63469900	-0.80469400
H	3.93544300	2.44829400	-1.03828600
C	2.34230500	-3.06467700	-1.03547400
H	1.38773300	-3.29900100	-1.51100700
H	2.94235800	-2.48474100	-1.73776300
H	2.86117500	-3.98066100	-0.76316900
H	1.09287100	1.29819000	2.35229700
B	-0.91680500	0.67108800	1.75323400
H	-1.04735600	-0.06636300	2.71368200
H	-1.36377500	1.76694100	2.00847900
C	-1.75660700	0.04988500	0.52919300
C	-3.10938900	-0.13715400	-1.24094300
C	-2.74135100	-1.38208700	-0.88319400
H	-3.74292900	0.20767200	-2.03798600

H	-2.98297300	-2.33999400	-1.30721100
C	-1.34203300	-2.37779800	0.91021200
H	-0.56105600	-2.83921400	0.30714400
H	-0.91778000	-2.02772000	1.84608300
H	-2.12168900	-3.11017200	1.11808600
C	-2.71410300	2.16510600	-0.36423500
H	-1.76007600	2.66478700	-0.22962900
H	-3.14690600	2.44309600	-1.32314300
H	-3.39728800	2.43763400	0.43916100
N	-1.92089200	-1.24965700	0.21073900
N	-2.50098000	0.72639400	-0.36409300

E(UwB97XD, vacuo)	-865.299658
Zero-point correction=	0.294393
Thermal correction to Energy=	0.313797
Thermal correction to Enthalpy=	0.314742
Thermal correction to Gibbs Free Energy=	**0.244927**
E(UwB97XD, MeCN)	**-865.316283**

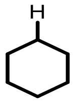

S3

C	1.37647000	-0.47626300	-0.22954200
C	1.10069200	0.95382600	0.22958400
C	-0.27572200	1.43007600	-0.22963100
C	-1.37645100	0.47628300	0.22958800
C	-1.10071600	-0.95381100	-0.22954800
C	0.27572100	-1.43008500	0.22953700
H	1.14664800	0.99375300	1.32390400
H	1.87722300	1.62693700	-0.14130800
H	1.43414000	-0.49615900	-1.32385400
H	2.34759100	-0.81230000	0.14147000
H	-1.43398300	0.49621500	1.32390800
H	-2.34759400	0.81234000	-0.14133200
H	-1.87723800	-1.62690100	0.14141800
H	-1.14680200	-0.99374100	-1.32386000
H	0.28729600	-1.48994200	1.32383900
H	0.47029400	-2.43911700	-0.14146000
H	-0.47029500	2.43911000	0.14127000
H	-0.28724400	1.48964900	-1.32392600

E(UwB97XD, vacuo)	-235.893931
Zero-point correction=	0.171535
Thermal correction to Energy=	0.177179
Thermal correction to Enthalpy=	0.178123
Thermal correction to Gibbs Free Energy=	**0.142822**
E(UwB97XD, MeCN)	**-235.894192**

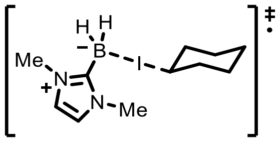

TS1

C	-4.56579800	1.27622200	0.61255100
C	-3.11717100	1.26618800	0.10359400
C	-2.42840500	-0.00872800	0.51693700
C	-3.14666500	-1.25351400	0.06389000
C	-4.59532200	-1.24573700	0.57269800
C	-5.32042000	0.03082300	0.15385200
H	-3.12481300	1.33704500	-0.98948200
H	-2.57638400	2.13827600	0.47569800
H	-4.56278200	1.31377300	1.70745500
H	-5.07444300	2.17981200	0.26668000
H	-3.15545800	-1.28969400	-1.03086900
H	-2.62654400	-2.14929500	0.40803200
H	-5.12480200	-2.12577900	0.19854400
H	-4.59372000	-1.31795400	1.66586300
H	-5.41060000	0.04916900	-0.93806300
H	-6.33741800	0.03645200	0.55349600
H	-2.16782500	-0.02838500	1.57472200
I	-0.17737000	-0.02109800	-0.44297500
C	4.04250700	0.71827700	1.40889300
C	4.05214000	-0.63049400	1.43849900
C	2.98680500	-0.00442800	-0.42983000
N	3.39719100	1.09030200	0.25512700
H	4.44168900	1.43692500	2.10171800
H	4.46139400	-1.31235400	2.16195900
N	3.41263200	-1.06202200	0.30235300
B	2.12772400	-0.03835100	-1.71399600
H	2.05549700	0.98085500	-2.33538900
H	2.06647500	-1.08535700	-2.28856700
C	3.08467400	2.44769900	-0.13674800
H	3.48747100	2.65477600	-1.12663500
H	2.00279500	2.58149300	-0.16147100
H	3.52400200	3.13028000	0.58721000
C	3.11991200	-2.43967700	-0.02955200
H	3.57330200	-3.08373200	0.72057700
H	2.04025100	-2.59169300	-0.04315300
H	3.52158600	-2.68240100	-1.01175400

E(UwB97XD, vacuo)	-863.955455
Zero-point correction=	0.312062
Thermal correction to Energy=	0.329128
Thermal correction to Enthalpy=	0.330072
Thermal correction to Gibbs Free Energy=	**0.262280**

E(UwB97XD, MeCN)	**-863.968877**

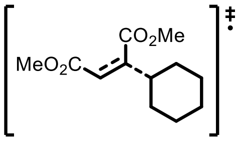

TS2

C	1.28137600	-0.48119900	1.31515800
C	0.49768400	0.61414800	1.21185300
H	1.26029400	-1.08828300	2.20966500
C	2.11612900	-0.94707100	0.20787500
O	2.22143000	-0.41156200	-0.86977900
O	2.77195200	-2.07401900	0.53341900
C	0.63323000	1.60638200	0.08661200
O	-0.13788700	1.77747700	-0.81547600
O	1.72400900	2.34904200	0.28188200
C	2.01818600	3.28902700	-0.74876500
H	2.17466500	2.76697100	-1.69241400
H	1.20439200	4.00504000	-0.86319300
H	2.92843900	3.79333700	-0.43600300
C	3.61643300	-2.60761900	-0.47907900
H	3.04016100	-2.85377100	-1.37132100
H	4.39355200	-1.89227800	-0.74939800
H	4.05902300	-3.50481500	-0.05420100
C	-1.66458200	-0.34962300	0.83853500
C	-2.68936200	0.73510500	0.79763600
C	-1.50633300	-1.14701300	-0.41167400
H	-1.59051500	-0.90301900	1.77153900
C	-4.05048000	0.13168900	0.39735300
H	-2.40244800	1.47468900	0.04632200
H	-2.77086000	1.24549900	1.75990200
C	-2.86782500	-1.74290700	-0.81417700
H	-1.15437800	-0.49262300	-1.21361100
H	-0.76647800	-1.93910000	-0.28139500
C	-3.93480600	-0.65426700	-0.90661900
H	-4.79009200	0.92991900	0.30006400
H	-4.40177700	-0.53395100	1.19292700
H	-2.77129400	-2.26630100	-1.76818200
H	-3.17358400	-2.48744500	-0.07125600
H	-4.90037800	-1.09621500	-1.16362400
H	-3.67455100	0.03421600	-1.71751700
H	0.00190600	1.00073000	2.09306800

E(UwB97XD, vacuo)	-769.602704
Zero-point correction=	0.298499
Thermal correction to Energy=	0.315794
Thermal correction to Enthalpy=	0.316738
Thermal correction to Gibbs Free Energy=	**0.250769**

E(UwB97XD, MeCN)	**-769.612212**

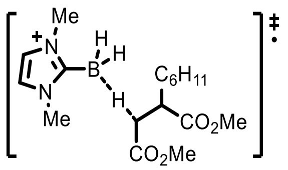

TS3

C	-0.03316700	0.21614700	-1.00436300
C	-1.12292900	0.33746600	0.02049600
H	-0.29463000	-0.10023500	-2.00535800
C	1.09811700	1.10419000	-0.89529900
O	1.37892000	1.77612000	0.08016600
O	1.90092300	1.05294200	-1.98429700
C	-1.74470300	1.72182100	-0.01707000
O	-1.90053300	2.39098000	-1.00283000
O	-2.13499400	2.11899300	1.20296600
C	-2.75312000	3.39899300	1.26534500
H	-2.06750900	4.17146600	0.91709200
H	-3.65334100	3.42363200	0.65064300
H	-3.00400100	3.55743100	2.31113600
C	3.05282600	1.87853900	-1.93313400
H	2.77448100	2.92962400	-1.84837700
H	3.68378400	1.61656000	-1.08188400
H	3.58473400	1.70755600	-2.86671200
C	-2.18251300	-0.77700900	-0.13668500
C	-3.01542800	-0.97532900	1.13020500
C	-3.09778700	-0.59882200	-1.34971100
H	-1.60260000	-1.69446400	-0.28645400
C	-3.97072400	-2.15687400	0.98666200
H	-3.58812900	-0.06626300	1.34060400
H	-2.35034200	-1.13082700	1.98388600
C	-4.04701400	-1.78450700	-1.50153300
H	-3.68515700	0.31824900	-1.23305800
H	-2.50983400	-0.46836000	-2.26049300
C	-4.87105400	-2.00141700	-0.23565000
H	-4.57404900	-2.26280800	1.89141600
H	-3.38635300	-3.07832600	0.88797800
H	-4.70458100	-1.62820300	-2.35979300
H	-3.46407800	-2.68820000	-1.71128400
H	-5.51500900	-2.87710400	-0.34639300
H	-5.53231400	-1.14006500	-0.08747100
H	-0.67705400	0.22302600	1.01043600
C	4.08391200	-0.06390900	1.59491200
C	4.63892800	-0.52488700	0.45662200
C	2.57317000	-1.37908800	0.59727000
N	2.82258400	-0.60175000	1.67139800
H	4.47195300	0.59648600	2.34903900
H	5.61083700	-0.35611900	0.02905100
N	3.70015300	-1.32912800	-0.14492500
B	1.22982300	-2.08510100	0.19392800
H	1.38211500	-3.00225300	-0.57434800
H	0.48377000	-2.28847600	1.12071900
H	0.64870700	-1.09715000	-0.50931100
C	1.84204400	-0.22999400	2.67162400
H	1.31638500	0.66702200	2.34155700
H	1.13447700	-1.04489800	2.79471200

H	2.35117900	-0.04016700	3.61483600
C	3.84330800	-1.95016300	-1.44600200
H	4.86700300	-1.81057000	-1.78663700
H	3.61933700	-3.01234200	-1.37939800
H	3.15420800	-1.48578800	-2.15126700

E(UwB97XD, vacuo)	-1101.177270
Zero-point correction=	0.462319
Thermal correction to Energy=	0.488819
Thermal correction to Enthalpy=	0.489764
Thermal correction to Gibbs Free Energy=	**0.402464**
E(UwB97XD, MeCN)	**-1101.195435**

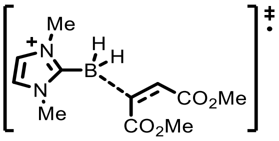

TS4

C	-0.47712800	1.12388900	1.35575200
C	-1.18030600	-0.01888800	1.51648600
H	0.03927500	1.56799200	2.19507900
C	-0.25390400	1.71743600	0.04827700
O	-0.65957100	1.29457400	-1.01139100
O	0.51916800	2.82783500	0.13243900
C	-2.11478900	-0.55312500	0.47442900
O	-2.04000000	-1.61458900	-0.08411700
O	-3.13247700	0.29345900	0.29682800
C	-4.05434400	-0.05943800	-0.72812300
H	-3.54261000	-0.10222300	-1.68975800
H	-4.51474700	-1.02575200	-0.52168200
H	-4.80520000	0.72637000	-0.73280400
C	0.78796500	3.47495300	-1.10200800
H	1.28128000	2.79537800	-1.79841400
H	-0.13449000	3.83119500	-1.56200100
H	1.43713600	4.31468700	-0.86495800
H	-1.32395400	-0.43318800	2.50553300
B	0.67038000	-1.88056600	1.76481200
H	0.98310700	-1.43759400	2.83127400
H	-0.00220800	-2.86277700	1.69443100
C	1.37826500	-1.37923500	0.51299000
C	1.95231200	-1.09285000	-1.63638300
C	2.53483600	-0.10221000	-0.93115400
H	1.96613900	-1.29833600	-2.69157500
H	3.15688300	0.71467500	-1.25001400
C	2.71903900	0.51042100	1.46807300
H	3.78265800	0.29824200	1.59498800
H	2.57657500	1.56974100	1.25612600
H	2.19702600	0.25631200	2.38427200
C	0.53276000	-3.07800400	-1.09563100
H	-0.49495900	-2.99455000	-0.75203800
H	0.55096200	-3.19342400	-2.17728300
H	1.00913900	-3.94161300	-0.63058800

N	2.17802500	-0.27896300	0.38402700
N	1.25638800	-1.87107100	-0.75273700

E(UwB97XD, vacuo)	-865.264533
Zero-point correction=	0.291706
Thermal correction to Energy=	0.311457
Thermal correction to Enthalpy=	0.312402
Thermal correction to Gibbs Free Energy=	**0.242138**
E(UwB97XD, MeCN)	**-865.279277**

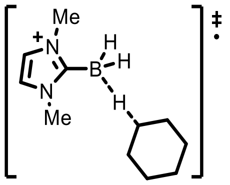

TS5

C	1.80436000	-1.25201000	0.90563200
C	2.41159100	-1.28090100	-0.49748800
C	2.19820800	0.00203400	-1.24313800
C	2.40786200	1.28457100	-0.49575600
C	1.80081600	1.25209200	0.90735600
C	2.22525400	0.00011400	1.66824200
H	3.49033300	-1.48358100	-0.40242900
H	1.99870300	-2.11509200	-1.07339300
H	0.71261700	-1.25359600	0.82882800
H	2.08799600	-2.15219300	1.45647000
H	3.48600400	1.49032600	-0.40052700
H	1.99244200	2.11826400	-1.07057600
H	2.08202200	2.15230500	1.45939400
H	0.70907100	1.25078300	0.83063000
H	3.31381500	0.00156700	1.79790700
H	1.78738400	-0.00120100	2.66958600
H	2.53952900	0.00331400	-2.27383100
C	-2.39424300	0.67186300	1.19642100
C	-2.39297400	-0.67500000	1.19623600
C	-1.46541500	-0.00042500	-0.73628500
N	-1.83030700	1.07227900	0.00488100
H	-2.74811500	1.37313700	1.93049000
H	-2.74555500	-1.37713800	1.93010100
N	-1.82824600	-1.07401500	0.00461800
B	-0.64186700	0.00017400	-2.05847000
H	-0.76484700	-1.02312500	-2.69190400
H	-0.76527100	1.02353600	-2.69168800
H	0.60152600	0.00020500	-1.59009700
C	-1.60673500	2.44146200	-0.40346500
H	-0.54026300	2.62198500	-0.53880700
H	-2.11077700	2.63678200	-1.34844400
H	-1.99462000	3.10460500	0.36693500
C	-1.60240100	-2.44265000	-0.40433700
H	-2.11054200	-2.64016400	-1.34668500
H	-0.53604200	-2.61961600	-0.54495700
H	-1.98414700	-3.10671700	0.36834100

E(UwB97XD, vacuo)	-566.730491
Zero-point correction=	0.316489
Thermal correction to Energy=	0.331911
Thermal correction to Enthalpy=	0.332856
Thermal correction to Gibbs Free Energy=	**0.272515**
E(UwB97XD, MeCN)	**-566.741958**

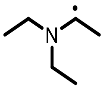

S4

C	-1.36195900	-0.73982500	0.15452900
N	-0.16458600	-0.07018700	0.37502100
C	0.98293700	-0.92148600	0.64232900
H	0.64396100	-1.71433000	1.31147500
H	1.72694500	-0.34797800	1.19825600
C	1.62030900	-1.53534900	-0.60082400
H	2.03909500	-0.76817400	-1.25544100
H	2.43029800	-2.21033800	-0.31920100
H	0.87866200	-2.09921800	-1.16875100
C	0.08990500	1.13505800	-0.39751100
H	0.27977000	0.89317100	-1.45483000
H	-0.81880600	1.73548600	-0.37698700
C	-2.61124600	0.00295100	-0.16737500
H	-2.57264900	0.50493700	-1.13909400
H	-3.44711500	-0.69565100	-0.20009700
H	-2.85667900	0.77225600	0.58113400
C	1.23465400	1.97462200	0.14564000
H	2.20321100	1.48977300	0.01551500
H	1.27189900	2.92578700	-0.38682600
H	1.09312100	2.18104100	1.20789200
H	-1.44721000	-1.67128200	0.70107700

E(UwB97XD, vacuo)	-291.780115
Zero-point correction=	0.193694
Thermal correction to Energy=	0.203037
Thermal correction to Enthalpy=	0.203981
Thermal correction to Gibbs Free Energy=	**0.159543**
E(UwB97XD, MeCN)	**-291.782334**

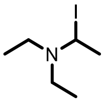

S5

C	0.23464800	0.82335100	0.86087700
N	1.42998800	0.29077300	0.47592900
C	1.84390500	-0.97981700	1.06422100
H	1.30391400	-1.08827800	2.00502000
H	2.90360500	-0.90476400	1.32074100

C	1.61504000	-2.21460300	0.19863900
H	2.20789100	-2.18317000	-0.71664100
H	1.91854300	-3.10119000	0.75714800
H	0.56635200	-2.32022000	-0.07487600
C	1.95738800	0.59901300	-0.84378700
H	1.51511200	-0.07975100	-1.58459000
H	1.63119000	1.60108500	-1.11116100
C	0.00039300	2.29807500	0.63645100
H	-0.08481900	2.56333300	-0.41465500
H	-0.92536500	2.59327400	1.12526800
H	0.83126200	2.85640200	1.07834600
C	3.47486400	0.54421300	-0.90858000
H	3.86051500	-0.46390800	-0.75360900
H	3.80752900	0.87427300	-1.89302200
H	3.91885100	1.20045700	-0.15839400
H	-0.02616600	0.50999000	1.86421600
I	-1.66407100	-0.19838400	-0.20419300

E(UwB97XD, vacuo)	-589.632635
Zero-point correction=	0.197625
Thermal correction to Energy=	0.208396
Thermal correction to Enthalpy=	0.209340
Thermal correction to Gibbs Free Energy=	**0.159888**
E(UwB97XD, MeCN)	**-589.640526**

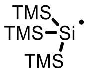

S6

Si	0.00022700	0.00109600	-0.69018000
Si	1.99994300	1.02841100	-0.05921200
Si	-1.89148600	1.21585900	-0.05880100
Si	-0.10833700	-2.24395500	-0.05866800
C	3.43448100	-0.16918700	-0.26581900
H	3.52668300	-0.50361500	-1.30110900
H	4.37608400	0.30646300	0.02061800
H	3.30294200	-1.05382000	0.36138700
C	2.31983800	2.57117500	-1.08209500
H	3.23494400	3.06897300	-0.75049500
H	2.43165600	2.32709800	-2.14024200
H	1.49634500	3.28219600	-0.98823800
C	1.88220200	1.52221300	1.75513100
H	2.81618000	1.98106800	2.09120500
H	1.07759200	2.24338900	1.91522900
H	1.68394700	0.65482000	2.38810600
C	1.06796500	-3.29225400	-1.08155300
H	1.04384800	-4.33307800	-0.74788600
H	0.79901800	-3.26942400	-2.13931800
H	2.09503900	-2.93249900	-0.99003500
C	0.38062600	-2.38559200	1.75498500
H	0.31430700	-3.42359900	2.09277500
H	1.40684800	-2.04621200	1.91303300
H	-0.27219300	-1.78058400	2.38784300

C	-1.86265500	-2.88895300	-0.26394800
H	-2.19710800	-2.80604700	-1.30000700
H	-1.92229400	-3.94087300	0.02735800
H	-2.56354900	-2.32921200	0.35955600
C	-3.38424500	0.72078200	-1.08623400
H	-3.58521100	-0.34868500	-0.99625000
H	-4.27508100	1.26061300	-0.75438400
H	-3.22754600	0.94343500	-2.14338600
C	-1.57495000	3.05821700	-0.26066200
H	-1.33173700	3.30828500	-1.29526900
H	-2.45857300	3.63383700	0.02716400
H	-0.74361500	3.38620900	0.36747500
C	-2.26381700	0.86139000	1.75376500
H	-1.41483500	1.12164500	2.38940900
H	-3.12904600	1.43972500	2.08964000
H	-2.48618700	-0.19662200	1.91045700

E(UwB97XD, vacuo)	-1517.349336
Zero-point correction=	0.337358
Thermal correction to Energy=	0.362914
Thermal correction to Enthalpy=	0.363858
Thermal correction to Gibbs Free Energy=	**0.281916**
E(UwB97XD, MeCN)	**-1517.351429**

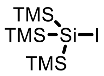

S7

Si	-0.15255600	0.00055600	-0.00019400
Si	-0.86148700	-1.77291000	1.36289200
Si	-0.84791000	2.07435100	0.84940900
Si	-0.83888600	-0.29401000	-2.22465500
C	-0.62890800	-3.39934500	0.45501100
H	0.41230800	-3.53226600	0.15372300
H	-0.90613200	-4.24211200	1.09333100
H	-1.24754300	-3.44199500	-0.44411500
C	0.10775700	-1.79343500	2.96690900
H	-0.23442600	-2.60867300	3.60961600
H	1.17326600	-1.93460200	2.77644300
H	-0.01201300	-0.85659700	3.51444200
C	-2.68806000	-1.50873100	1.72884100
H	-3.08866000	-2.34769200	2.30359300
H	-2.84458300	-0.59989800	2.31417300
H	-3.27355200	-1.42171000	0.81099800
C	0.13548400	-1.67712800	-3.03126500
H	-0.19504800	-1.82655000	-4.06238800
H	1.20210300	-1.44570300	-3.04601000
H	0.00572500	-2.61843100	-2.49370800
C	-2.66652000	-0.73980700	-2.19576200
H	-3.05800800	-0.81988900	-3.21310100
H	-2.82838600	-1.69986500	-1.70052400
H	-3.25599500	0.01334700	-1.66842600
C	-0.59200200	1.30255300	-3.18053900

H	0.44921500	1.62838200	-3.13280300
H	-0.85592700	1.16822900	-4.23260600
H	-1.21464900	2.10476500	-2.77855900
C	0.12961100	3.46496400	0.05948400
H	0.01127700	3.46551200	-1.02576900
H	-0.20789600	4.43305600	0.43849300
H	1.19431500	3.36620500	0.27881500
C	-0.61435900	2.10642300	2.71167800
H	0.42653100	1.91045600	2.97746000
H	-0.88922400	3.08252300	3.11939500
H	-1.23440600	1.35201700	3.20093200
C	-2.67343100	2.26929000	0.43762100
H	-3.26488600	1.43833600	0.82806400
H	-3.06670000	3.19278100	0.87029700
H	-2.83068500	2.31463400	-0.64235900
I	2.34694900	-0.00631700	0.01159200

E(UwB97XD, vacuo)	-1815.233921
Zero-point correction=	0.338909
Thermal correction to Energy=	0.366336
Thermal correction to Enthalpy=	0.367281
Thermal correction to Gibbs Free Energy=	**0.281117**

E(UwB97XD, MeCN) **-1815.237601**

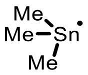

S8

Sn	0.00000200	0.00002000	-0.29626800
C	-1.70175600	1.08249500	0.49917000
H	-1.65131200	1.05005500	1.59003500
H	-1.68466300	2.12278300	0.17666100
H	-2.63670200	0.62637000	0.17619700
C	-0.08664500	-2.01502300	0.49912700
H	0.77677900	-2.59609000	0.17763300
H	-0.08564700	-1.95507300	1.58999300
H	-0.99522500	-2.52099700	0.17515200
C	1.78838900	0.93243300	0.49917400
H	1.73488200	0.90535900	1.59004000
H	2.68064300	0.39709900	0.17701300
H	1.86121500	1.97005600	0.17586100

E(UwB97XD, vacuo)	-334.081207
Zero-point correction=	0.107025
Thermal correction to Energy=	0.115545
Thermal correction to Enthalpy=	0.116490
Thermal correction to Gibbs Free Energy=	**0.072357**

E(UwB97XD, MeCN) **-334.082302**

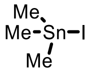

S9

Sn	-0.97610100	-0.00000100	-0.00002500
C	-1.57463000	-0.07660900	2.05072800
H	-2.66295100	-0.07915300	2.12306300
H	-1.18535200	-0.98072500	2.51667500
H	-1.18508800	0.79008500	2.58285200
C	-1.57430600	1.81441800	-0.95904200
H	-1.18864500	1.83977400	-1.97722300
H	-2.66259500	1.88087600	-0.98888600
H	-1.18087900	2.66976400	-0.41162900
C	-1.57438300	-1.73775300	-1.09175900
H	-2.66268500	-1.80102500	-1.12751200
H	-1.18769800	-1.68772400	-2.10864500
H	-1.18208900	-2.63167600	-0.60897100
I	1.74043300	-0.00000900	0.00003700

E(UwB97XD, vacuo)	-631.965891
Zero-point correction=	0.109211
Thermal correction to Energy=	0.119547
Thermal correction to Enthalpy=	0.120491
Thermal correction to Gibbs Free Energy=	**0.070734**
E(UwB97XD, MeCN)	**-631.970799**

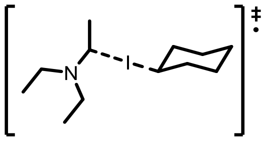

TS6

C	2.50041400	-0.98970600	-0.46562500
N	3.17833700	0.11137100	-0.01368900
C	3.36989600	1.22971200	-0.92889700
H	3.30380600	0.83000400	-1.94094400
H	4.39146700	1.59925500	-0.80691800
C	2.38824900	2.38674300	-0.77164400
H	2.47841700	2.86319800	0.20586700
H	2.60223900	3.14332700	-1.52816700
H	1.35923200	2.05214200	-0.89589400
C	3.16040700	0.42541400	1.40530800
H	2.24415000	0.97817000	1.65212000
H	3.11174900	-0.51158300	1.95513700
C	2.64834000	-2.28953400	0.28055900
H	2.18626300	-2.27269000	1.26632300
H	2.16944800	-3.08478300	-0.28734700
H	3.71085500	-2.53067500	0.39638800
C	4.38573100	1.19903100	1.86439500
H	4.43213200	2.19815100	1.42975000
H	4.35663800	1.31450500	2.94829700
H	5.30123500	0.66723600	1.60029800
H	2.58574900	-1.09516100	-1.54069700
I	-0.00287100	-0.66965600	-0.48216900

C	-2.62310600	-0.28886600	-0.40475000
C	-3.10189200	-0.83491600	0.90697400
C	-2.84913400	1.18226200	-0.58669200
H	-2.85445700	-0.90209100	-1.27241400
C	-4.59325900	-0.50950100	1.09672100
H	-2.53667700	-0.37221700	1.72385500
H	-2.93352400	-1.91114100	0.96977600
C	-4.34024600	1.50997500	-0.39852500
H	-2.27268400	1.73632000	0.16288800
H	-2.50465600	1.51484900	-1.56726200
C	-4.85281300	0.98728400	0.94156700
H	-4.92700000	-0.85550300	2.07836800
H	-5.17884500	-1.05729400	0.35071900
H	-4.49558400	2.58943100	-0.47244000
H	-4.91462600	1.05002300	-1.20970400
H	-5.92021200	1.19730700	1.04435600
H	-4.34680700	1.52403500	1.75209200

E(UwB97XD, vacuo)	-824.854265
Zero-point correction=	0.355752
Thermal correction to Energy=	0.373537
Thermal correction to Enthalpy=	0.374481
Thermal correction to Gibbs Free Energy=	**0.305455**
E(UwB97XD, MeCN)	**-824.860556**

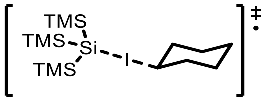

TS7

Si	-1.58622300	0.00121400	-0.04212800
Si	-2.13863700	2.24761300	-0.36413900
Si	-1.91645800	-1.33138100	-1.93194300
Si	-2.44919900	-0.91885500	1.92348900
C	-1.43419000	-0.36819500	-3.47135500
H	-2.06233100	0.51669300	-3.59623900
H	-0.39511800	-0.03723700	-3.41321000
H	-1.54393000	-0.98657500	-4.36600200
C	-3.73762000	-1.79856900	-2.03567500
H	-3.93356400	-2.39756000	-2.92918800
H	-4.04133000	-2.38557000	-1.16589800
H	-4.37279000	-0.91113100	-2.07796800
C	-0.88316000	-2.89361300	-1.81894400
H	-1.04283500	-3.52543100	-2.69666200
H	0.17968700	-2.65139800	-1.75953700
H	-1.14202500	-3.47441100	-0.93141800
C	-0.93523600	3.04981700	-1.55988900
H	-0.94680600	2.54624700	-2.52854800
H	-1.19386200	4.09984300	-1.72006300
H	0.08446000	3.00313000	-1.17270200
C	-3.87954700	2.34836500	-1.07470200
H	-4.18149500	3.38991500	-1.21449900
H	-3.93571900	1.85025600	-2.04530400
H	-4.60582200	1.87211000	-0.41252500

C	-2.08586200	3.15054200	1.28242000
H	-1.10067400	3.05889800	1.74454500
H	-2.30061600	4.21394600	1.14876800
H	-2.82287300	2.74463700	1.97893700
C	-1.62678700	-0.14573700	3.42242500
H	-1.78528600	0.93423500	3.44613100
H	-2.03232700	-0.56861000	4.34536000
H	-0.54981400	-0.32383500	3.40820200
C	-2.15327100	-2.77414000	1.92878200
H	-2.67333600	-3.25907500	1.09960100
H	-1.08863000	-2.99839000	1.83493700
H	-2.51386400	-3.22165200	2.85857700
C	-4.30116200	-0.58461200	1.99049600
H	-4.74277700	-1.03189800	2.88519700
H	-4.50546600	0.48816000	2.01694100
H	-4.81065200	-1.00026100	1.11849900
C	5.68814100	-1.26390400	0.04795200
C	4.15853800	-1.25957300	-0.06266800
C	3.59639900	-0.00371600	0.56619200
C	4.16592800	1.26261400	-0.03433000
C	5.69557900	1.25522600	0.07610300
C	6.29160100	0.00079000	-0.55641200
H	3.87512800	-1.29386500	-1.11979200
H	3.73989600	-2.14921300	0.41058400
H	5.97284500	-1.33263400	1.10336800
H	6.08839800	-2.15295500	-0.44471600
H	3.88244400	1.32268800	-1.09023300
H	3.75296100	2.14381400	0.45931800
H	6.10114800	2.15264900	-0.39663800
H	5.98071000	1.29863500	1.13275200
H	6.09439400	0.01339800	-1.63402700
H	7.37710000	-0.00374000	-0.43532400
H	3.70071200	-0.01621700	1.65145500
I	1.30953200	0.00453500	0.34171800

E(UwB97XD, vacuo)	-2050.439538
Zero-point correction=	0.498400
Thermal correction to Energy=	0.532729
Thermal correction to Enthalpy=	0.533673
Thermal correction to Gibbs Free Energy=	**0.427393**
E(UwB97XD, MeCN)	**-2050.443391**

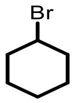

S10

C	2.13215400	-1.25795200	0.19374900
C	0.63924100	-1.26074500	-0.14108400
C	-0.01931200	0.00000000	0.39322900
C	0.63924100	1.26074500	-0.14108400
C	2.13215400	1.25795200	0.19374900
C	2.81953700	0.00000000	-0.32893400
H	0.50481700	-1.29793800	-1.22628200

H	0.15300700	-2.14418400	0.27462500
H	2.25978800	-1.31542300	1.28022200
H	2.59948400	-2.15211000	-0.22348400
H	0.50481800	1.29793700	-1.22628300
H	0.15300600	2.14418400	0.27462500
H	2.59948400	2.15211000	-0.22348400
H	2.25978800	1.31542200	1.28022300
H	2.78642100	0.00000000	-1.42379700
H	3.87402700	0.00000000	-0.04540000
H	0.00259600	0.00000000	1.48332800
Br	-1.93586700	0.00000000	-0.03548700

E(UwB97XD, vacuo) -2809.514453
Zero-point correction= 0.162042
Thermal correction to Energy= 0.168931
Thermal correction to Enthalpy= 0.169876
Thermal correction to Gibbs Free Energy= **0.129990**

E(UwB97XD, MeCN) **-2809.517658**

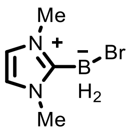

S11

C	2.53747500	-0.67472200	-0.57598500
C	2.53753000	0.67480500	-0.57583500
C	0.70756100	-0.00000700	0.50710000
N	1.40631100	-1.06997200	0.09669600
H	3.23561700	-1.37799300	-0.99278900
H	3.23570800	1.37811200	-0.99251600
N	1.40625800	1.06999500	0.09669900
B	-0.70119500	-0.00004500	1.25570800
H	-0.84682000	-1.00873200	1.89615500
H	-0.84684000	1.00860000	1.89621500
C	0.96935100	-2.44279900	0.28054900
H	0.98714600	-2.70521500	1.33626800
H	-0.04618000	-2.55021700	-0.09616400
H	1.63950000	-3.09629100	-0.27311300
C	0.96922700	2.44280200	0.28053700
H	1.63940000	3.09633000	-0.27305500
H	-0.04627900	2.55020100	-0.09625100
H	0.98693400	2.70519200	1.33626400
Br	-2.06991600	-0.00001200	-0.31061500

E(UwB97XD, vacuo) -2905.180334
Zero-point correction= 0.154569
Thermal correction to Energy= 0.164531
Thermal correction to Enthalpy= 0.165475
Thermal correction to Gibbs Free Energy= **0.117166**

E(UwB97XD, MeCN) **-2905.197546**

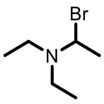

S12

C	-0.29945100	0.69342700	0.80128100
N	0.98244500	0.30725800	0.49394200
C	1.46235300	-0.95619900	1.04496200
H	0.90631900	-1.13624900	1.96572900
H	2.50691900	-0.82269200	1.33679800
C	1.34189300	-2.17155900	0.13039600
H	1.95003900	-2.06118800	-0.76888200
H	1.69765500	-3.05585200	0.66151000
H	0.30965400	-2.33890200	-0.17281400
C	1.55071000	0.71168800	-0.78303900
H	1.19657900	0.04602700	-1.58064600
H	1.17361100	1.70416900	-1.01936300
C	-0.65402100	2.15184200	0.62540700
H	-0.68334900	2.45451300	-0.41889100
H	-1.63885300	2.33629600	1.04892700
H	0.08282700	2.76135000	1.15485000
C	3.06984400	0.75917200	-0.77020400
H	3.51404500	-0.22973300	-0.65140400
H	3.42841000	1.16731400	-1.71562700
H	3.43091500	1.39674900	0.03839500
H	-0.57784000	0.32653500	1.78268000
Br	-1.80005700	-0.33798200	-0.32604800

E(UwB97XD, vacuo)	-2866.064313
Zero-point correction=	0.198033
Thermal correction to Energy=	0.208611
Thermal correction to Enthalpy=	0.209555
Thermal correction to Gibbs Free Energy=	**0.161324**
E(UwB97XD, MeCN)	**-2866.071034**

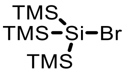

S13

Si	-0.00070900	-0.00035000	0.13734800
Si	0.96725600	2.02024300	-0.55838800
Si	1.26757000	-1.84601800	-0.56103500
Si	-2.23291300	-0.17261700	-0.56331900
C	-0.25599000	3.42728200	-0.33579500
H	-0.58664900	3.49575900	0.70269000
H	0.19839400	4.38294700	-0.60927400
H	-1.14000900	3.28527900	-0.96134500
C	2.51878200	2.36286700	0.43662900
H	2.97841800	3.30309200	0.12142900
H	2.28836400	2.43662500	1.50107100
H	3.25426300	1.56617600	0.30921500
C	1.40232700	1.84391200	-2.38037700
H	1.80046800	2.78310000	-2.77277600
H	2.16083800	1.07237400	-2.53064200

H	0.52832700	1.57568400	-2.97797400
C	-3.30634100	1.00364000	0.42585800
H	-4.34997200	0.93116100	0.10929400
H	-3.25684100	0.77076200	1.49117500
H	-2.98323700	2.03822100	0.29539400
C	-2.29292800	0.28657100	-2.38692400
H	-3.30493500	0.16365700	-2.78099100
H	-2.00049400	1.32785300	-2.53994900
H	-1.62435300	-0.34023000	-2.98115700
C	-2.84138100	-1.93425200	-0.33575300
H	-2.73656000	-2.25136200	0.70391900
H	-3.89597700	-2.01878800	-0.60997800
H	-2.27632900	-2.63133900	-0.95839500
C	0.78487300	-3.36509000	0.42583800
H	-0.27223400	-3.60331700	0.29307100
H	1.37083400	-4.23189100	0.10967300
H	0.95950000	-3.20659000	1.49164700
C	3.09654400	-1.48992200	-0.32925300
H	3.31646000	-1.24084400	0.71096700
H	3.69874400	-2.36002600	-0.60279300
H	3.41769000	-0.65117200	-0.95084800
C	0.90510600	-2.12717700	-2.38573000
H	1.11225900	-1.23357000	-2.97858200
H	1.52114900	-2.93983400	-2.77892600
H	-0.14162000	-2.39778000	-2.54167600
Br	-0.00340900	-0.00258700	2.41612300

E(UwB97XD, vacuo)	-4091.666727
Zero-point correction=	0.338995
Thermal correction to Energy=	0.366402
Thermal correction to Enthalpy=	0.367347
Thermal correction to Gibbs Free Energy=	**0.281316**
E(UwB97XD, MeCN)	**-4091.670598**

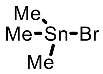

S14

Sn	-0.63861200	-0.00000400	-0.00002500
C	-1.21158800	0.06360800	2.05608700
H	-2.29859200	0.06569800	2.14526300
H	-0.81459300	-0.80640400	2.57730900
H	-0.81572900	0.96486200	2.52218100
C	-1.21139400	1.74889500	-1.08319000
H	-0.81521300	1.70201200	-2.09662500
H	-2.29838000	1.82514500	-1.12988500
H	-0.81448100	2.63522800	-0.59015100
C	-1.21126200	-1.81257800	-0.97298400
H	-2.29824500	-1.89112800	-1.01578500
H	-0.81422000	-1.82887200	-1.98703900
H	-0.81516500	-2.66672400	-0.42548500
Br	1.87202000	0.00002400	0.00005600

E(UwB97XD, vacuo)	-2908.398079
Zero-point correction=	0.109364
Thermal correction to Energy=	0.119615
Thermal correction to Enthalpy=	0.120559
Thermal correction to Gibbs Free Energy=	**0.071606**
E(UwB97XD, MeCN)	**-2908.403824**

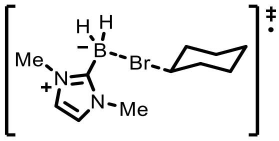

TS8

C	4.39821200	1.27230000	-0.54255100
C	2.94460000	1.26168200	-0.04811700
C	2.26364400	-0.01293700	-0.46441200
C	2.97368200	-1.25666800	-0.00550200
C	4.42734600	-1.25050100	-0.49974100
C	5.14824300	0.02694000	-0.07547300
H	2.93945800	1.33151700	1.04486000
H	2.40425000	2.13102400	-0.42691200
H	4.40715300	1.30957300	-1.63743600
H	4.90329600	2.17592700	-0.19170500
H	2.96960400	-1.28957500	1.08920700
H	2.45363300	-2.15054600	-0.35430800
H	4.95297400	-2.12977900	-0.11853800
H	4.43771900	-1.32471500	-1.59273000
H	5.22810200	0.04644000	1.01718200
H	6.16888700	0.03215100	-0.46563400
H	1.98706400	-0.03388300	-1.51719100
C	-3.89304000	0.71320800	-1.31927500
C	-3.90418100	-0.63548700	-1.34549500
C	-2.79780600	-0.00587800	0.49911600
N	-3.22100000	1.08784300	-0.18127600
H	-4.30760900	1.43025800	-2.00473700
H	-4.33037700	-1.31850700	-2.05804500
N	-3.23882900	-1.06514700	-0.22309300
B	-1.89637200	-0.03708100	1.75148200
H	-1.80039000	0.98330900	2.36822500
H	-1.81269100	-1.08275200	2.32620700
C	-2.89556100	2.44490600	0.19946600
H	-3.28731200	2.66142100	1.19202700
H	-1.81285500	2.57089600	0.21376100
H	-3.33699000	3.12658500	-0.52424900
C	-2.93626200	-2.44119700	0.10466300
H	-3.39325600	-3.08716500	-0.64180000
H	-1.85601400	-2.58737200	0.10877900
H	-3.32745000	-2.68771100	1.09040900
Br	0.19257700	-0.02225000	0.46113500

E(UwB97XD, vacuo)	-3140.377613
Zero-point correction=	0.312330
Thermal correction to Energy=	0.329305

```
Thermal correction to Enthalpy=                    0.330250
Thermal correction to Gibbs Free Energy=           0.263123

E(UwB97XD, MeCN)                               -3140.391117
```

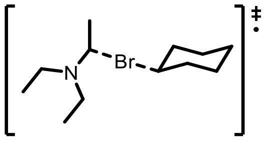

TS9

C	2.34233800	-1.05155400	-0.57534600
N	2.98163100	0.04979900	-0.07627000
C	3.11113900	1.21669900	-0.94011300
H	3.08369000	0.85727700	-1.96900400
H	4.10407900	1.64723100	-0.78763500
C	2.05035600	2.29602400	-0.74754300
H	2.09680400	2.73294200	0.25148800
H	2.21901200	3.09924700	-1.46672200
H	1.05009600	1.89274800	-0.89918600
C	2.95520800	0.29915600	1.35634200
H	2.00850100	0.78031400	1.63427500
H	2.97377900	-0.66287800	1.86342000
C	2.47738500	-2.36789600	0.13515700
H	1.98076100	-2.37678600	1.10452300
H	2.01980200	-3.14798700	-0.46988900
H	3.53542900	-2.61239000	0.28122400
C	4.13212300	1.12921100	1.84274000
H	4.10638700	2.14983000	1.45927200
H	4.10883400	1.18791100	2.93137900
H	5.07780300	0.67400000	1.54389000
H	2.42630500	-1.11476800	-1.65351300
C	-2.36888000	-0.39690100	-0.48633000
C	-2.83712400	-0.95642700	0.82374300
C	-2.56664400	1.08252100	-0.63273800
H	-2.64722000	-0.98289300	-1.35885100
C	-4.31504500	-0.60089400	1.05152700
H	-2.24184000	-0.52296100	1.63530400
H	-2.69137600	-2.03719300	0.86124000
C	-4.04391700	1.44243000	-0.40773600
H	-1.95994400	1.60430800	0.11633800
H	-2.23232800	1.42641500	-1.61310000
C	-4.54266800	0.90444700	0.93170000
H	-4.63727500	-0.95864600	2.03286200
H	-4.92937400	-1.11938500	0.30758600
H	-4.17484000	2.52660300	-0.45626200
H	-4.64614900	1.01379300	-1.21594400
H	-5.60256700	1.13704400	1.06015100
H	-4.00826300	1.41259000	1.74248000
Br	-0.00734700	-0.76959600	-0.58599600

```
E(UwB97XD, vacuo)                              -3101.278798
Zero-point correction=                             0.356184
Thermal correction to Energy=                      0.373776
```

```
Thermal correction to Enthalpy=                    0.374720
Thermal correction to Gibbs Free Energy=           0.307136

E(UwB97XD, MeCN)                               -3101.284838
```

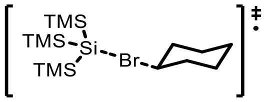

TS10

C	2.34233800	-1.05155400	-0.57534600
N	2.98163100	0.04979900	-0.07627000
C	3.11113900	1.21669900	-0.94011300
H	3.08369000	0.85727700	-1.96900400
H	4.10407900	1.64723100	-0.78763500
C	2.05035600	2.29602400	-0.74754300
H	2.09680400	2.73294200	0.25148800
H	2.21901200	3.09924700	-1.46672200
H	1.05009600	1.89274800	-0.89918600
C	2.95520800	0.29915600	1.35634200
H	2.00850100	0.78031400	1.63427500
H	2.97377900	-0.66287800	1.86342000
C	2.47738500	-2.36789600	0.13515700
H	1.98076100	-2.37678600	1.10452300
H	2.01980200	-3.14798700	-0.46988900
H	3.53542900	-2.61239000	0.28122400
C	4.13212300	1.12921100	1.84274000
H	4.10638700	2.14983000	1.45927200
H	4.10883400	1.18791100	2.93137900
H	5.07780300	0.67400000	1.54389000
H	2.42630500	-1.11476800	-1.65351300
C	-2.36888000	-0.39690100	-0.48633000
C	-2.83712400	-0.95642700	0.82374300
C	-2.56664400	1.08252100	-0.63273800
H	-2.64722000	-0.98289300	-1.35885100
C	-4.31504500	-0.60089400	1.05152700
H	-2.24184000	-0.52296100	1.63530400
H	-2.69137600	-2.03719300	0.86124000
C	-4.04391700	1.44243000	-0.40773600
H	-1.95994400	1.60430800	0.11633800
H	-2.23232800	1.42641500	-1.61310000
C	-4.54266800	0.90444700	0.93170000
H	-4.63727500	-0.95864600	2.03286200
H	-4.92937400	-1.11938500	0.30758600
H	-4.17484000	2.52660300	-0.45626200
H	-4.64614900	1.01379300	-1.21594400
H	-5.60256700	1.13704400	1.06015100
H	-4.00826300	1.41259000	1.74248000
Br	-0.00734700	-0.76959600	-0.58599600

```
E(UwB97XD, vacuo)                              -3101.278798
Zero-point correction=                             0.356184
Thermal correction to Energy=                      0.373776
Thermal correction to Enthalpy=                    0.374720
Thermal correction to Gibbs Free Energy=           0.307136
```

E(UwB97XD, MeCN) -3101.284838

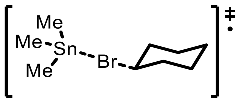

TS11

C	-4.69418100	-1.26032700	-0.00563200
C	-3.16247600	-1.26051000	0.08174200
C	-2.61290900	-0.00013700	-0.53768300
C	-3.16218700	1.26044000	0.08158400
C	-4.69389200	1.26059900	-0.00578800
C	-5.28441700	0.00024100	0.62097700
H	-2.86228400	-1.30527600	1.13358600
H	-2.75007500	-2.14301400	-0.40960500
H	-4.99587700	-1.31754000	-1.05697600
H	-5.08959600	-2.15302500	0.48427600
H	-2.86198600	1.30526600	1.13342300
H	-2.74958100	2.14278800	-0.40987000
H	-5.08910000	2.15344700	0.48401200
H	-4.99557600	1.31775300	-1.05713800
H	-5.07190600	0.00028300	1.69573100
H	-6.37152500	0.00036000	0.51523400
H	-2.69612500	-0.00019700	-1.62381700
Sn	2.38008100	0.00002300	0.07077500
C	3.24083100	-1.75061400	-0.85037900
H	4.32131300	-1.75038200	-0.69287800
H	2.82068100	-2.65484300	-0.41159500
H	3.04041900	-1.75279800	-1.92112400
C	3.23507800	1.76917200	-0.81981900
H	2.81116100	2.66428100	-0.36622000
H	4.31542000	1.77032900	-0.66136900
H	3.03555400	1.78867900	-1.89055700
C	2.81865700	-0.01764200	2.18281500
H	3.90051200	-0.01653700	2.33043700
H	2.39614600	0.86327800	2.66467200
H	2.40009100	-0.90881300	2.64901700
Br	-0.47482100	-0.00035800	-0.30328200

E(UwB97XD, vacuo) -3143.592934
Zero-point correction= 0.268204
Thermal correction to Energy= 0.285515
Thermal correction to Enthalpy= 0.286459
Thermal correction to Gibbs Free Energy= 0.216635

E(UwB97XD, MeCN) -3143.596689

11. Cyclic voltammetry

Since we hypothesized that the success of the reported manifold would also depend on the redox potentials of the borane (and the photocatalyst), we performed cyclic voltammetry measurements (see **Figure S21** below) to determine the oxidation potential of different boranes.

The electrochemical measurements were carried out with a PalmSens EmStat3+. Electrochemical measurements (cyclic voltammetry) were performed in a three-electrodes cell (volume 8 mL; tetrabutylammonium perchlorate (TBAP) 0.1 M in MeCN, scan rate: 100 mV/s, WE: glassy carbon disk, CE: Pt wire; RE: $AgNO_3$ 0.01 M/TBAP 0.1 M, 2 mM concentration of the tested compound).

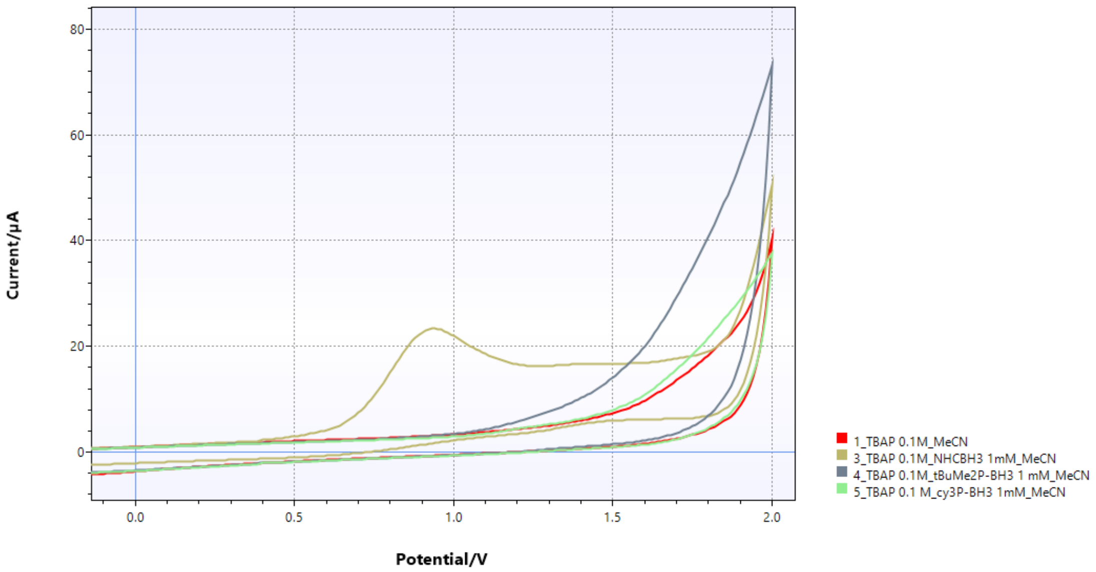

Figure S21: Cyclic voltammetry profiles of *t*-butyl dimethyl phosphine borane (in blue) and tricyclohexyl phosphine borane (in green); that of model ligated borane **B1** (in yellow) is also shown for comparison.

Figure S21 clearly shows the uniqueness of model borane **B1**: it can be oxidized by the commercially available acridinium dye ($E(Acr^*/Acr_{red})$ = +2.06 V vs SCE; $E_{pa}(\mathbf{B1}^{\bullet+}/\mathbf{B1})$= +0.89 V vs SCE), thus delivering the coveted ligated boryl radical under the described photoredox conditions.

The oxidation potential of trimethylamine borane complex has been reported by Leonori to be $E_{1/2}^{ox}$ = +2.60 V vs SCE.[40]

12. References

(1) Stoll, S.; Schweiger, A. EasySpin, a Comprehensive Software Package for Spectral Simulation and Analysis in EPR. *J. Magn. Reson.* **2006**, *178* (1), 42–55. https://doi.org/10.1016/j.jmr.2005.08.013.

(2) Thomas, C. cwEPR - File Exchange - MATLAB Central https://nl.mathworks.com/matlabcentral/fileexchange/73292-cwepr (accessed Jul 25, 2022).

(3) Gardner, S.; Kawamoto, T.; Curran, D. P. Synthesis of 1,3-Dialkylimidazol-2-Ylidene Boranes from 1,3-Dialkylimidazolium Iodides and Sodium Borohydride. *J. Org. Chem.* **2015**, *80* (19), 9794–9797. https://doi.org/10.1021/acs.joc.5b01682.

(4) Lutter, F. H.; Grokenberger, L.; Benz, M.; Knochel, P. Cobalt-Catalyzed Csp 3 –Csp 3 Cross-Coupling of Functionalized Alkylzinc Reagents with Alkyl Iodides. *Org. Lett.* **2020**, *22* (8), 3028–3032. https://doi.org/10.1021/acs.orglett.0c00795.

(5) Barré, B.; Gonnard, L.; Campagne, R.; Reymond, S.; Marin, J.; Ciapetti, P.; Brellier, M.; Guérinot, A.; Cossy, J. Iron- and Cobalt-Catalyzed Arylation of Azetidines, Pyrrolidines, and Piperidines with Grignard Reagents. *Org. Lett.* **2014**, *16* (23), 6160–6163. https://doi.org/10.1021/ol503043r.

(6) Artaryan, A.; Mardyukov, A.; Kulbitski, K.; Avigdori, I.; Nisnevich, G. A.; Schreiner, P. R.; Gandelman, M. Aliphatic C–H Bond Iodination by a N -Iodoamide and Isolation of an Elusive N -Amidyl Radical. *J. Org. Chem.* **2017**, *82* (14), 7093–7100. https://doi.org/10.1021/acs.joc.7b00557.

(7) Chen, K.-Q.; Wang, Z.-X.; Chen, X.-Y. Photochemical Decarboxylative C(Sp 3)–X Coupling Facilitated by Weak Interaction of N-Heterocyclic Carbene. *Org. Lett.* **2020**, *22* (20), 8059–8064. https://doi.org/10.1021/acs.orglett.0c03006.

(8) Matier, C. D.; Schwaben, J.; Peters, J. C.; Fu, G. C. Copper-Catalyzed Alkylation of Aliphatic Amines Induced by Visible Light. *J. Am. Chem. Soc.* **2017**, *139* (49), 17707–17710. https://doi.org/10.1021/jacs.7b09582.

(9) Harman, D. G.; Blanksby, S. J. Investigation of the Gas Phase Reactivity of the 1-Adamantyl Radical Using a Distonic Radical Anion Approach. *Org. Biomol. Chem.* **2007**, *5* (21), 3495. https://doi.org/10.1039/b711156h.

(10) Liu, W.; Li, L.; Chen, Z.; Li, C.-J. A Transition-Metal-Free Heck-Type Reaction between Alkenes and Alkyl Iodides Enabled by Light in Water. *Org. Biomol. Chem.* **2015**, *13* (22), 6170–6174. https://doi.org/10.1039/C5OB00515A.

(11) Aydin, B. O.; Anil, D.; Demir, Y. Synthesis of N -alkylated Pyrazolo[3,4- d]Pyrimidine Analogs and Evaluation of Acetylcholinesterase and Carbonic Anhydrase Inhibition Properties. *Arch. Pharm. (Weinheim).* **2021**, *354* (5), 2000330. https://doi.org/10.1002/ardp.202000330.

(12) Chen, J.; Lin, J.-H.; Xiao, J.-C. Halogenation through Deoxygenation of Alcohols and Aldehydes. *Org. Lett.* **2018**, *20* (10), 3061–3064. https://doi.org/10.1021/acs.orglett.8b01058.

(13) Donslund, A. S.; Pedersen, S. S.; Gaardbo, C.; Neumann, K. T.; Kingston, L.; Elmore, C. S.; Skrydstrup, T. Direct Access to Isotopically Labeled Aliphatic Ketones Mediated by Nickel(I) Activation. *Angew. Chemie Int. Ed.* **2020**, *59* (21), 8099–8103. https://doi.org/10.1002/anie.201916391.

(14) Combe, S. H.; Hosseini, A.; Song, L.; Hausmann, H.; Schreiner, P. R. Catalytic Halogen Bond Activation in the Benzylic C–H Bond Iodination with Iodohydantoins. *Org. Lett.* **2017**, *19* (22), 6156–6159.

https://doi.org/10.1021/acs.orglett.7b03034.

(15) Ueng, S. H.; Fensterbank, L.; Lacôte, E.; Malacria, M.; Curran, D. P. Radical Reductions of Alkyl Halides Bearing Electron Withdrawing Groups with N-Heterocyclic Carbene Boranes. *Org. Biomol. Chem.* **2011**, *9* (9), 3415–3420. https://doi.org/10.1039/C0OB01075H.

(16) Caputo, D. F. J.; Arroniz, C.; Dürr, A. B.; Mousseau, J. J.; Stepan, A. F.; Mansfield, S. J.; Anderson, E. A. Synthesis and Applications of Highly Functionalized 1-Halo-3-Substituted Bicyclo[1.1.1]Pentanes. *Chem. Sci.* **2018**, *9* (23), 5295–5300. https://doi.org/10.1039/C8SC01355A.

(17) Gurjar, M. K.; Yellol, G. S.; Mohapatra, D. K. A Carbohydrate-Based Synthesis of the C13-C22 Fragment of Amphidinolide X. *European J. Org. Chem.* **2012**, *2012* (9), 1753–1758. https://doi.org/10.1002/ejoc.201101605.

(18) Rousseau, J.; Kriščiūnienė, V.; Rimkevičiūtė, I.; Rousseau, C.; Amankavičienė, V.; Šačkus, A.; Tatibouët, A.; Rollin, P. Modular Access to Heterocycles: Methyl 3-Aminobenzo[b]Thiophene-2-Carboxylate–Thiourea Linkage or Pyrimidine-4-One-2-Thione Formation. *Monatshefte für Chemie - Chem. Mon.* **2009**, *140* (3), 339–348. https://doi.org/10.1007/s00706-008-0030-5.

(19) Nugent, J.; Arroniz, C.; Shire, B. R.; Sterling, A. J.; Pickford, H. D.; Wong, M. L. J.; Mansfield, S. J.; Caputo, D. F. J.; Owen, B.; Mousseau, J. J.; Duarte, F.; Anderson, E. A. A General Route to Bicyclo[1.1.1]Pentanes through Photoredox Catalysis. *ACS Catal.* **2019**, *9* (10), 9568–9574. https://doi.org/10.1021/acscatal.9b03190.

(20) Yamamoto, T.; Iwasaki, T.; Morita, T.; Yoshimi, Y. Strategy for O -Alkylation of Serine and Threonine from Serinyl and Threoninyl Acetic Acids by Photoinduced Decarboxylative Radical Reactions: Connection between Serine/Threonine and Carbohydrates/Amino Acids at the Side Chain. *J. Org. Chem.* **2018**, *83* (7), 3702–3709. https://doi.org/10.1021/acs.joc.8b00061.

(21) Tsudaka, T.; Kotani, H.; Ohkubo, K.; Nakagawa, T.; Tkachenko, N. V.; Lemmetyinen, H.; Fukuzumi, S. Photoinduced Electron Transfer in 9-Substituted 10-Methylacridinium Ions. *Chem. - A Eur. J.* **2017**, *23* (6), 1306–1317. https://doi.org/10.1002/chem.201604527.

(22) Pan, X.; Lacôte, E.; Lalevée, J.; Curran, D. P. Polarity Reversal Catalysis in Radical Reductions of Halides by N-Heterocyclic Carbene Boranes. *J. Am. Chem. Soc.* **2012**, *134* (12), 5669–5674. https://doi.org/10.1021/ja300416f.

(23) Supranovich, V. I.; Levin, V. V.; Struchkova, M. I.; Korlyukov, A. A.; Dilman, A. D. Radical Silyldifluoromethylation of Electron-Deficient Alkenes. *Org. Lett.* **2017**, *19* (12), 3215–3218. https://doi.org/10.1021/acs.orglett.7b01334.

(24) MacKenzie, I. A.; Wang, L.; Onuska, N. P. R.; Williams, O. F.; Begam, K.; Moran, A. M.; Dunietz, B. D.; Nicewicz, D. A. Discovery and Characterization of an Acridine Radical Photoreductant. *Nature* **2020**, *580* (7801), 76–80. https://doi.org/10.1038/s41586-020-2131-1.

(25) A New Sensitive Chemical Actinometer - II. Potassium Ferrioxalate as a Standard Chemical Actinometer. *Proc. R. Soc. London. Ser. A. Math. Phys. Sci.* **1956**, *235* (1203), 518–536. https://doi.org/10.1098/rspa.1956.0102.

(26) Reiß, B.; Hu, Q.; Riedle, E.; Wagenknecht, H. The Dependence of Chemical Quantum Yields of Visible Light Photoredox Catalysis on the Irradiation Power. *ChemPhotoChem* **2021**, *5* (11), 1009–1019. https://doi.org/10.1002/cptc.202100090.

(27) Pitre, S. P.; McTiernan, C. D.; Vine, W.; DiPucchio, R.; Grenier, M.; Scaiano, J. C. Visible-Light Actinometry and Intermittent Illumination as Convenient Tools to Study Ru(Bpy)3Cl2 Mediated Photoredox Transformations. *Sci. Rep.* **2015**, *5* (1), 16397. https://doi.org/10.1038/srep16397.

(28) Pak, Y. L.; Park, S. J.; Wu, D.; Cheon, B.; Kim, H. M.; Bouffard, J.; Yoon, J. N-Heterocyclic Carbene Boranes as Reactive Oxygen Species-Responsive Materials: Application to the Two-Photon Imaging of Hypochlorous Acid in Living Cells and Tissues. *Angew. Chemie Int. Ed.* **2018**, *57* (6), 1567–1571. https://doi.org/10.1002/anie.201711188.

(29) Constantin, T.; Zanini, M.; Regni, A.; Sheikh, N. S.; Juliá, F.; Leonori, D. Aminoalkyl Radicals as Halogen-Atom Transfer Agents for Activation of Alkyl and Aryl Halides. *Science (80-.).* **2020**, *367* (6481), 1021–1026. https://doi.org/10.1126/science.aba2419.

(30) Rohe, S.; Morris, A. O.; McCallum, T.; Barriault, L. Hydrogen Atom Transfer Reactions via Photoredox Catalyzed Chlorine Atom Generation. *Angew. Chemie Int. Ed.* **2018**, *57* (48), 15664–15669. https://doi.org/10.1002/anie.201810187.

(31) El-Hage, F.; Schöll, C.; Pospech, J. Photo-Mediated Decarboxylative Giese-Type Reaction Using Organic Pyrimidopteridine Photoredox Catalysts. *J. Org. Chem.* **2020**, *85* (21), 13853–13867. https://doi.org/10.1021/acs.joc.0c01955.

(32) Fini, F.; Beltrani, M.; Mancuso, R.; Gabriele, B.; Carfagna, C. Selective Aryl α-Diimine/Palladium-Catalyzed Bis-Alkoxy- Carbonylation of Olefins for the Synthesis of Substituted Succinic Diesters. *Adv. Synth. Catal.* **2015**, *357* (1), 177–184. https://doi.org/10.1002/ADSC.201400501.

(33) Xue, F.; Wang, F.; Liu, J.; Di, J.; Liao, Q.; Lu, H.; Zhu, M.; He, L.; He, H.; Zhang, D.; Song, H.; Liu, X.; Qin, Y. A Desulfurative Strategy for the Generation of Alkyl Radicals Enabled by Visible-Light Photoredox Catalysis. *Angew. Chemie Int. Ed.* **2018**, *57* (22), 6667–6671. https://doi.org/10.1002/anie.201802710.

(34) Huang, M.; Tang, M.; Hu, J.; Westcott, S. A.; Radius, U.; Marder, T. B. Cu-Mediated vs. Cu-Free Selective Borylation of Aryl Alkyl Sulfones. *Chem. Commun.* **2022**, *58* (3), 395–398. https://doi.org/10.1039/D1CC06144E.

(35) An, Q.; Wang, Z.; Chen, Y.; Wang, X.; Zhang, K.; Pan, H.; Liu, W.; Zuo, Z. Cerium-Catalyzed C–H Functionalizations of Alkanes Utilizing Alcohols as Hydrogen Atom Transfer Agents. *J. Am. Chem. Soc.* **2020**, *142* (13), 6216–6226. https://doi.org/10.1021/jacs.0c00212.

(36) Zhou, Z.; Kweon, J.; Jung, H.; Kim, D.; Seo, S.; Chang, S. Photoinduced Transition-Metal-Free Chan–Evans–Lam-Type Coupling: Dual Photoexcitation Mode with Halide Anion Effect. *J. Am. Chem. Soc.* **2022**, *144* (20), 9161–9171. https://doi.org/10.1021/jacs.2c03343.

(37) Yasu, Y.; Koike, T.; Akita, M. Visible Light-Induced Selective Generation of Radicals from Organoborates by Photoredox Catalysis. *Adv. Synth. Catal.* **2012**, *354* (18), 3414–3420. https://doi.org/10.1002/ADSC.201200588.

(38) Geant, P.-Y.; Mohamed, B. S.; Périgaud, C.; Peyrottes, S.; Uttaro, J.-P.; Mathé, C. Probing the Reactivity of H-Phosphonate Derivatives for the Hydrophosphonylation of Various Alkenes and Alkynes under Free-Radical Conditions. *New J. Chem.* **2016**, *40* (6), 5318–5324. https://doi.org/10.1039/C6NJ00123H.

(39) Rackl, D.; Kreitmeier, P.; Reiser, O. Synthesis of a Polyisobutylene-Tagged Fac-Ir(Ppy) 3 Complex and Its Application as Recyclable Visible-Light Photocatalyst in a Continuous Flow Process. *Green Chem.*

2016, *18* (1), 214–219. https://doi.org/10.1039/C5GC01792K.

(40) Kim, J. H.; Constantin, T.; Simonetti, M.; Llaveria, J.; Sheikh, N. S.; Leonori, D. A Radical Approach for the Selective C–H Borylation of Azines. *Nature* **2021**, *595* (7869), 677–683. https://doi.org/10.1038/s41586-021-03637-6.

13. NMR spectra

13.1 NMR spectra of starting materials

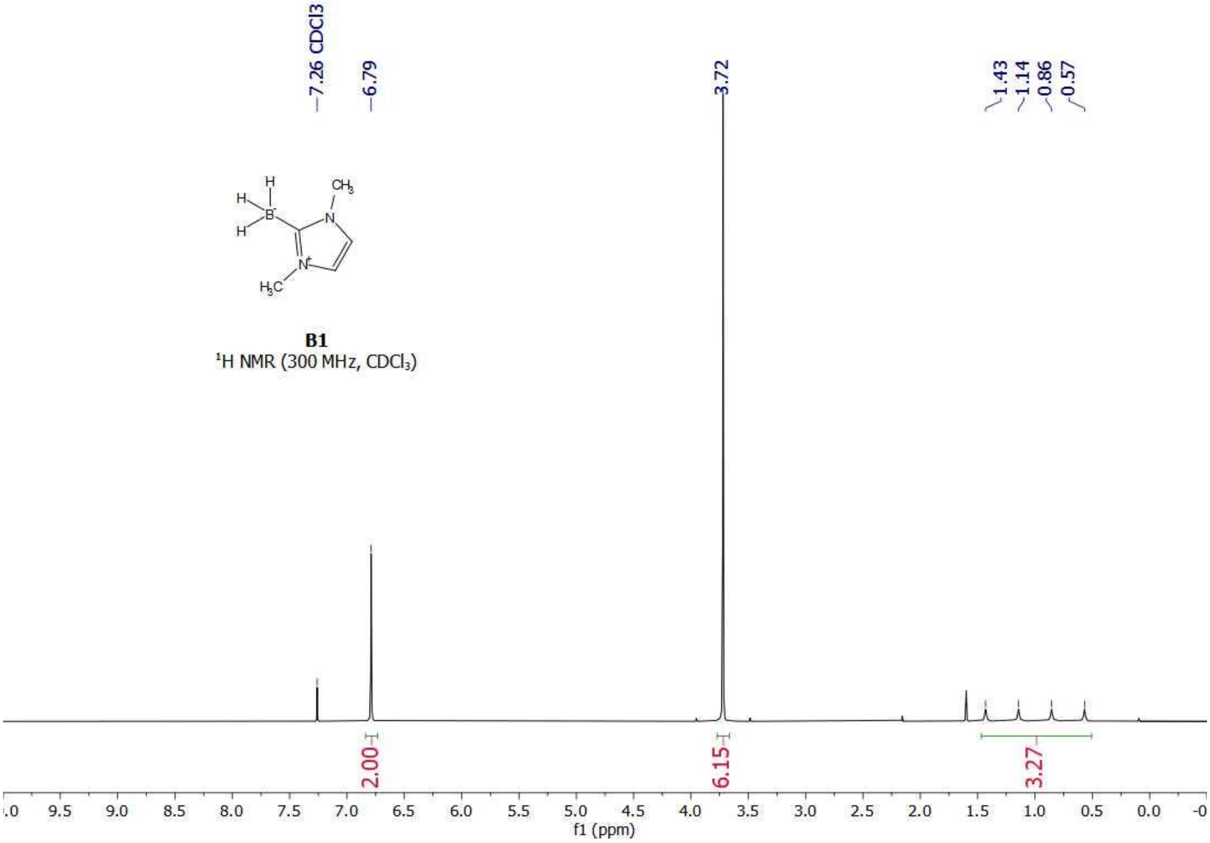

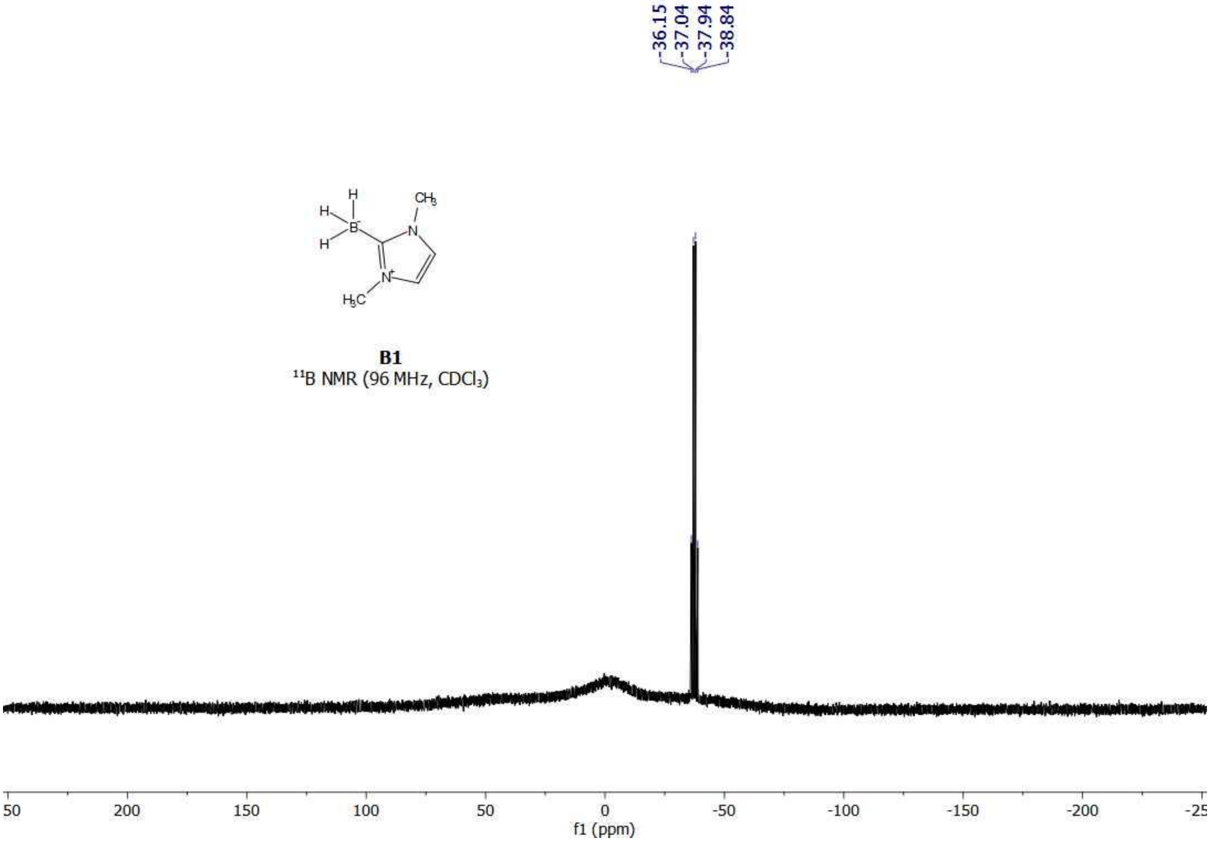

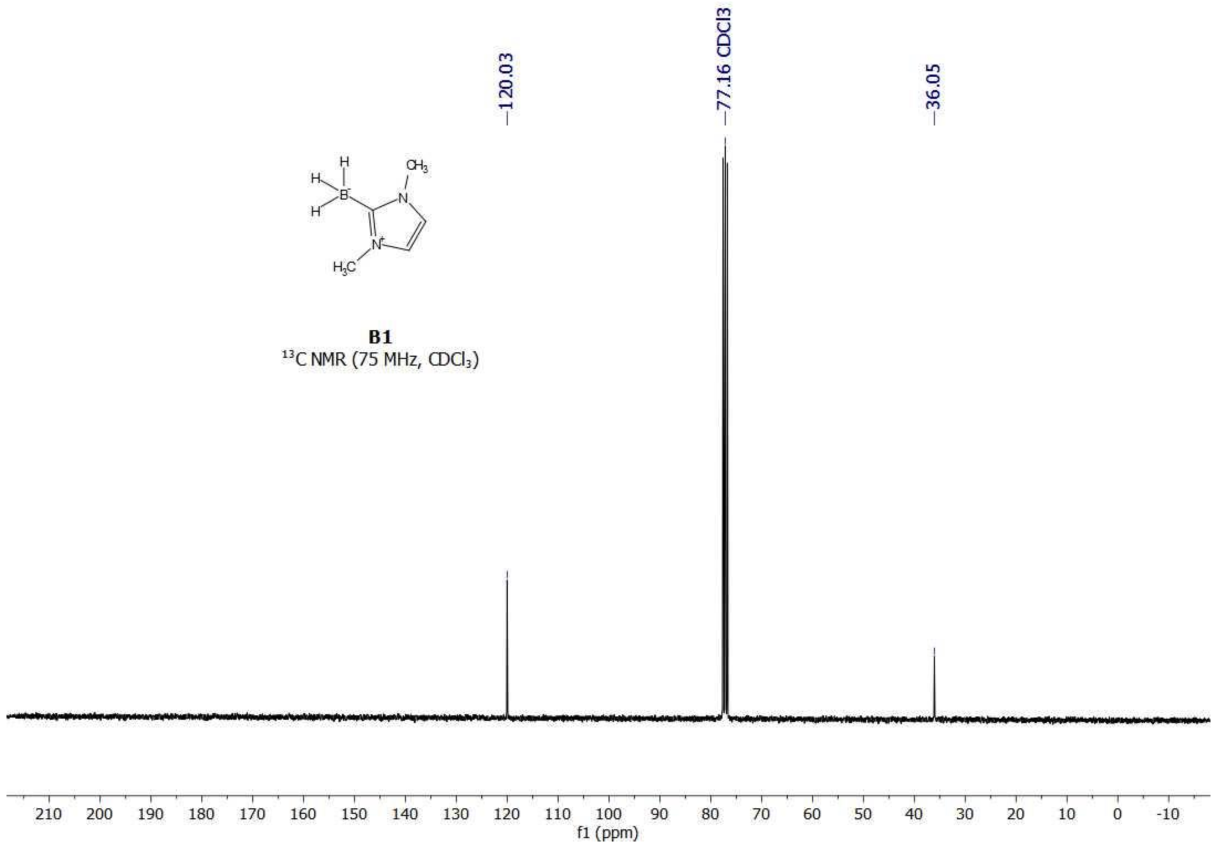

120.03
77.16 CDCl3
36.05
B1
13C NMR (75 MHz, CDCl3)
210 200 190 180 170 160 150 140 130 120 110 100 90 80 70 60 50 40 30 20 10 0 -10
f1 (ppm)

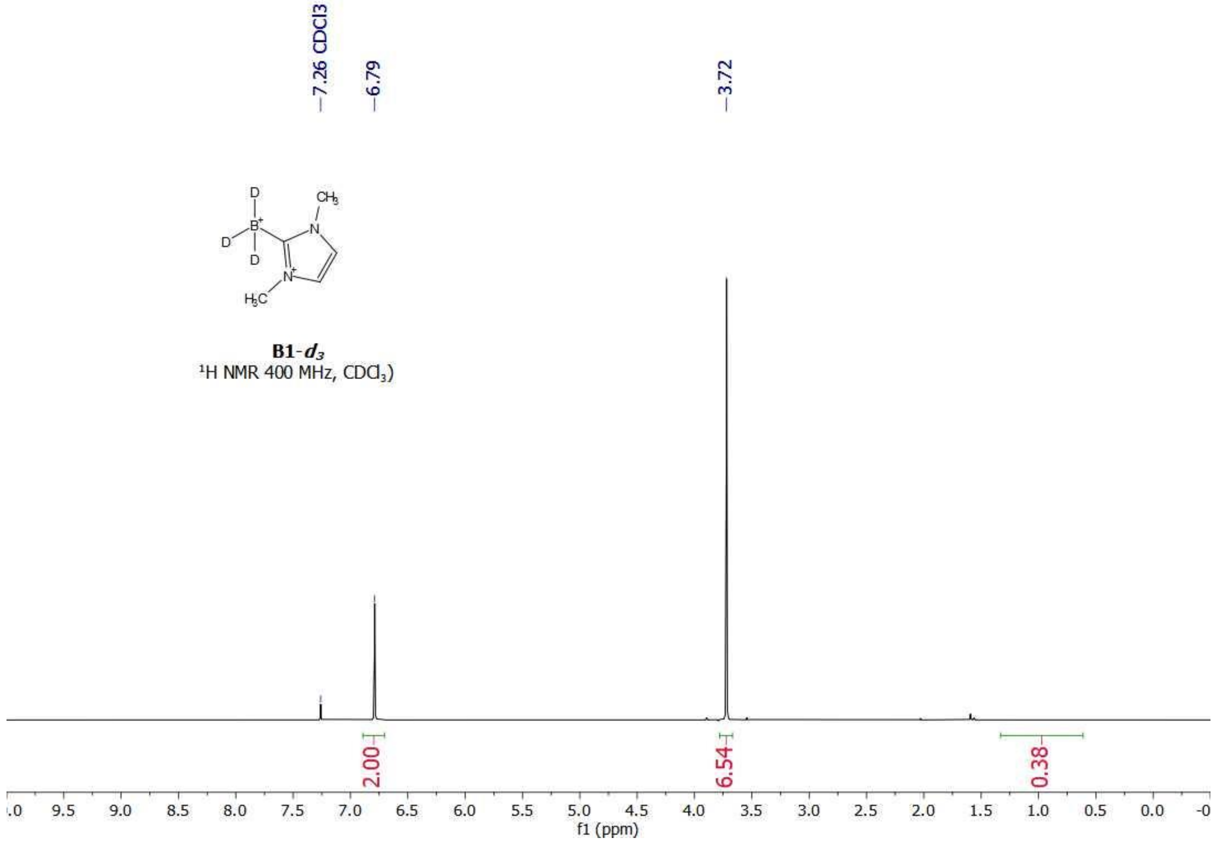

7.26 CDCl3
6.79
3.72
B1-d3
1H NMR 400 MHz, CDCl3)
2.00
6.54
0.38
9.5 9.0 8.5 8.0 7.5 7.0 6.5 6.0 5.5 5.0 4.5 4.0 3.5 3.0 2.5 2.0 1.5 1.0 0.5 0.0
f1 (ppm)

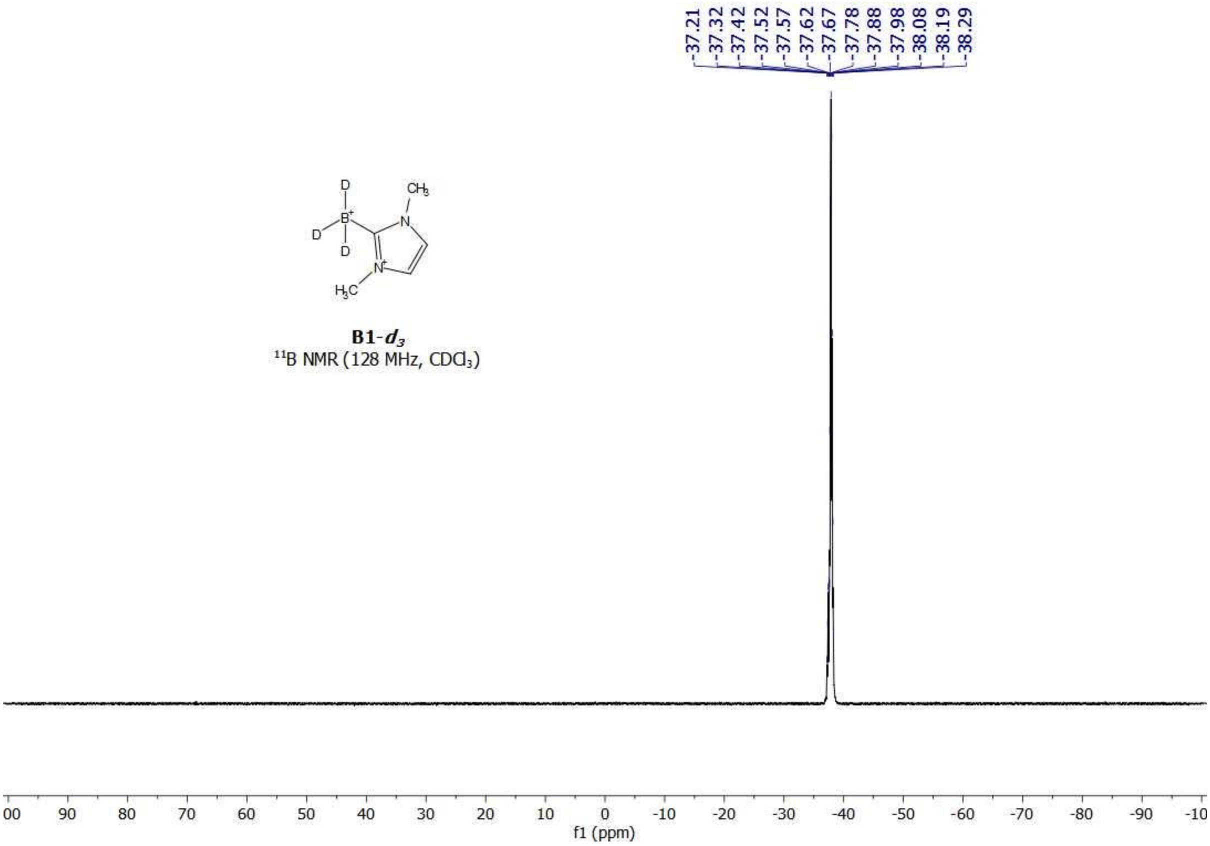

B1-*d*$_3$
^{11}B NMR (128 MHz, $CDCl_3$)

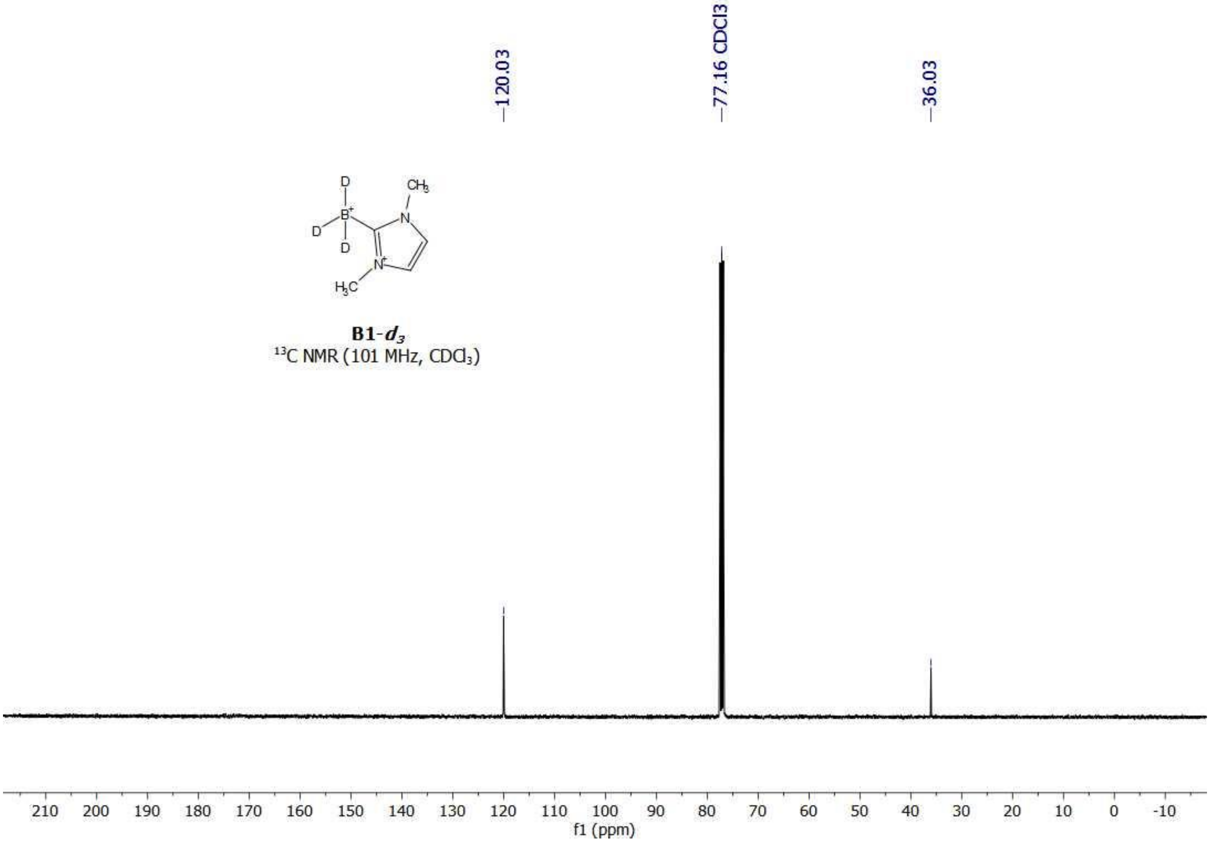

B1-*d*$_3$
^{13}C NMR (101 MHz, $CDCl_3$)

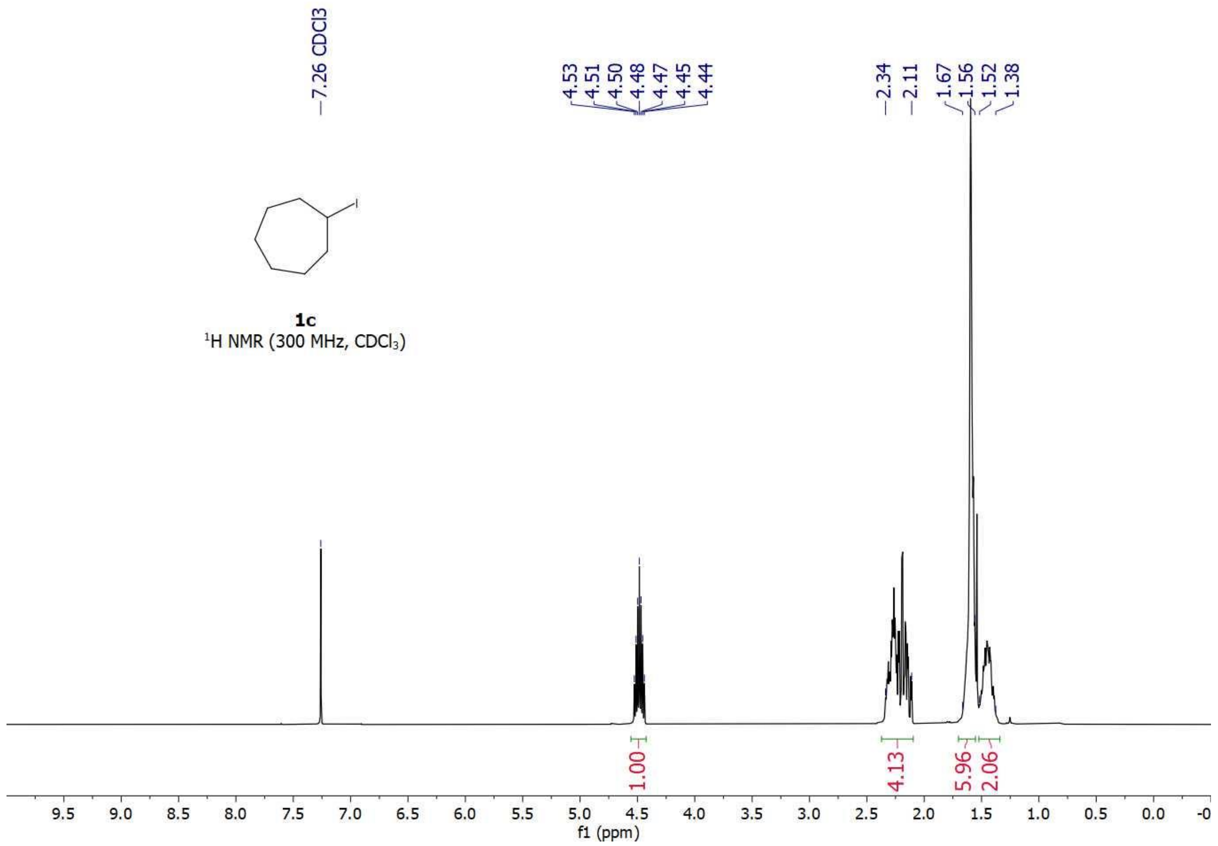
7.26 CDCl3
4.53
4.51
4.50
4.48
4.47
4.45
4.44
2.34
2.11
1.67
1.56
1.52
1.38
1c
^{1}H NMR (300 MHz, $CDCl_3$)
1.00
4.13
5.96
2.06
9.5 9.0 8.5 8.0 7.5 7.0 6.5 6.0 5.5 5.0 4.5 4.0 3.5 3.0 2.5 2.0 1.5 1.0 0.5 0.0 -0
f1 (ppm)

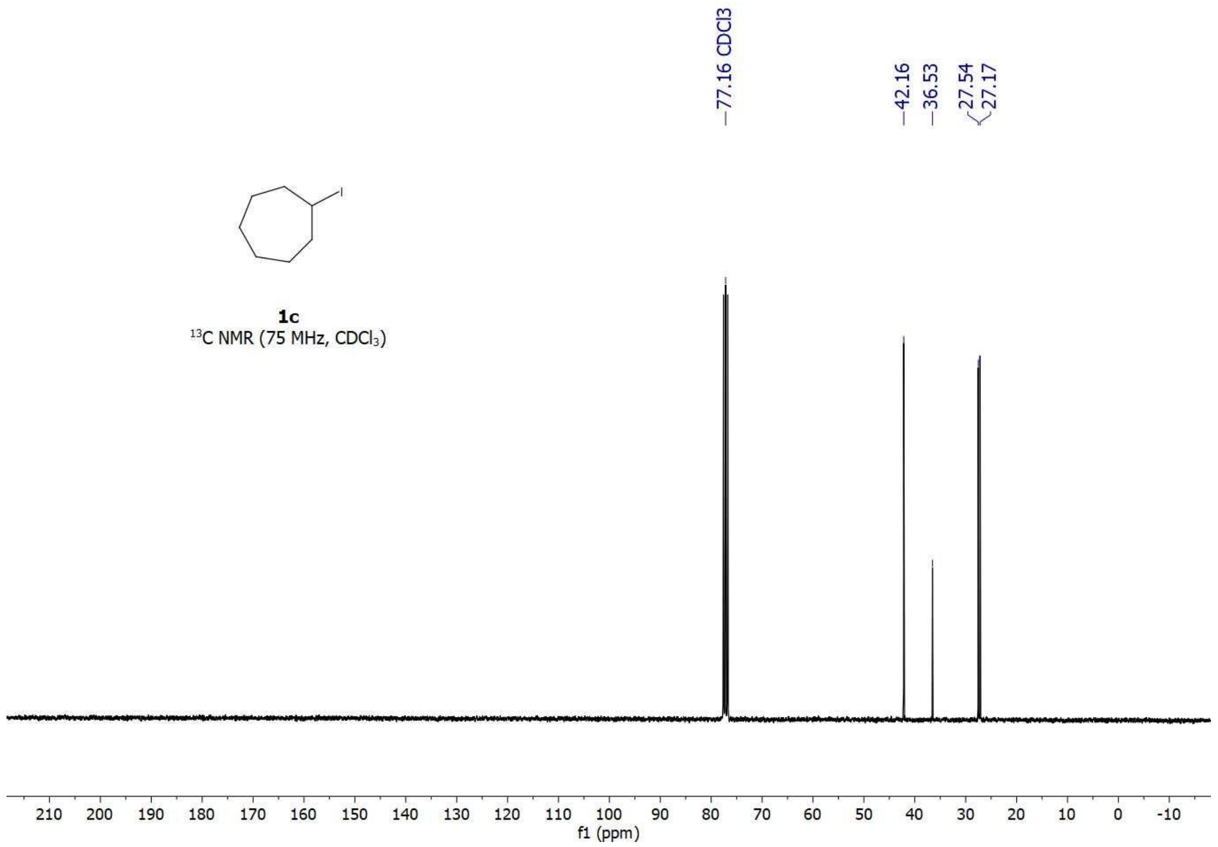
77.16 CDCl3
42.16
36.53
27.54
27.17
1c
^{13}C NMR (75 MHz, $CDCl_3$)
210 200 190 180 170 160 150 140 130 120 110 100 90 80 70 60 50 40 30 20 10 0 -10
f1 (ppm)

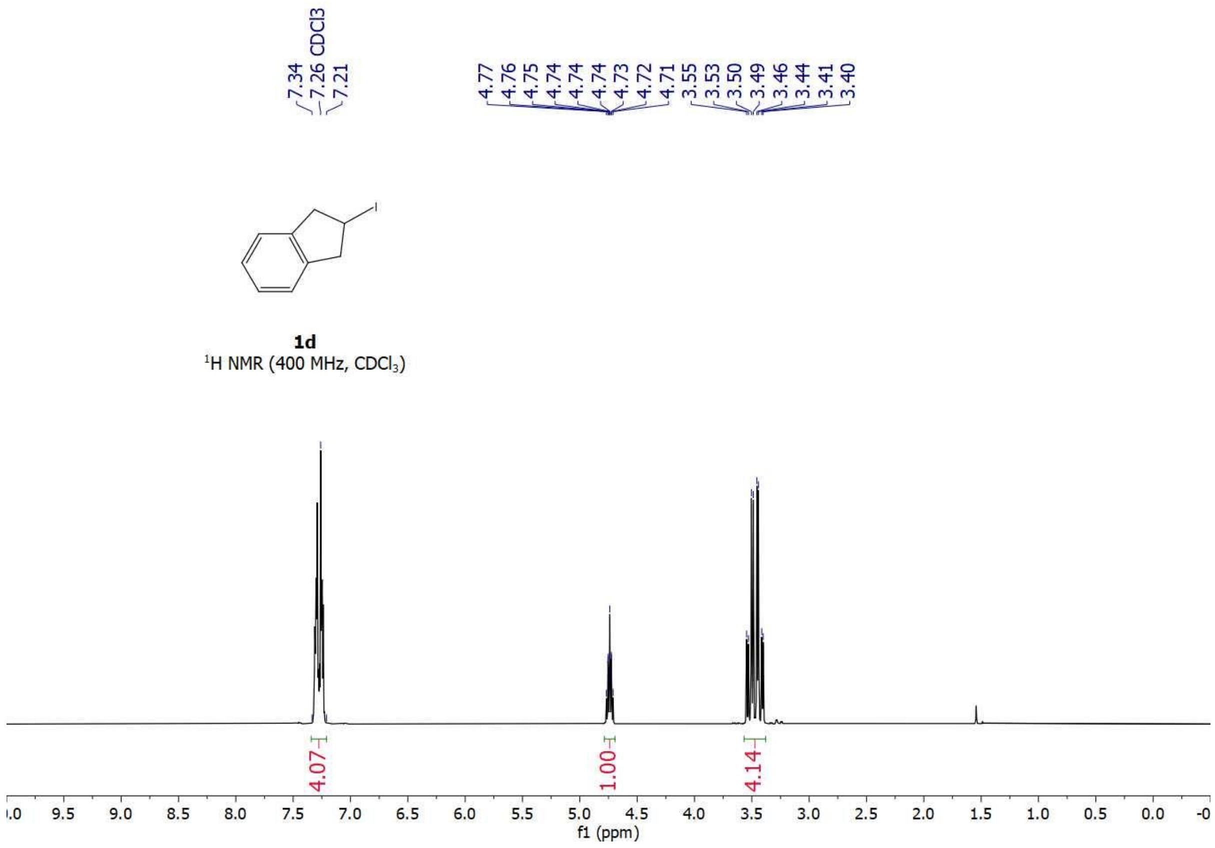
7.34
7.26 CDCl3
7.21
4.77
4.76
4.75
4.74
4.74
4.74
4.73
4.72
4.71
3.55
3.53
3.50
3.49
3.46
3.44
3.41
3.40
I
1d
1H NMR (400 MHz, CDCl3)
4.07
1.00
4.14
f1 (ppm)

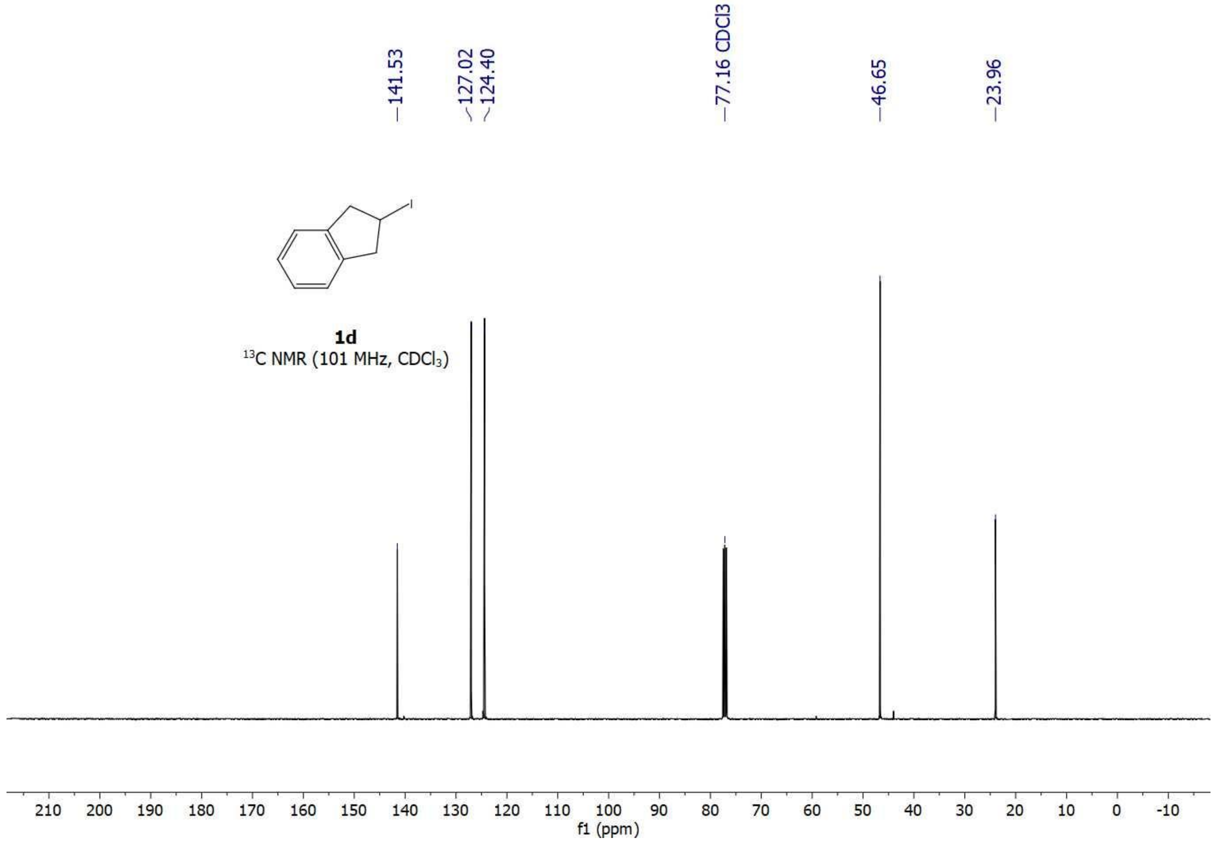
141.53
127.02
124.40
77.16 CDCl3
46.65
23.96
I
1d
13C NMR (101 MHz, CDCl3)
f1 (ppm)

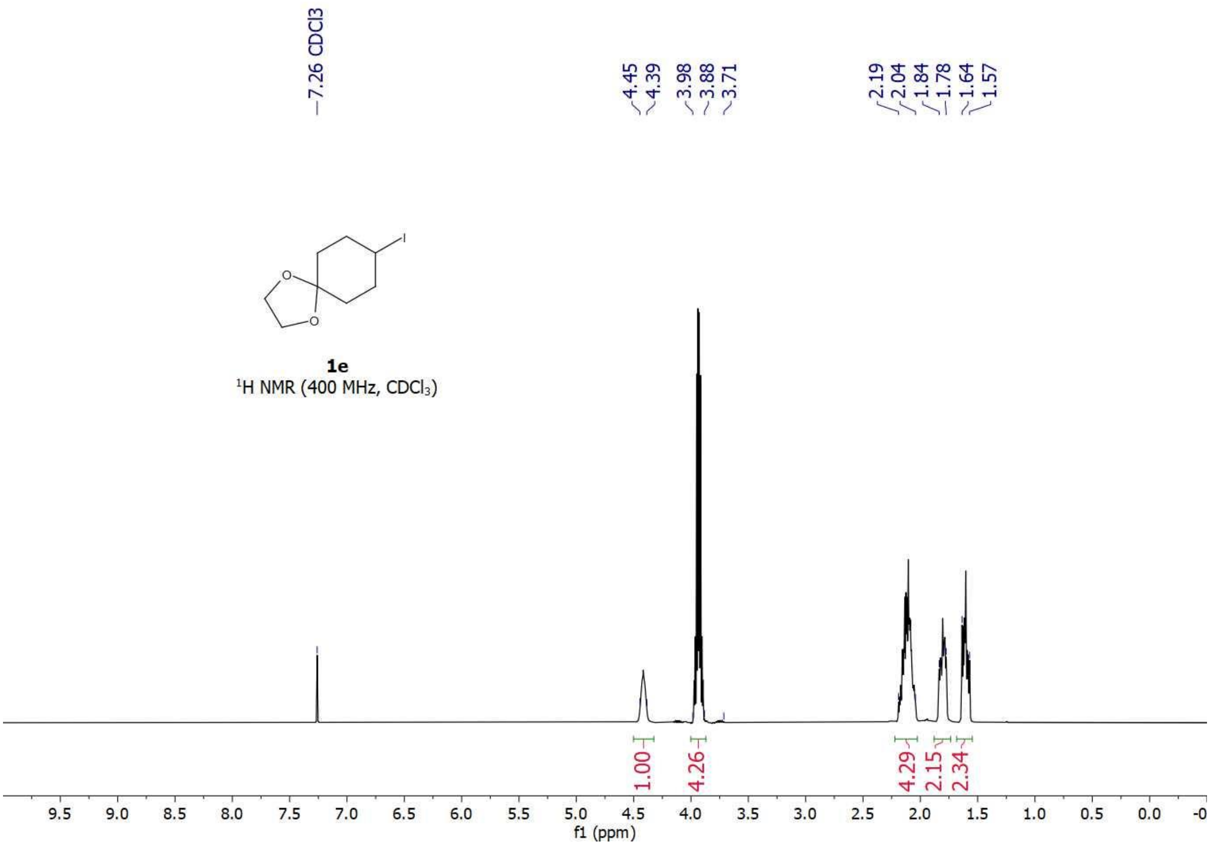

1e
^1H NMR (400 MHz, $CDCl_3$)

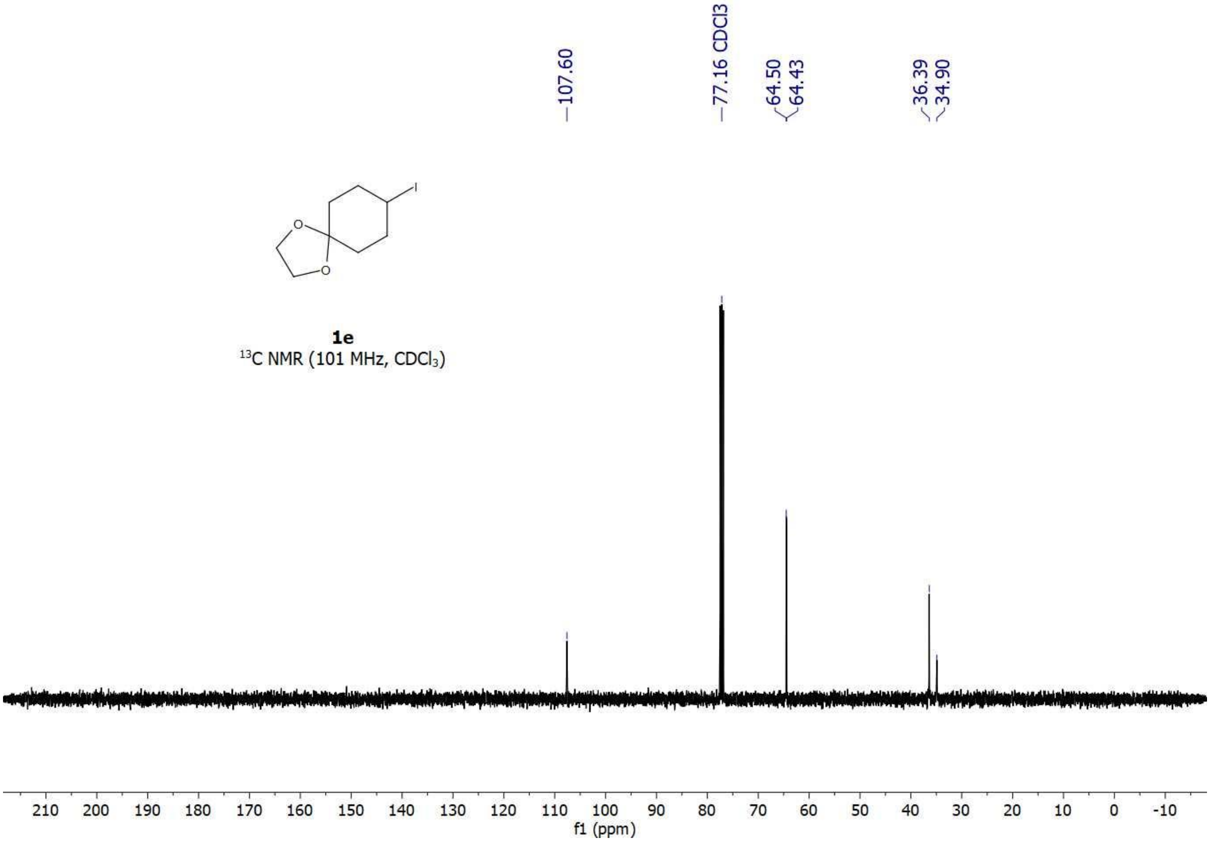

1e
^{13}C NMR (101 MHz, $CDCl_3$)

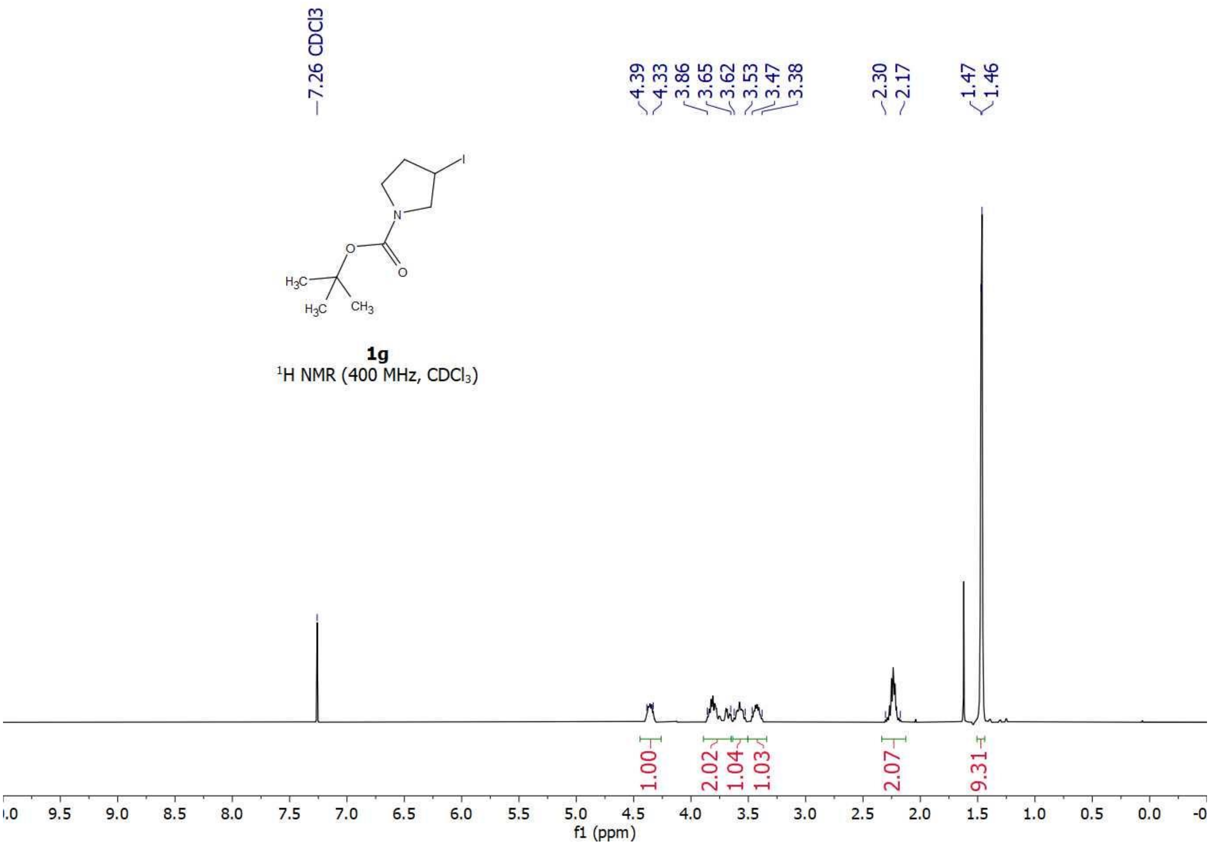
7.26 CDCl3
4.39
4.33
3.86
3.65
3.62
3.53
3.47
3.38
2.30
2.17
1.47
1.46
1g
1H NMR (400 MHz, CDCl3)
1.00
2.02
1.04
1.03
2.07
9.31
f1 (ppm)

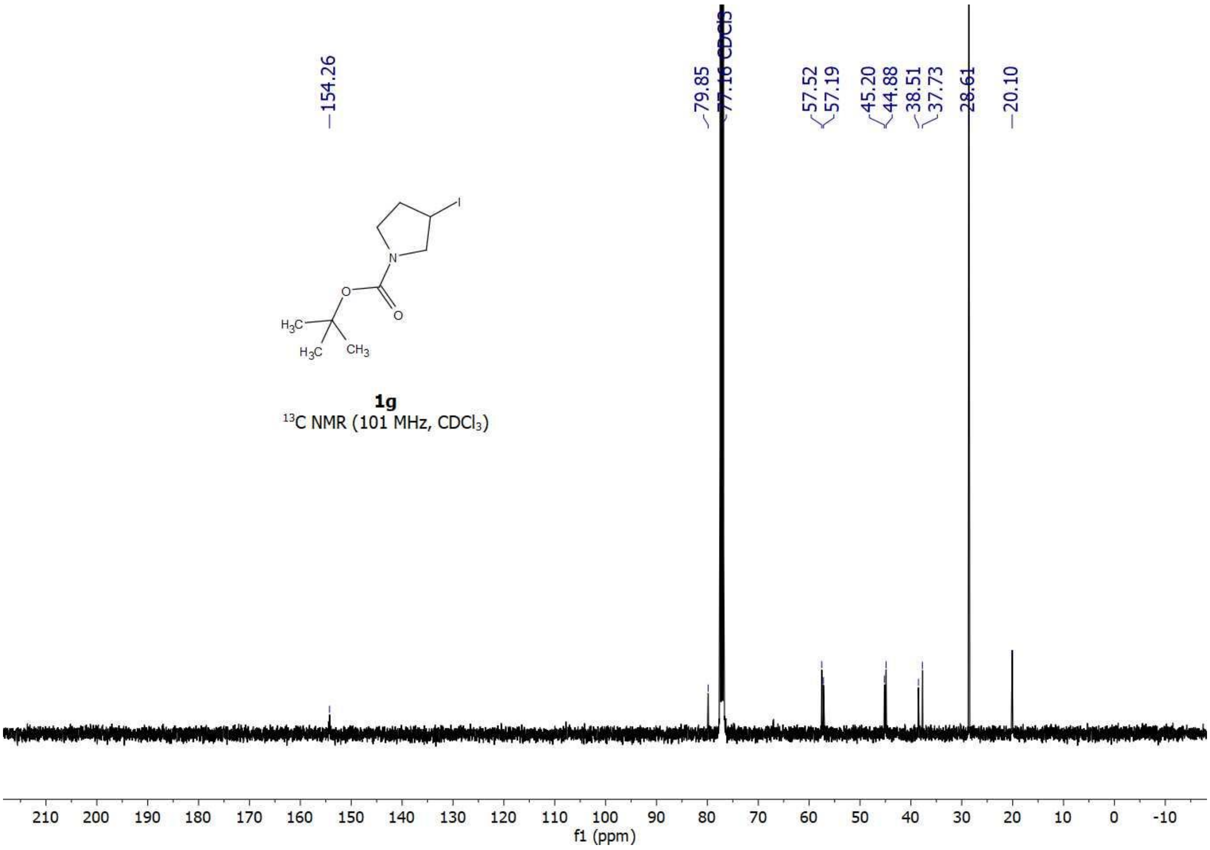
154.26
79.85
77.16 CDCl3
57.52
57.19
45.20
44.88
38.51
37.73
28.61
20.10
1g
13C NMR (101 MHz, CDCl3)
f1 (ppm)

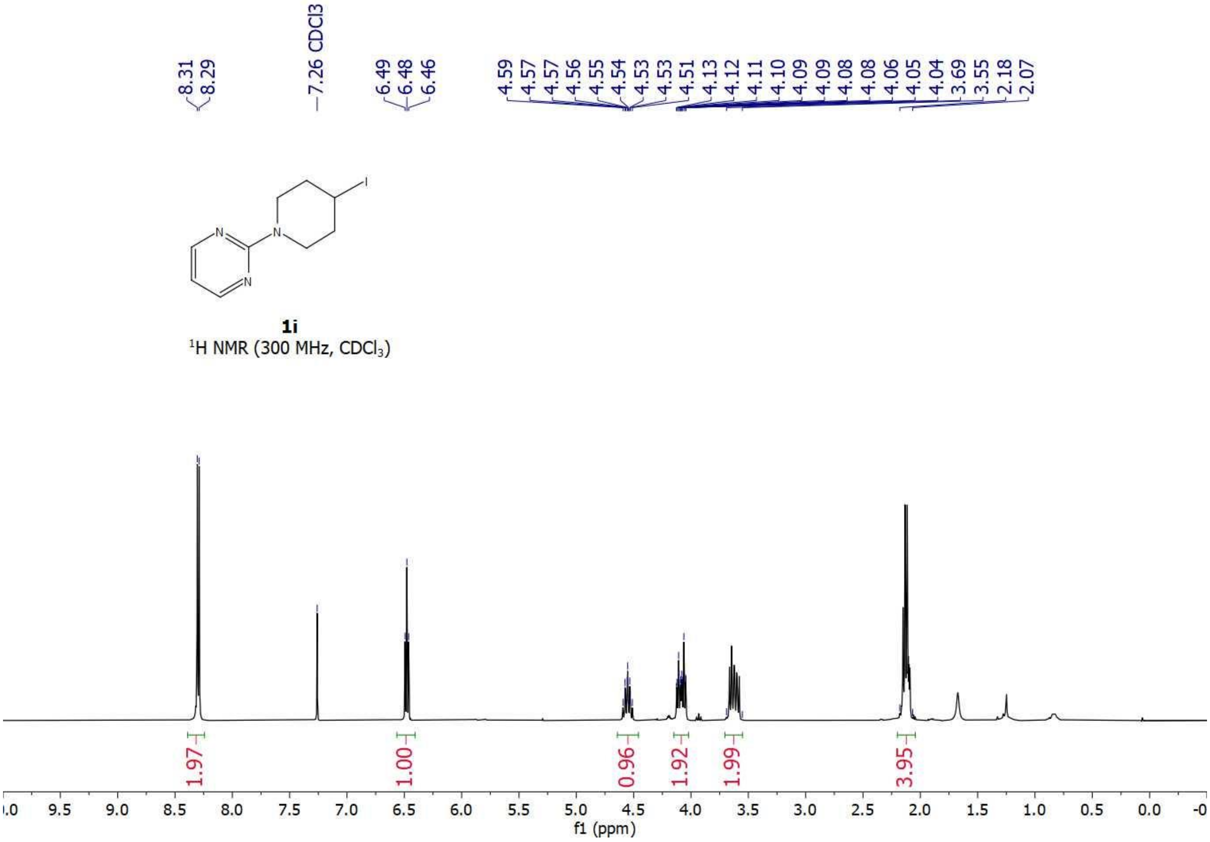

1i

^{1}H NMR (300 MHz, $CDCl_3$)

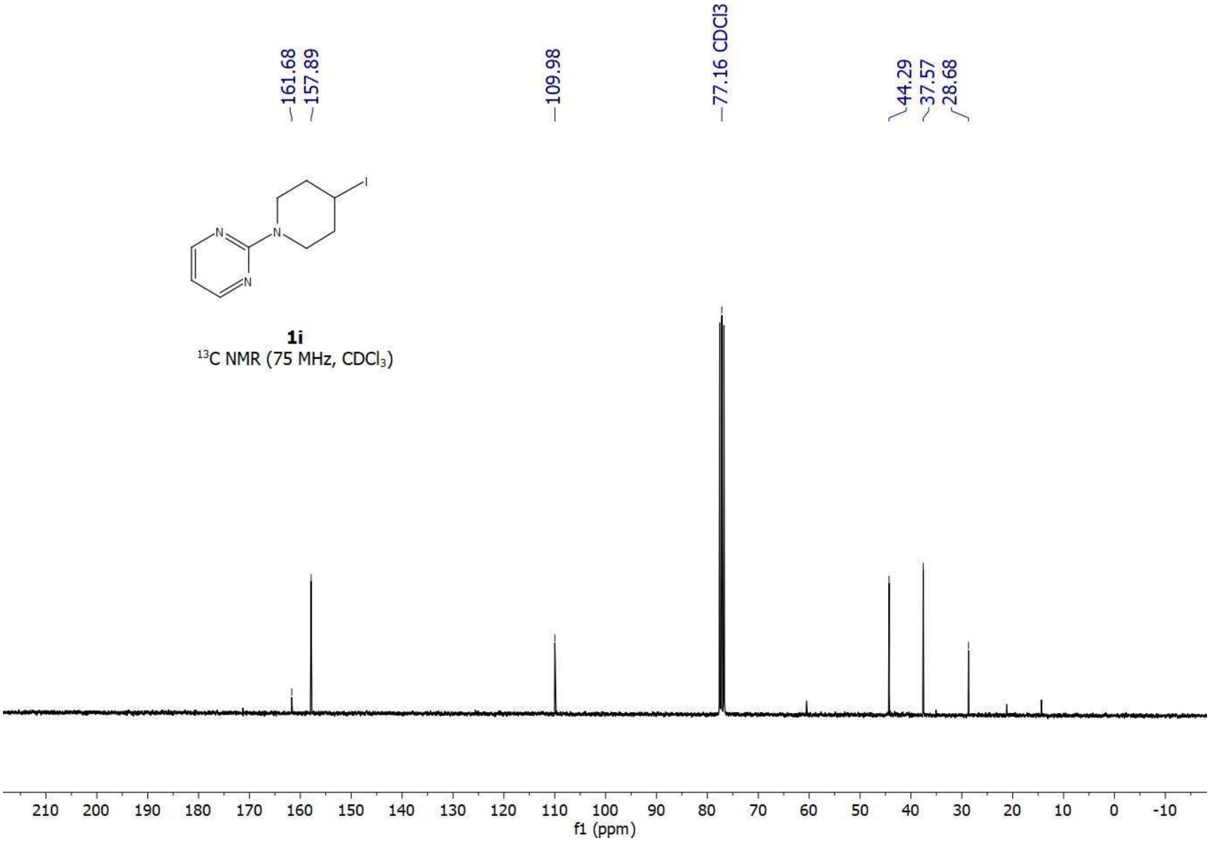

1i

^{13}C NMR (75 MHz, $CDCl_3$)

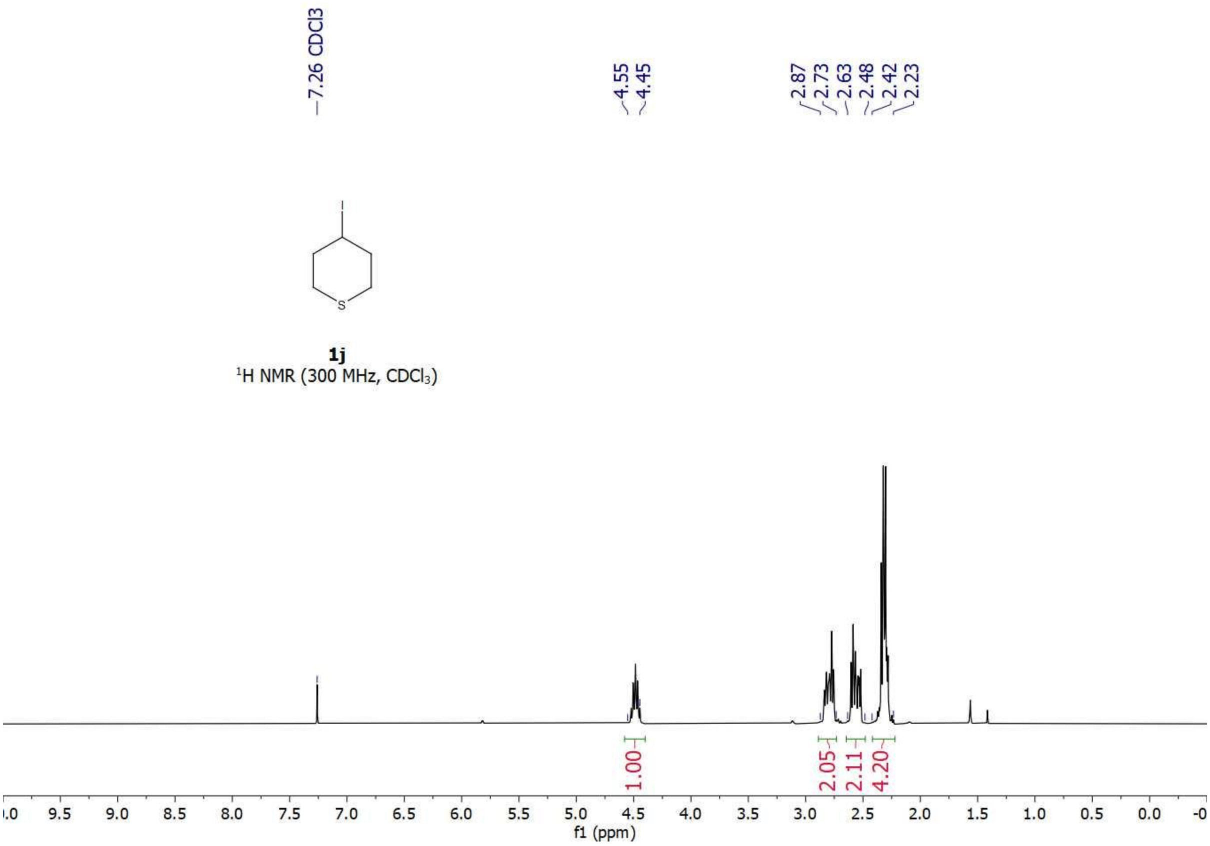

7.26 CDCl3
4.55
4.45
2.87
2.73
2.63
2.48
2.42
2.23
I
S
1j
1H NMR (300 MHz, CDCl3)
1.00
2.05
2.11
4.20
f1 (ppm)

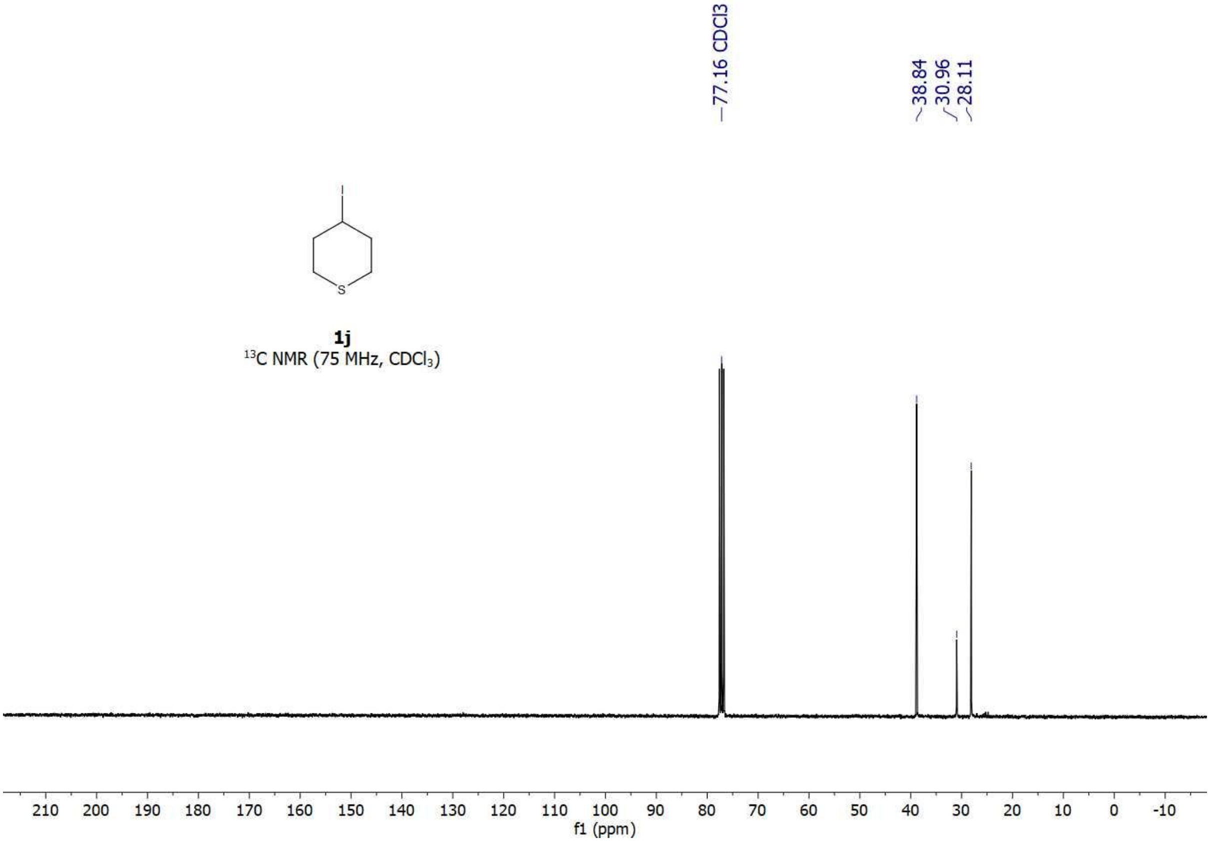

77.16 CDCl3
38.84
30.96
28.11
I
S
1j
13C NMR (75 MHz, CDCl3)
f1 (ppm)

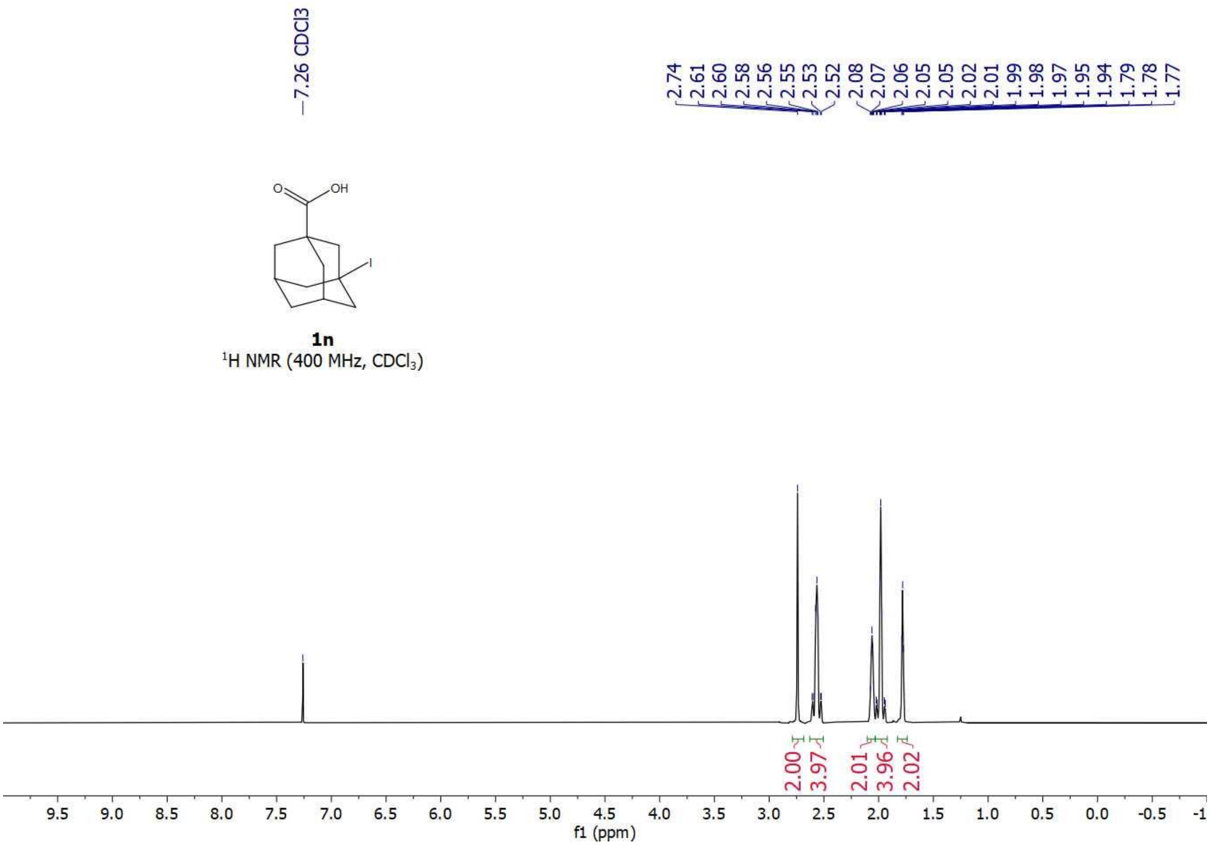

1n
^{1}H NMR (400 MHz, $CDCl_3$)

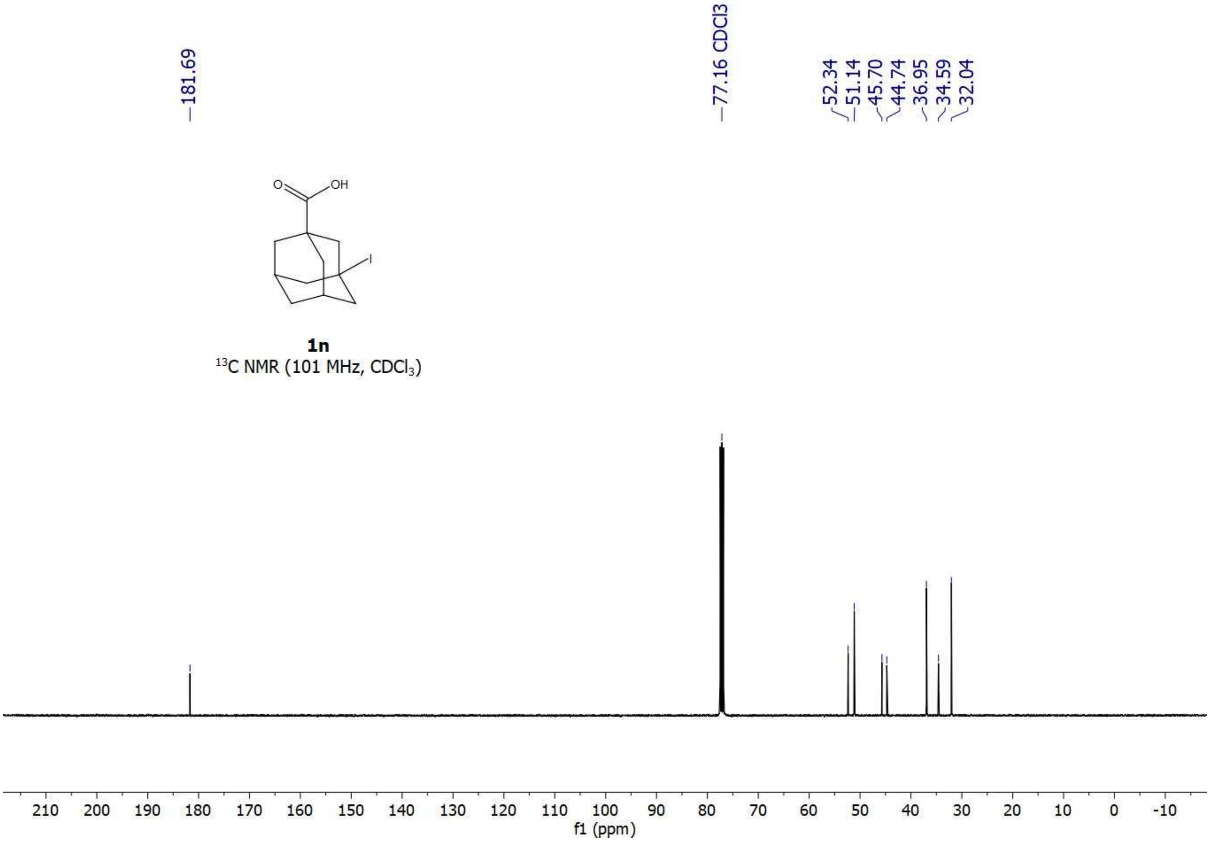

1n
^{13}C NMR (101 MHz, $CDCl_3$)

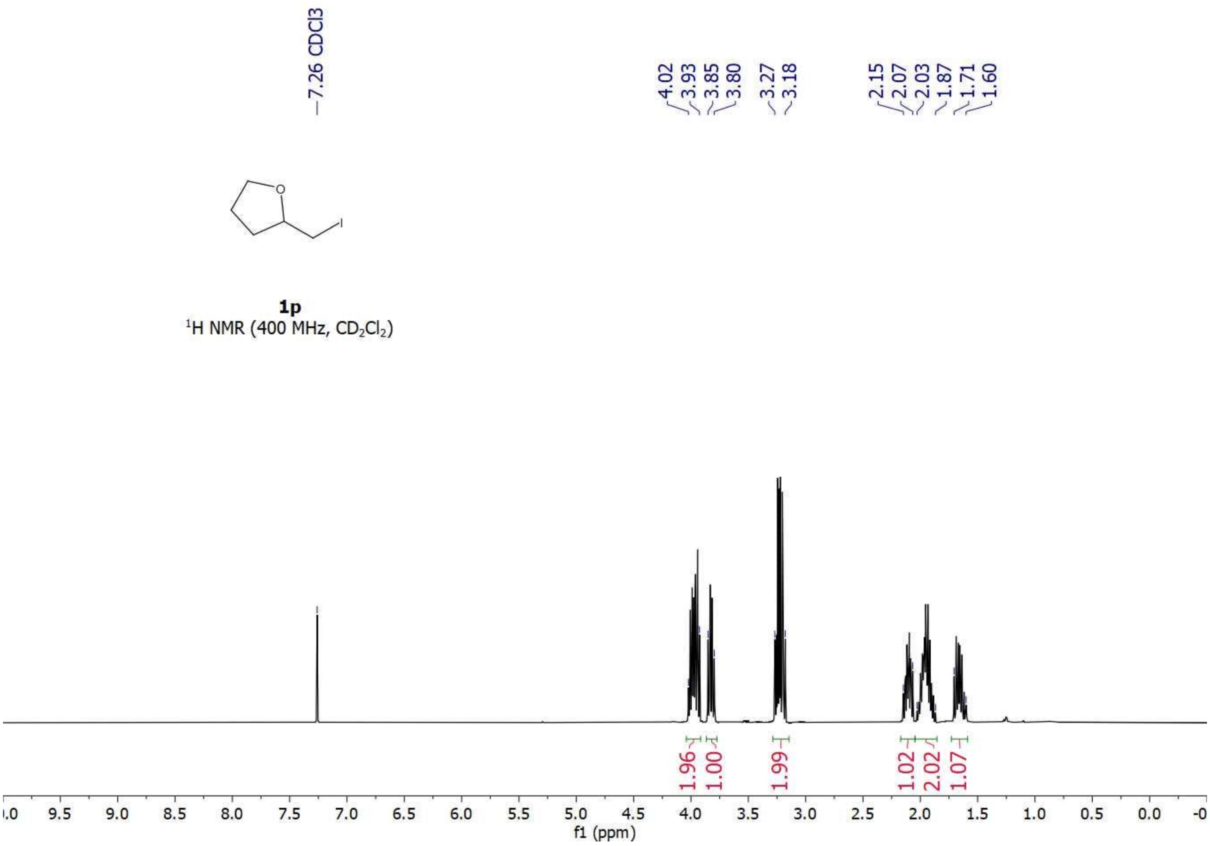

1p
^{1}H NMR (400 MHz, CD_2Cl_2)

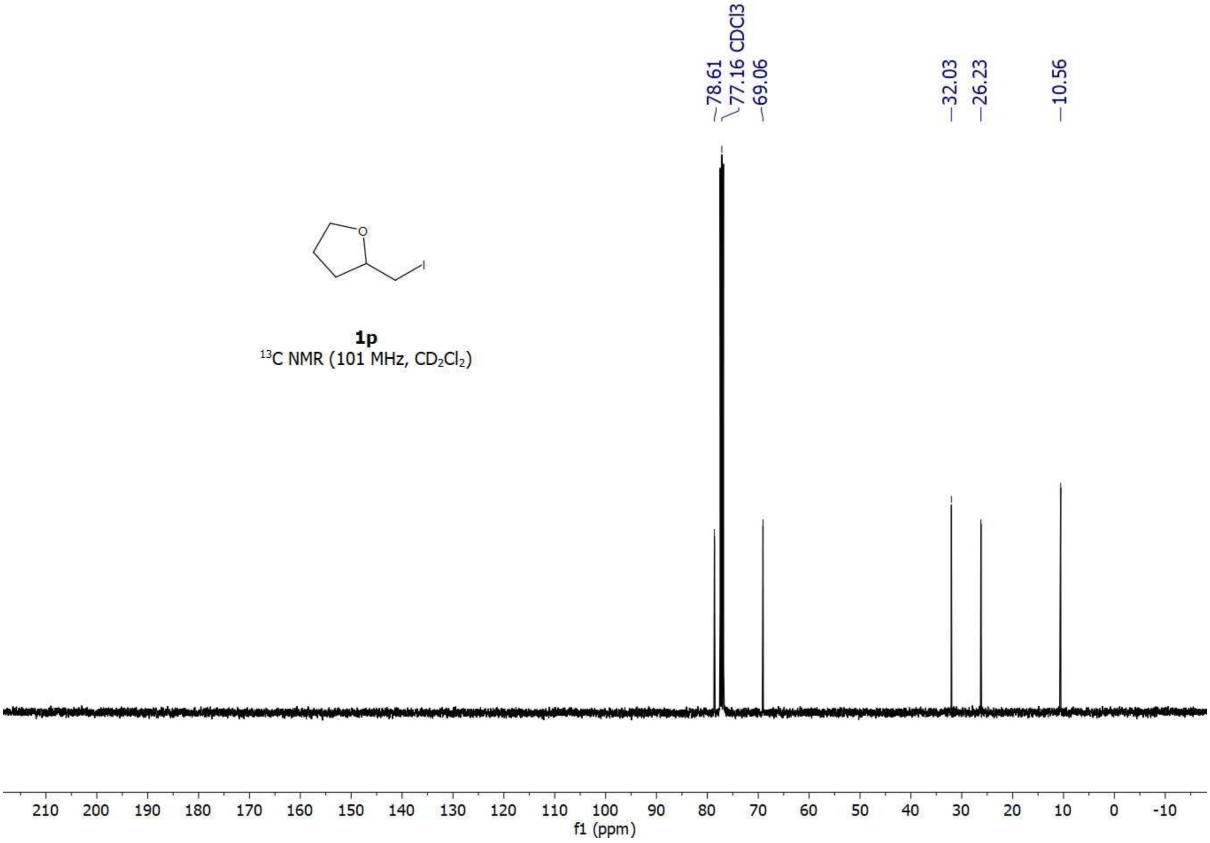

1p
^{13}C NMR (101 MHz, CD_2Cl_2)

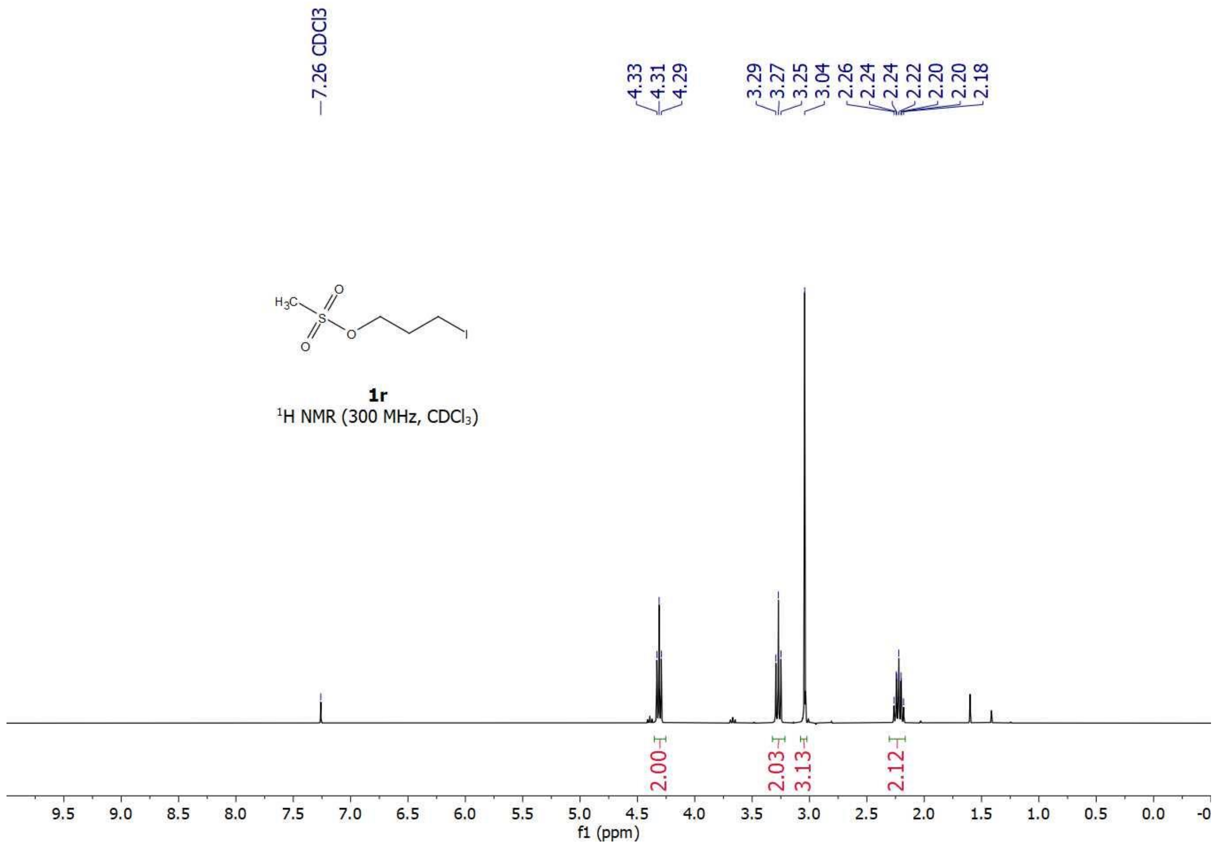
7.26 CDCl3
4.33
4.31
4.29
3.29
3.27
3.25
3.04
2.26
2.24
2.24
2.22
2.20
2.20
2.18
1r
1H NMR (300 MHz, CDCl3)
2.00
2.03
3.13
2.12
f1 (ppm)

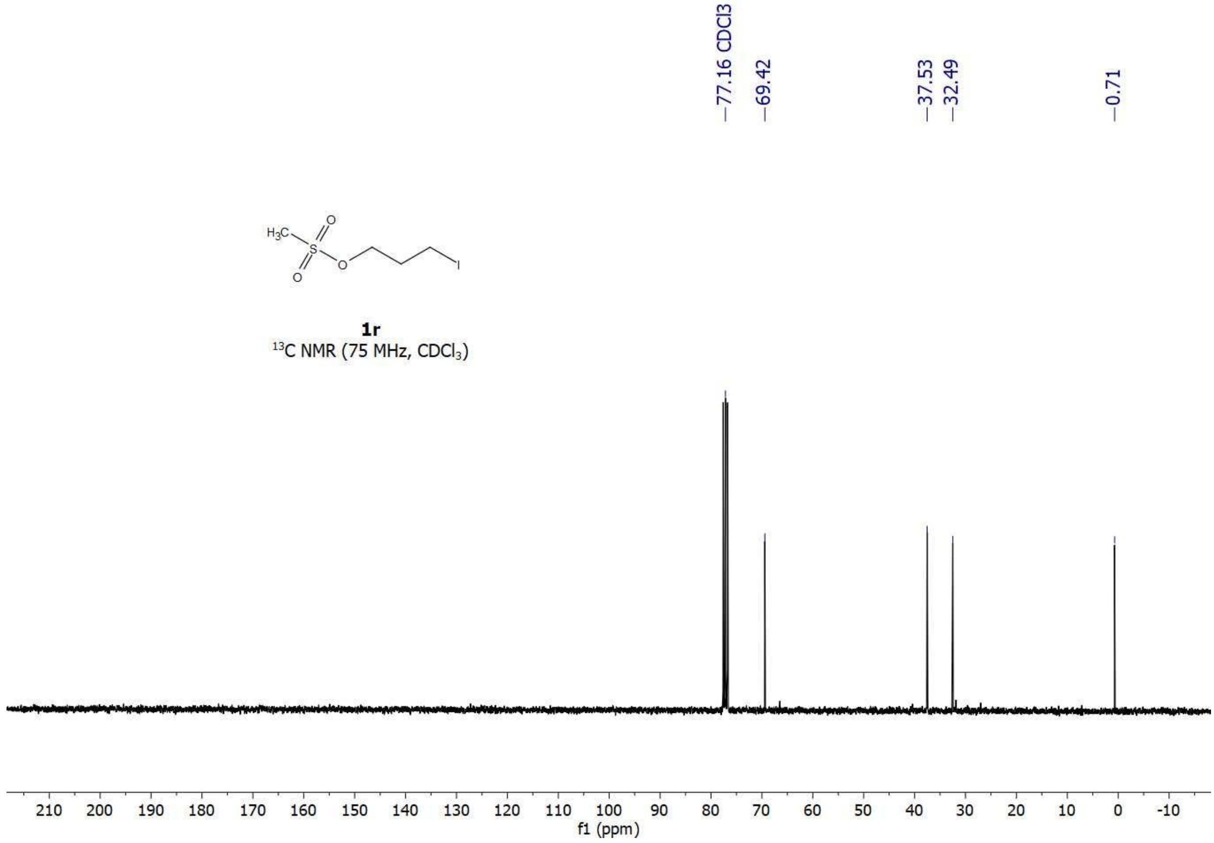
77.16 CDCl3
69.42
37.53
32.49
0.71
1r
13C NMR (75 MHz, CDCl3)
f1 (ppm)

1s
^{1}H NMR (400 MHz, $CDCl_3$)

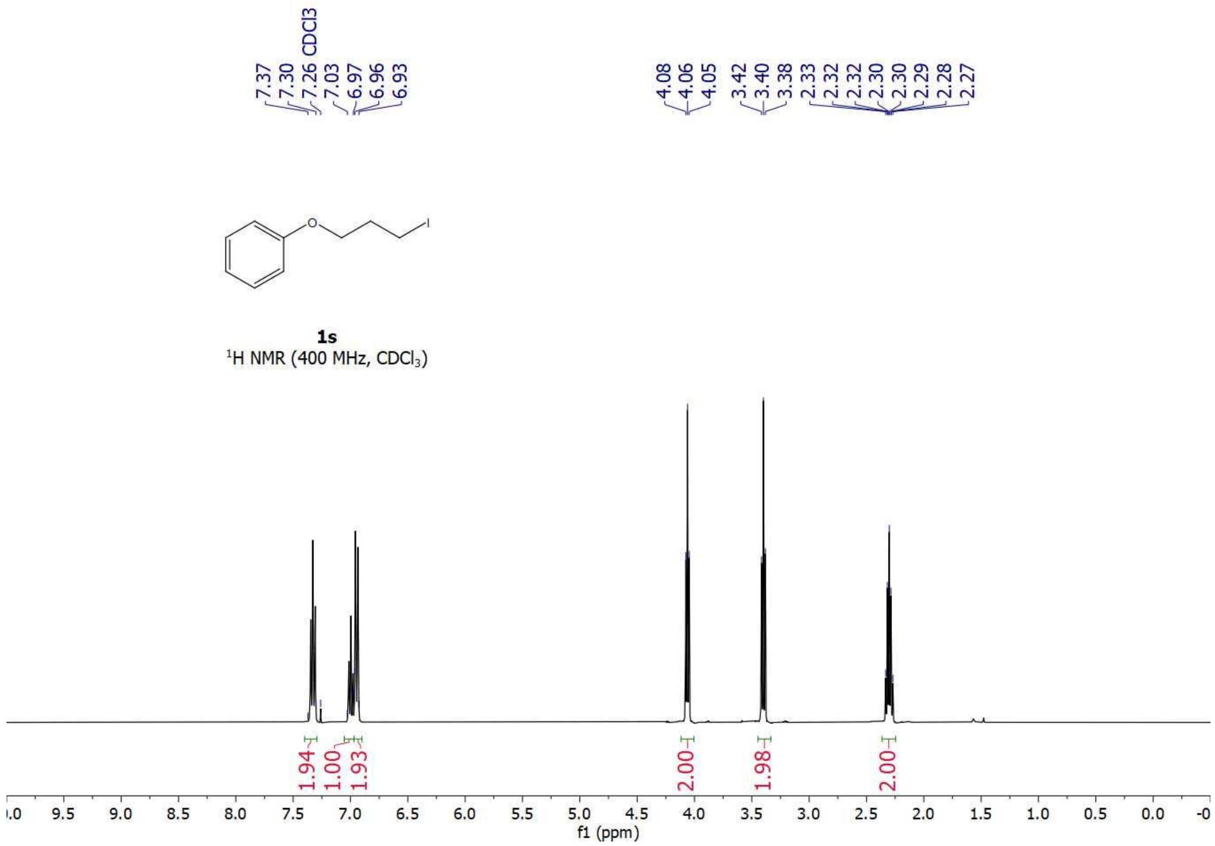

1s
^{13}C NMR (101 MHz, $CDCl_3$)

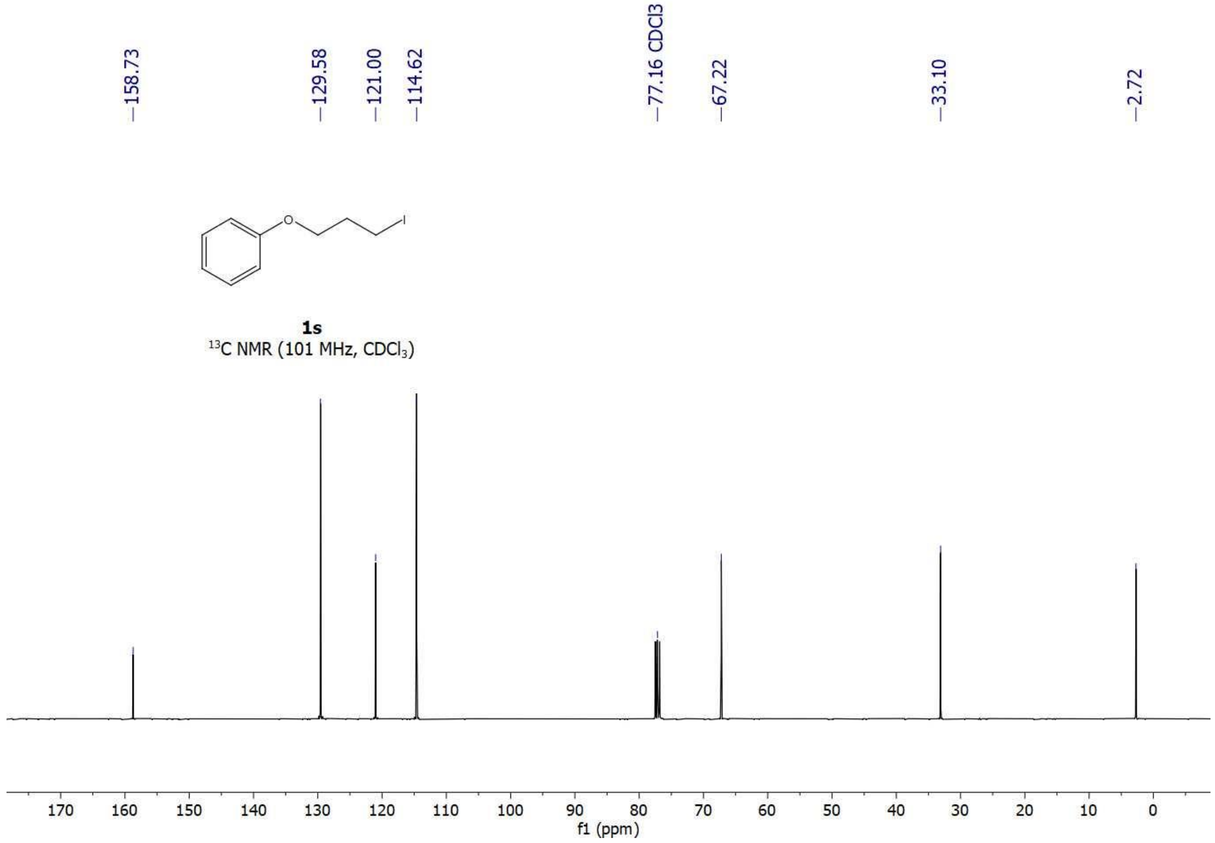

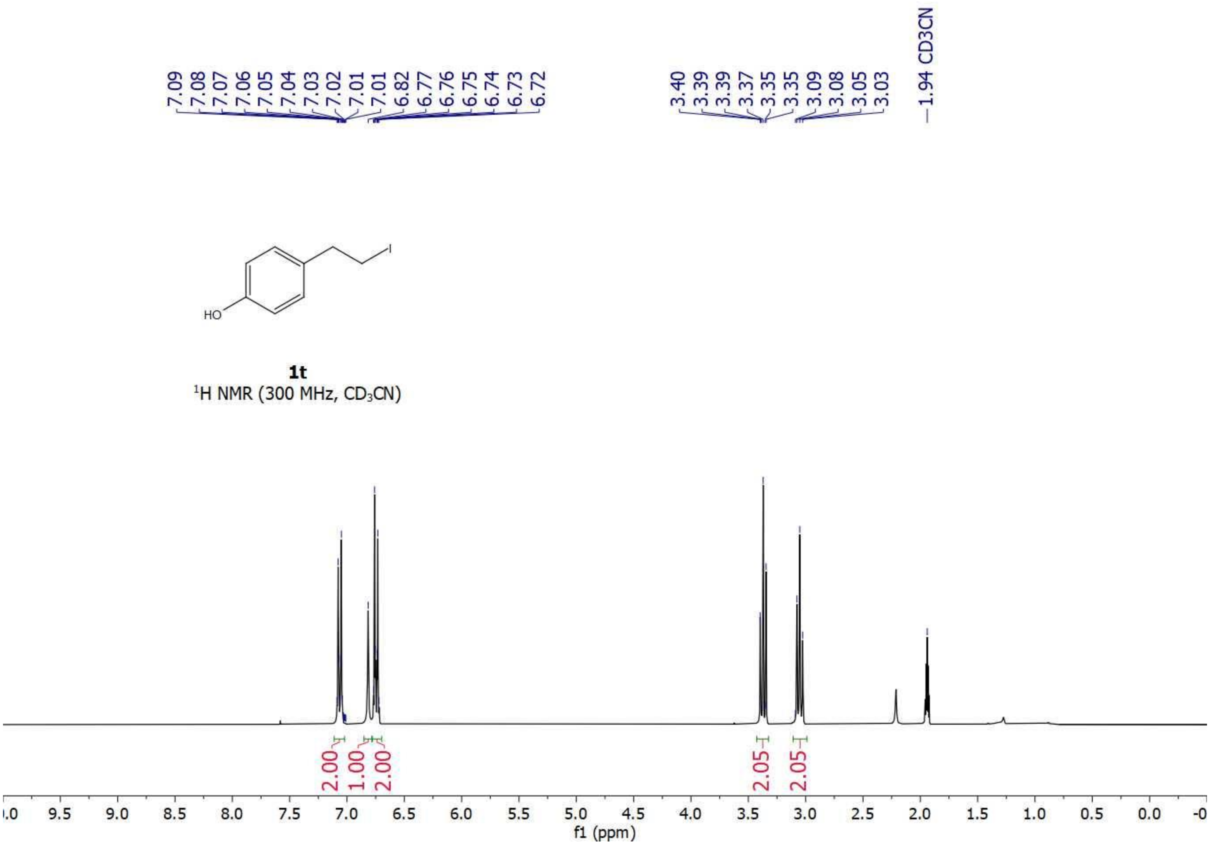
7.09 7.08 7.07 7.06 7.05 7.04 7.03 7.02 7.01 6.82 6.77 6.76 6.75 6.74 6.73 6.72
3.40 3.39 3.39 3.37 3.35 3.35 3.09 3.08 3.05 3.03
1.94 CD3CN
HO
I
1t
^{1}H NMR (300 MHz, CD_3CN)
2.00 1.00 2.00 2.05 2.05
f1 (ppm)

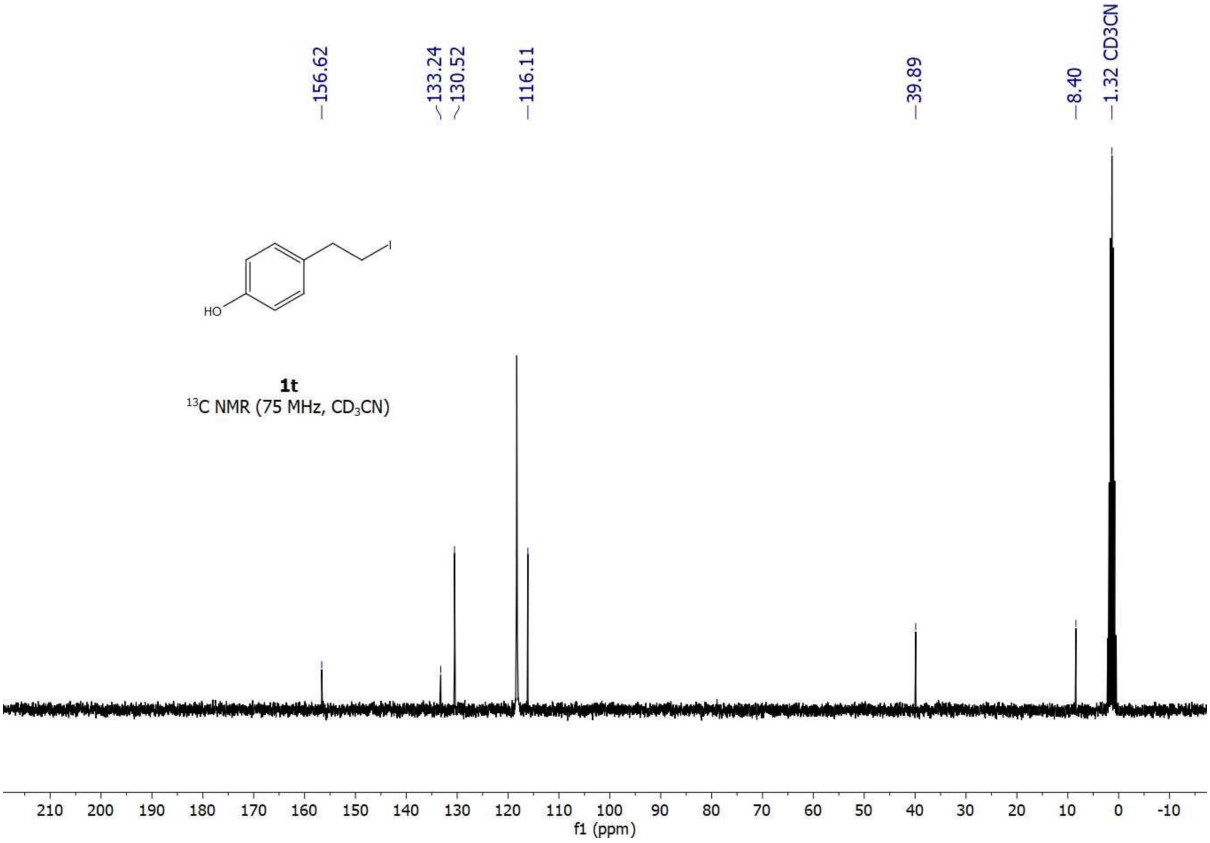
156.62
133.24 130.52
116.11
39.89
8.40
1.32 CD3CN
HO
I
1t
^{13}C NMR (75 MHz, CD_3CN)
f1 (ppm)

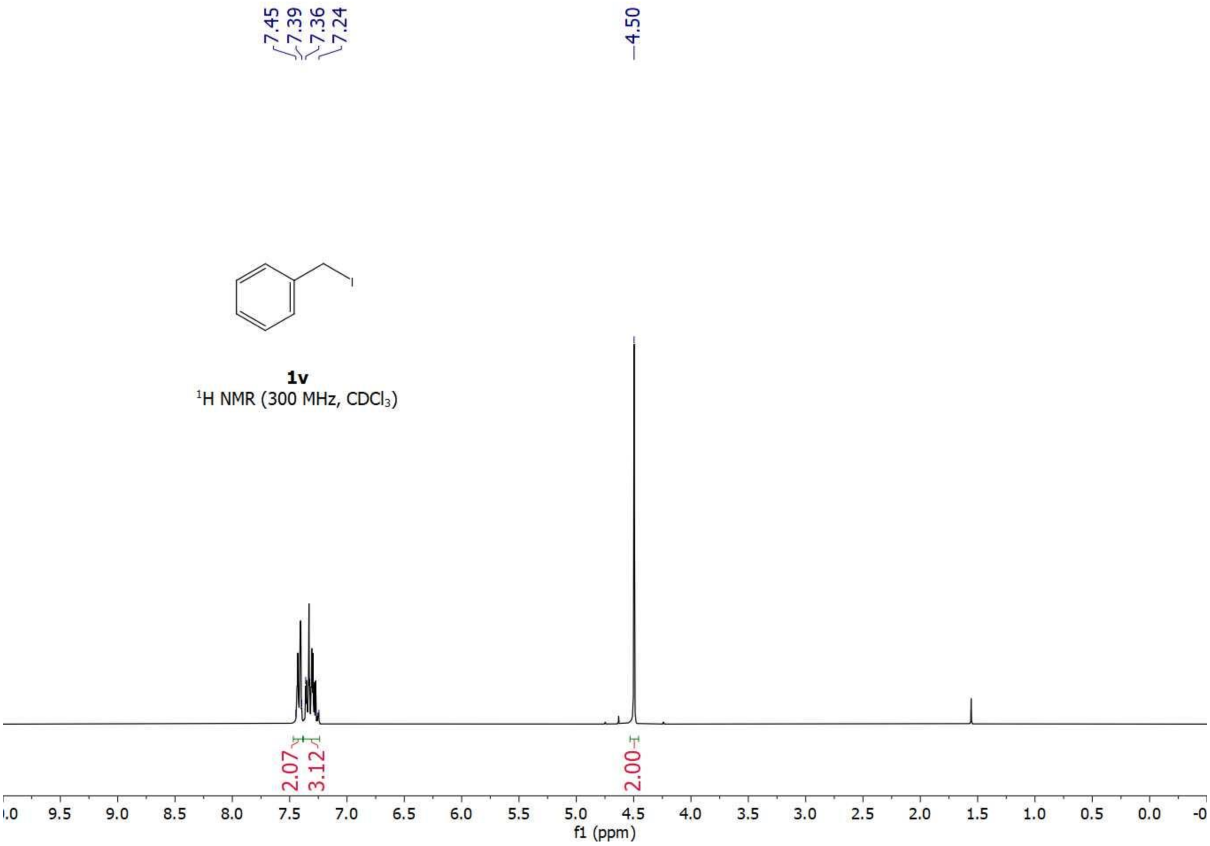
7.45
7.39
7.36
7.24
4.50
1v
^{1}H NMR (300 MHz, $CDCl_3$)
2.07
3.12
2.00
f1 (ppm)

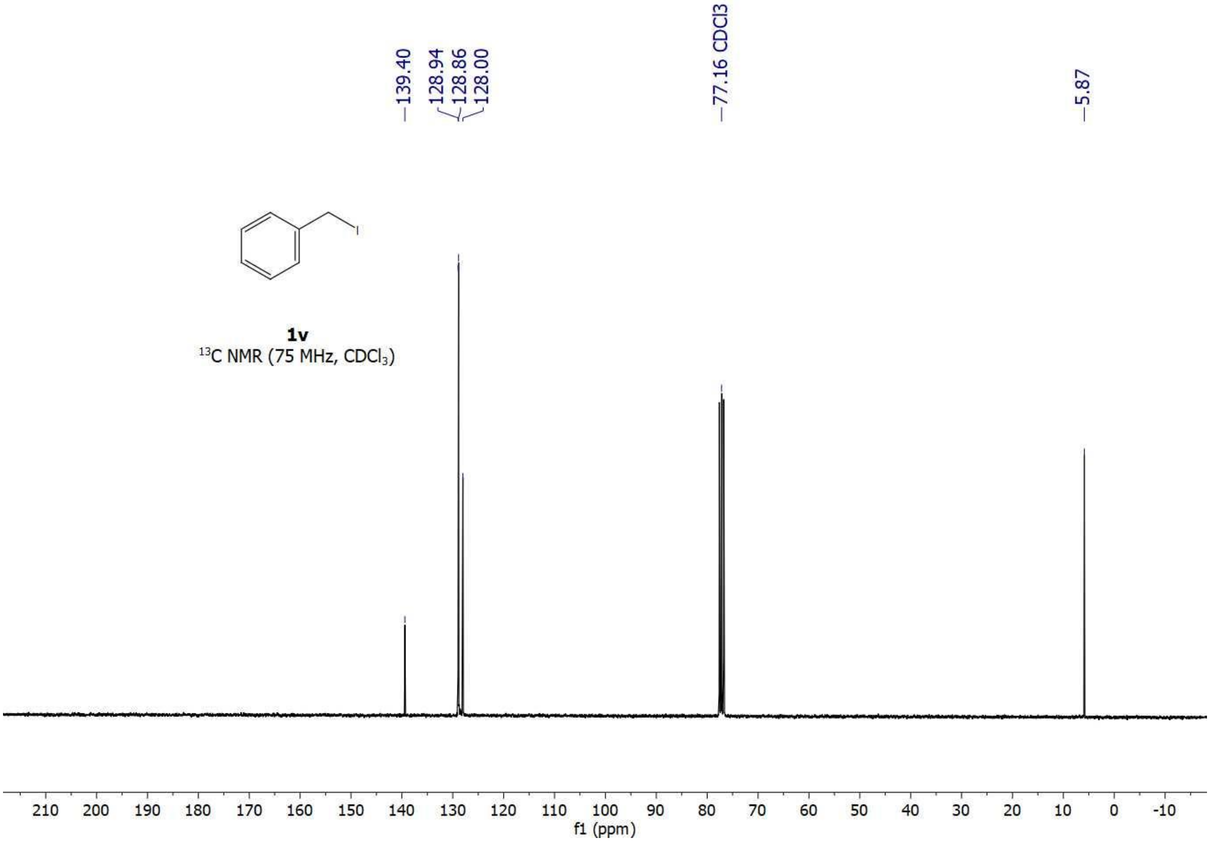
139.40
128.94
128.86
128.00
77.16 CDCl3
5.87
1v
^{13}C NMR (75 MHz, $CDCl_3$)
f1 (ppm)

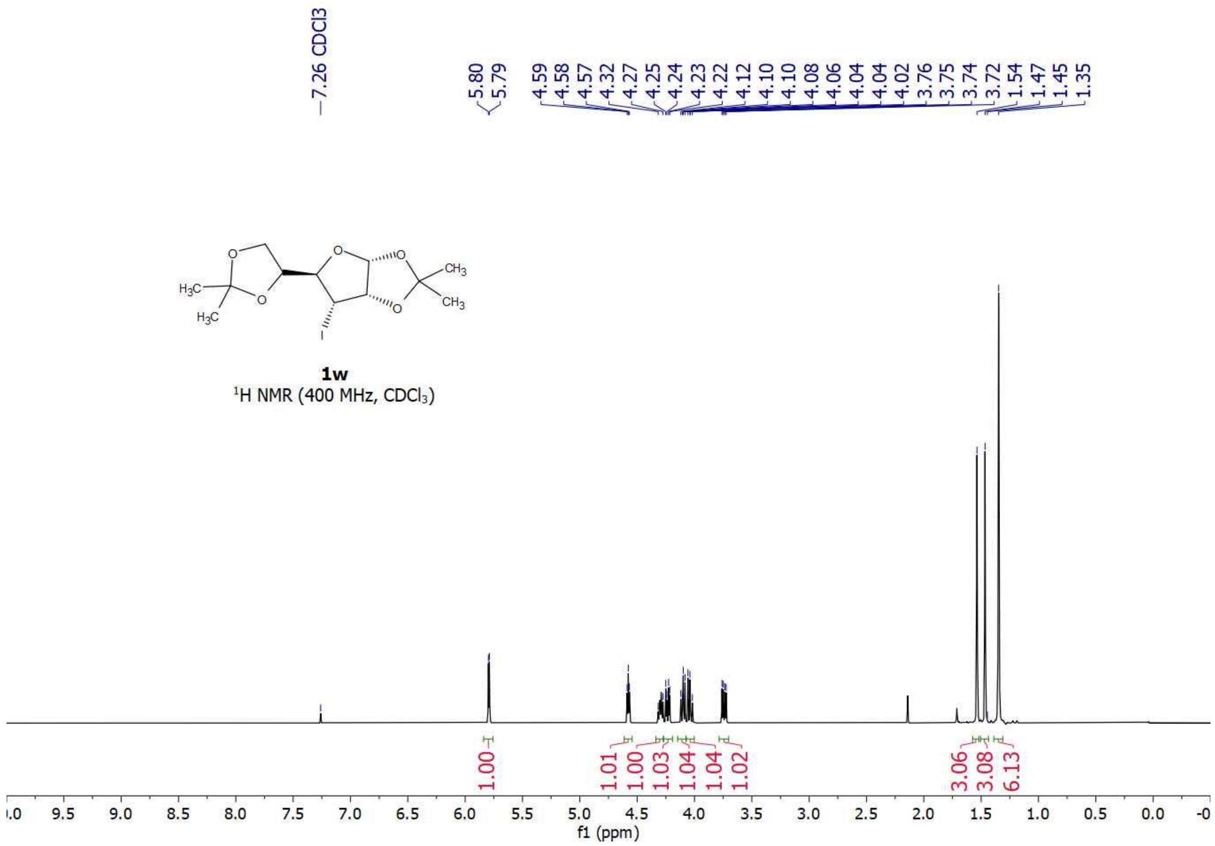

1w
^{1}H NMR (400 MHz, $CDCl_3$)

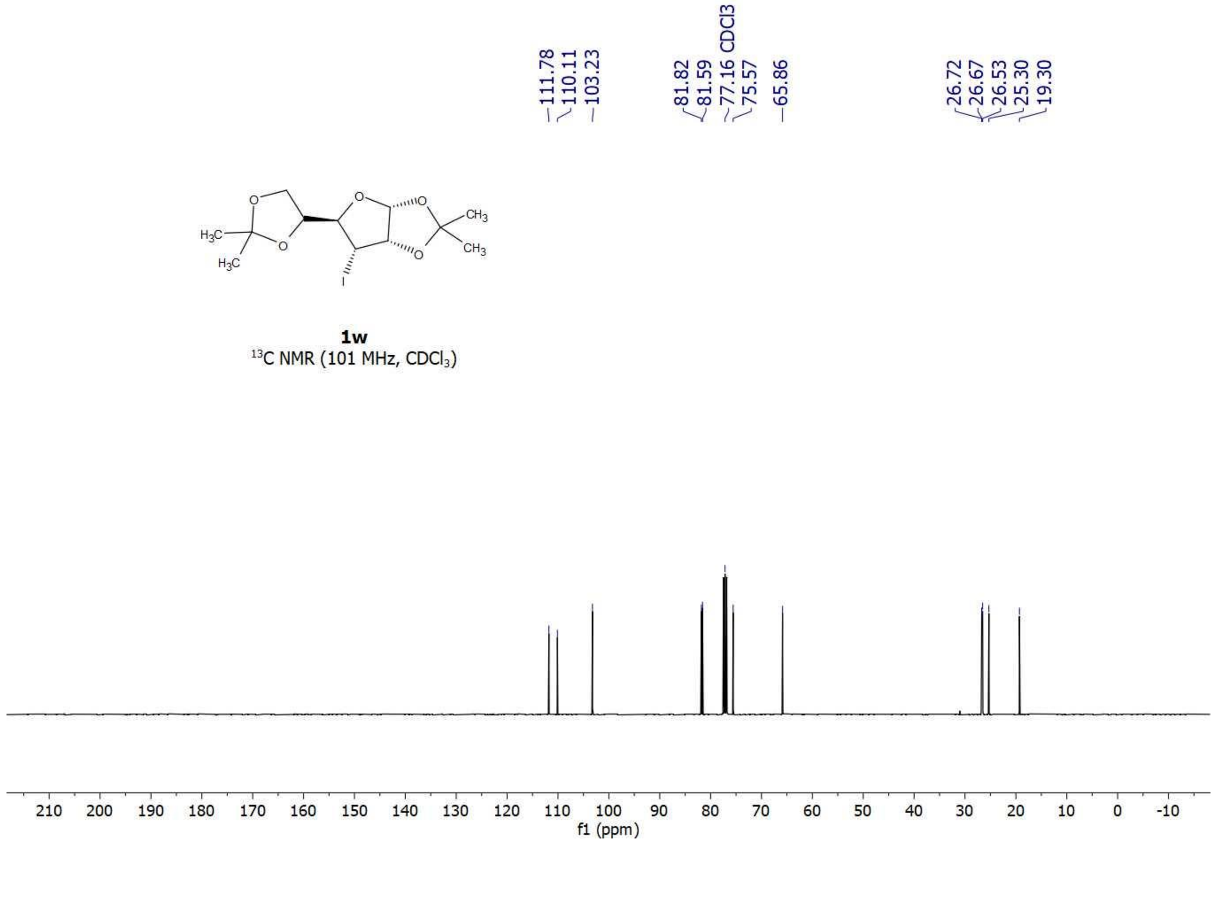

1w
^{13}C NMR (101 MHz, $CDCl_3$)

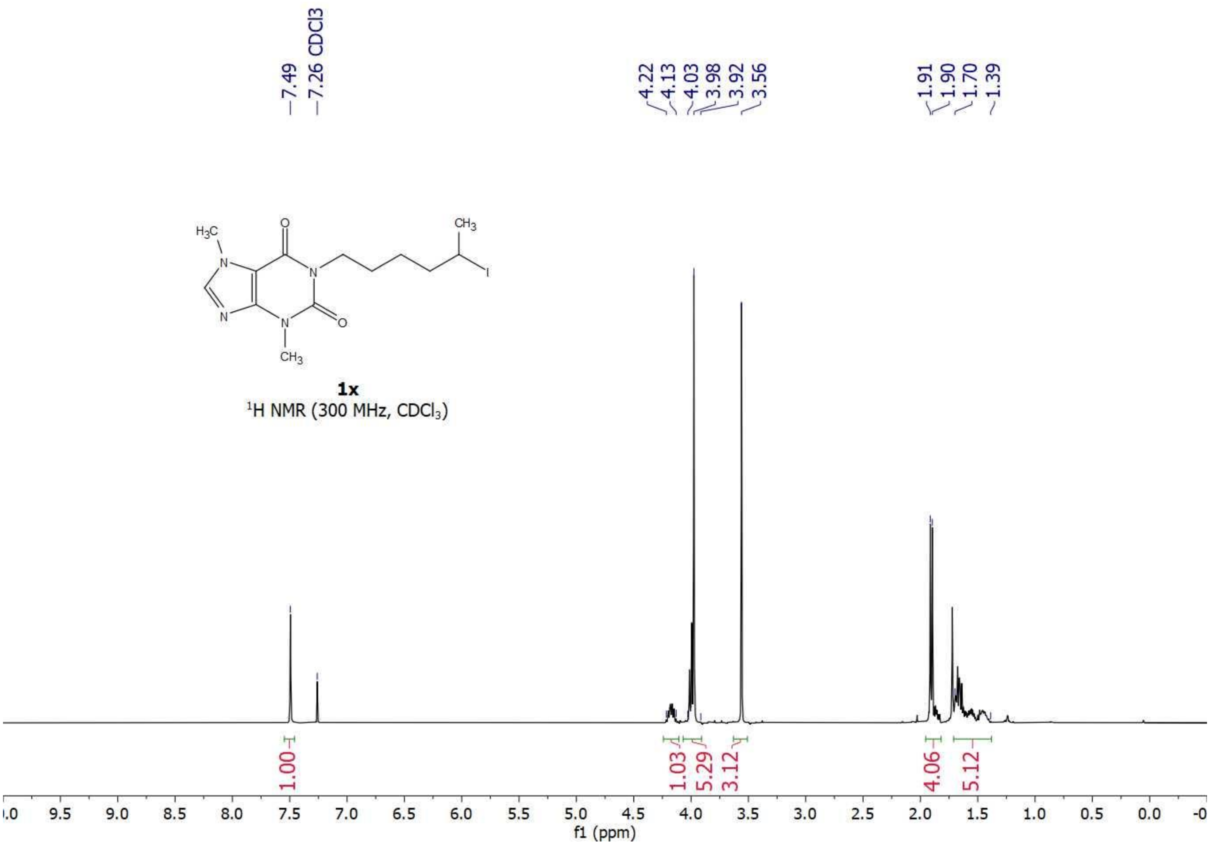
7.49
7.26 CDCl3
4.22
4.13
4.03
3.98
3.92
3.56
1.91
1.90
1.70
1.39
H_3C
CH_3
N
O
I
1x
^1H NMR (300 MHz, $CDCl_3$)
1.00
1.03
5.29
3.12
4.06
5.12
9.5 9.0 8.5 8.0 7.5 7.0 6.5 6.0 5.5 5.0 4.5 4.0 3.5 3.0 2.5 2.0 1.5 1.0 0.5 0.0
f1 (ppm)

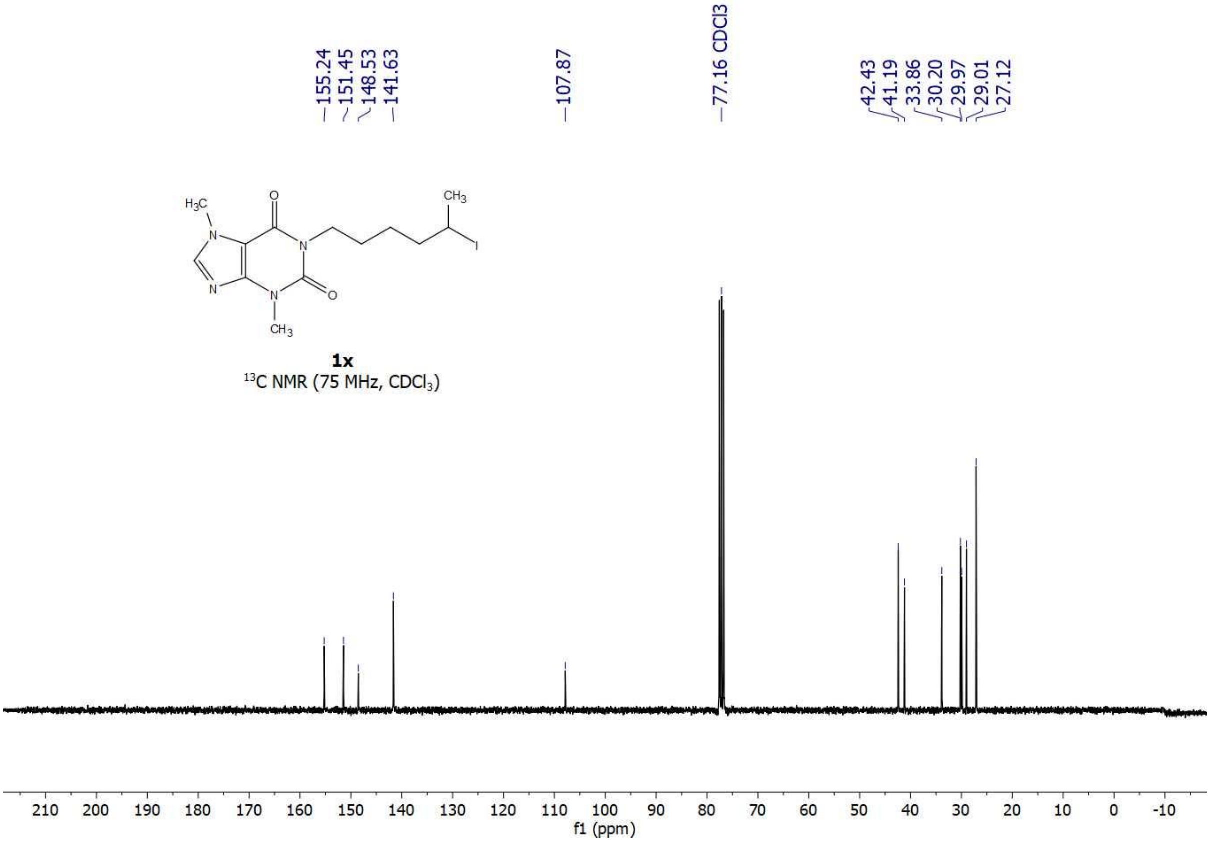
155.24
151.45
148.53
141.63
107.87
77.16 CDCl3
42.43
41.19
33.86
30.20
29.97
29.01
27.12
H_3C
CH_3
N
O
I
1x
^{13}C NMR (75 MHz, $CDCl_3$)
210 200 190 180 170 160 150 140 130 120 110 100 90 80 70 60 50 40 30 20 10 0 -10
f1 (ppm)

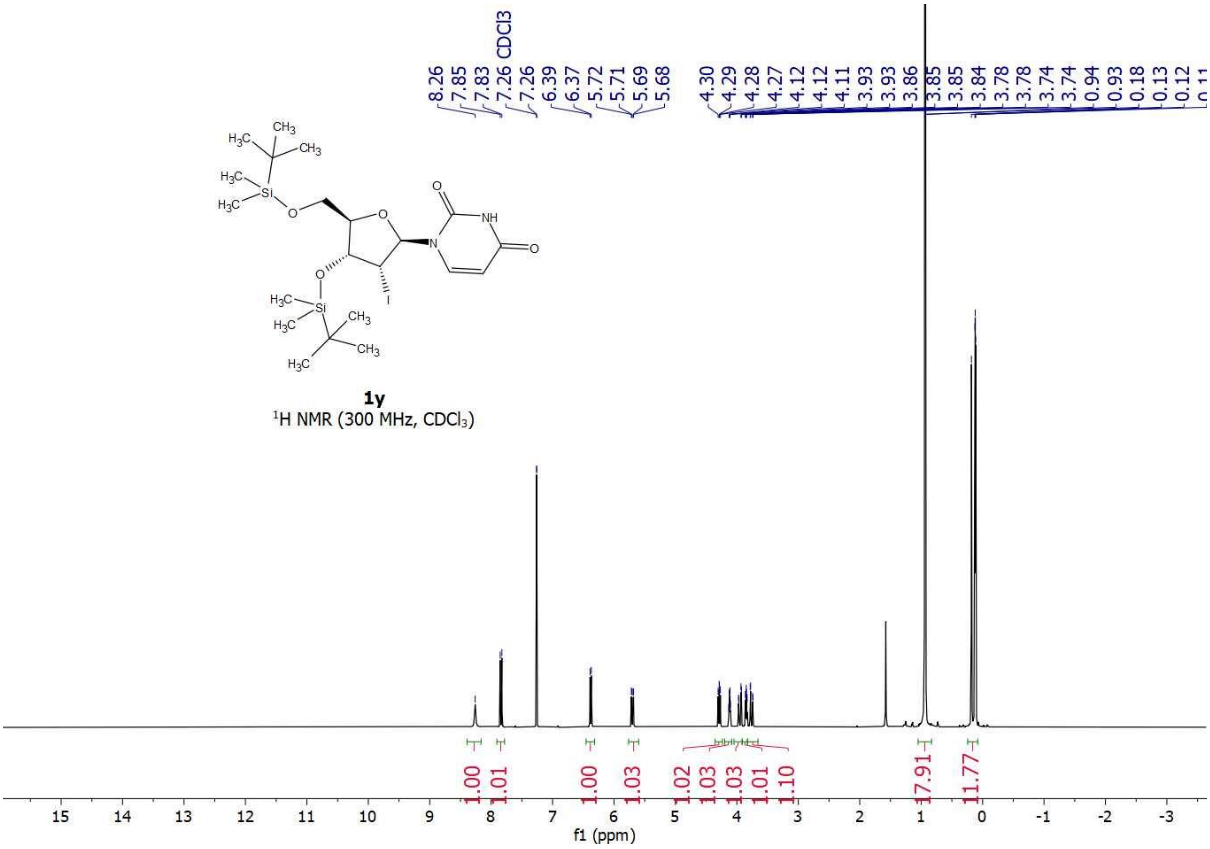
8.26
7.85
7.83
7.26 CDCl3
7.26
6.39
6.37
5.72
5.71
5.69
5.68
4.30
4.29
4.28
4.27
4.12
4.12
4.11
3.93
3.93
3.86
3.85
3.85
3.84
3.78
3.78
3.74
3.74
0.94
0.93
0.18
0.13
0.12
1y
^{1}H NMR (300 MHz, $CDCl_3$)
1.00
1.01
1.00
1.03
1.02
1.03
1.03
1.01
1.10
17.91
11.77
15 14 13 12 11 10 9 8 7 6 5 4 3 2 1 0 -1 -2 -3
f1 (ppm)

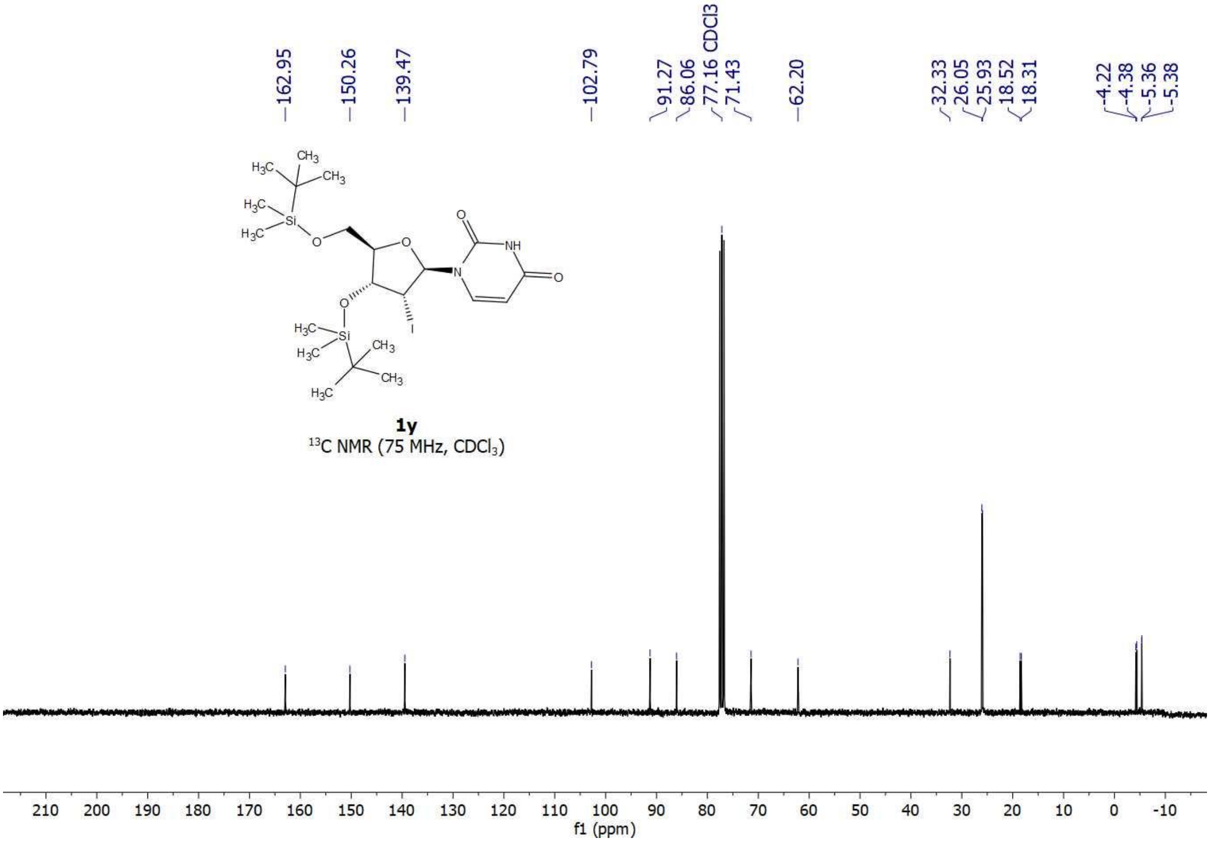
162.95
150.26
139.47
102.79
91.27
86.06
77.16 CDCl3
71.43
62.20
32.33
26.05
25.93
18.52
18.31
-4.22
-4.38
-5.36
-5.38
1y
^{13}C NMR (75 MHz, $CDCl_3$)
210 200 190 180 170 160 150 140 130 120 110 100 90 80 70 60 50 40 30 20 10 0 -10
f1 (ppm)

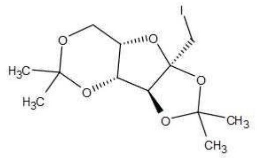

1z
^{1}H NMR (300 MHz, $CDCl_3$)

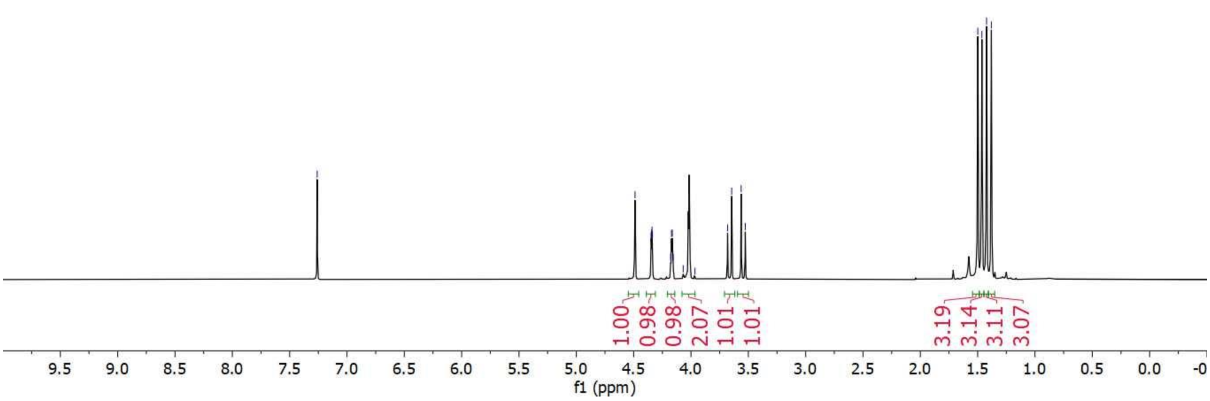

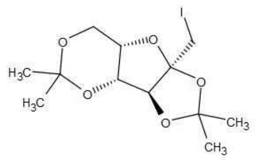

1z
^{13}C NMR (75 MHz, $CDCl_3$)

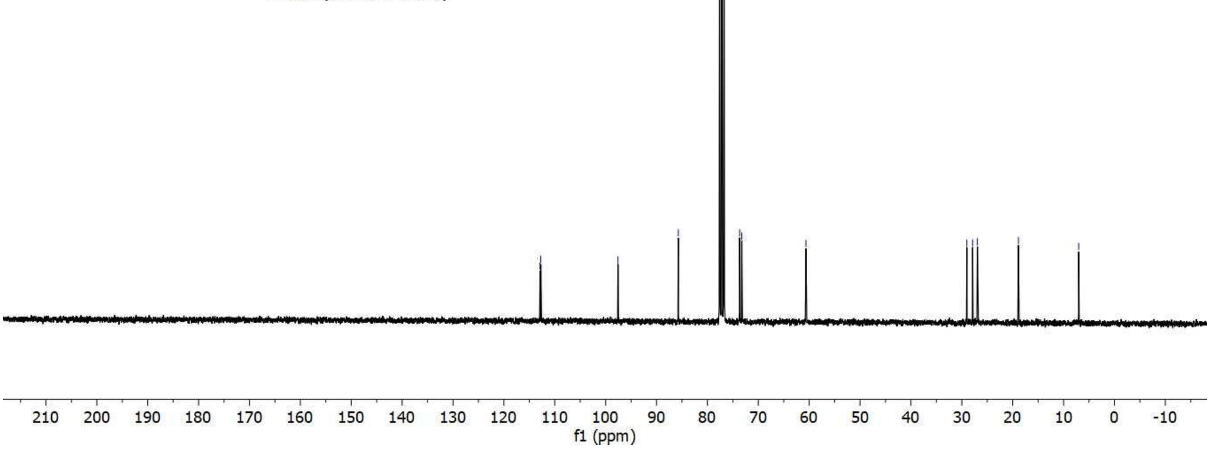

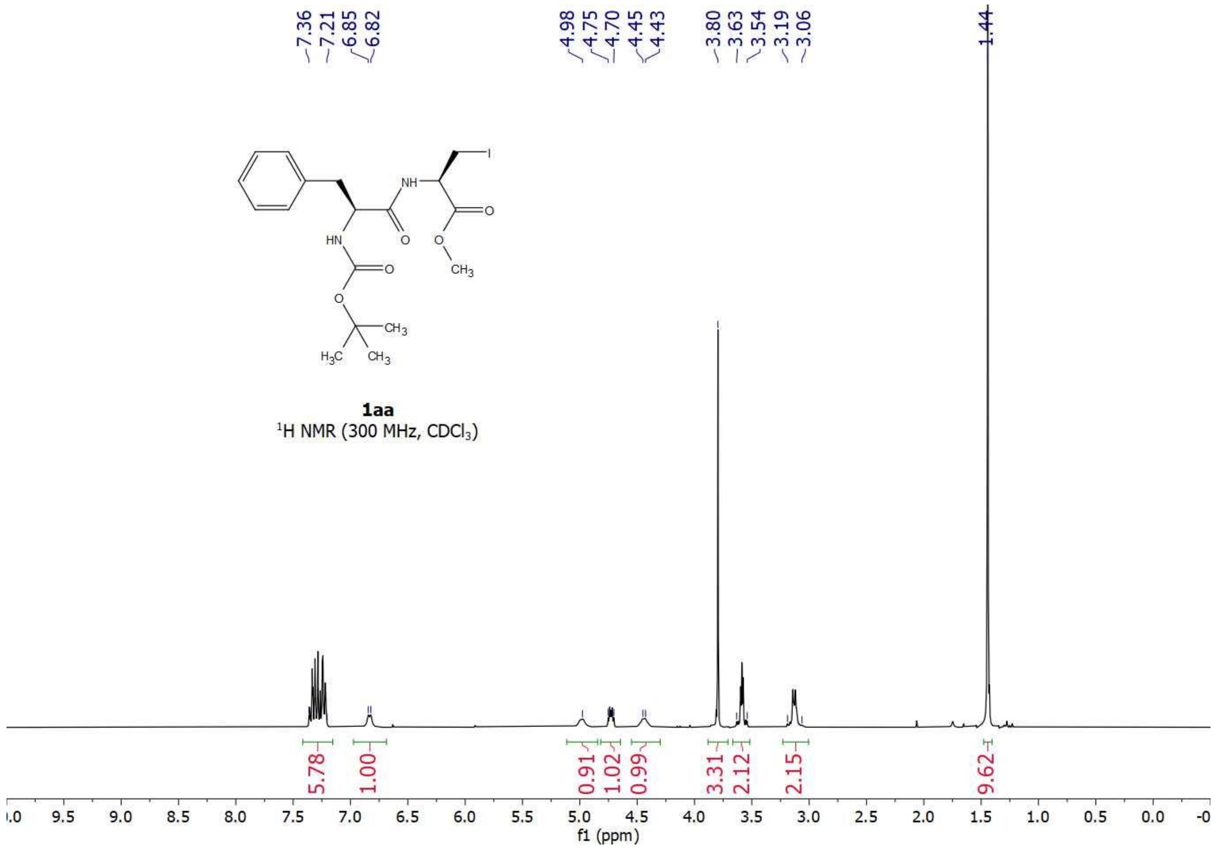

1aa
^{1}H NMR (300 MHz, $CDCl_3$)

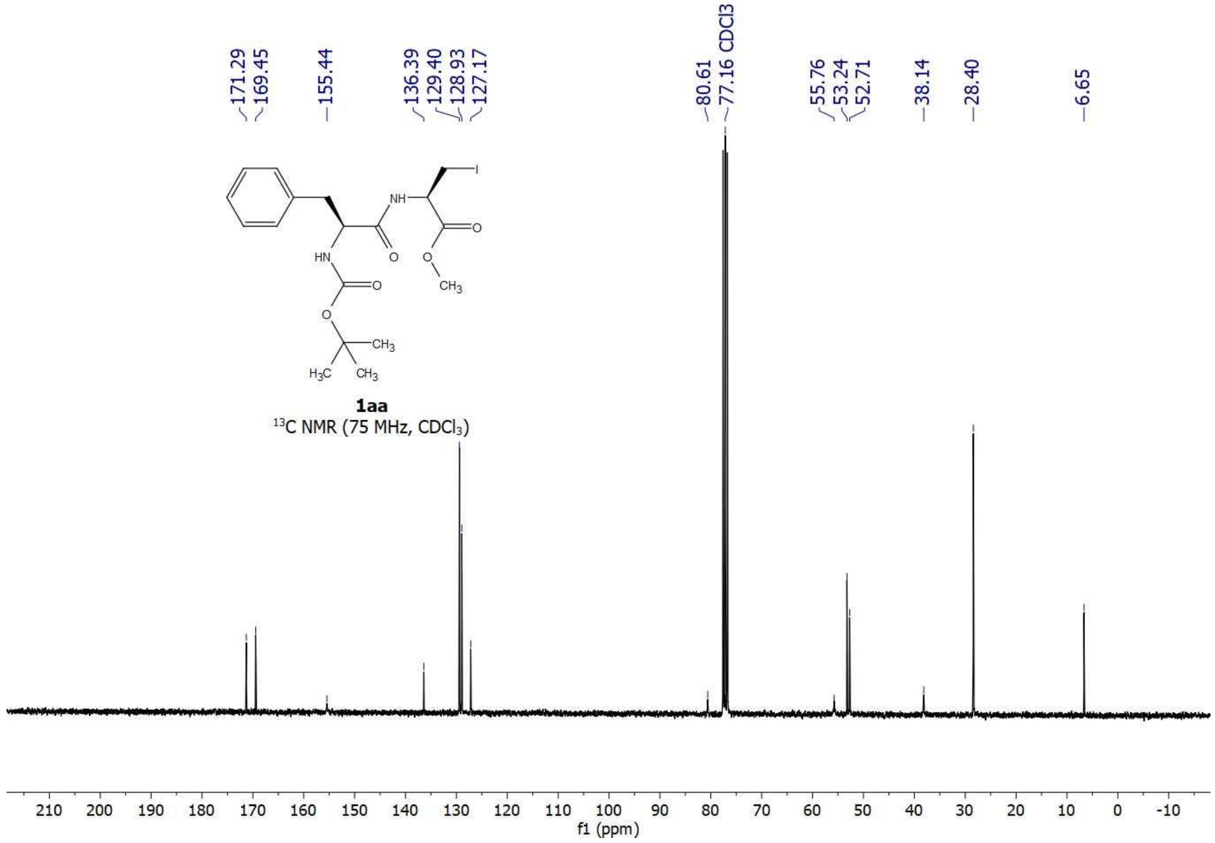

1aa
^{13}C NMR (75 MHz, $CDCl_3$)

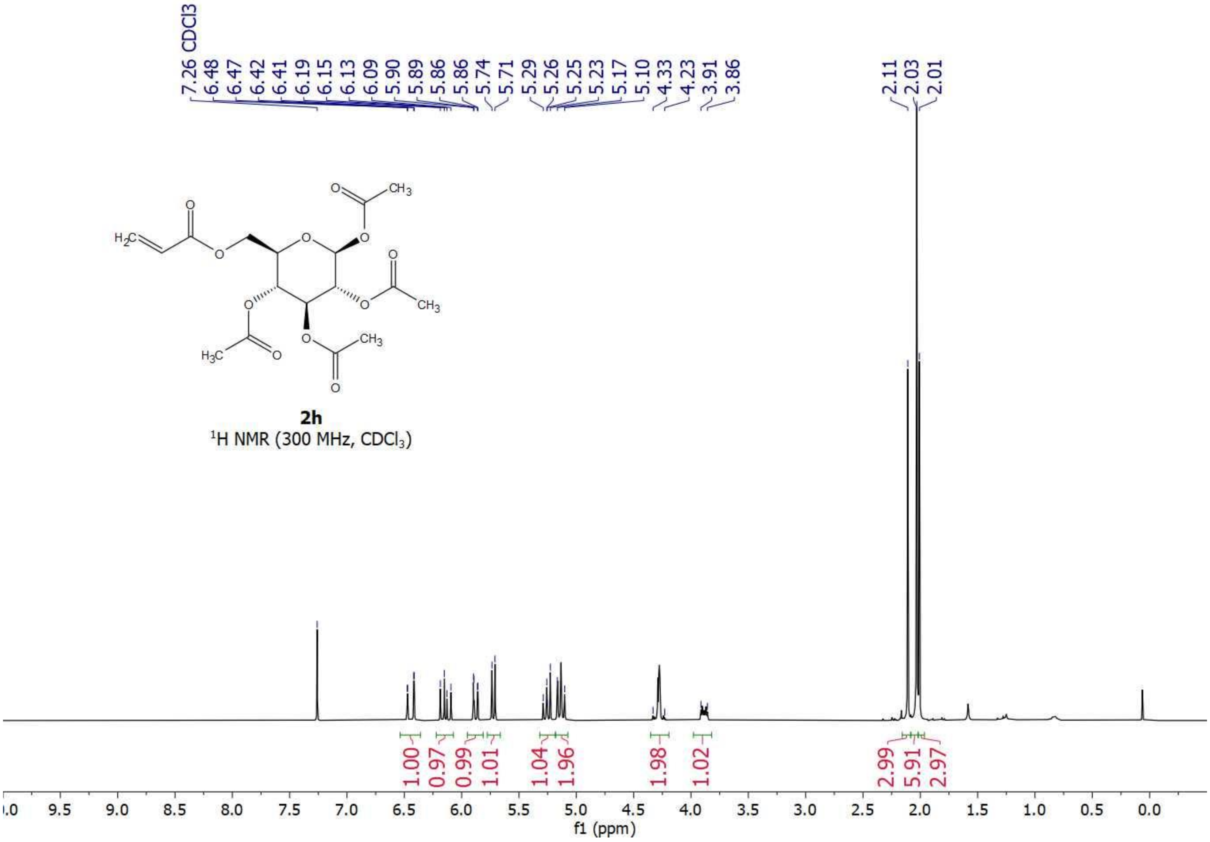

2h

^{1}H NMR (300 MHz, $CDCl_3$)

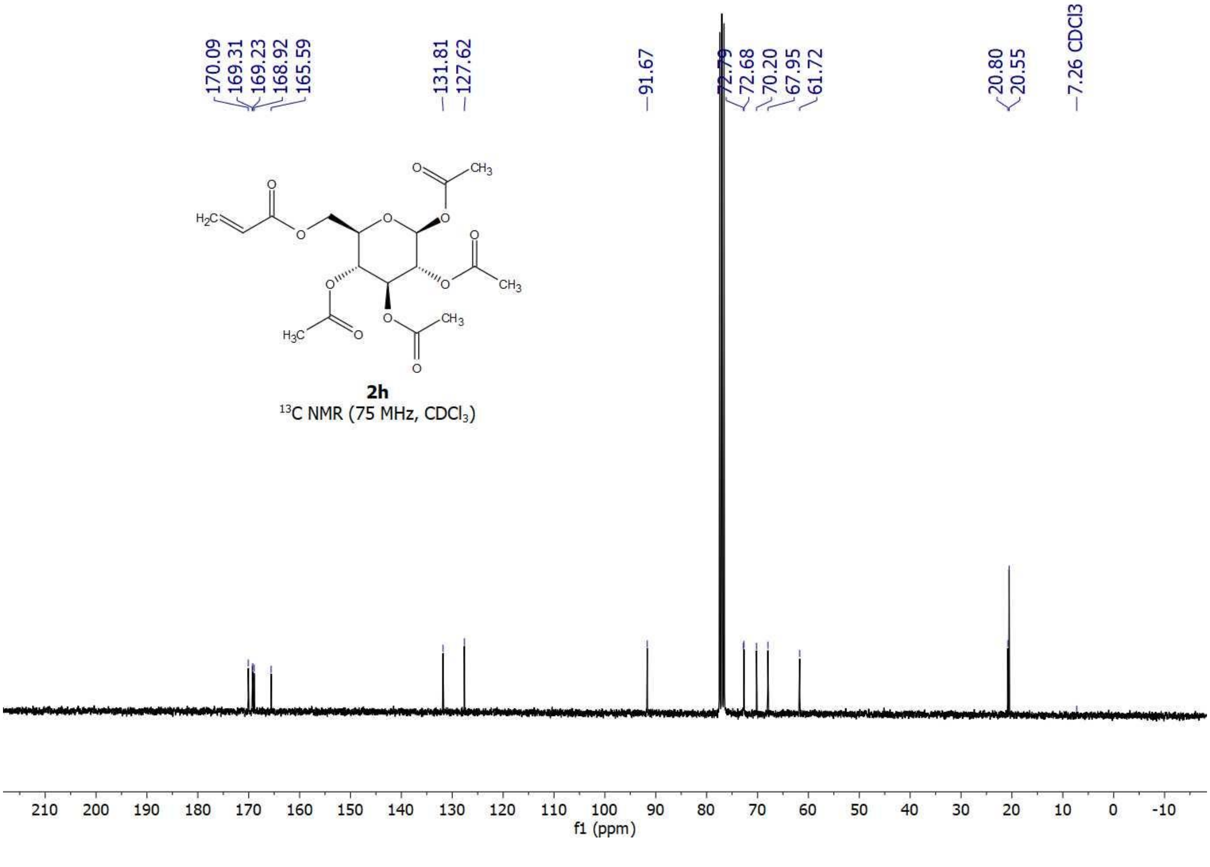

2h

^{13}C NMR (75 MHz, $CDCl_3$)

13.2 NMR spectra of products

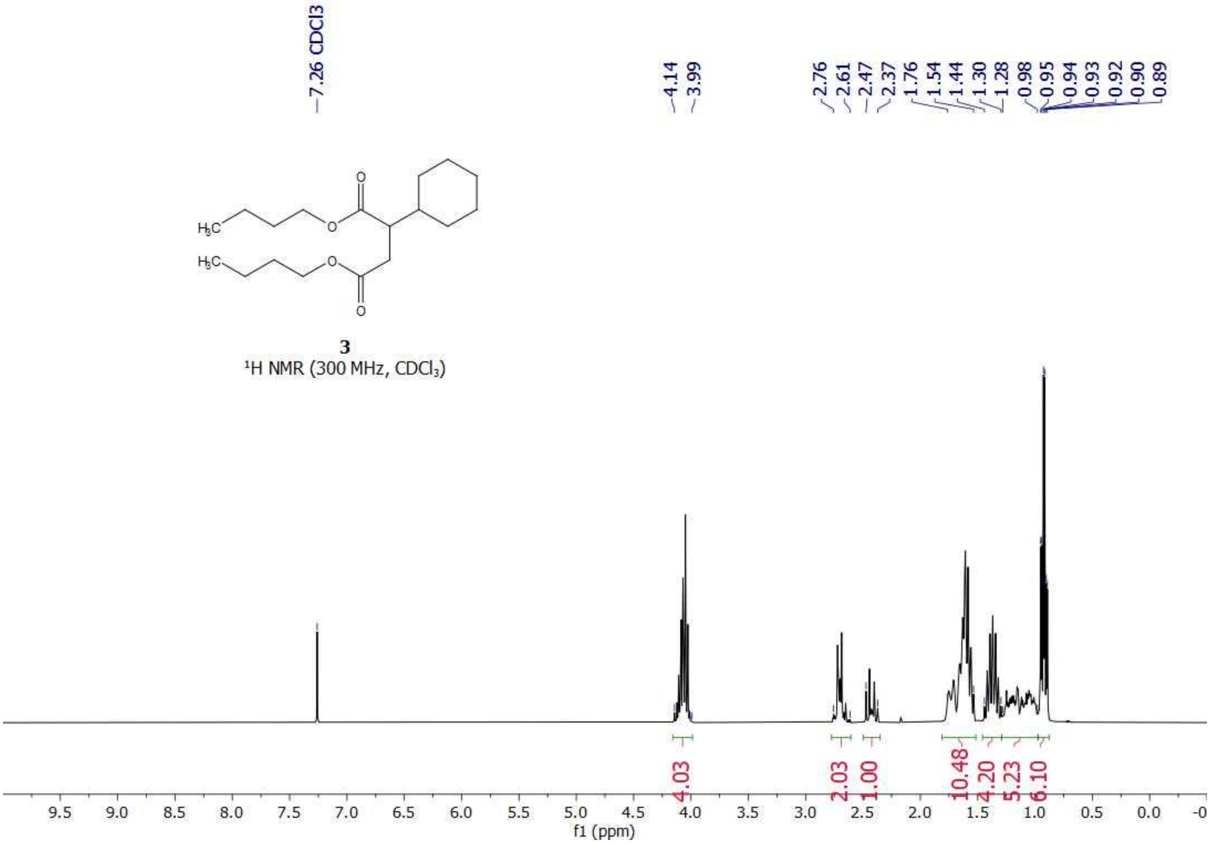

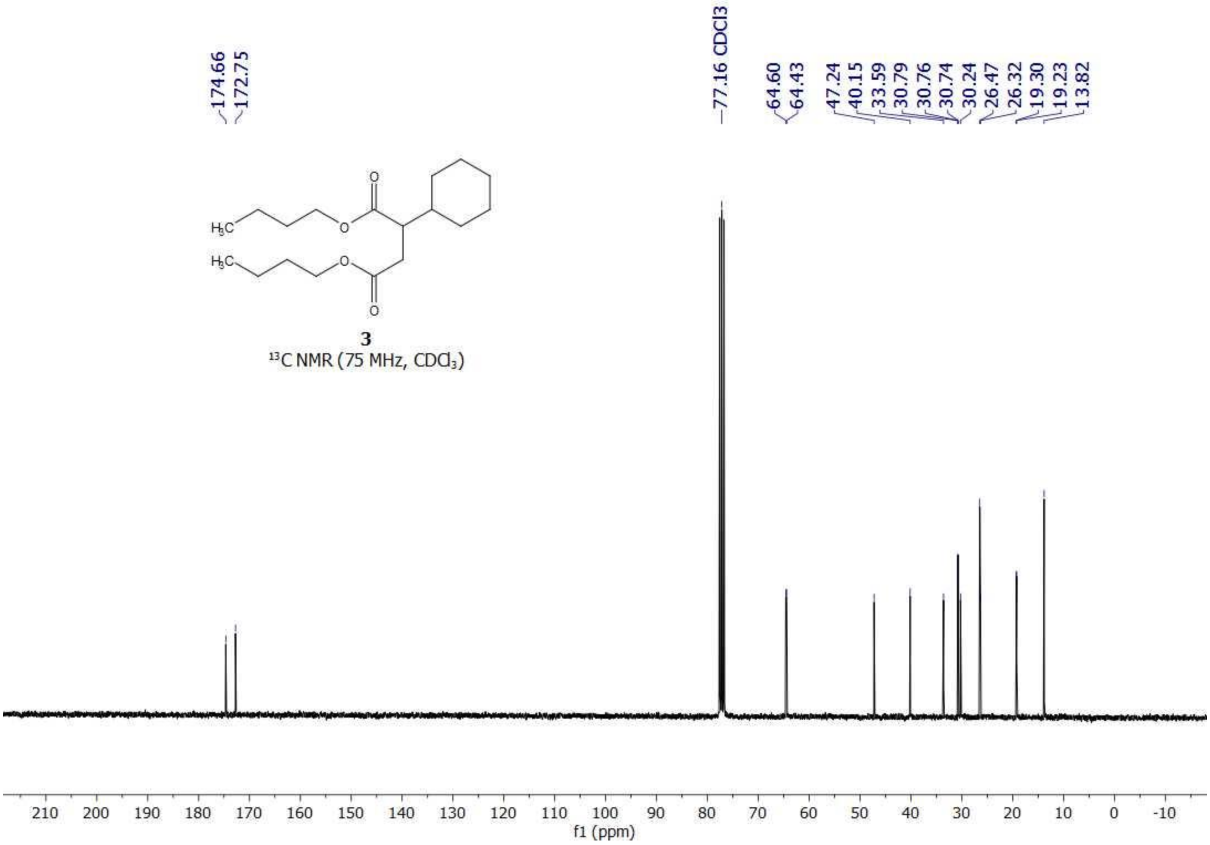

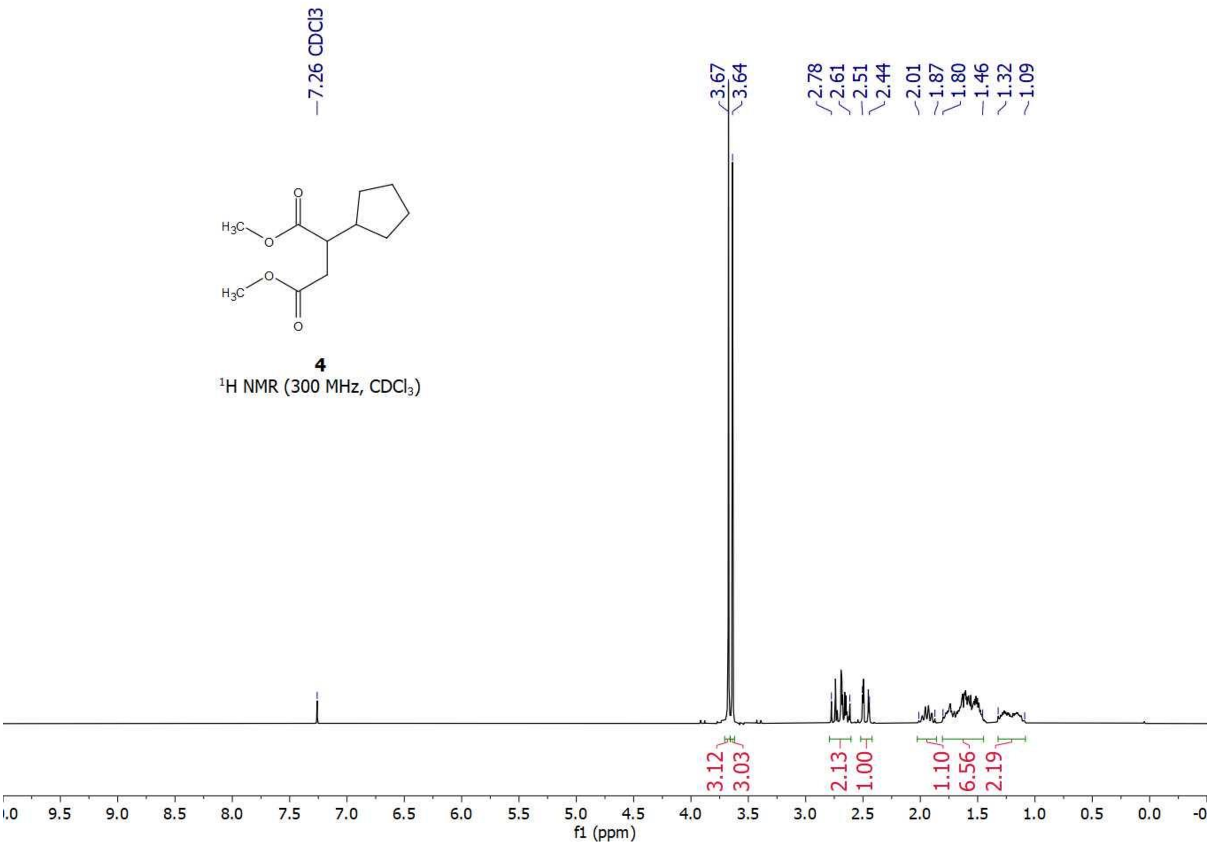
7.26 CDCl3
3.67
3.64
2.78
2.61
2.51
2.44
2.01
1.87
1.80
1.46
1.32
1.09
H_3C
O
4
^{1}H NMR (300 MHz, $CDCl_3$)
3.12
3.03
2.13
1.00
1.10
6.56
2.19
f1 (ppm)

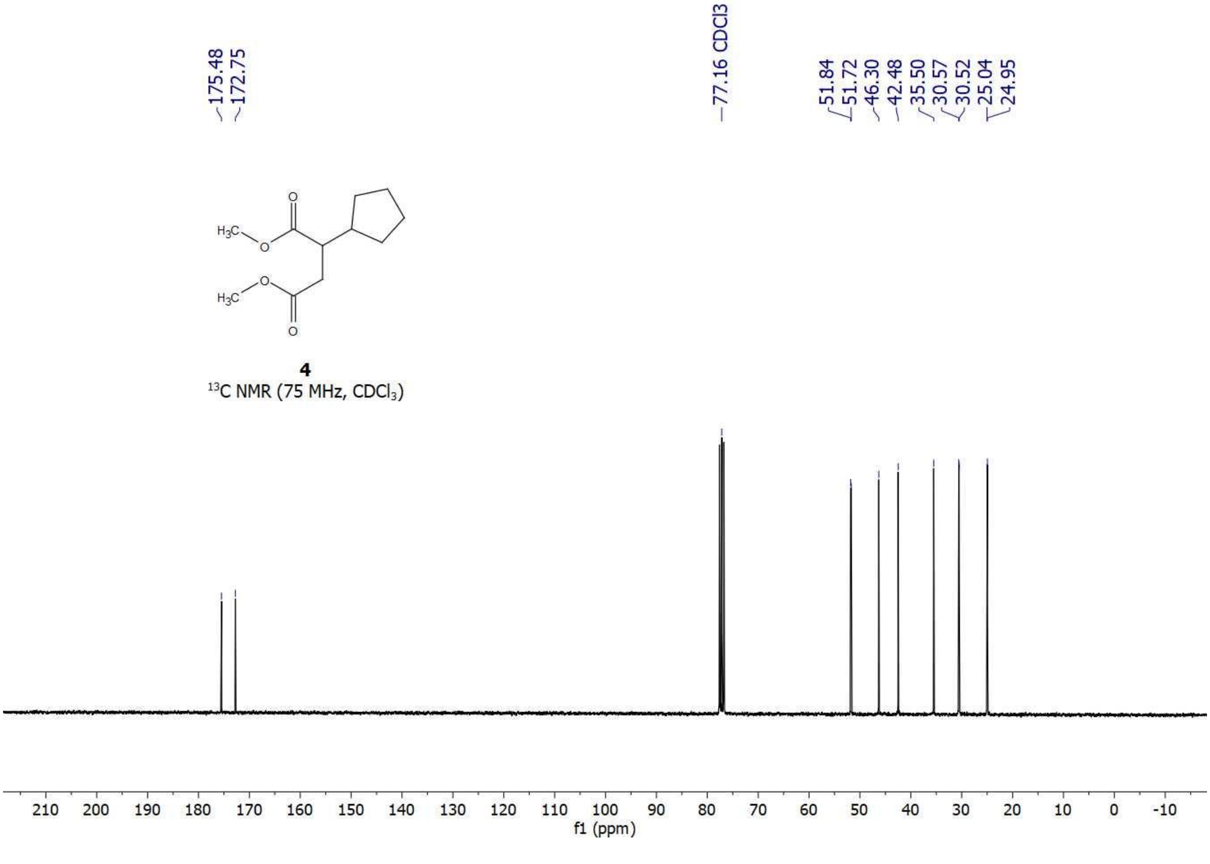
175.48
172.75
77.16 CDCl3
51.84
51.72
46.30
42.48
35.50
30.57
30.52
25.04
24.95
H_3C
O
4
^{13}C NMR (75 MHz, $CDCl_3$)
f1 (ppm)

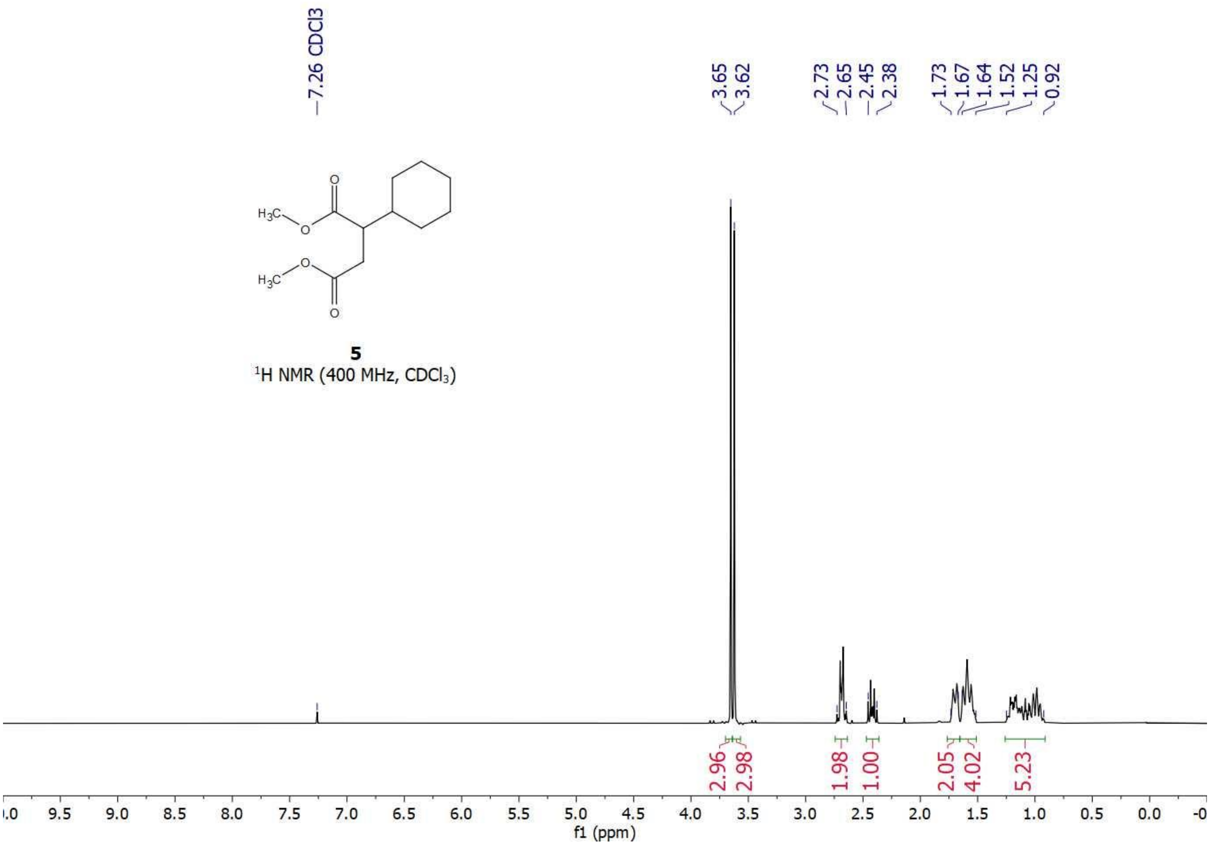

7.26 CDCl3
3.65
3.62
2.73
2.65
2.45
2.38
1.73
1.67
1.64
1.52
1.25
0.92
H3C O O
5
1H NMR (400 MHz, CDCl3)
2.96
2.98
1.98
1.00
2.05
4.02
5.23
f1 (ppm)

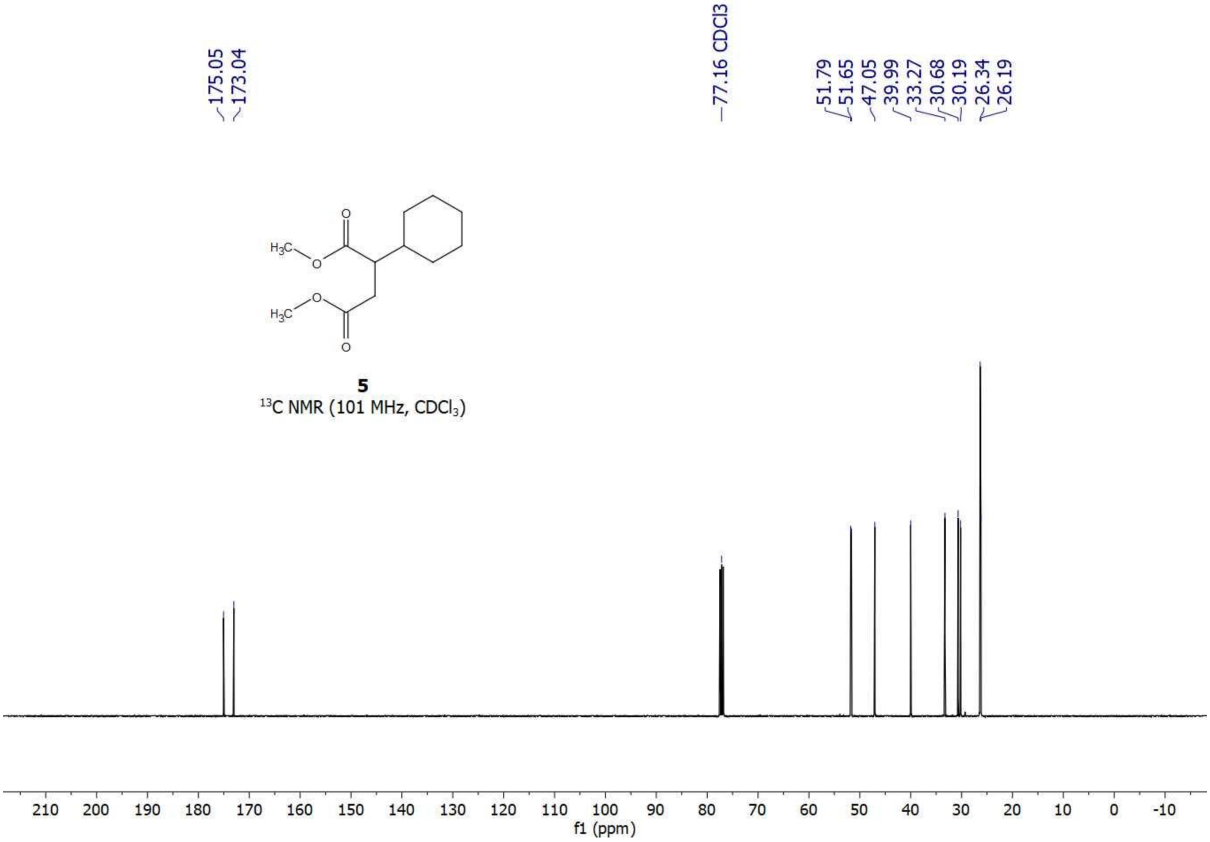

175.05
173.04
77.16 CDCl3
51.79
51.65
47.05
39.99
33.27
30.68
30.19
26.34
26.19
H3C O O
5
13C NMR (101 MHz, CDCl3)
f1 (ppm)

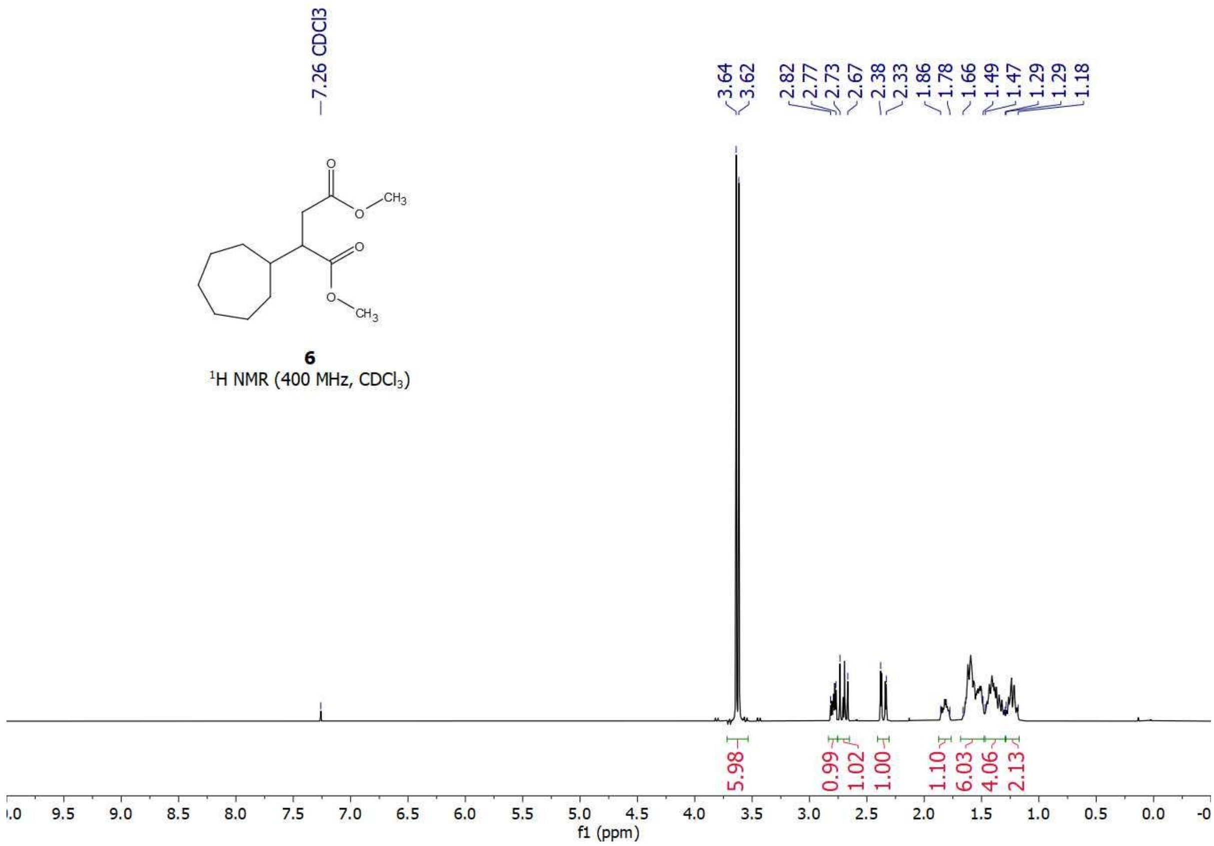

7.26 CDCl3
3.64
3.62
2.82
2.77
2.73
2.67
2.38
2.33
1.86
1.78
1.66
1.49
1.47
1.29
1.29
1.18
CH3
O
6
1H NMR (400 MHz, CDCl3)
5.98
0.99
1.02
1.00
1.10
6.03
4.06
2.13
f1 (ppm)

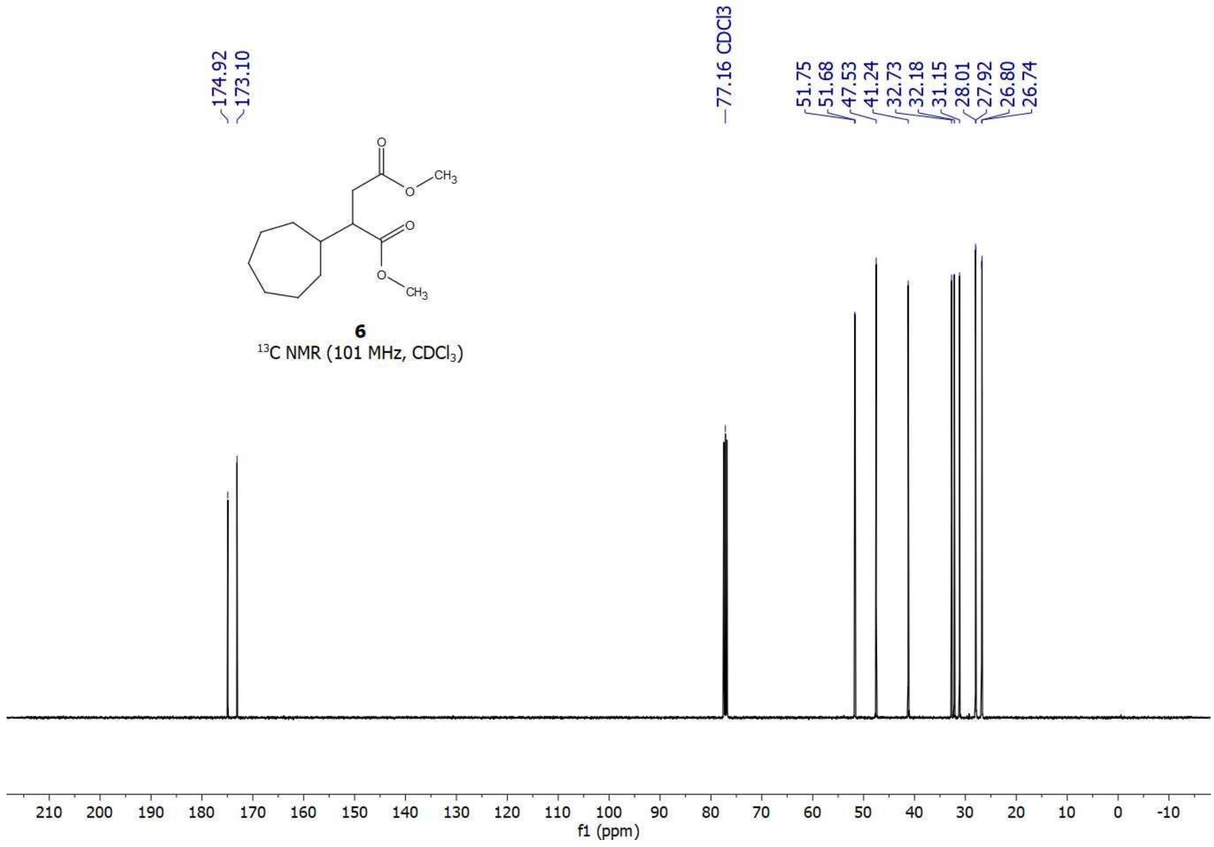

174.92
173.10
77.16 CDCl3
51.75
51.68
47.53
41.24
32.73
32.18
31.15
28.01
27.92
26.80
26.74
CH3
O
6
13C NMR (101 MHz, CDCl3)
f1 (ppm)

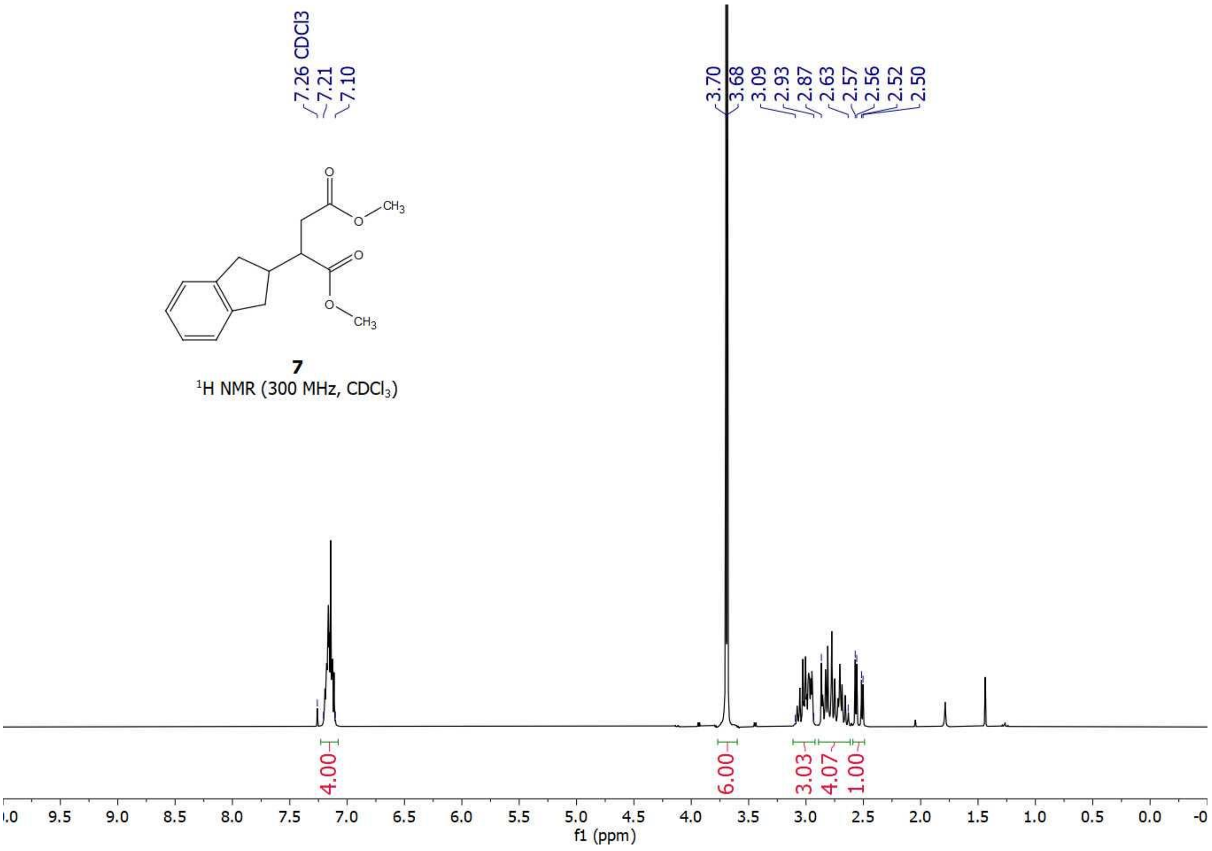
7.26 CDCl3
7.21
7.10
3.70
3.68
3.09
2.93
2.87
2.63
2.57
2.56
2.52
2.50
CH3
O
O
O
O
CH3
7
^{1}H NMR (300 MHz, $CDCl_3$)
4.00
6.00
3.03
4.07
1.00
9.5
9.0
8.5
8.0
7.5
7.0
6.5
6.0
5.5
5.0
4.5
4.0
3.5
3.0
2.5
2.0
1.5
1.0
0.5
0.0
f1 (ppm)

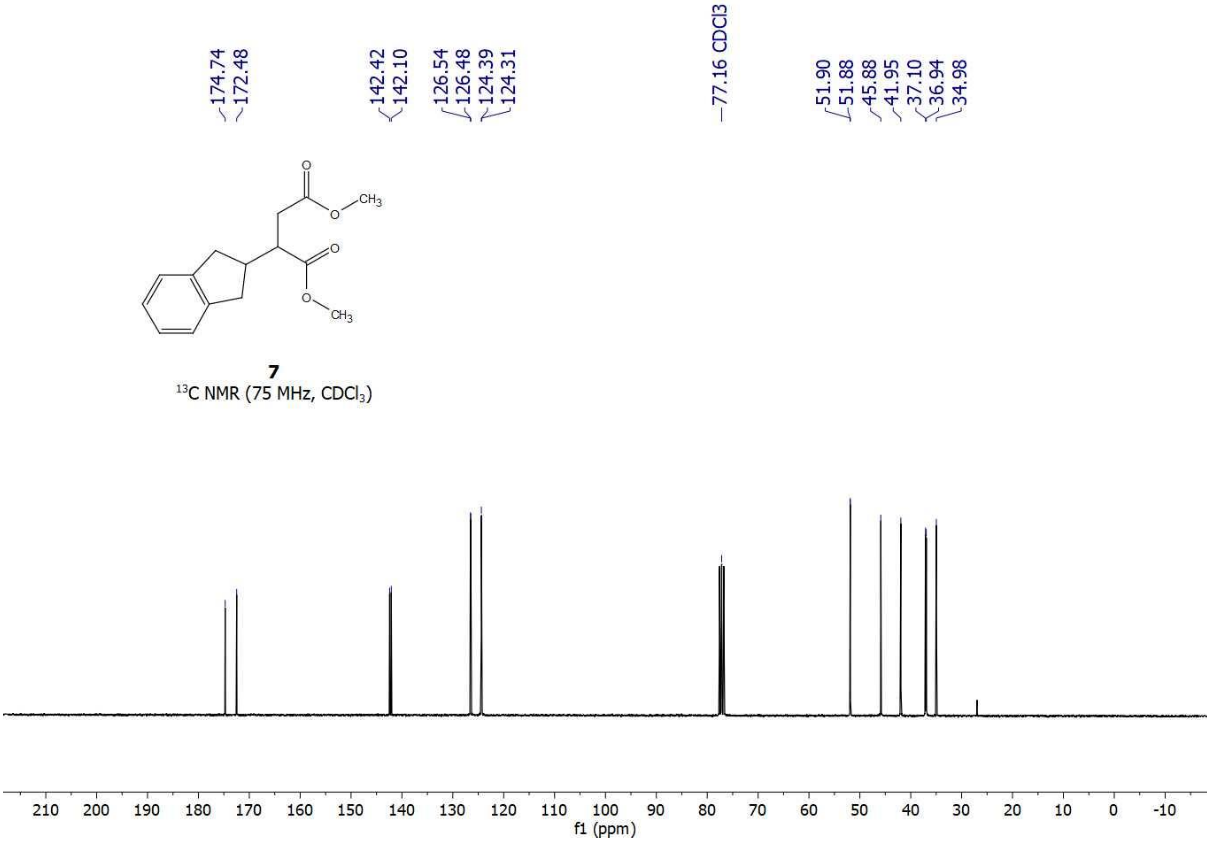
174.74
172.48
142.42
142.10
126.54
126.48
124.39
124.31
77.16 CDCl3
51.90
51.88
45.88
41.95
37.10
36.94
34.98
CH3
O
O
O
O
CH3
7
^{13}C NMR (75 MHz, $CDCl_3$)
210
200
190
180
170
160
150
140
130
120
110
100
90
80
70
60
50
40
30
20
10
0
-10
f1 (ppm)

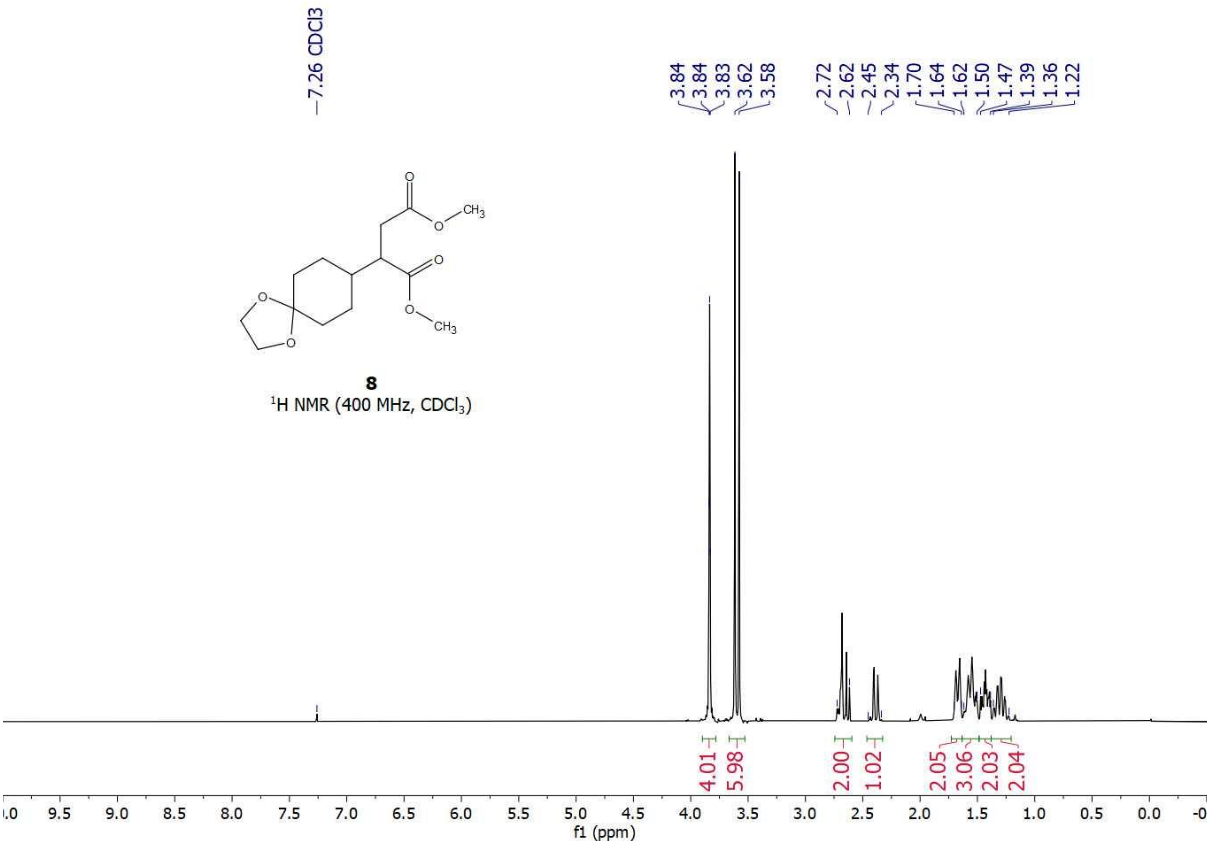
7.26 CDCl3
3.84
3.84
3.83
3.62
3.58
2.72
2.62
2.45
2.34
1.70
1.64
1.62
1.50
1.47
1.39
1.36
1.22
CH3
CH3
O
O
O
O
O
O
8
1H NMR (400 MHz, CDCl3)
4.01
5.98
2.00
1.02
2.05
3.06
2.03
2.04
f1 (ppm)

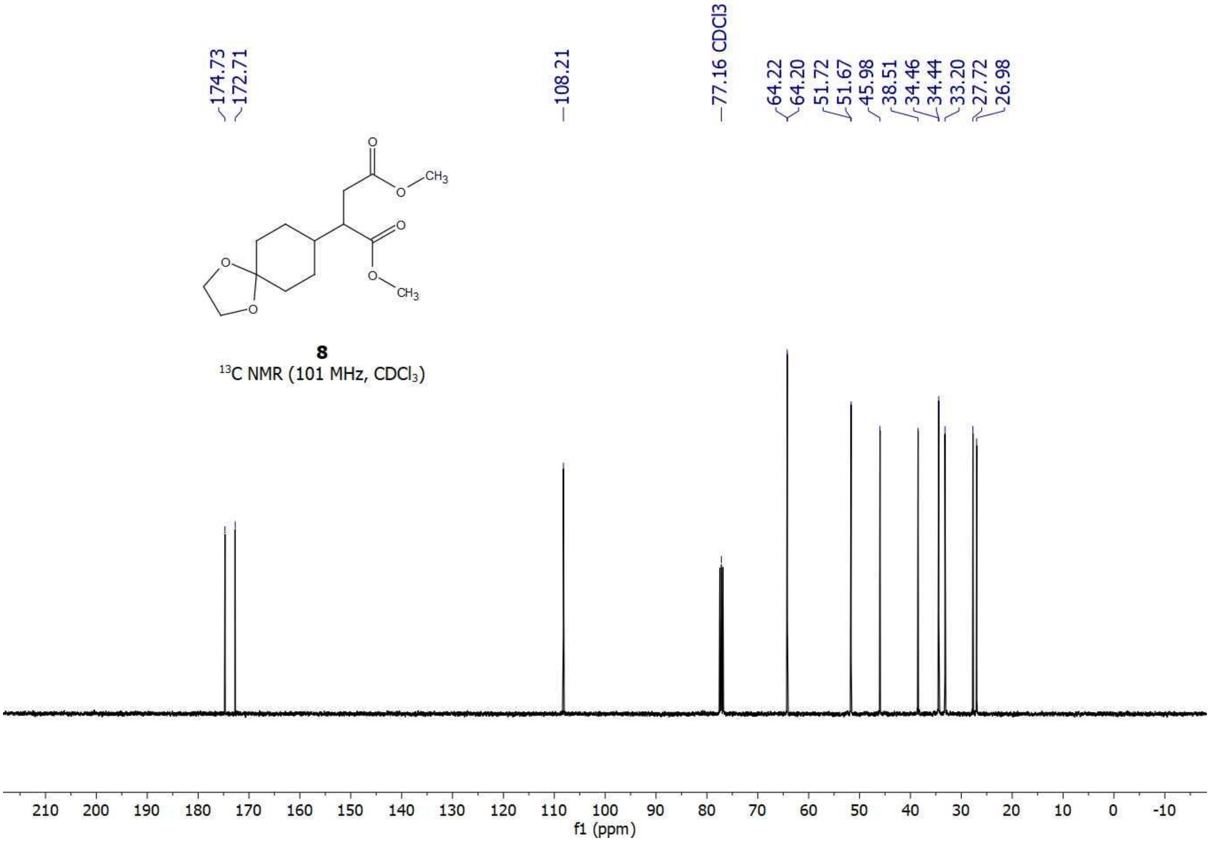
174.73
172.71
108.21
77.16 CDCl3
64.22
64.20
51.72
51.67
45.98
38.51
34.46
34.44
33.20
27.72
26.98
CH3
CH3
O
O
O
O
O
O
8
13C NMR (101 MHz, CDCl3)
f1 (ppm)

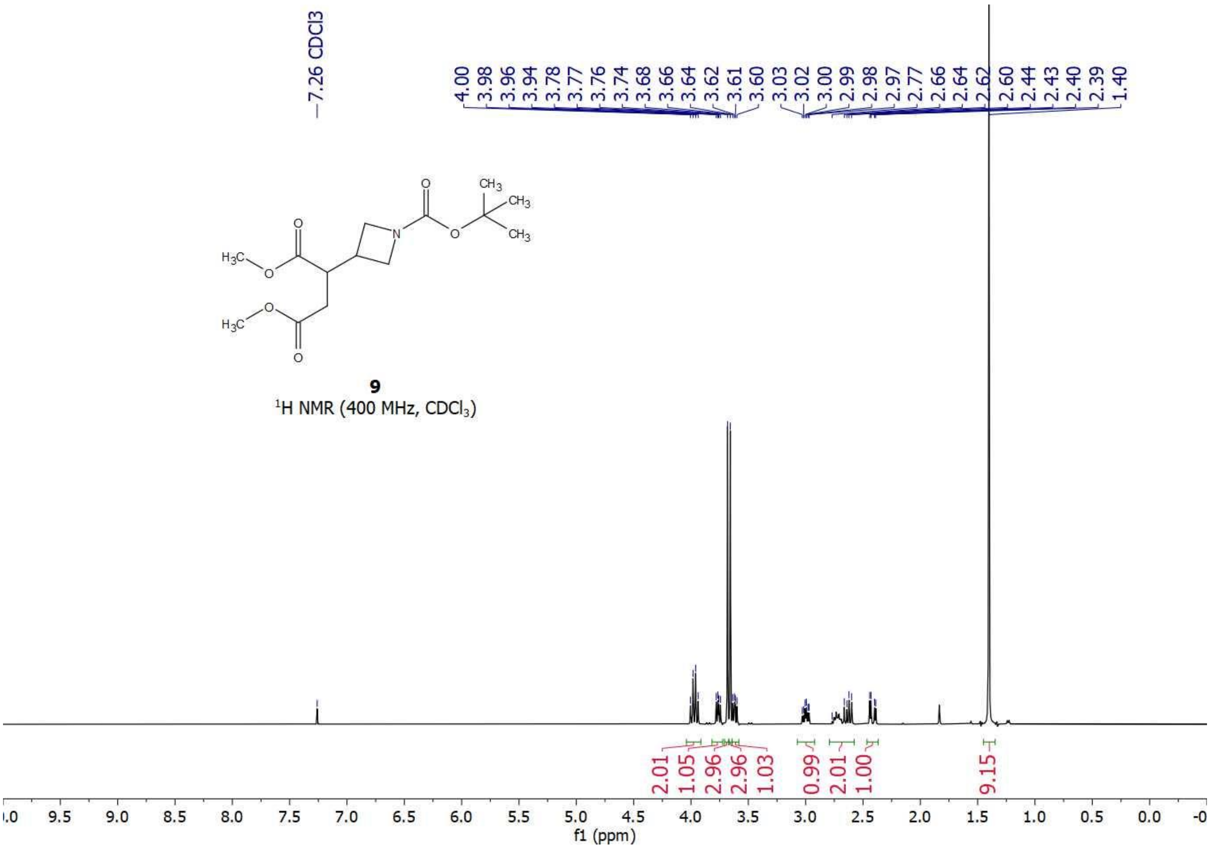
7.26 CDCl3
4.00 3.98 3.96 3.94 3.78 3.77 3.76 3.74 3.68 3.66 3.64 3.62 3.61 3.60 3.03 3.02 3.00 2.99 2.98 2.97 2.77 2.66 2.64 2.62 2.60 2.44 2.43 2.40 2.39 1.40
9
^{1}H NMR (400 MHz, $CDCl_3$)
2.01 1.05 2.96 2.96 1.03 0.99 2.01 1.00 9.15
f1 (ppm)

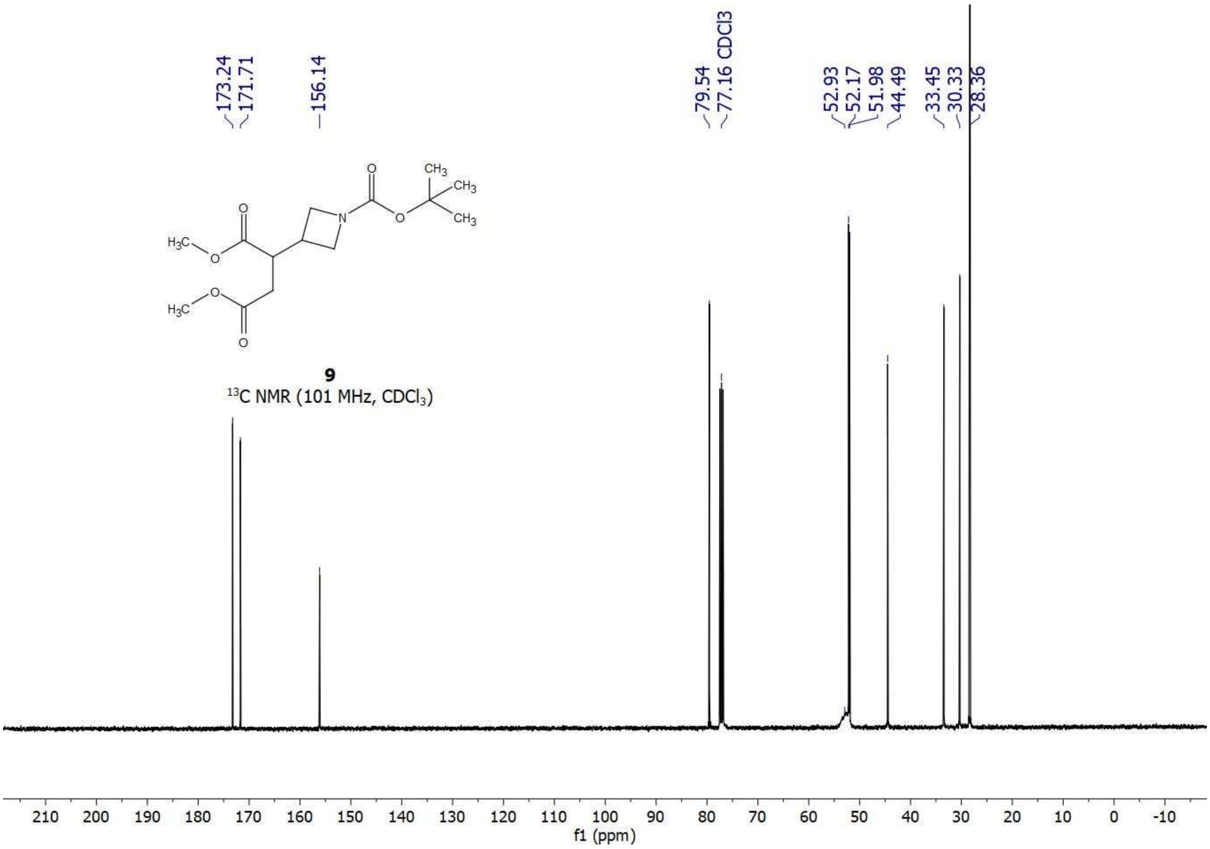
173.24 171.71 156.14 79.54 77.16 CDCl3 52.93 52.17 51.98 44.49 33.45 30.33 28.36
9
^{13}C NMR (101 MHz, $CDCl_3$)
f1 (ppm)

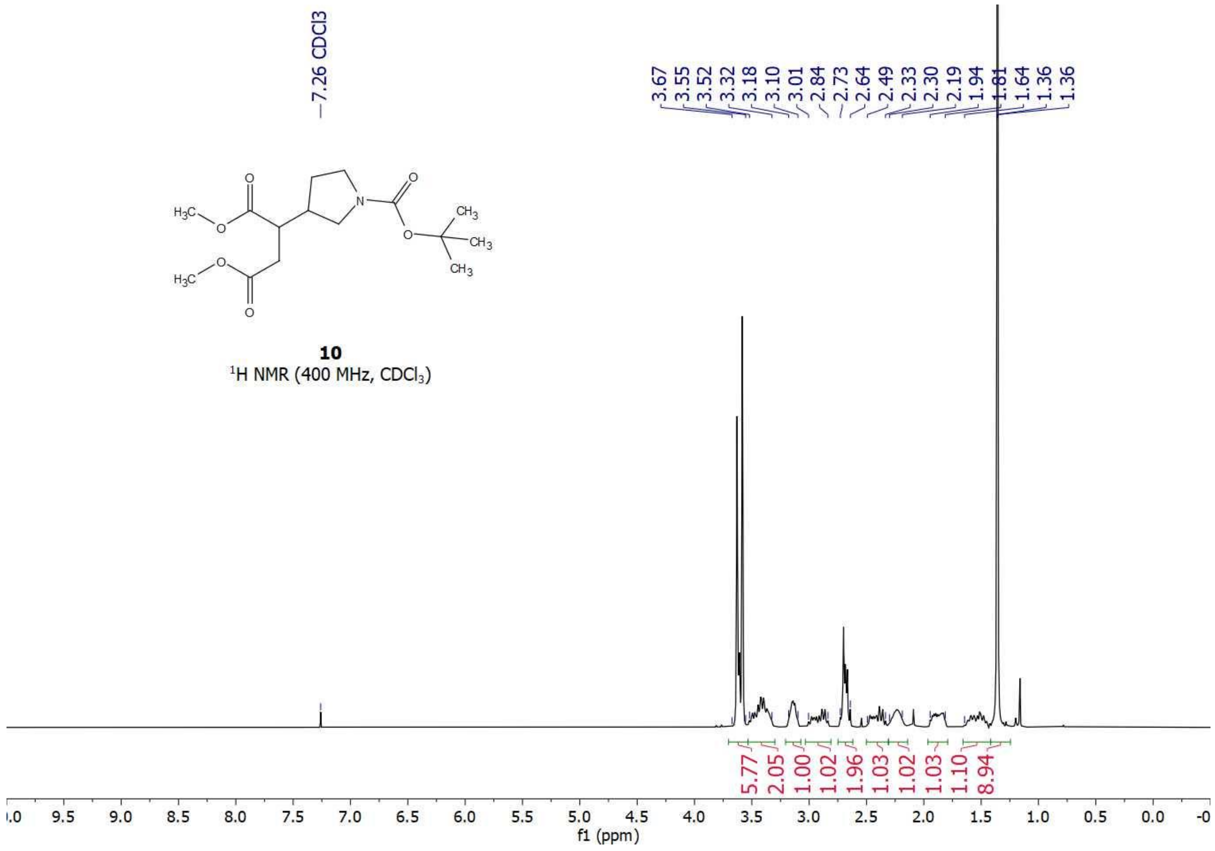

10

^{1}H NMR (400 MHz, $CDCl_3$)

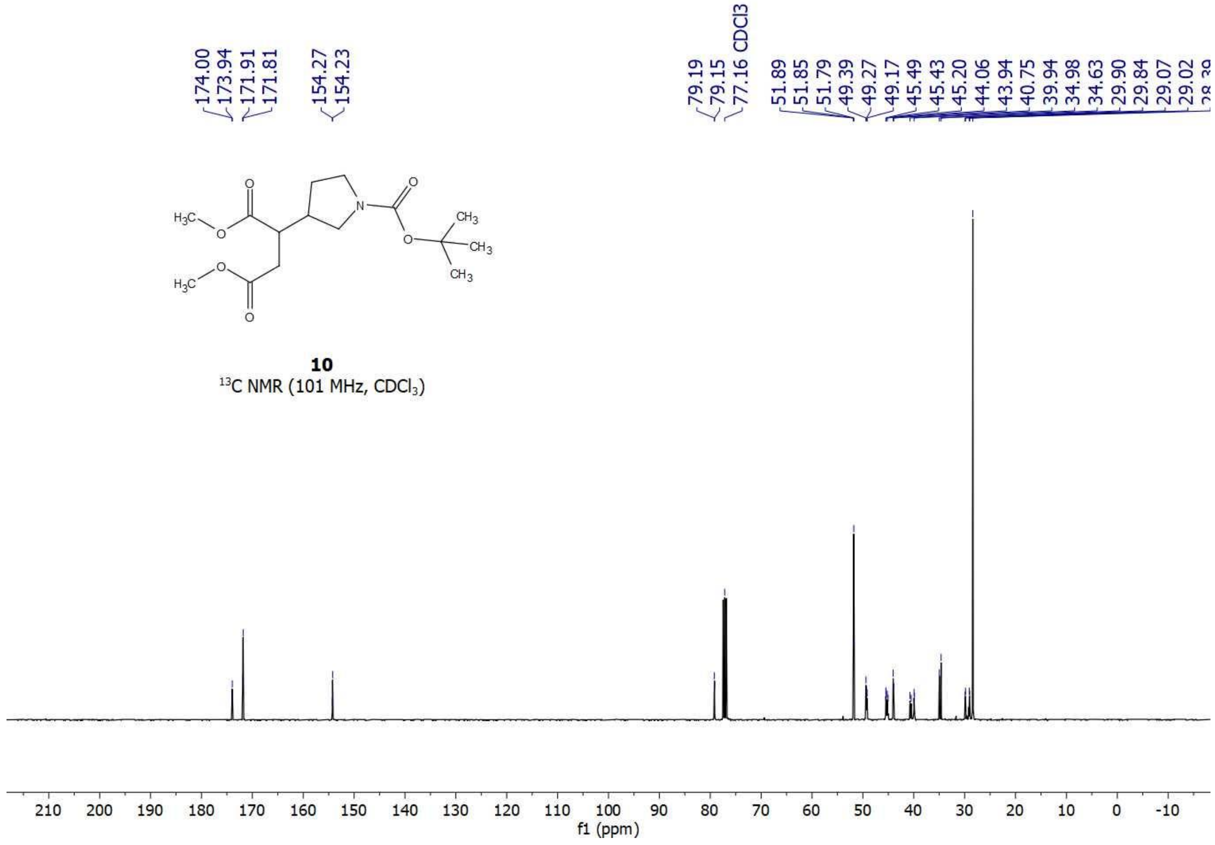

10

^{13}C NMR (101 MHz, $CDCl_3$)

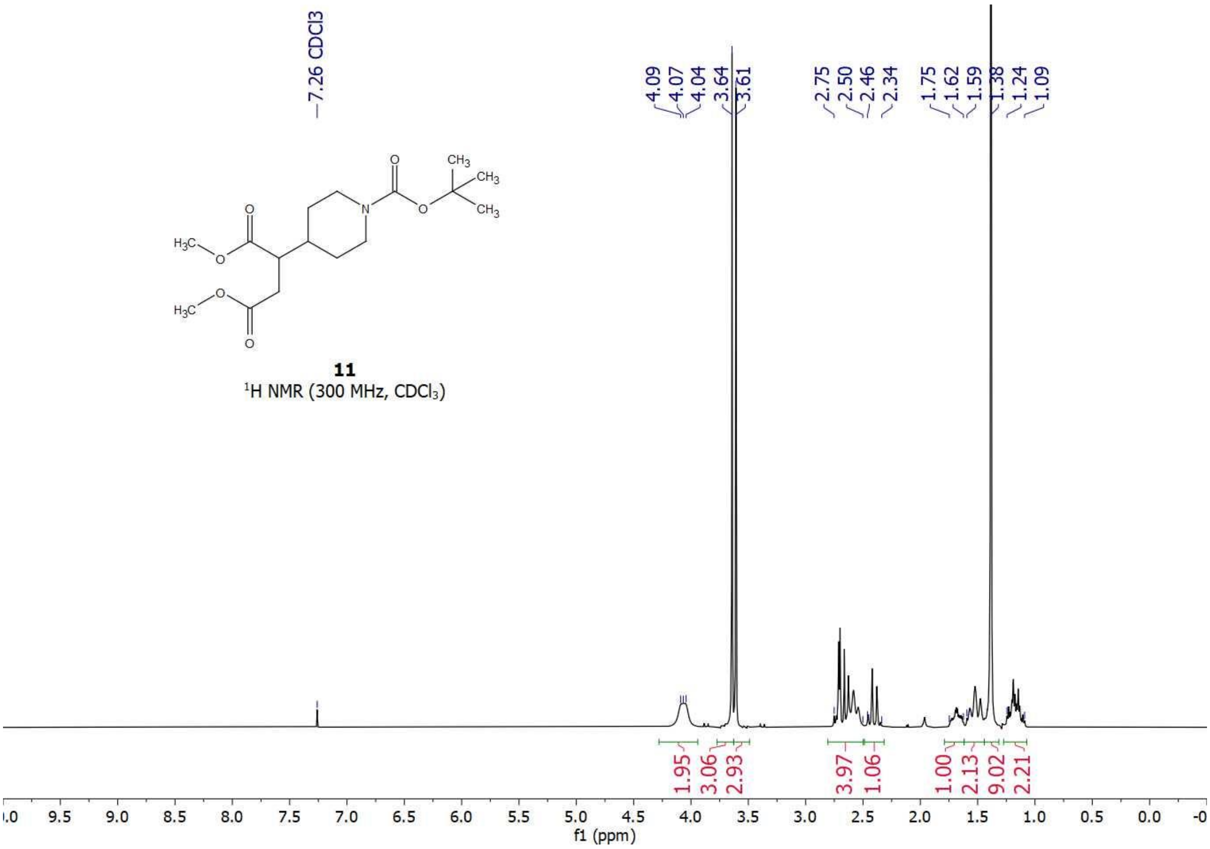
7.26 CDCl3
4.09
4.07
4.04
3.64
3.61
2.75
2.50
2.46
2.34
1.75
1.62
1.59
1.38
1.24
1.09
11
1H NMR (300 MHz, CDCl3)
1.95
3.06
2.93
3.97
1.06
1.00
2.13
9.02
2.21
f1 (ppm)

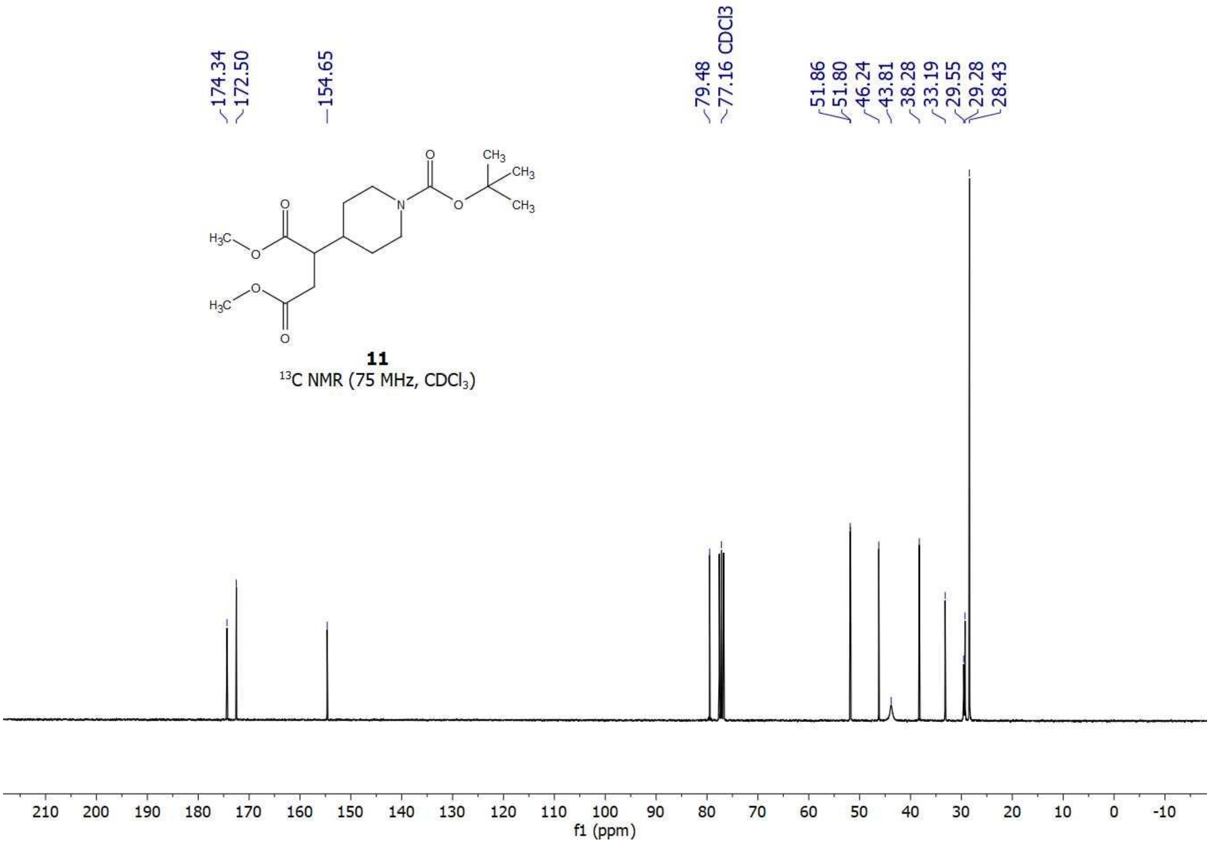
174.34
172.50
154.65
79.48
77.16 CDCl3
51.86
51.80
46.24
43.81
38.28
33.19
29.55
29.28
28.43
11
13C NMR (75 MHz, CDCl3)
f1 (ppm)

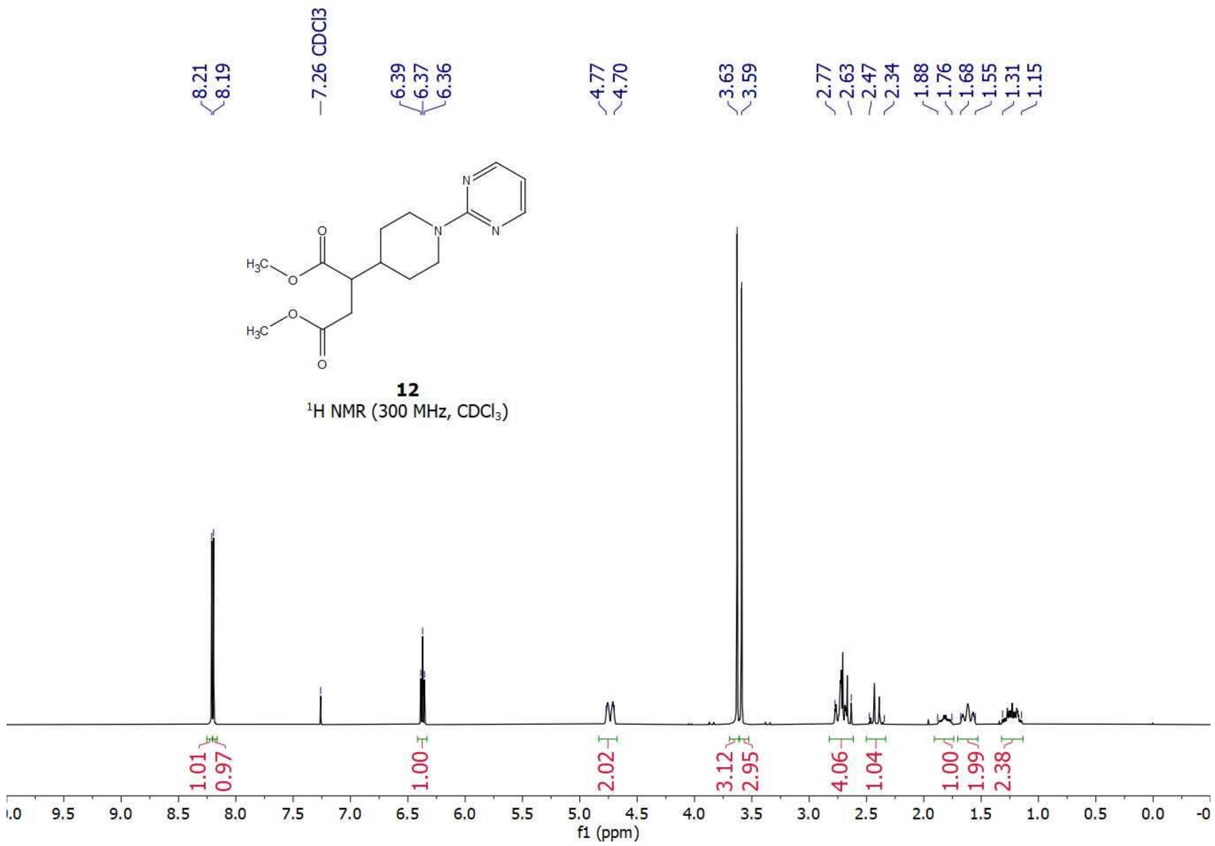

12

^{1}H NMR (300 MHz, $CDCl_3$)

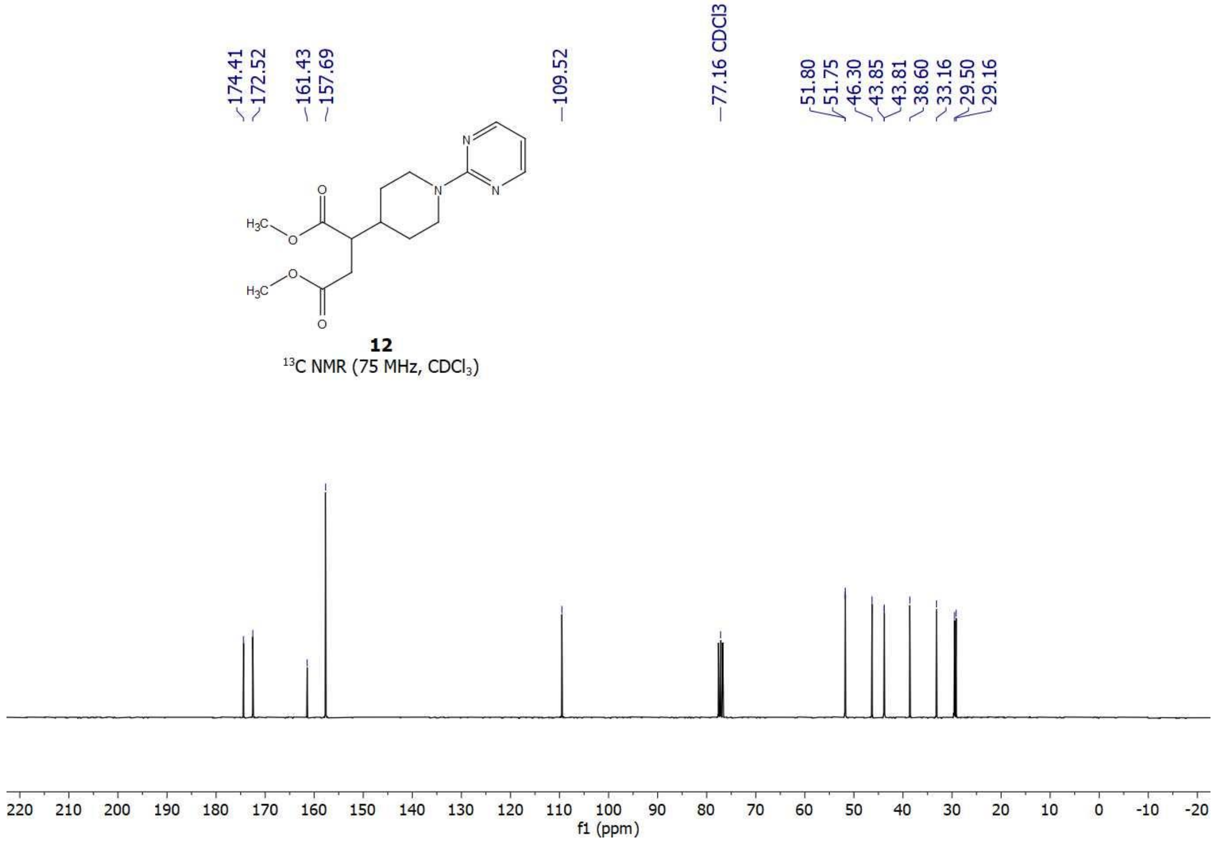

12

^{13}C NMR (75 MHz, $CDCl_3$)

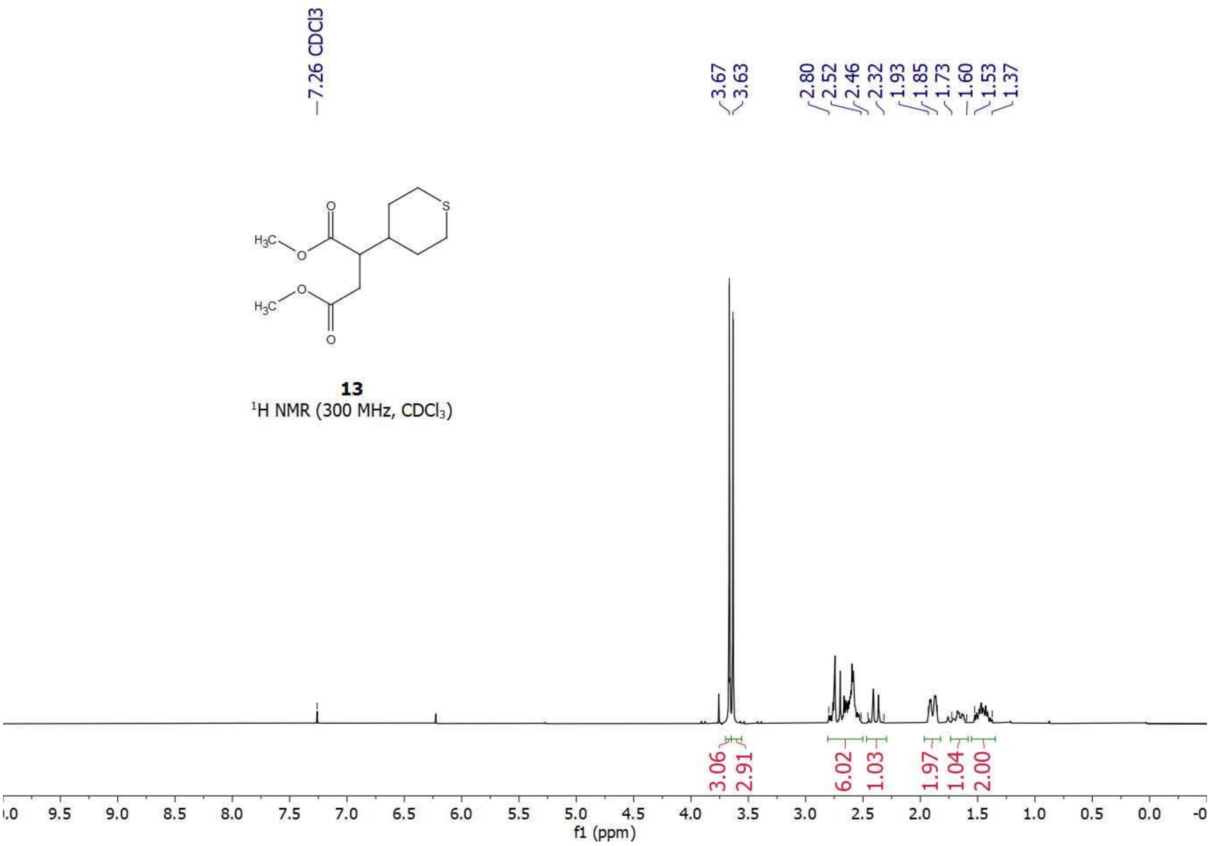

7.26 CDCl3
3.67
3.63
2.80
2.52
2.46
2.32
1.93
1.85
1.73
1.60
1.53
1.37
H3C
O
O
S
13
1H NMR (300 MHz, CDCl3)
3.06
2.91
6.02
1.03
1.97
1.04
2.00
f1 (ppm)

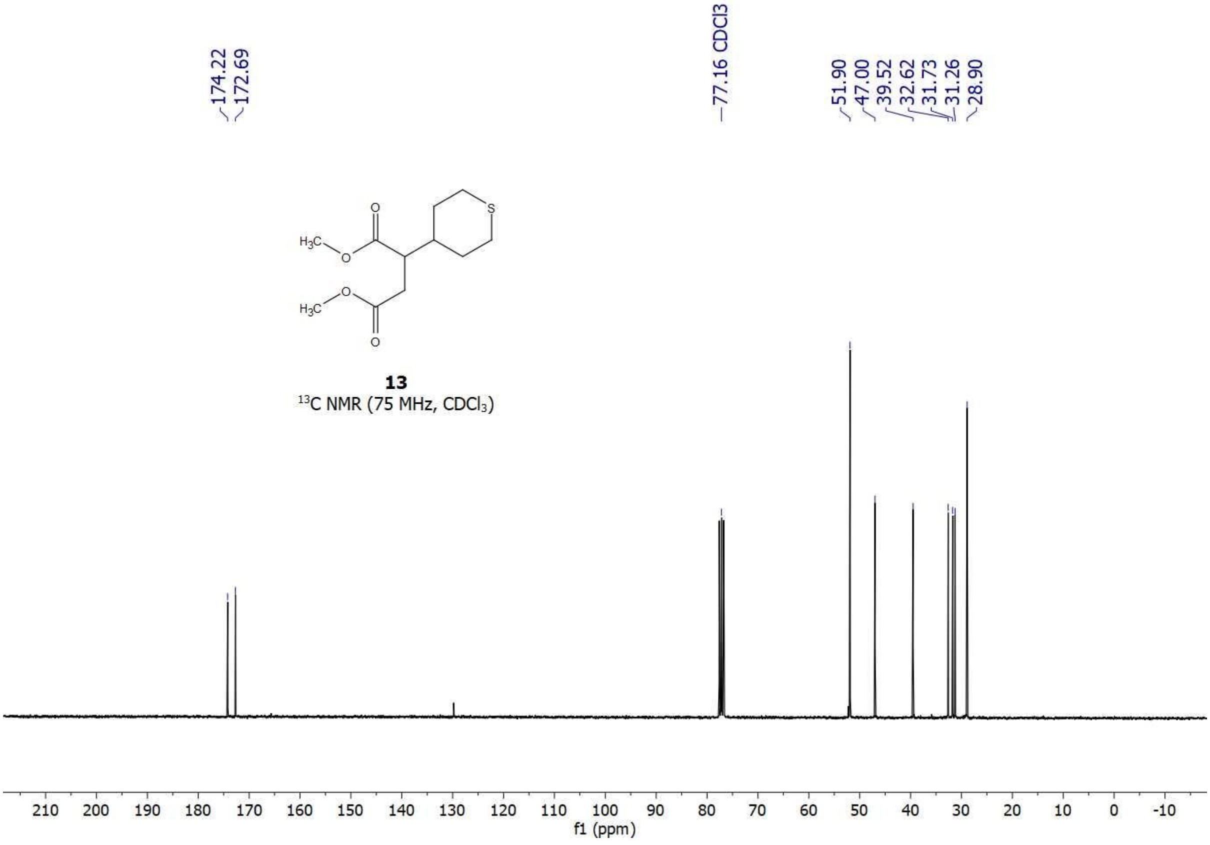

174.22
172.69
77.16 CDCl3
51.90
47.00
39.52
32.62
31.73
31.26
28.90
H3C
O
O
S
13
13C NMR (75 MHz, CDCl3)
f1 (ppm)

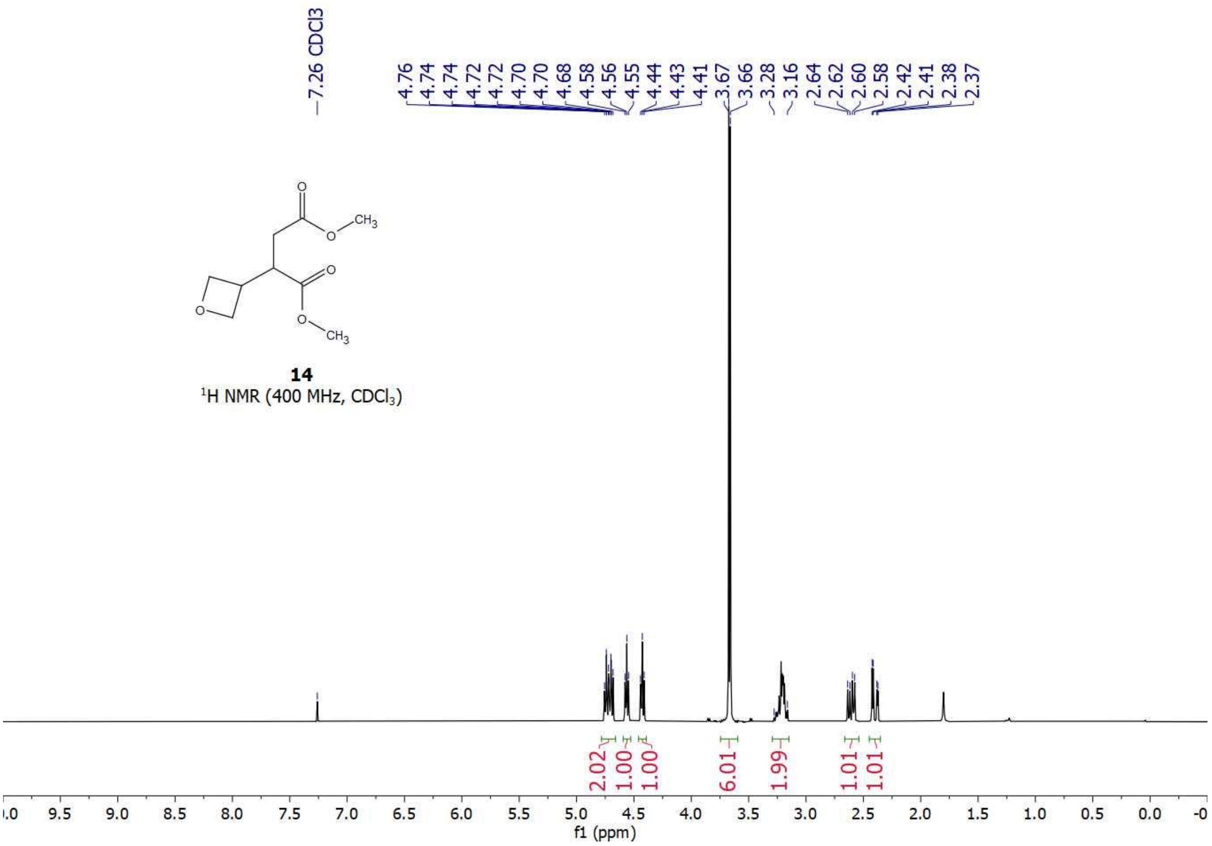

14
^{1}H NMR (400 MHz, $CDCl_3$)

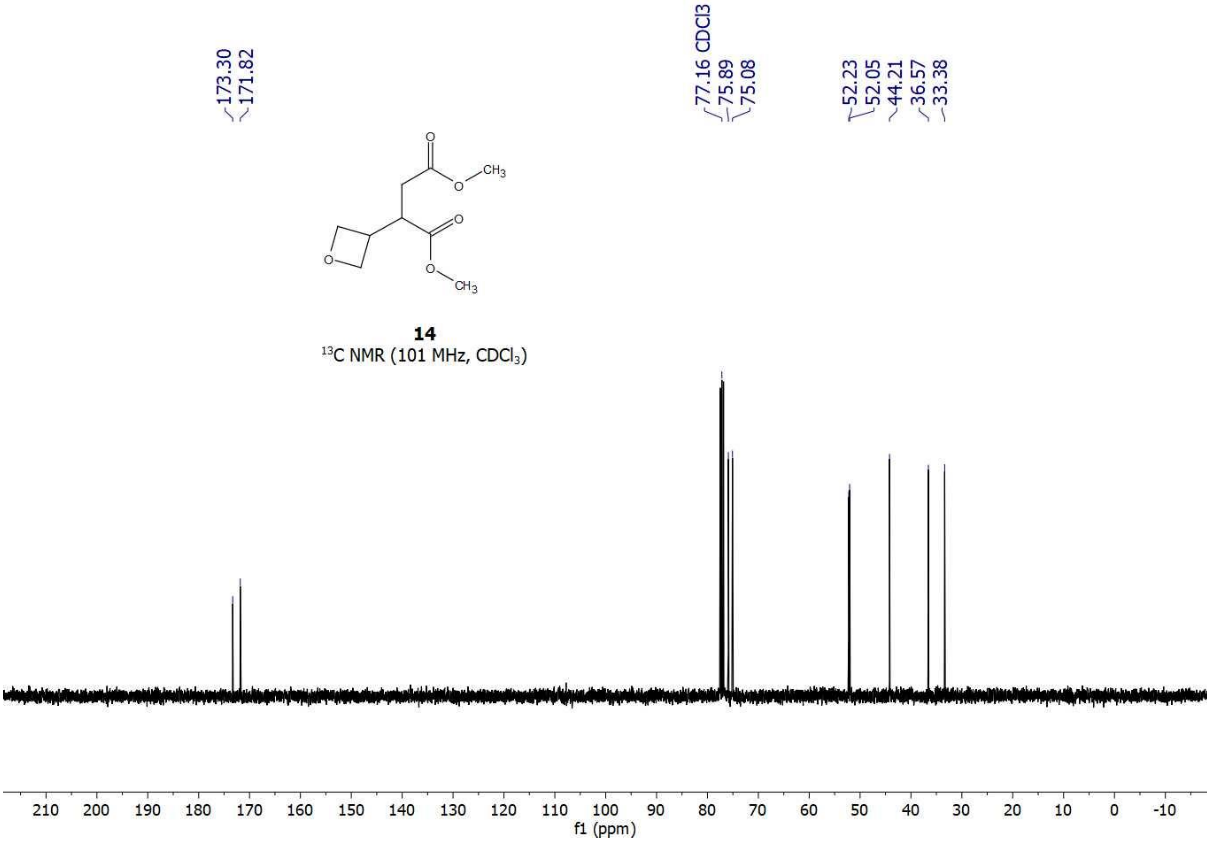

14
^{13}C NMR (101 MHz, $CDCl_3$)

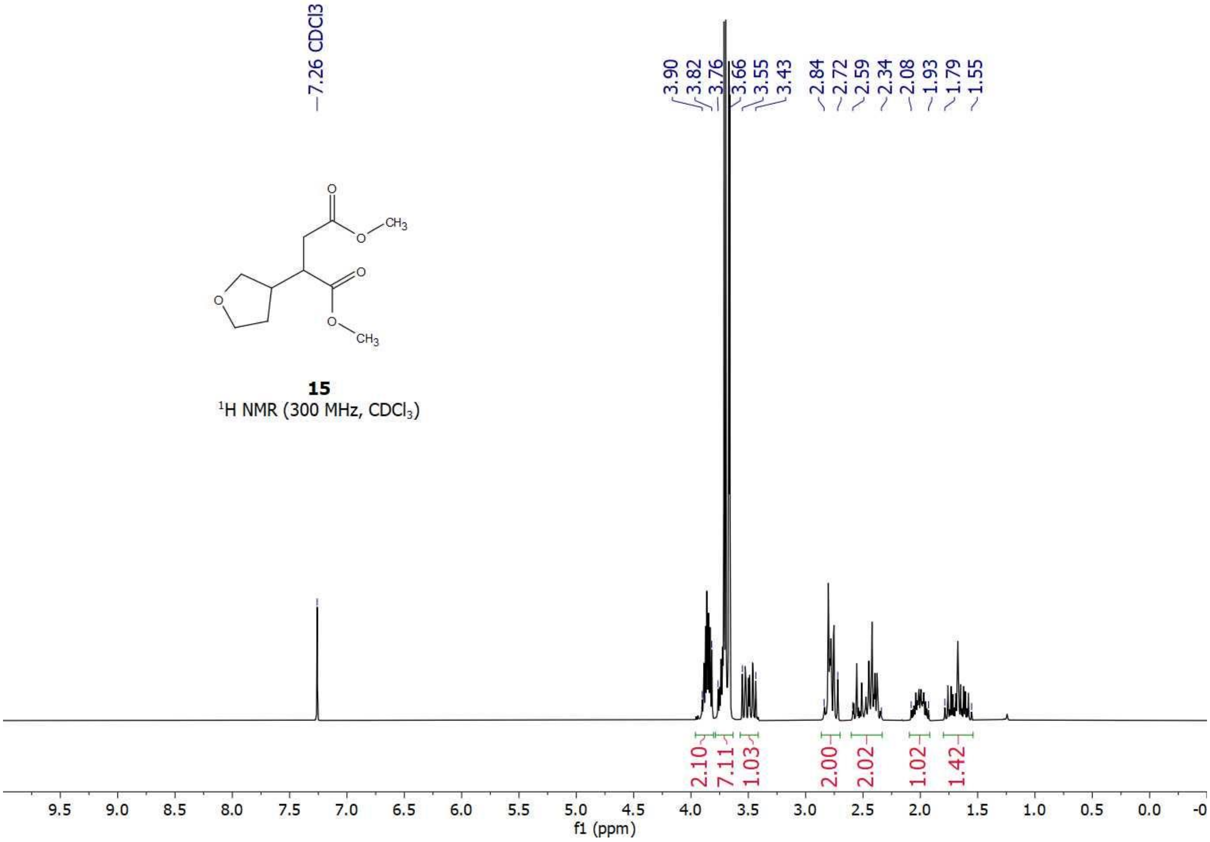

15

^{1}H NMR (300 MHz, $CDCl_3$)

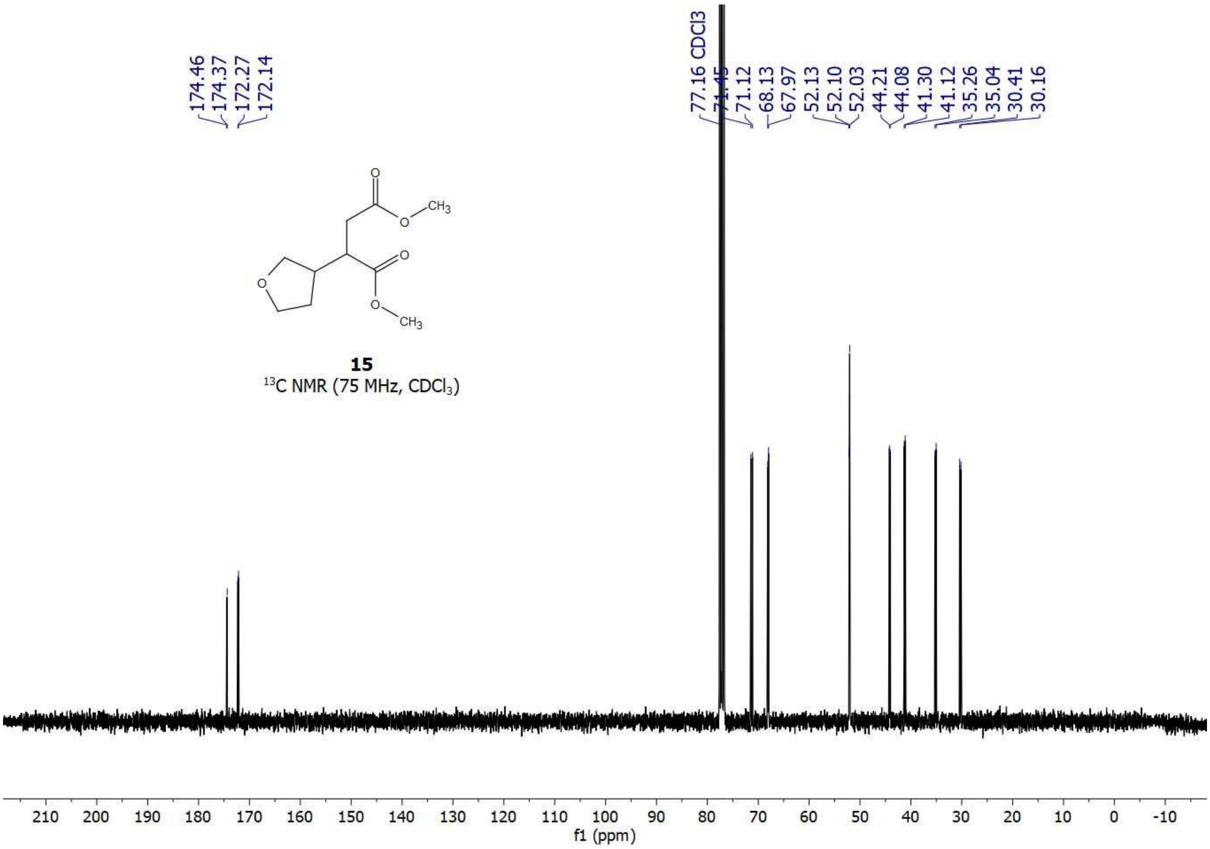

15

^{13}C NMR (75 MHz, $CDCl_3$)

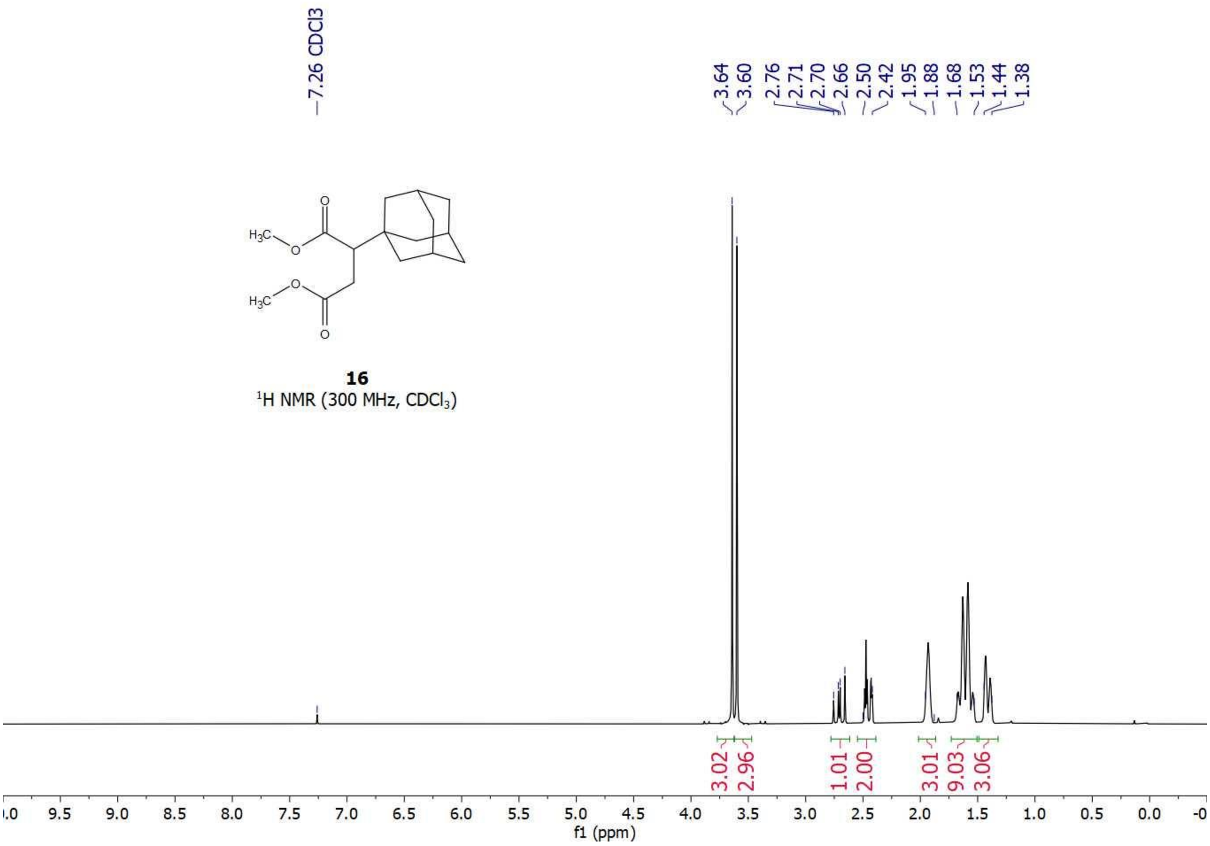

16
^{1}H NMR (300 MHz, $CDCl_3$)

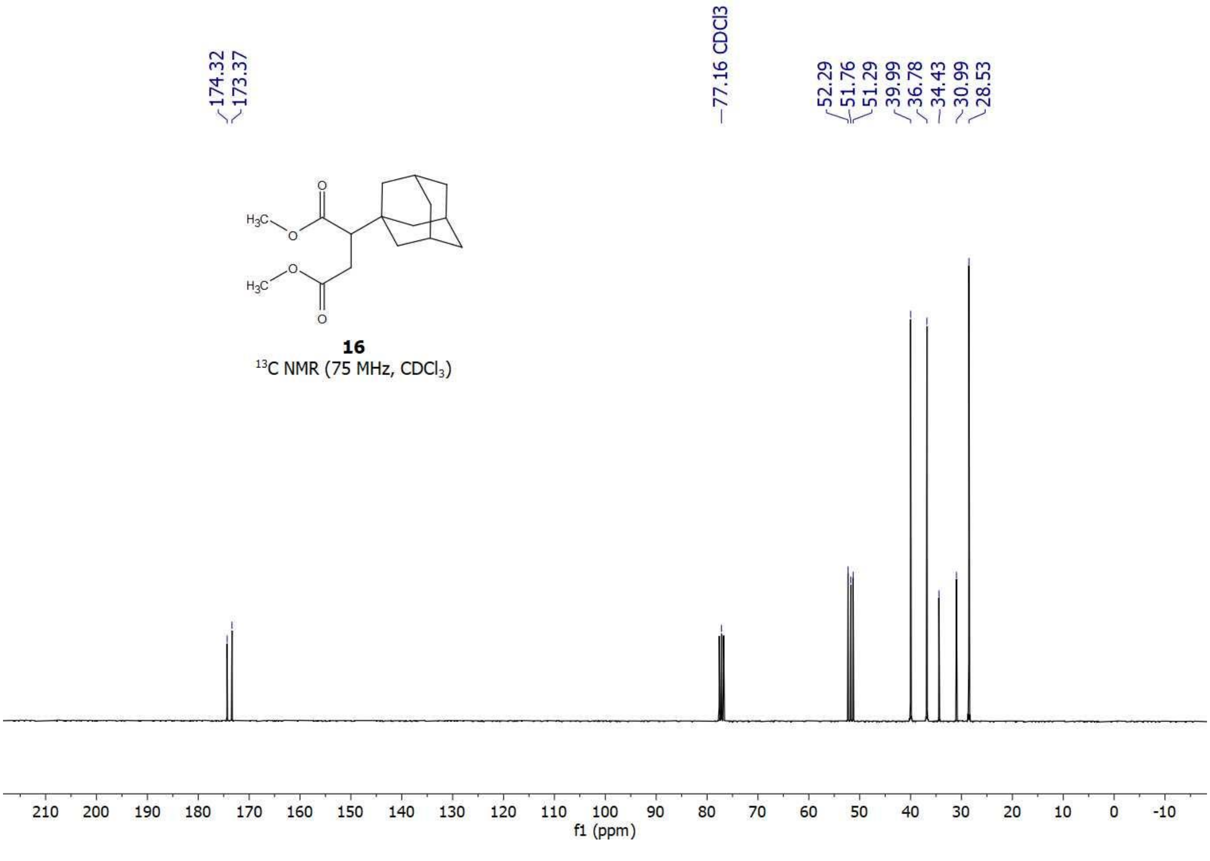

16
^{13}C NMR (75 MHz, $CDCl_3$)

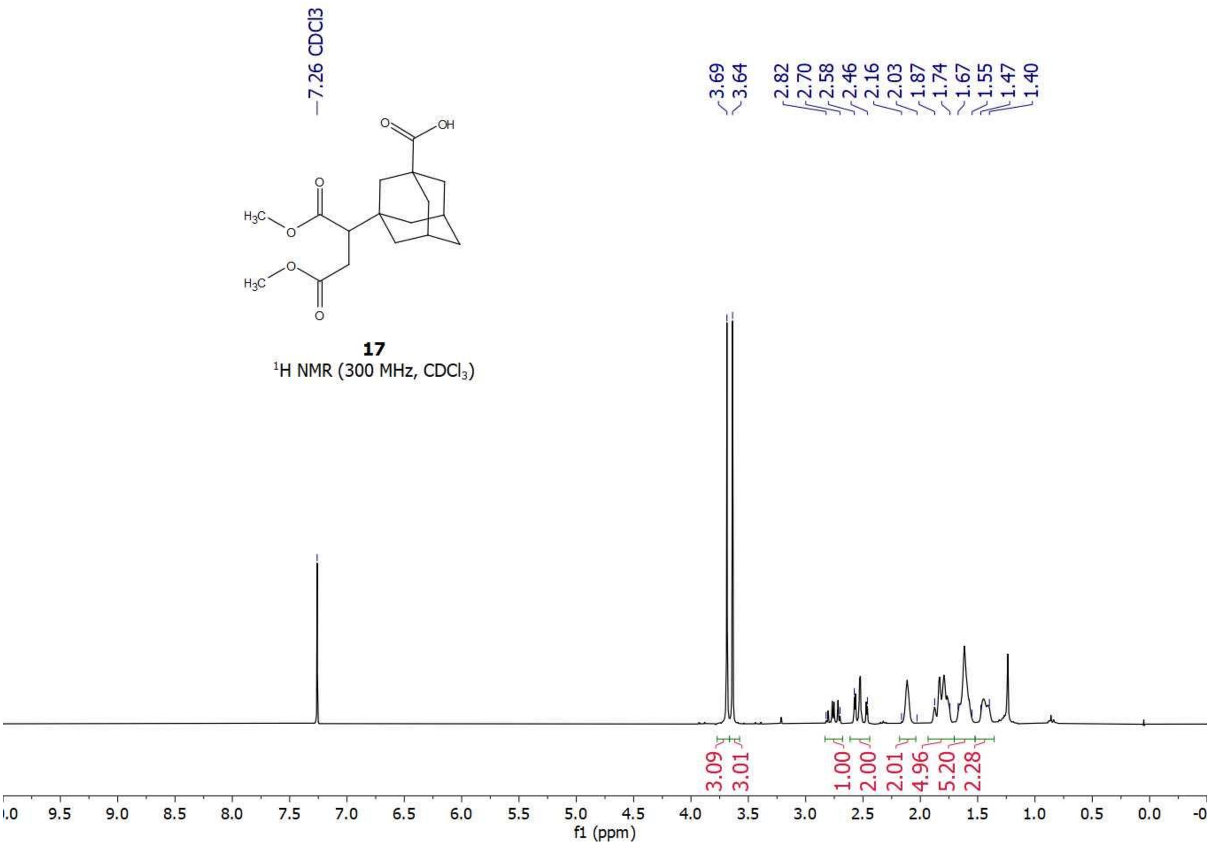

17

^{1}H NMR (300 MHz, $CDCl_3$)

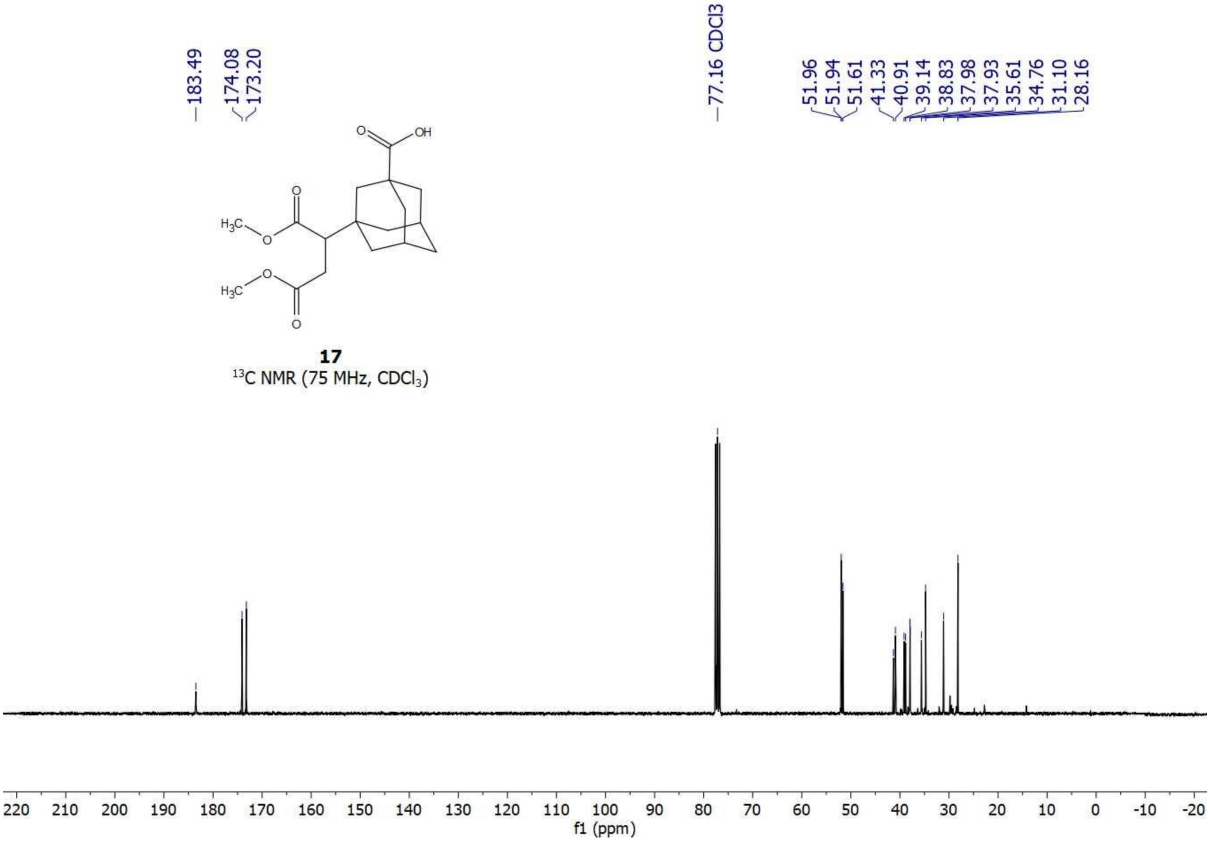

17

^{13}C NMR (75 MHz, $CDCl_3$)

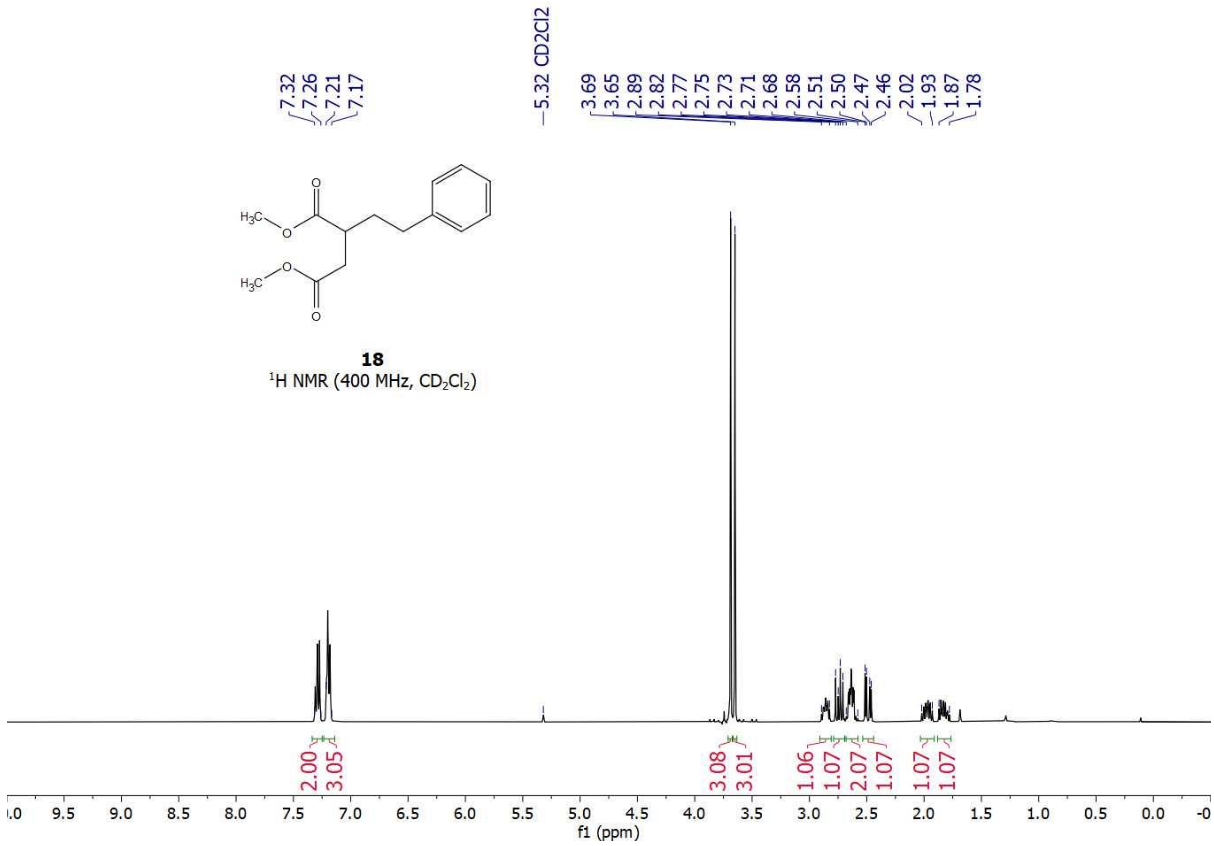

18
^{1}H NMR (400 MHz, CD_2Cl_2)

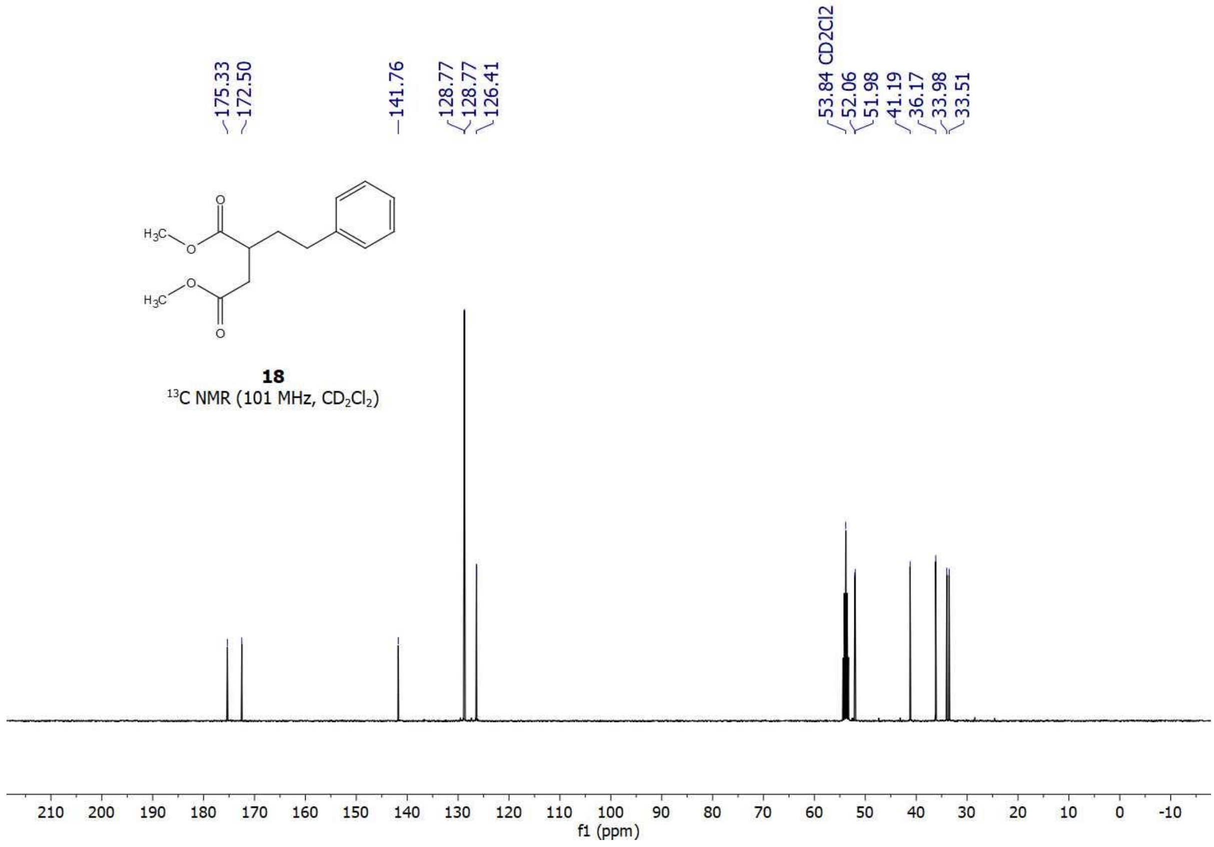

18
^{13}C NMR (101 MHz, CD_2Cl_2)

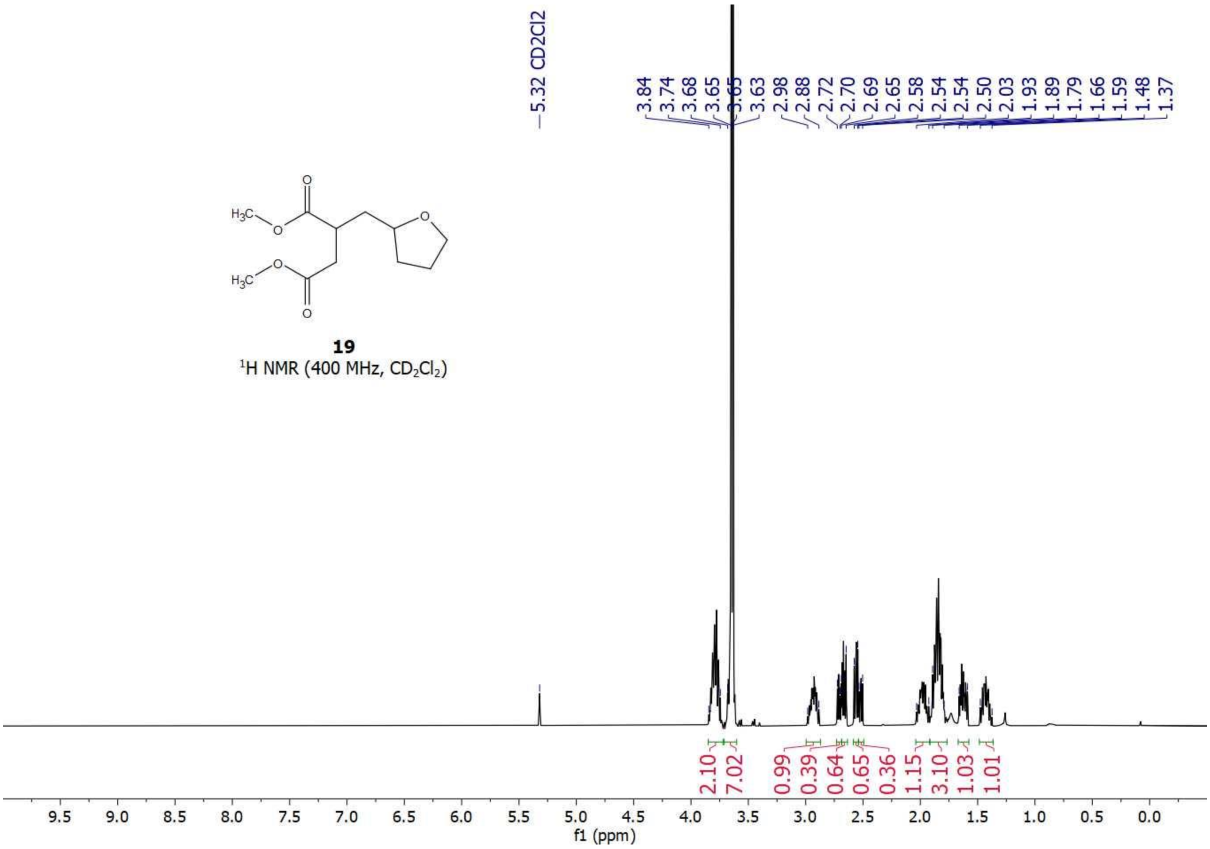

5.32 CD2Cl2
3.84
3.74
3.68
3.65
3.65
3.63
2.98
2.88
2.72
2.70
2.69
2.65
2.58
2.54
2.54
2.50
2.03
1.93
1.89
1.79
1.66
1.59
1.48
1.37
H_3C
O
O
O
O
H_3C
O
19
^1H NMR (400 MHz, CD_2Cl_2)
2.10
7.02
0.99
0.39
0.64
0.65
0.36
1.15
3.10
1.03
1.01
9.5 9.0 8.5 8.0 7.5 7.0 6.5 6.0 5.5 5.0 4.5 4.0 3.5 3.0 2.5 2.0 1.5 1.0 0.5 0.0
f1 (ppm)

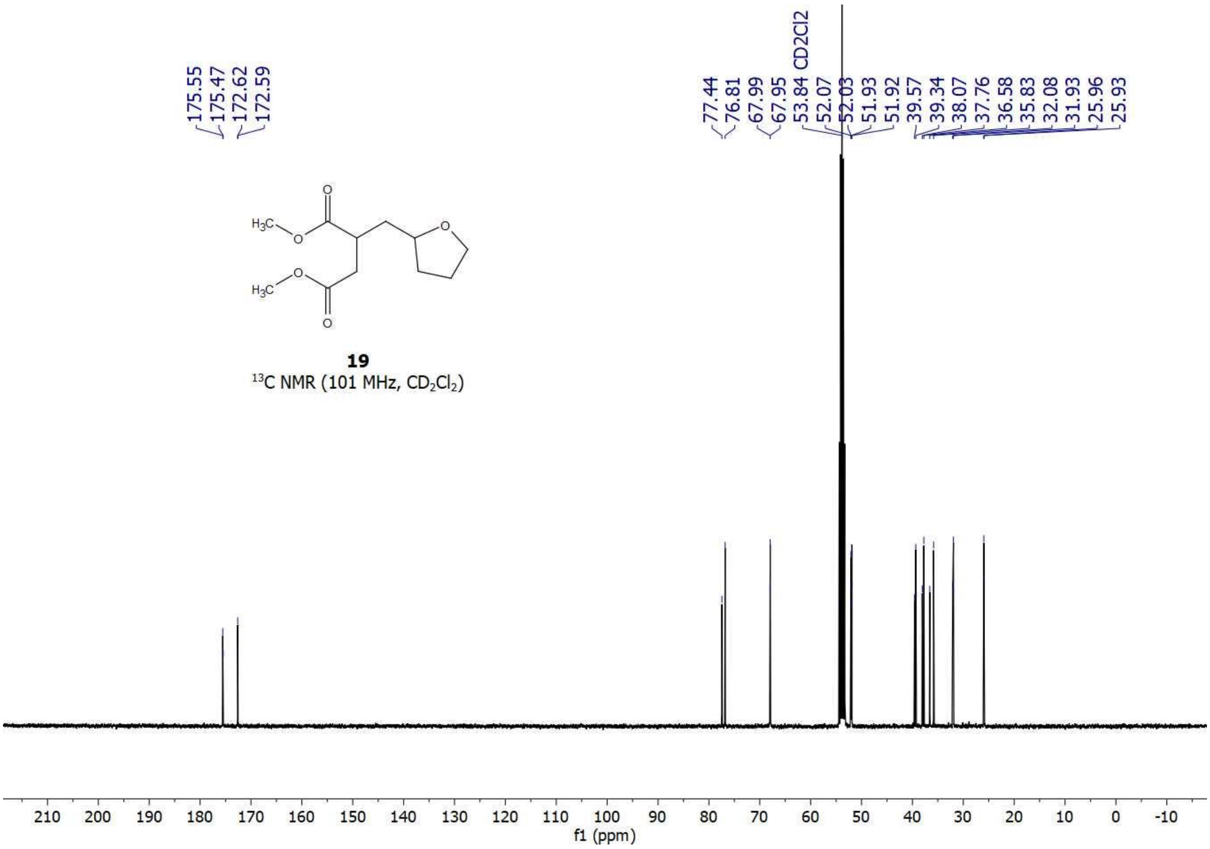

175.55
175.47
172.62
172.59
77.44
76.81
67.99
67.95
53.84 CD2Cl2
52.07
52.03
51.93
51.92
39.57
39.34
38.07
37.76
36.58
35.83
32.08
31.93
25.96
25.93
H_3C
O
O
O
O
H_3C
O
19
^{13}C NMR (101 MHz, CD_2Cl_2)
210 200 190 180 170 160 150 140 130 120 110 100 90 80 70 60 50 40 30 20 10 0 -10
f1 (ppm)

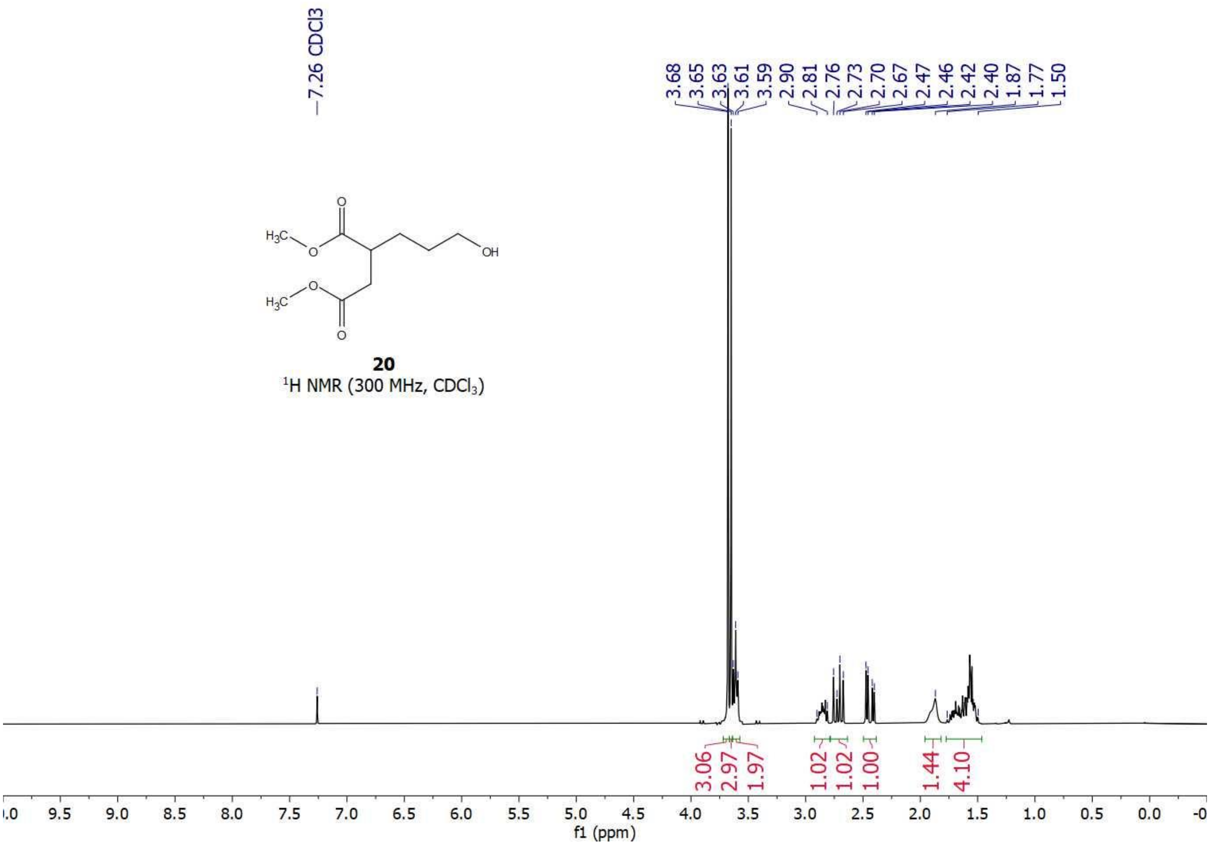

7.26 CDCl3
3.68
3.65
3.63
3.61
3.59
2.90
2.81
2.76
2.73
2.70
2.67
2.47
2.46
2.42
2.40
1.87
1.77
1.50
20
^{1}H NMR (300 MHz, CDCl$_3$)
3.06
2.97
1.97
1.02
1.02
1.00
1.44
4.10
f1 (ppm)

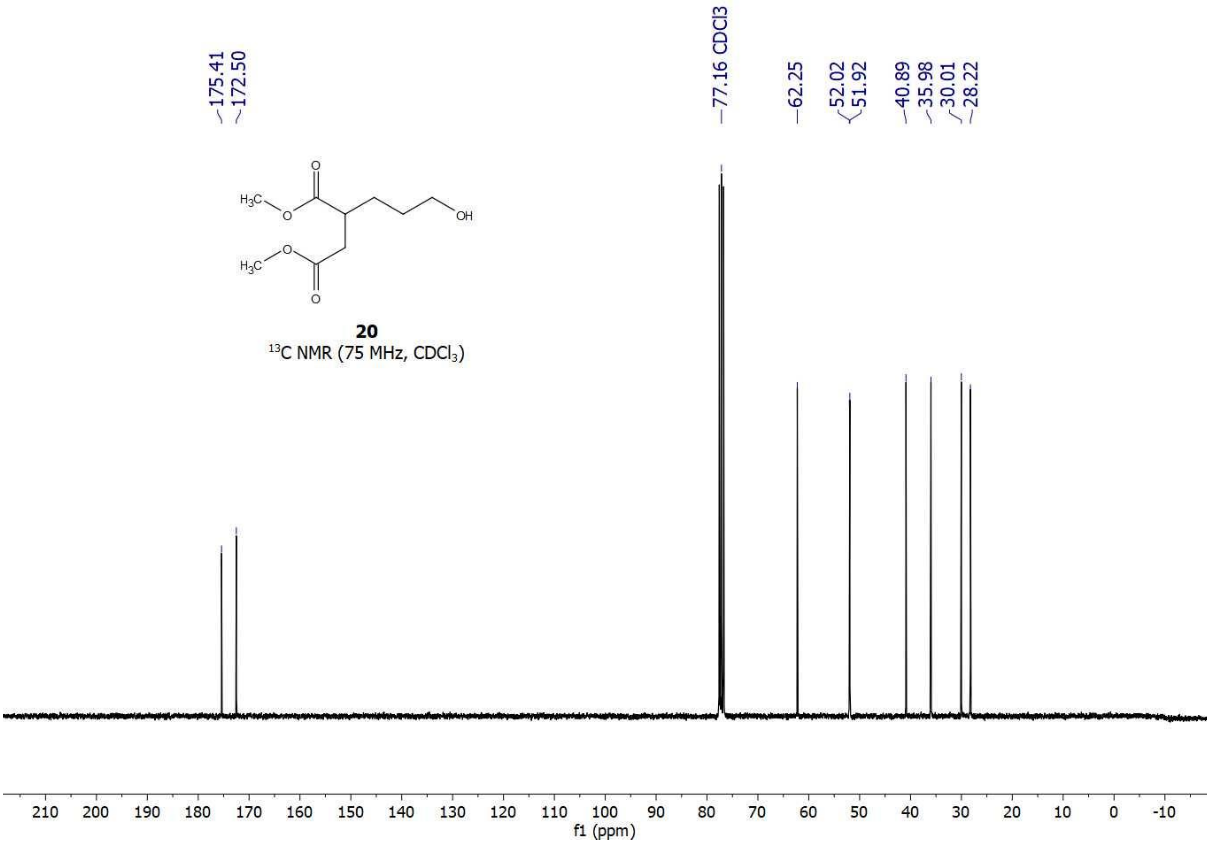

175.41
172.50
77.16 CDCl3
62.25
52.02
51.92
40.89
35.98
30.01
28.22
20
^{13}C NMR (75 MHz, CDCl$_3$)
f1 (ppm)

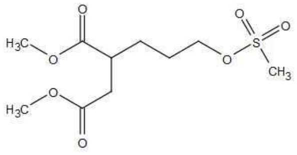

21
^{1}H NMR (400 MHz, $CDCl_3$)

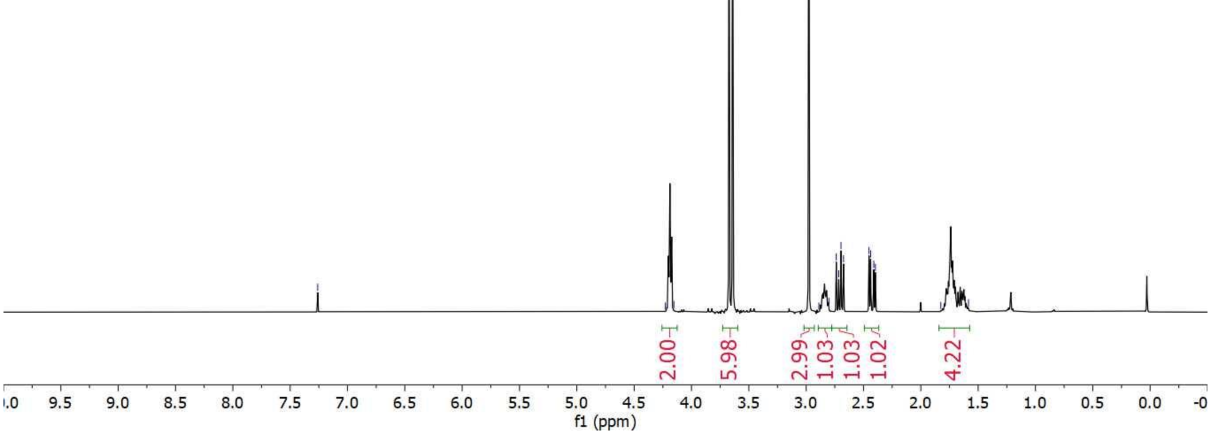

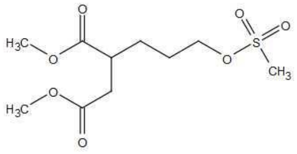

21
^{13}C NMR (101 MHz, $CDCl_3$)

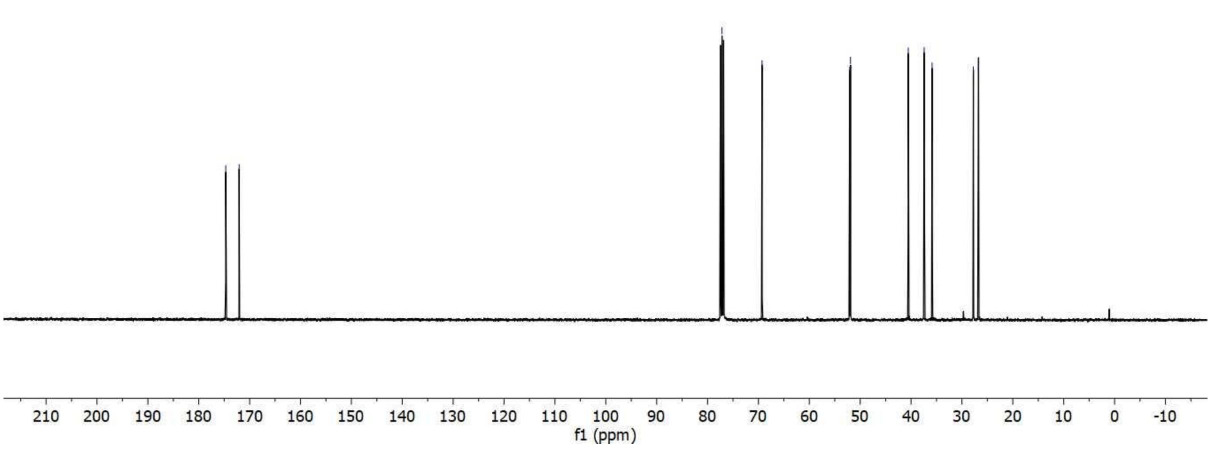

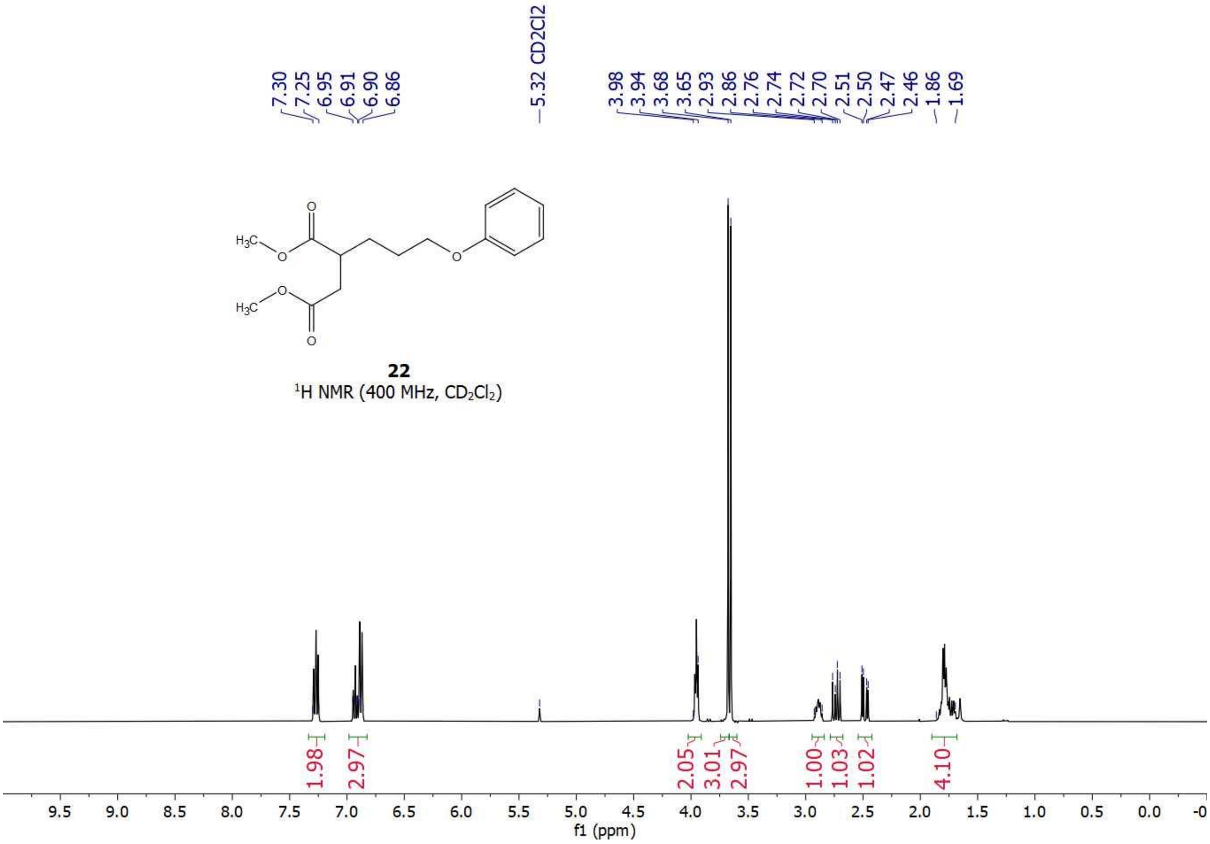

7.30
7.25
6.95
6.91
6.90
6.86
5.32 CD2Cl2
3.98
3.94
3.68
3.65
2.93
2.86
2.76
2.74
2.72
2.70
2.51
2.50
2.47
2.46
1.86
1.69
H3C
O
O
O
H3C
O
O
O
22
1H NMR (400 MHz, CD2Cl2)
1.98
2.97
2.05
3.01
2.97
1.00
1.03
1.02
4.10
9.5 9.0 8.5 8.0 7.5 7.0 6.5 6.0 5.5 5.0 4.5 4.0 3.5 3.0 2.5 2.0 1.5 1.0 0.5 0.0 -0
f1 (ppm)

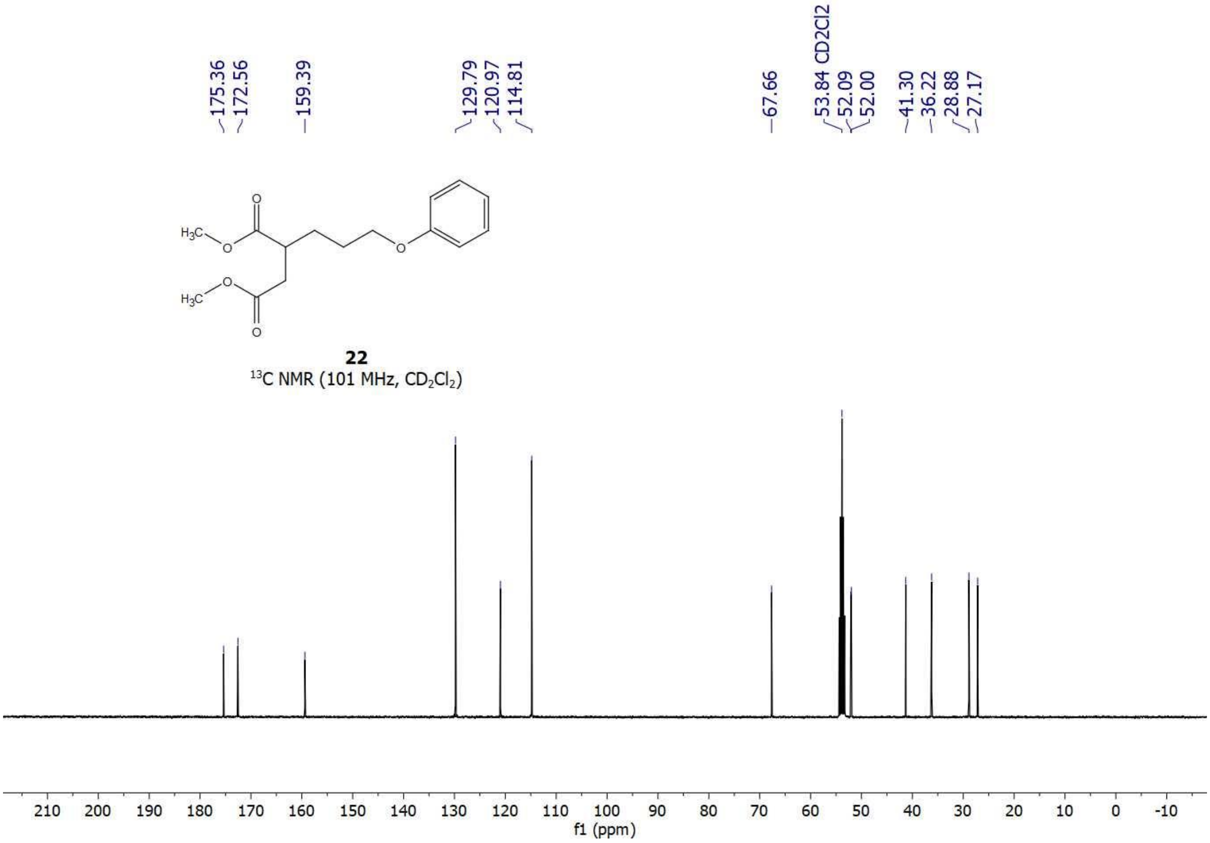

175.36
172.56
159.39
129.79
120.97
114.81
67.66
53.84 CD2Cl2
52.09
52.00
41.30
36.22
28.88
27.17
H3C
O
O
O
H3C
O
O
O
22
13C NMR (101 MHz, CD2Cl2)
210 200 190 180 170 160 150 140 130 120 110 100 90 80 70 60 50 40 30 20 10 0 -10
f1 (ppm)

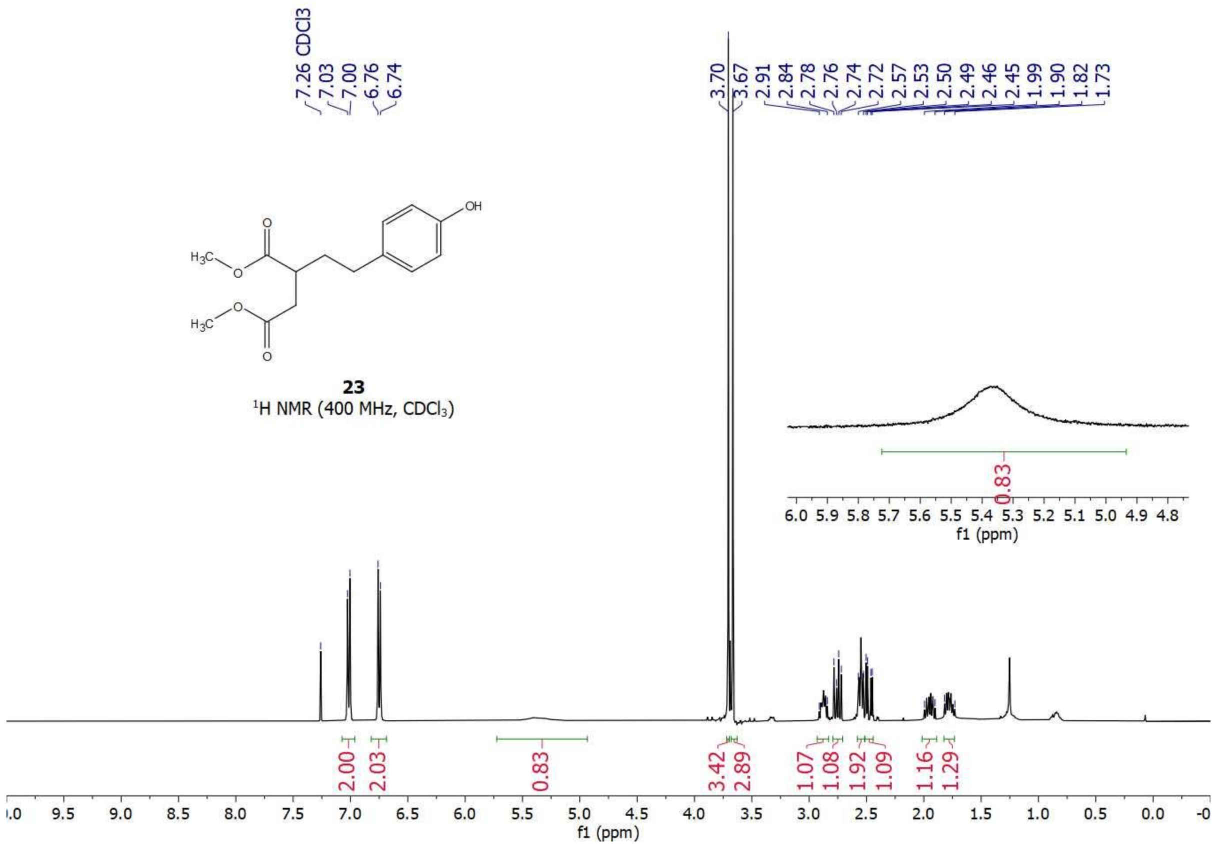
7.26 CDCl3
7.03
7.00
6.76
6.74
3.70
3.67
2.91
2.84
2.78
2.76
2.74
2.72
2.57
2.53
2.50
2.49
2.46
2.45
1.99
1.90
1.82
1.73
OH
O
H_3C
O
H_3C
O
O
23
^{1}H NMR (400 MHz, $CDCl_3$)
0.83
6.0 5.9 5.8 5.7 5.6 5.5 5.4 5.3 5.2 5.1 5.0 4.9 4.8
f1 (ppm)
2.00
2.03
0.83
3.42
2.89
1.07
1.08
1.92
1.09
1.16
1.29
9.5 9.0 8.5 8.0 7.5 7.0 6.5 6.0 5.5 5.0 4.5 4.0 3.5 3.0 2.5 2.0 1.5 1.0 0.5 0.0
f1 (ppm)

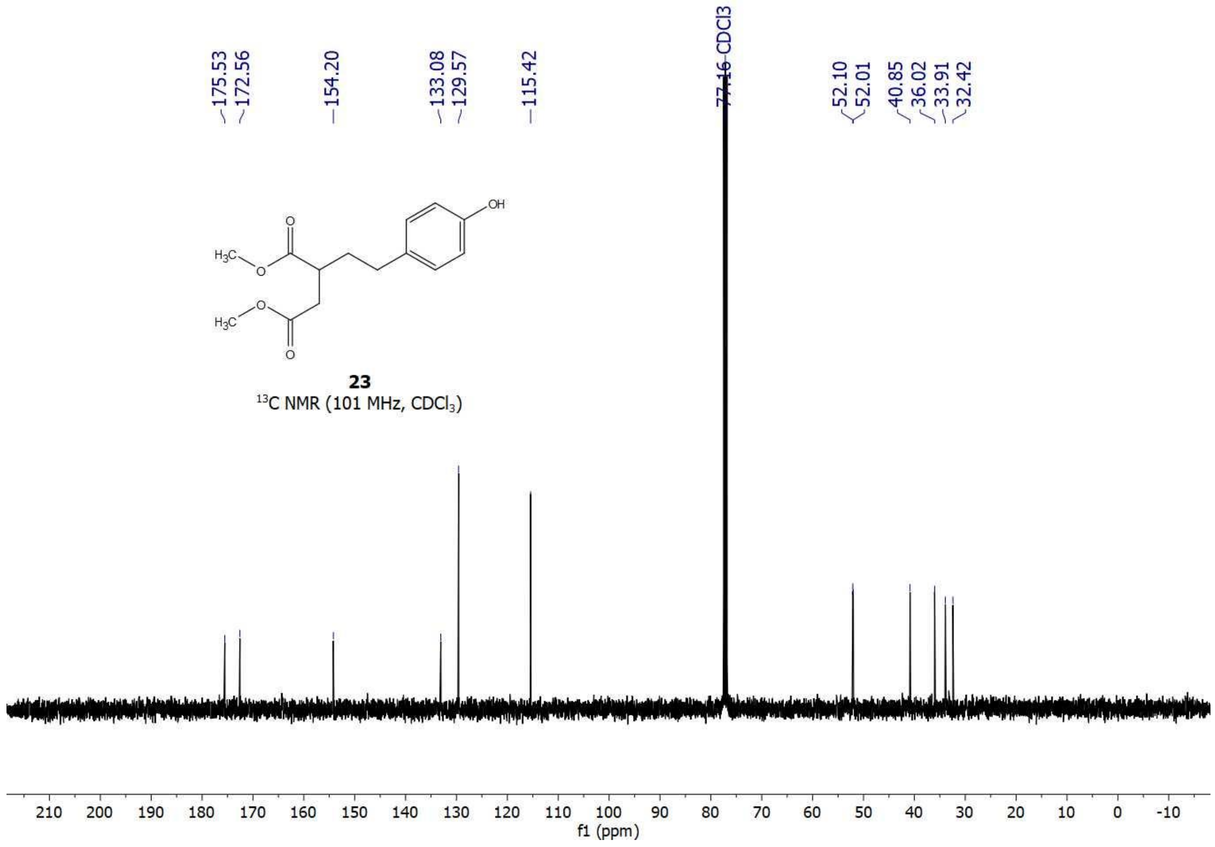
175.53
172.56
154.20
133.08
129.57
115.42
77.16 CDCl3
52.10
52.01
40.85
36.02
33.91
32.42
OH
O
H_3C
O
H_3C
O
O
23
^{13}C NMR (101 MHz, $CDCl_3$)
210 200 190 180 170 160 150 140 130 120 110 100 90 80 70 60 50 40 30 20 10 0 -10
f1 (ppm)

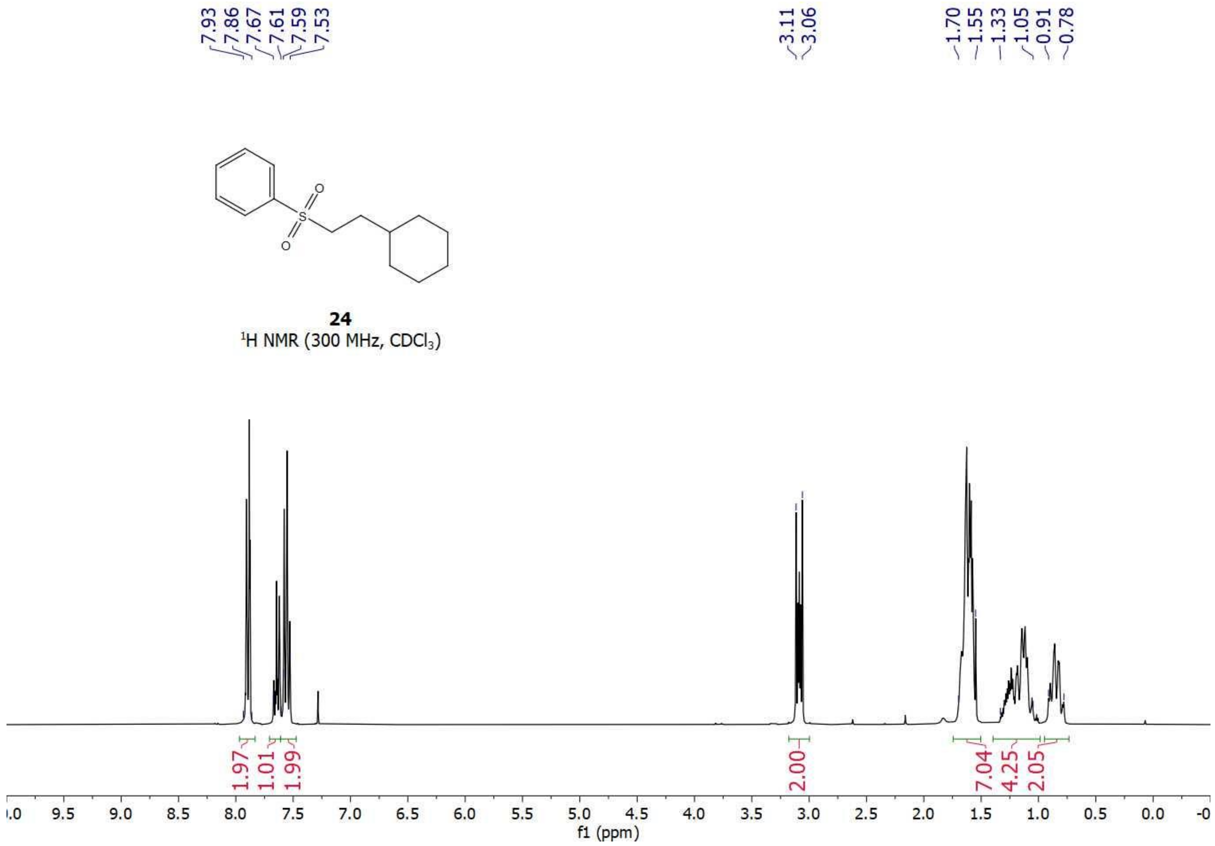

24
^{1}H NMR (300 MHz, $CDCl_3$)

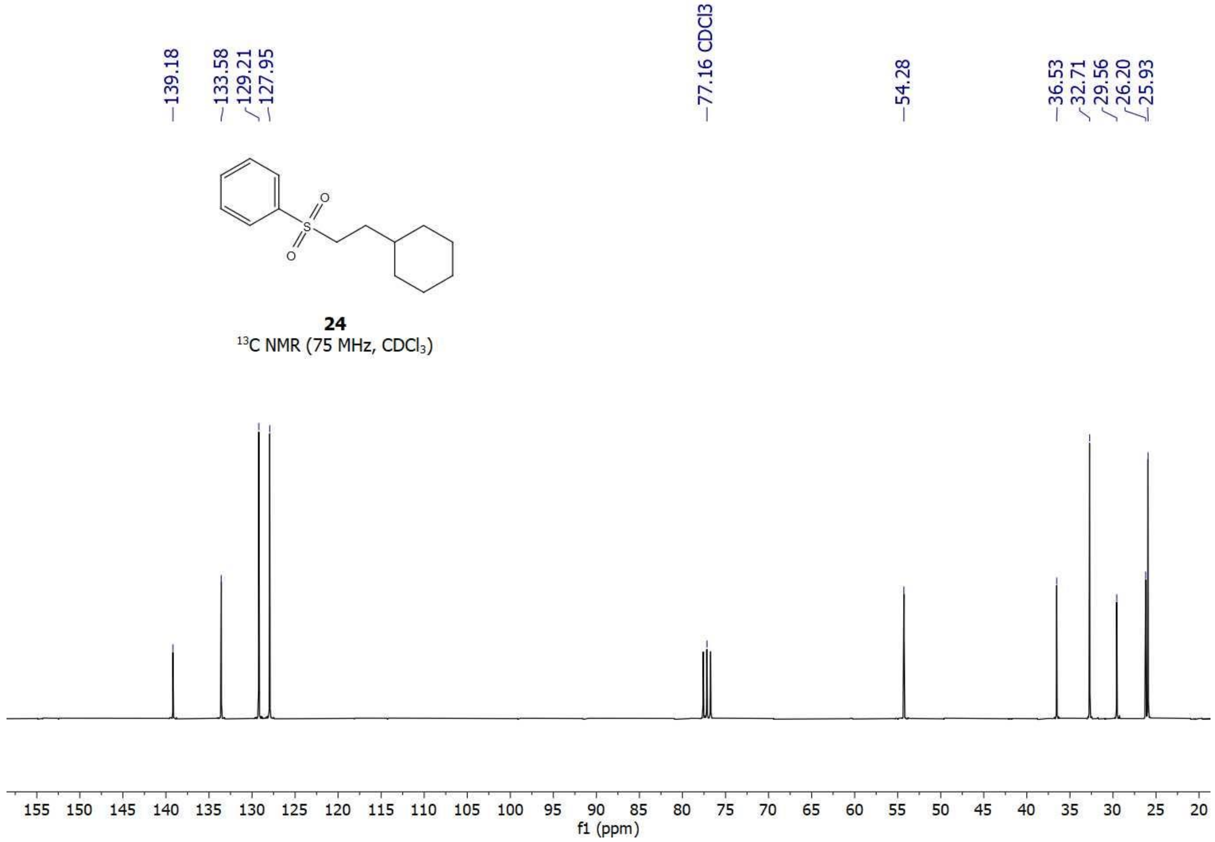

24
^{13}C NMR (75 MHz, $CDCl_3$)

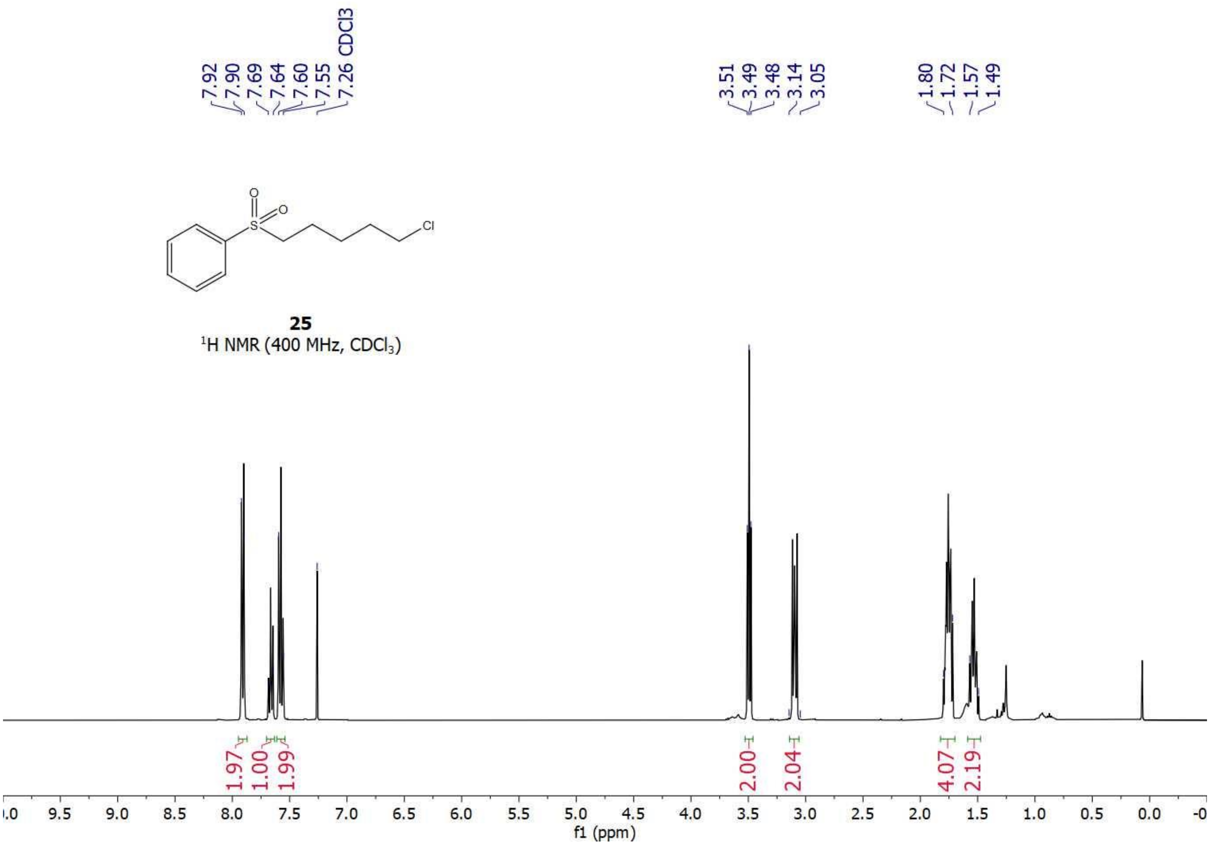
7.92
7.90
7.69
7.64
7.60
7.55
7.26 CDCl3
3.51
3.49
3.48
3.14
3.05
1.80
1.72
1.57
1.49
Cl
25
1H NMR (400 MHz, CDCl3)
1.97
1.00
1.99
2.00
2.04
4.07
2.19
f1 (ppm)

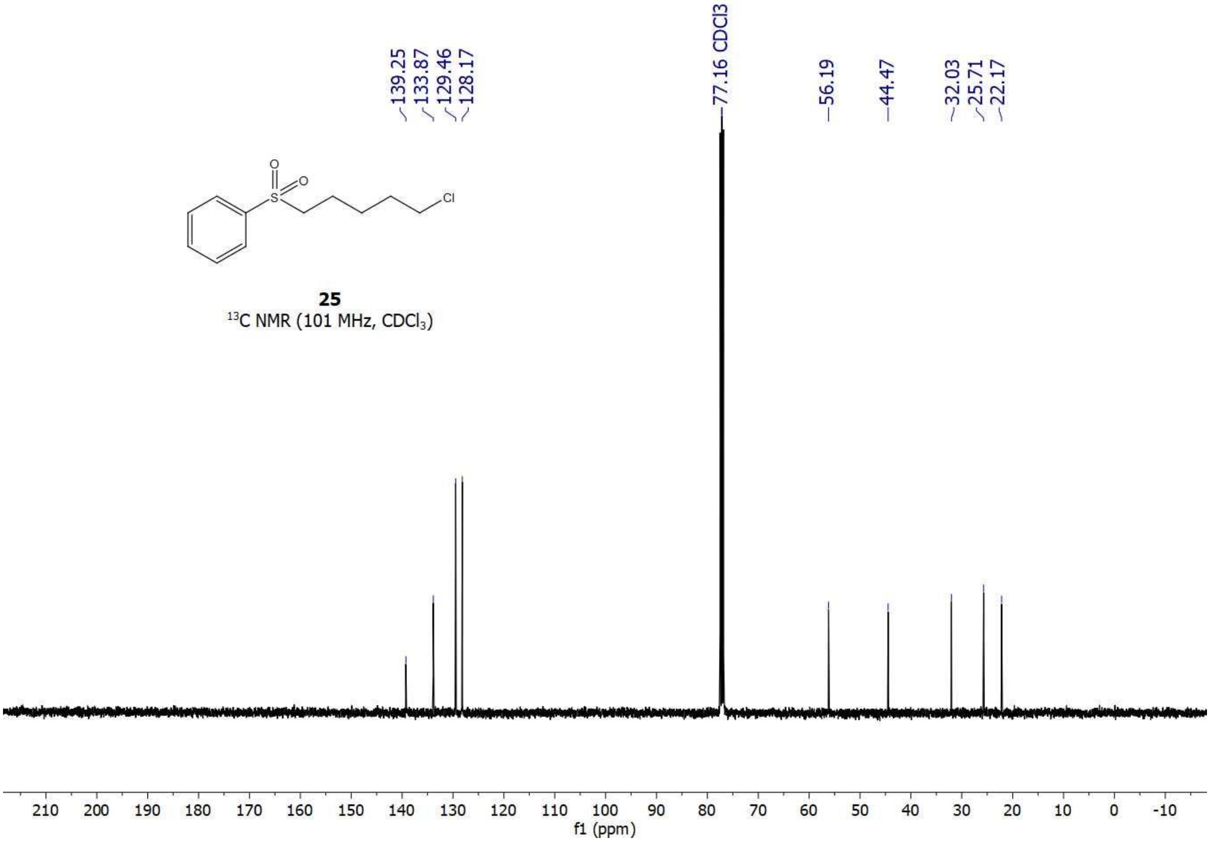
139.25
133.87
129.46
128.17
77.16 CDCl3
56.19
44.47
32.03
25.71
22.17
Cl
25
13C NMR (101 MHz, CDCl3)
f1 (ppm)

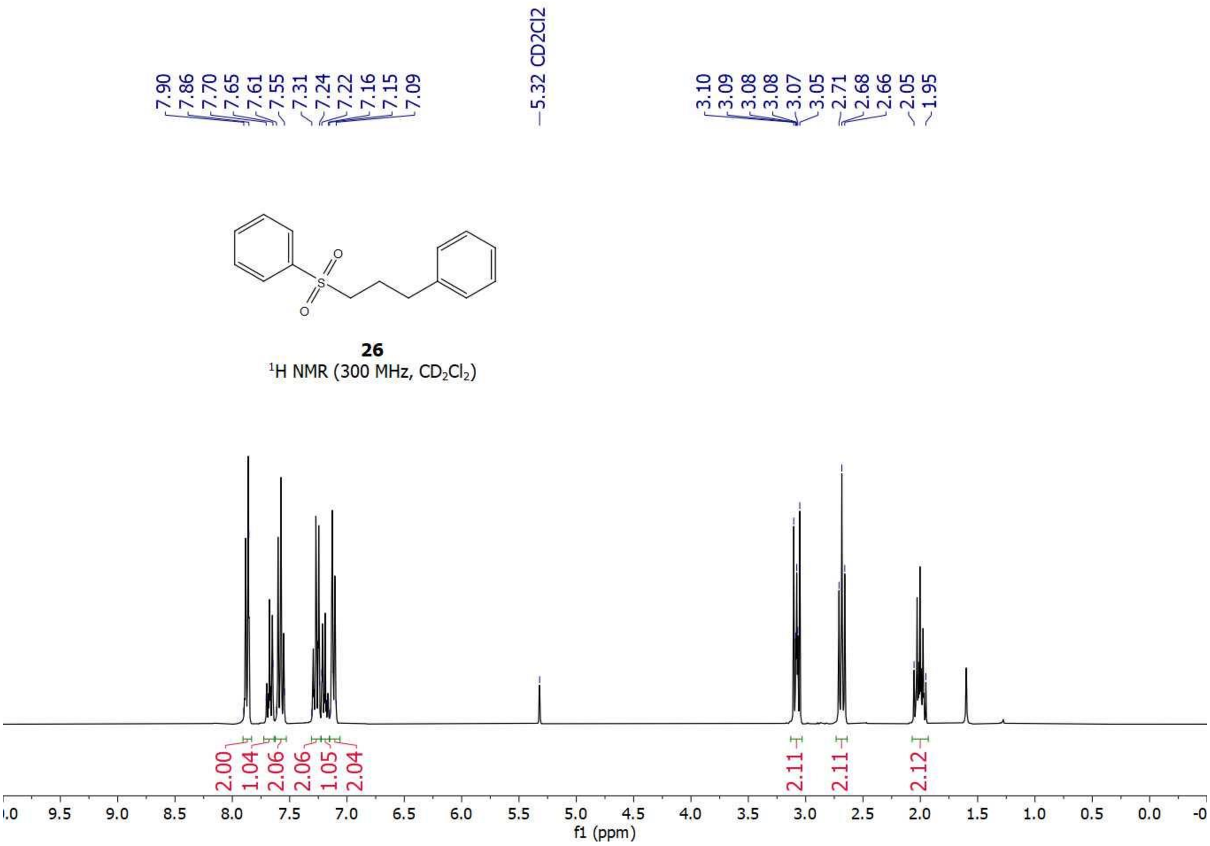
7.90
7.86
7.70
7.65
7.61
7.55
7.31
7.24
7.22
7.16
7.15
7.09
5.32 CD2Cl2
3.10
3.09
3.08
3.08
3.07
3.05
2.71
2.68
2.66
2.05
1.95
26
1H NMR (300 MHz, CD2Cl2)
2.00
1.04
2.06
2.06
1.05
2.04
2.11
2.11
2.12
f1 (ppm)

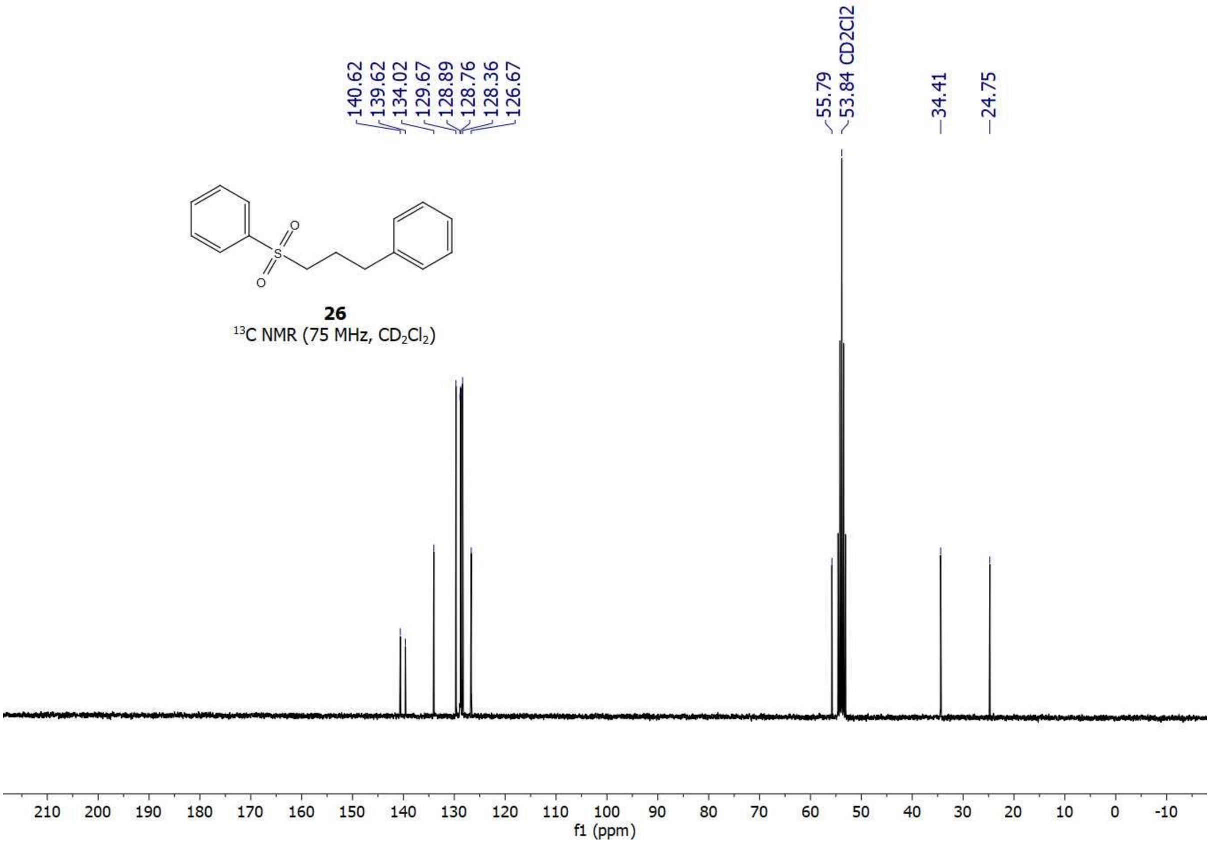
140.62
139.62
134.02
129.67
128.89
128.76
128.36
126.67
55.79
53.84 CD2Cl2
34.41
24.75
26
13C NMR (75 MHz, CD2Cl2)
f1 (ppm)

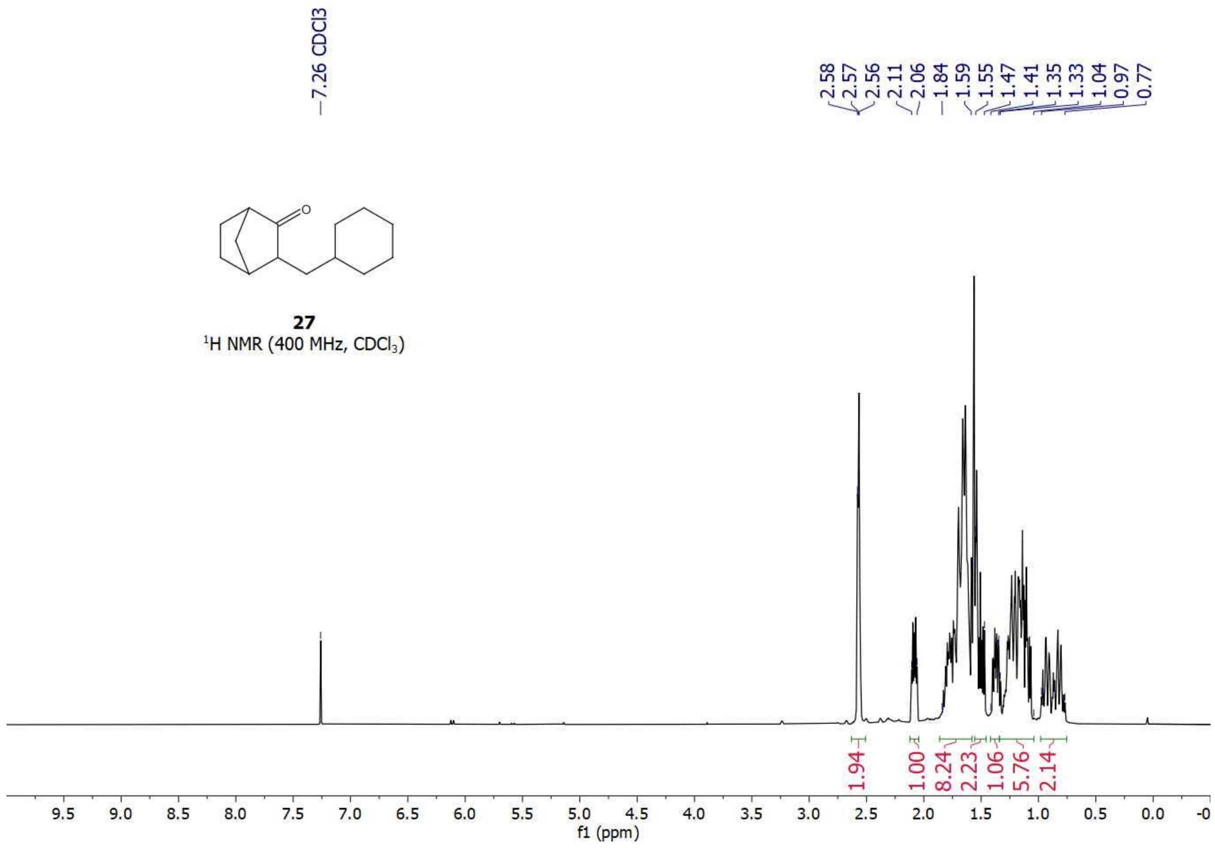
7.26 CDCl3
2.58
2.57
2.56
2.11
2.06
1.84
1.59
1.55
1.47
1.41
1.35
1.33
1.04
0.97
0.77
27
1H NMR (400 MHz, CDCl3)
1.94
1.00
8.24
2.23
1.06
5.76
2.14
9.5 9.0 8.5 8.0 7.5 7.0 6.5 6.0 5.5 5.0 4.5 4.0 3.5 3.0 2.5 2.0 1.5 1.0 0.5 0.0 -0
f1 (ppm)

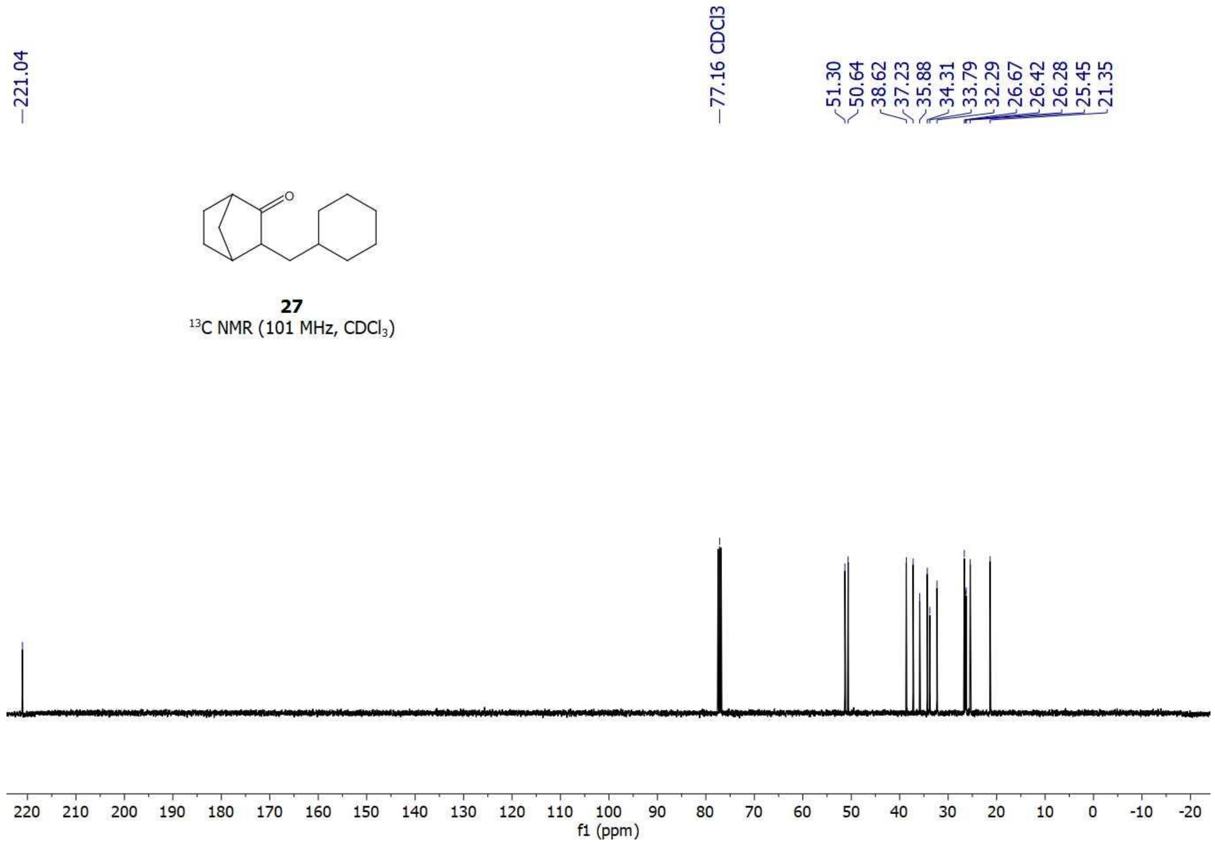
221.04
77.16 CDCl3
51.30
50.64
38.62
37.23
35.88
34.31
33.79
32.29
26.67
26.42
26.28
25.45
21.35
27
13C NMR (101 MHz, CDCl3)
220 210 200 190 180 170 160 150 140 130 120 110 100 90 80 70 60 50 40 30 20 10 0 -10 -20
f1 (ppm)

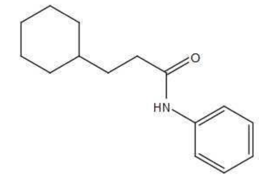

28
^{1}H NMR (300 MHz, CD_2Cl_2)

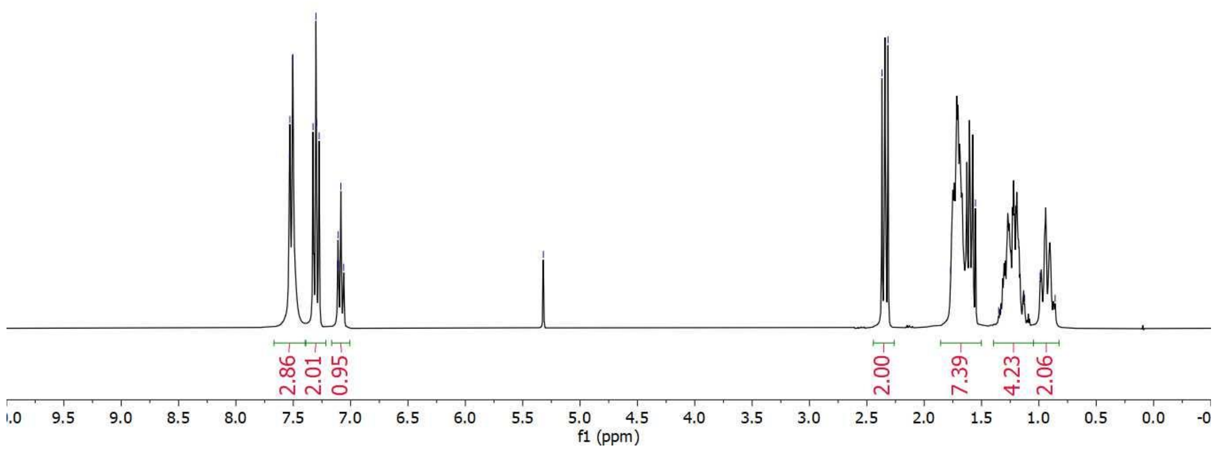

28
^{13}C NMR (75 MHz, CD_2Cl_2)

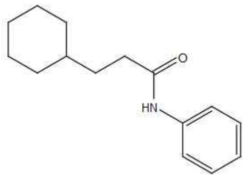

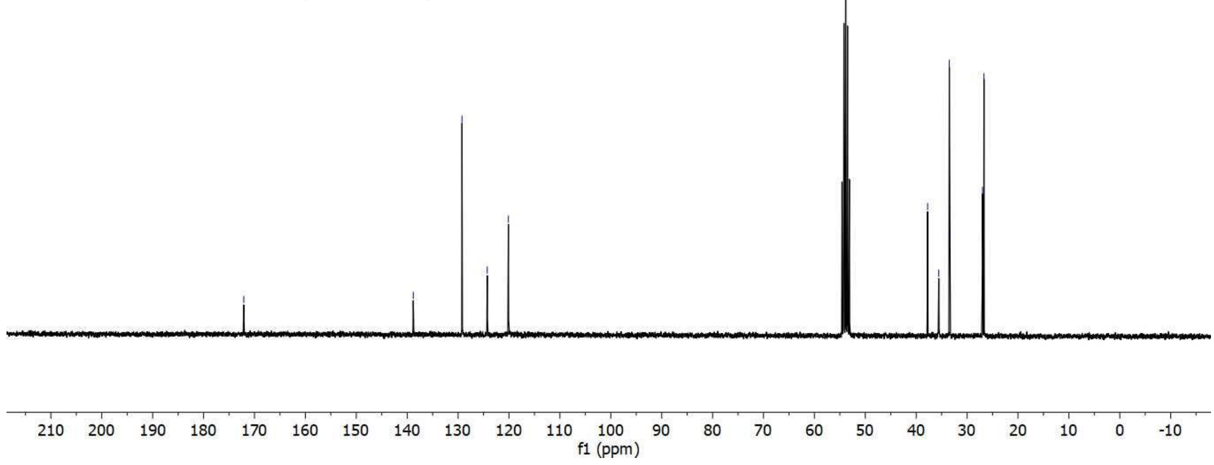

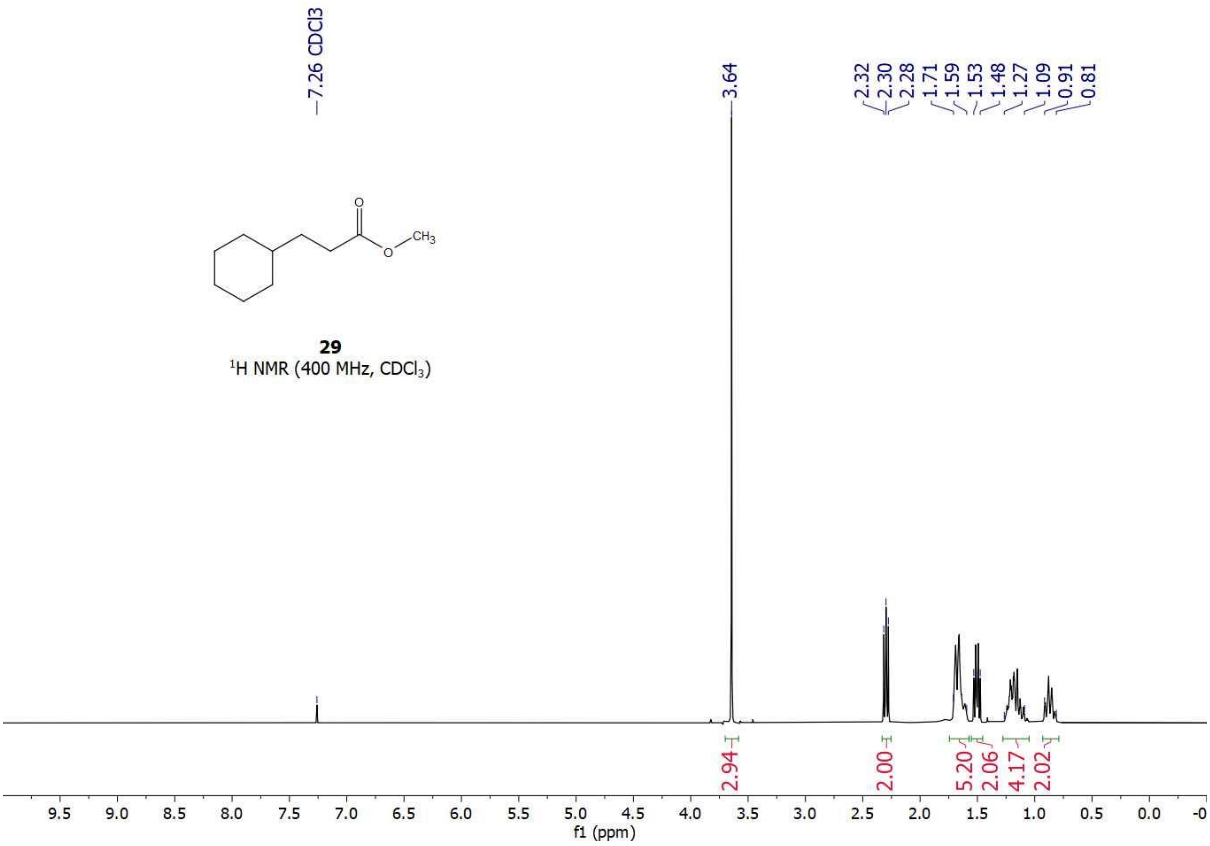

29
^{1}H NMR (400 MHz, $CDCl_3$)

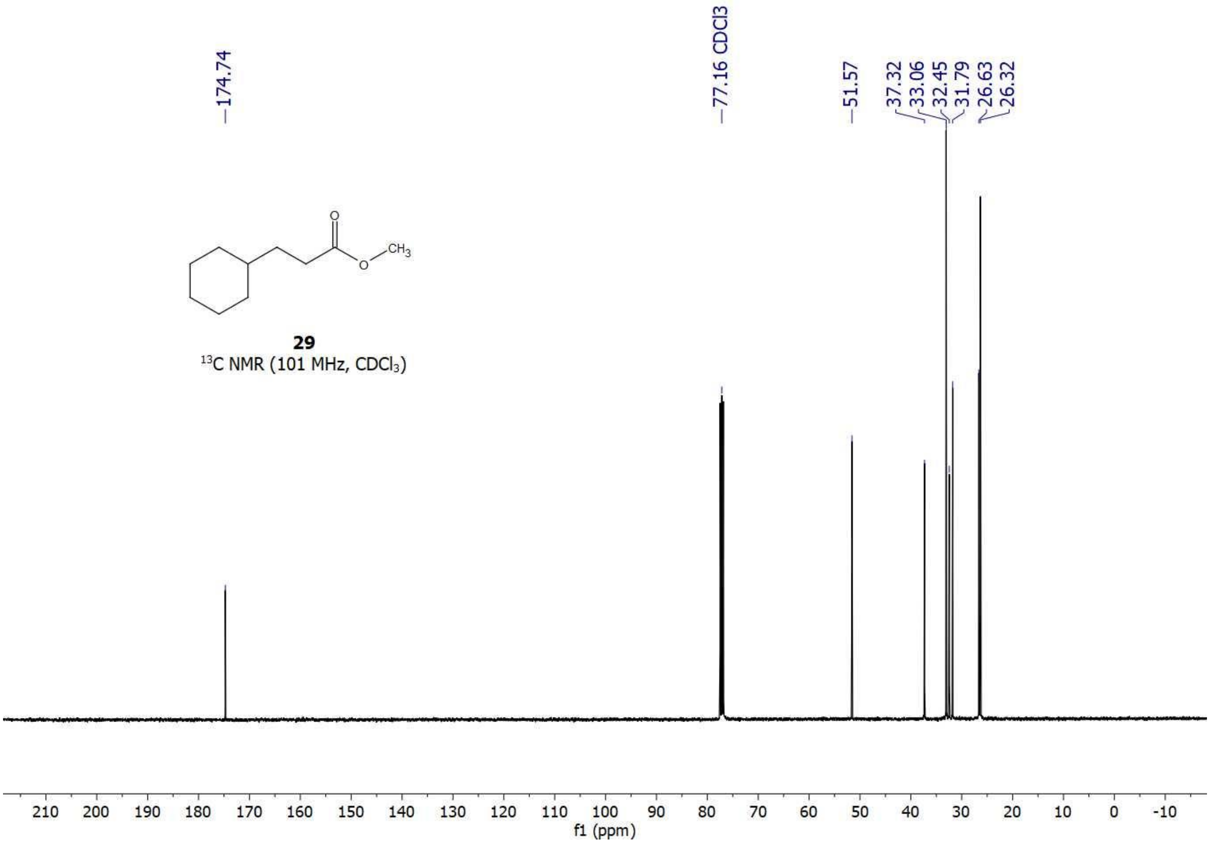

29
^{13}C NMR (101 MHz, $CDCl_3$)

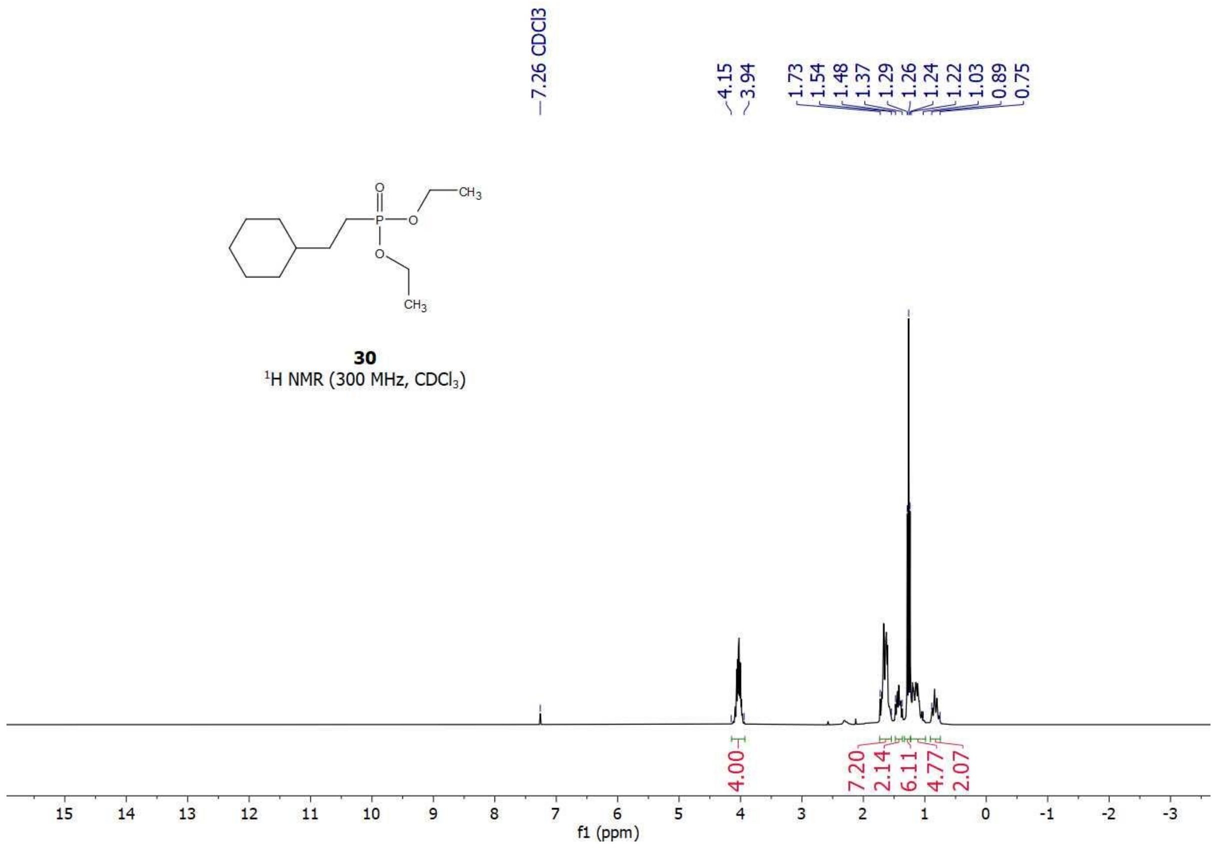

30
^{1}H NMR (300 MHz, $CDCl_3$)

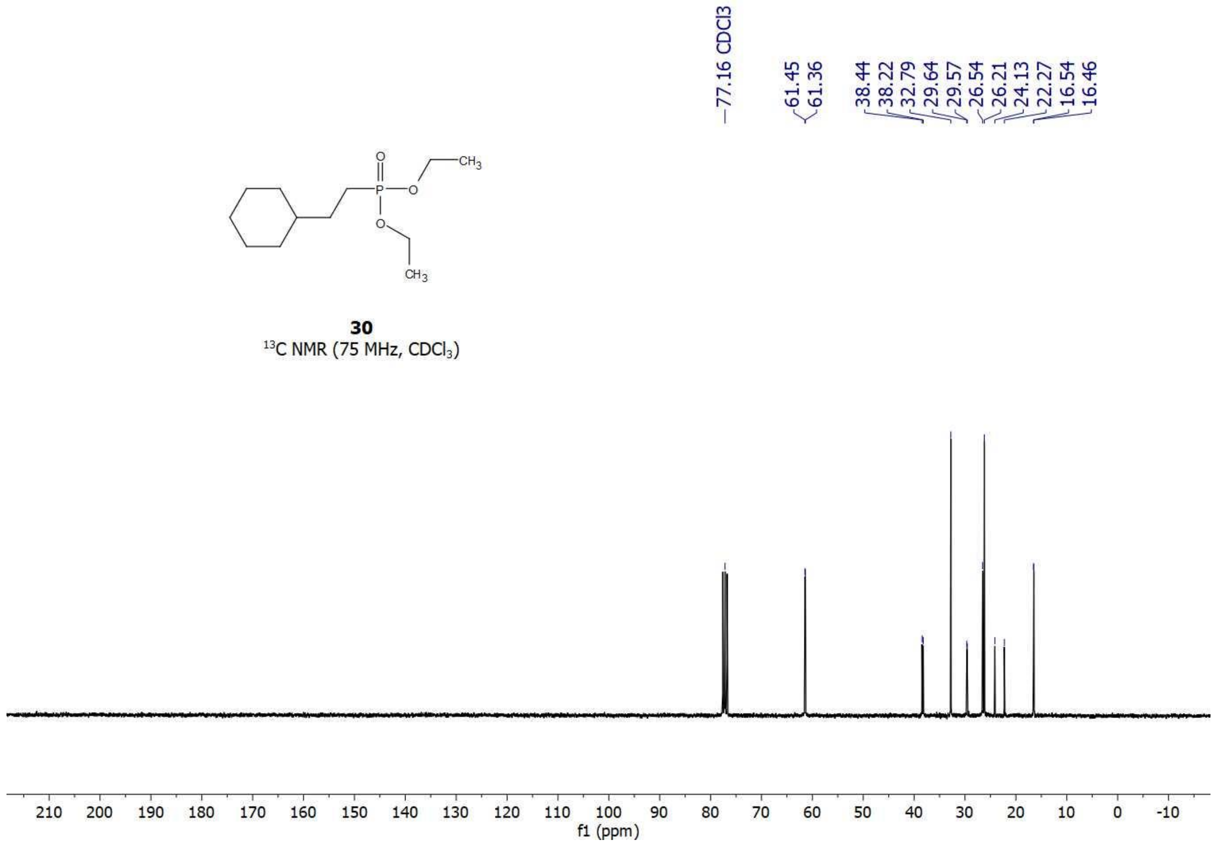

30
^{13}C NMR (75 MHz, $CDCl_3$)

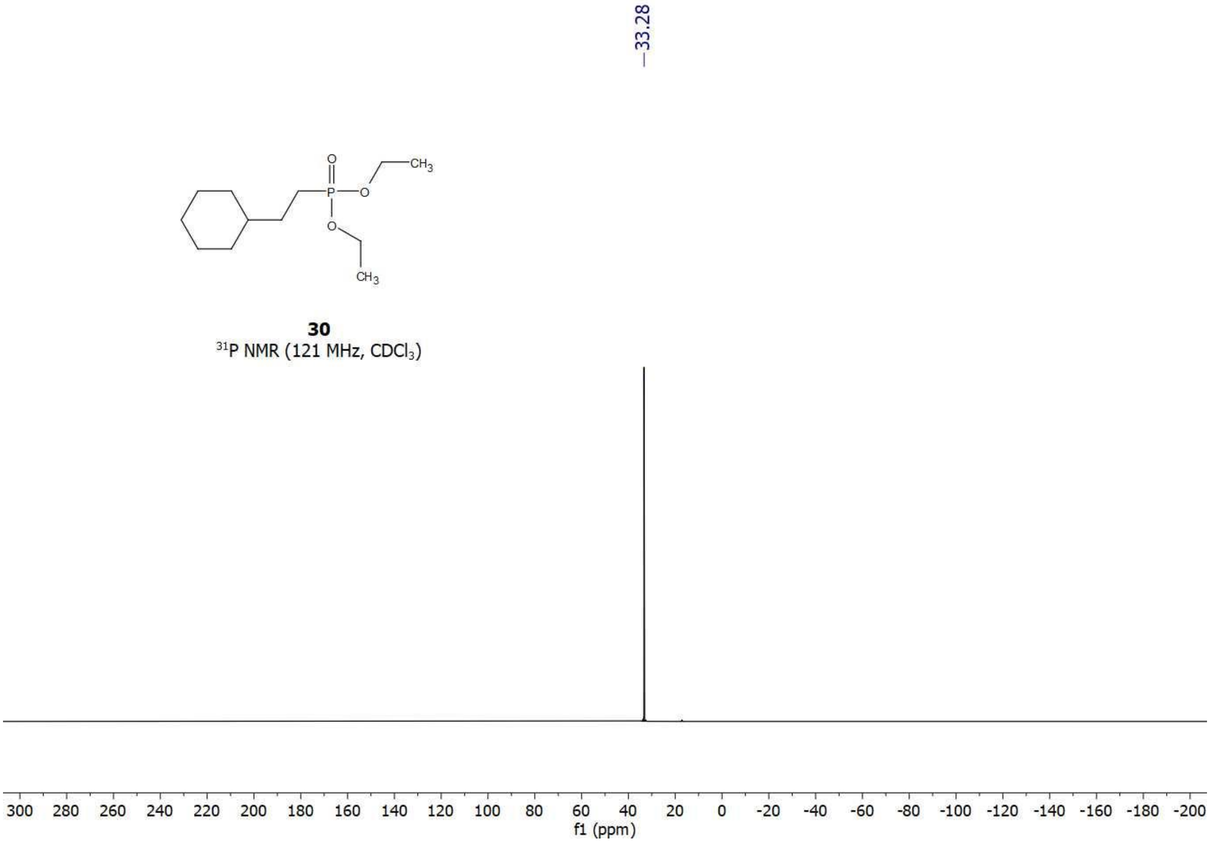

30
^{31}P NMR (121 MHz, $CDCl_3$)

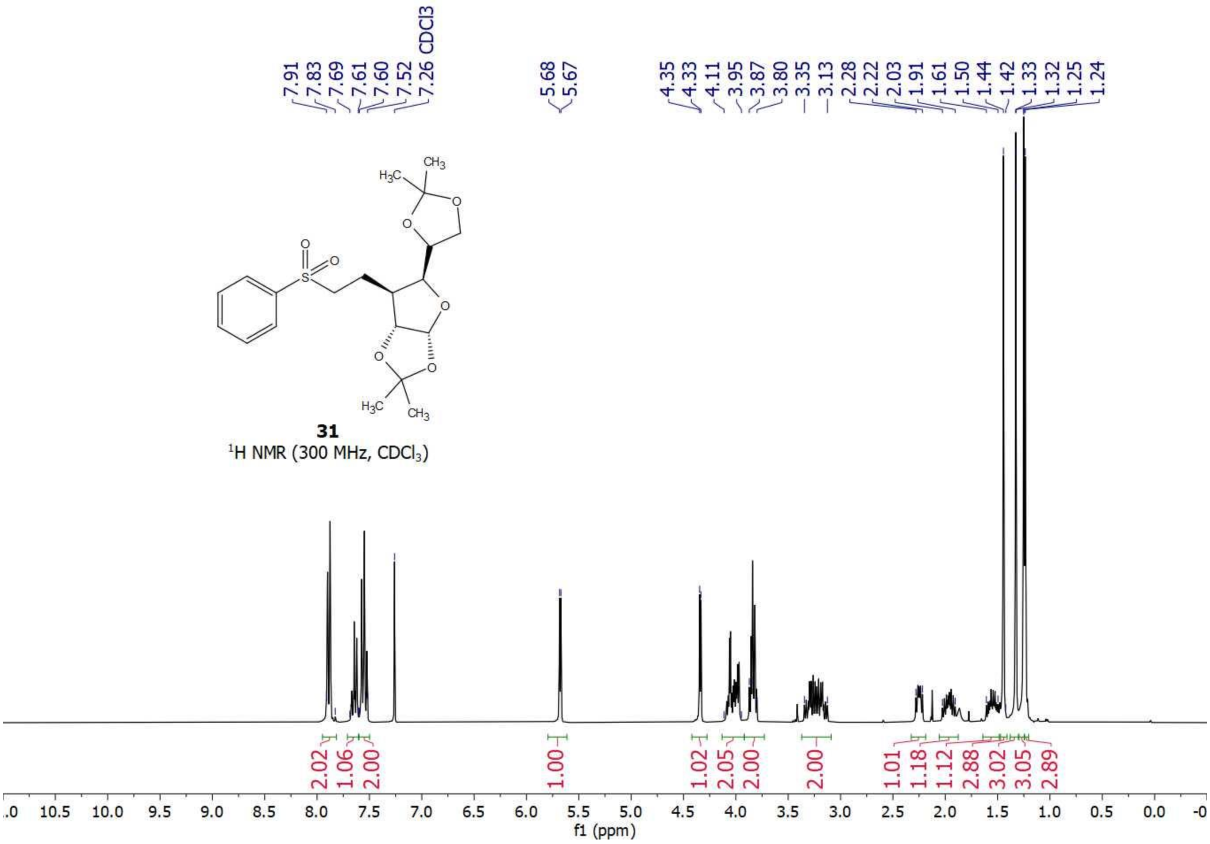

31
^{1}H NMR (300 MHz, $CDCl_3$)

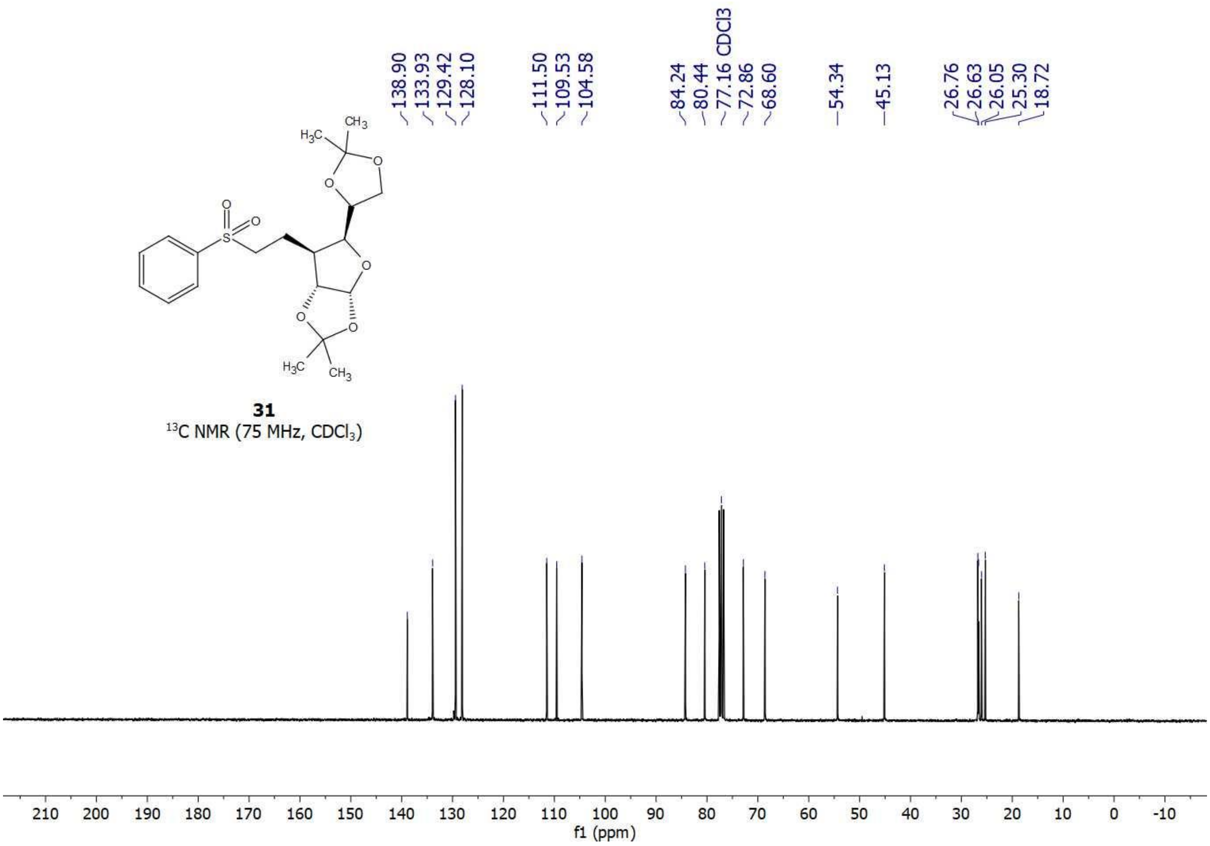

31
^{13}C NMR (75 MHz, $CDCl_3$)

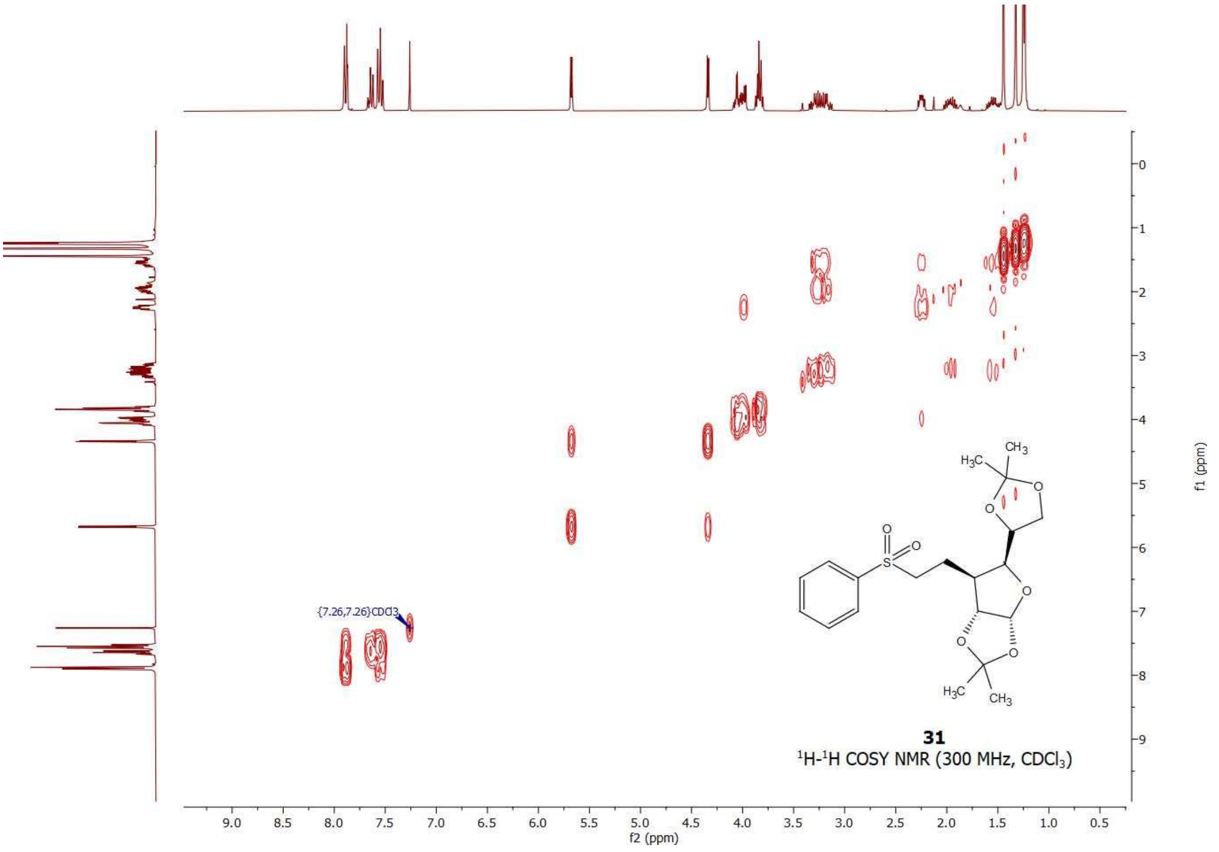

31
^{1}H-^{1}H COSY NMR (300 MHz, $CDCl_3$)

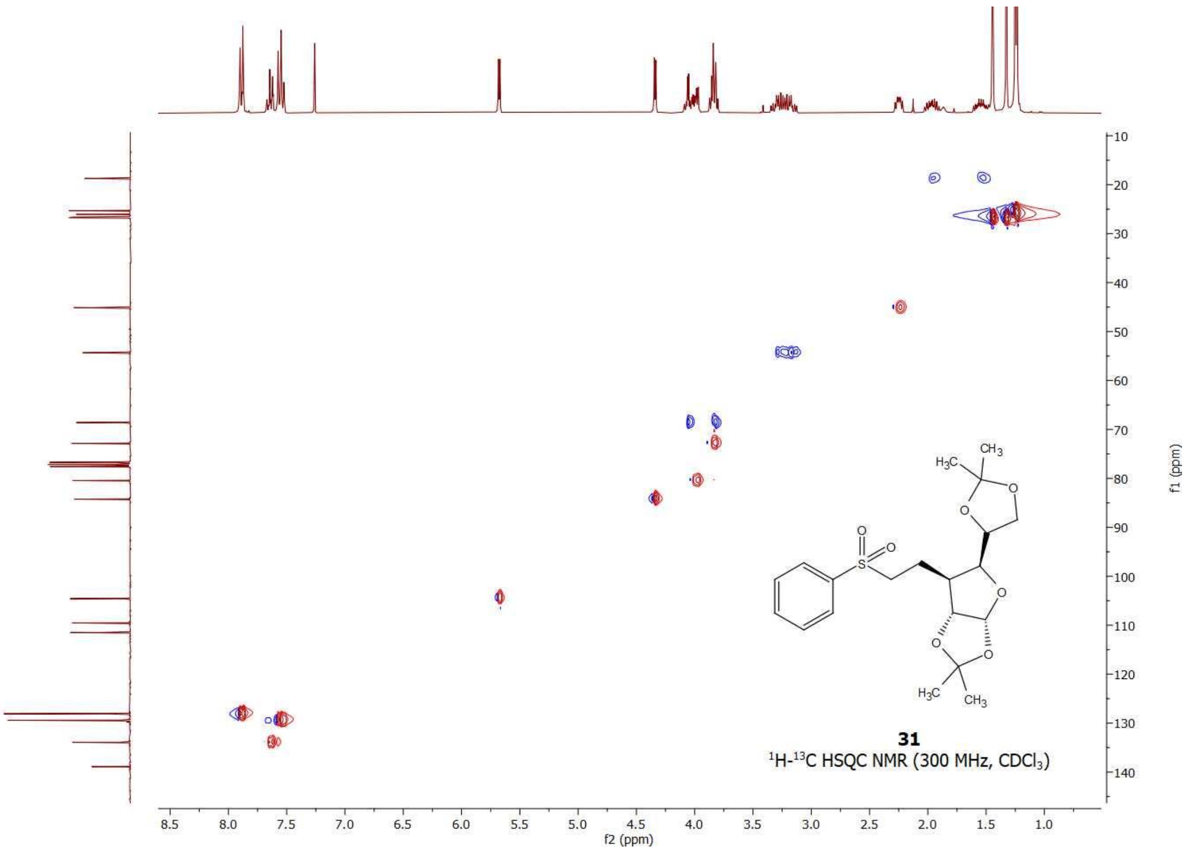
31
^{1}H-^{13}C HSQC NMR (300 MHz, CDCl$_3$)
f2 (ppm)
f1 (ppm)

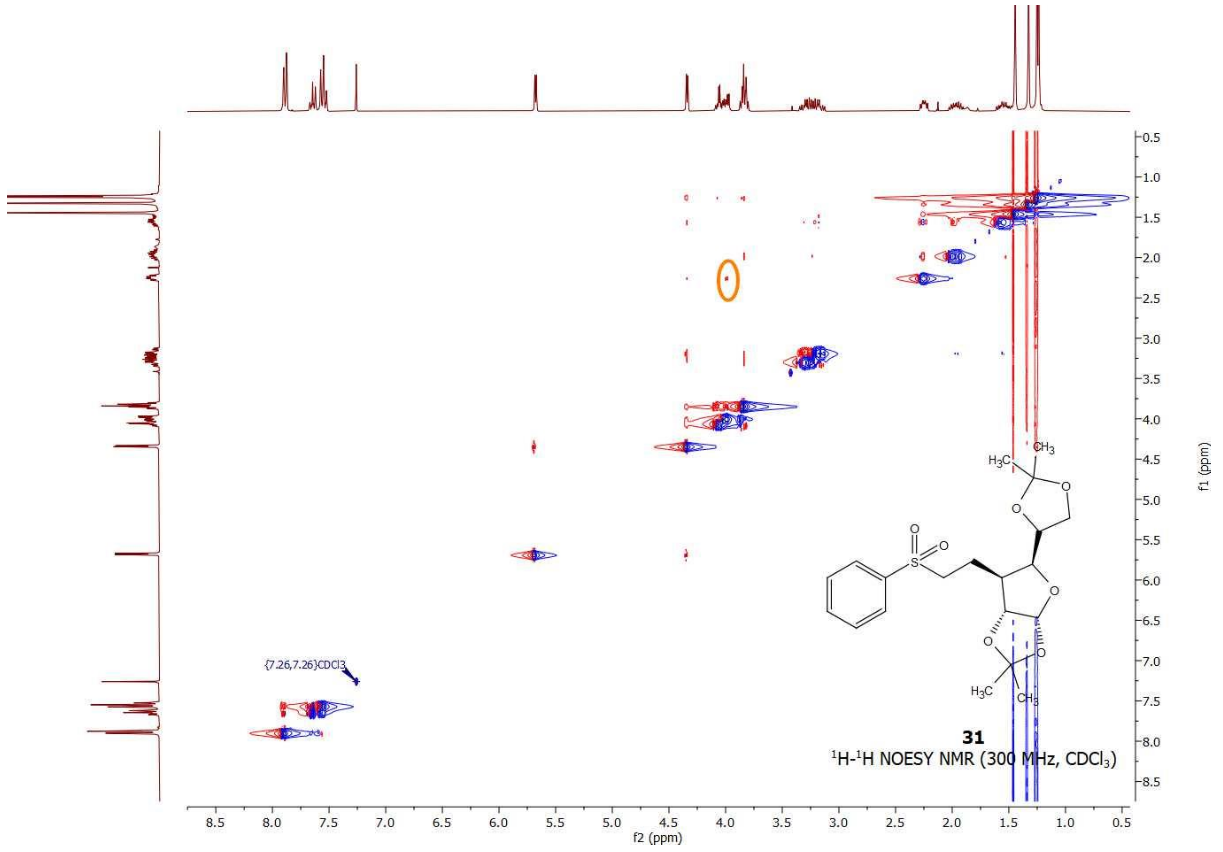
31
^{1}H-^{1}H NOESY NMR (300 MHz, CDCl$_3$)
{7.26,7.26}CDCl3
f2 (ppm)
f1 (ppm)

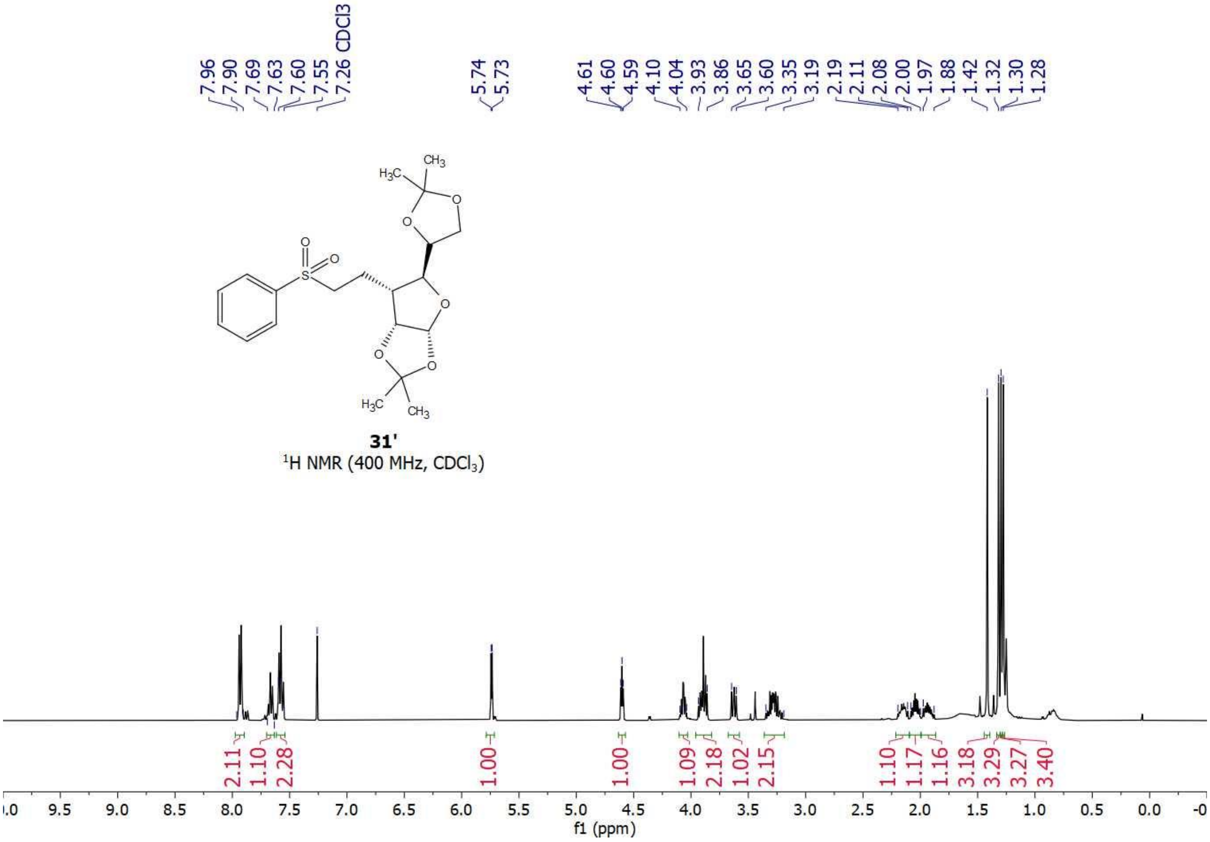

31'
^{1}H NMR (400 MHz, $CDCl_3$)

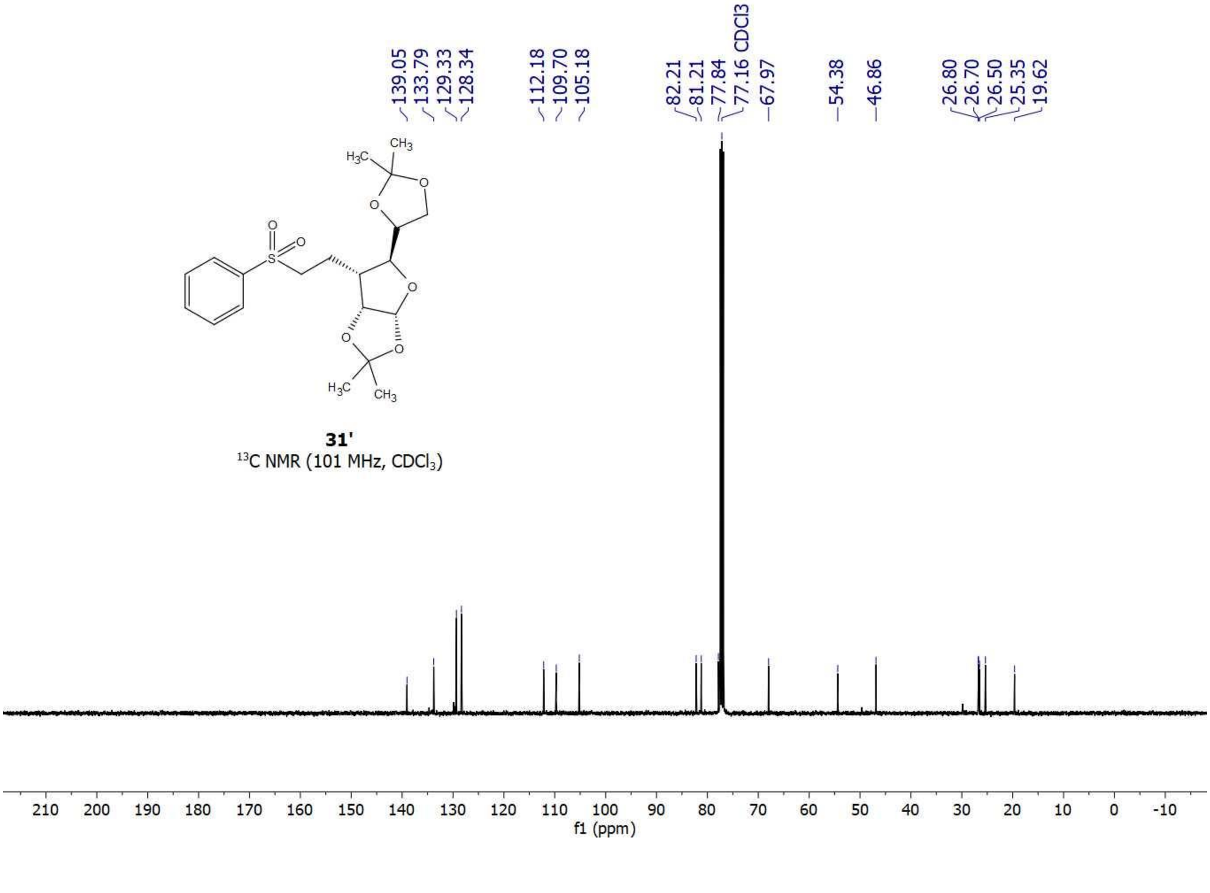

31'
^{13}C NMR (101 MHz, $CDCl_3$)

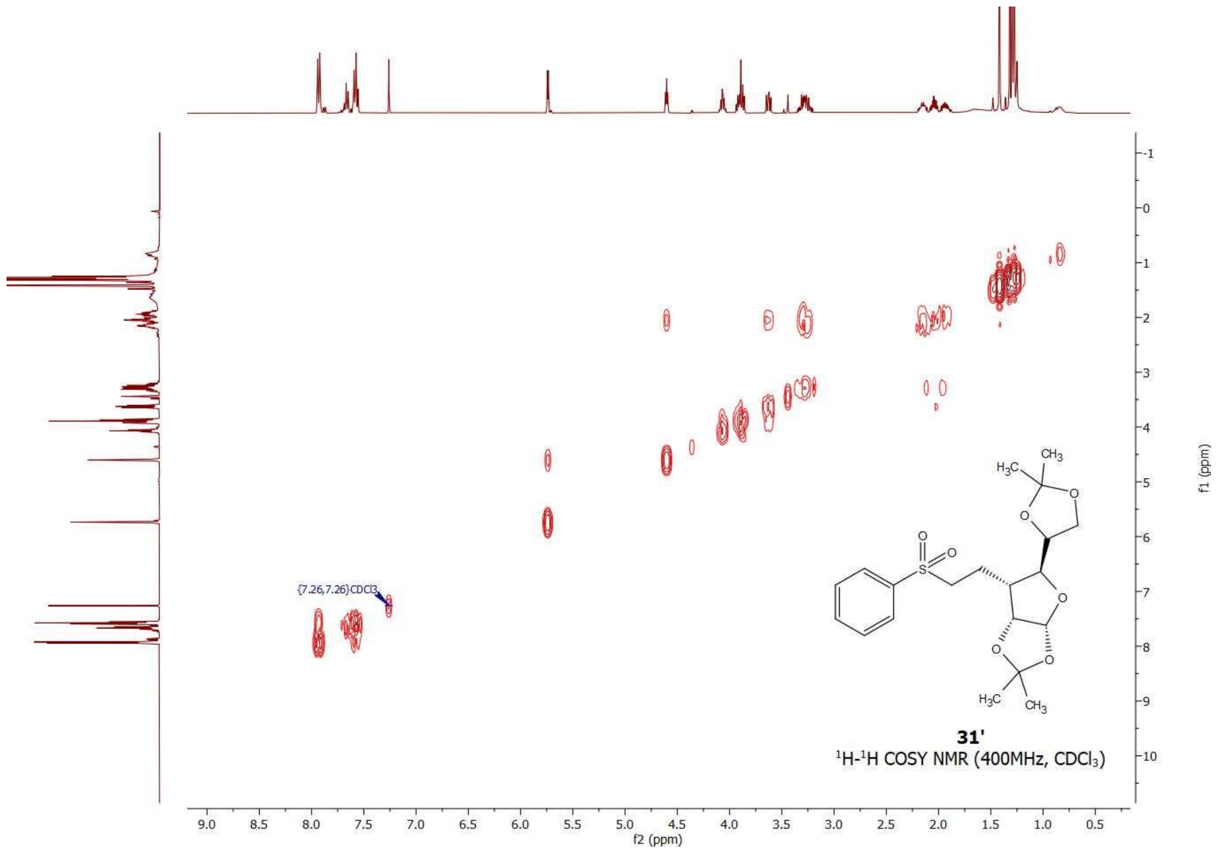
{7.26,7.26}CDCl3
31'
^{1}H-^{1}H COSY NMR (400MHz, $CDCl_3$)
f2 (ppm)
f1 (ppm)

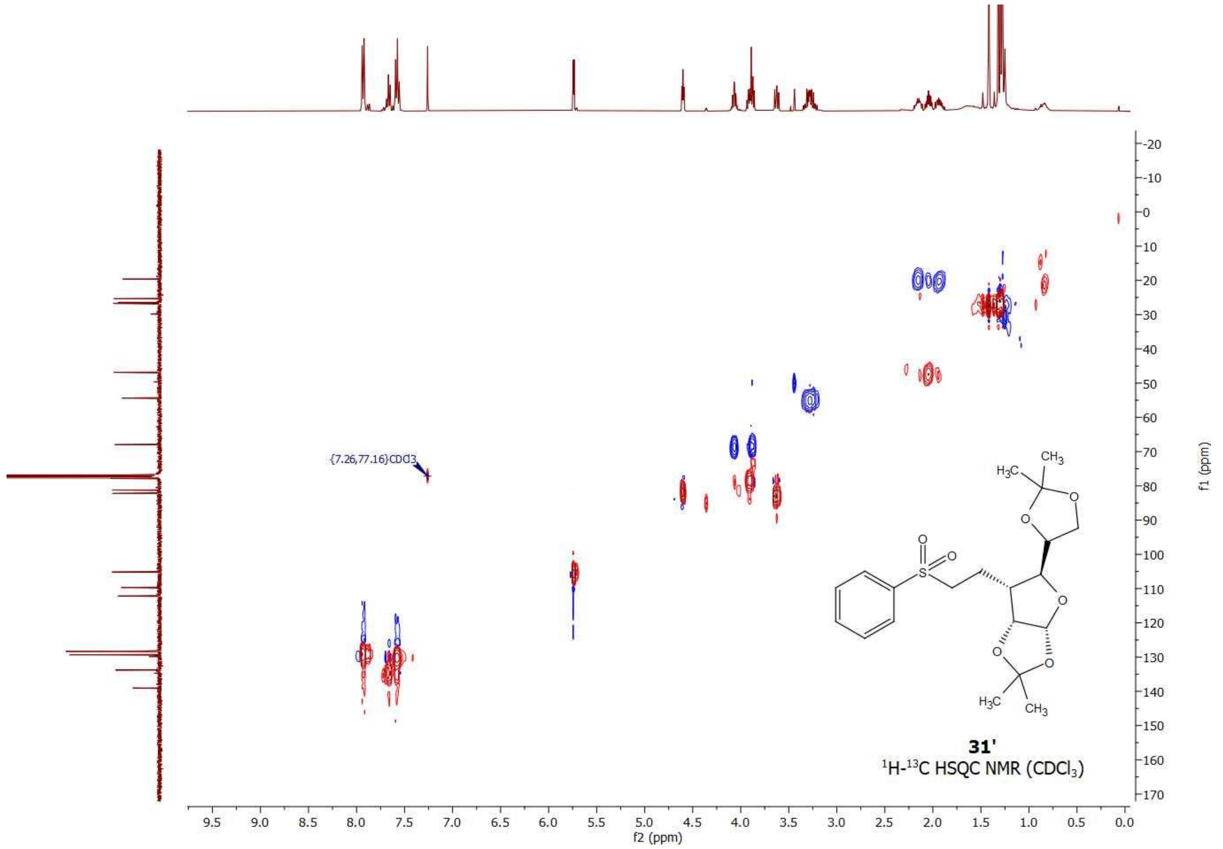
{7.26,77.16}CDCl3
31'
^{1}H-^{13}C HSQC NMR ($CDCl_3$)
f2 (ppm)
f1 (ppm)

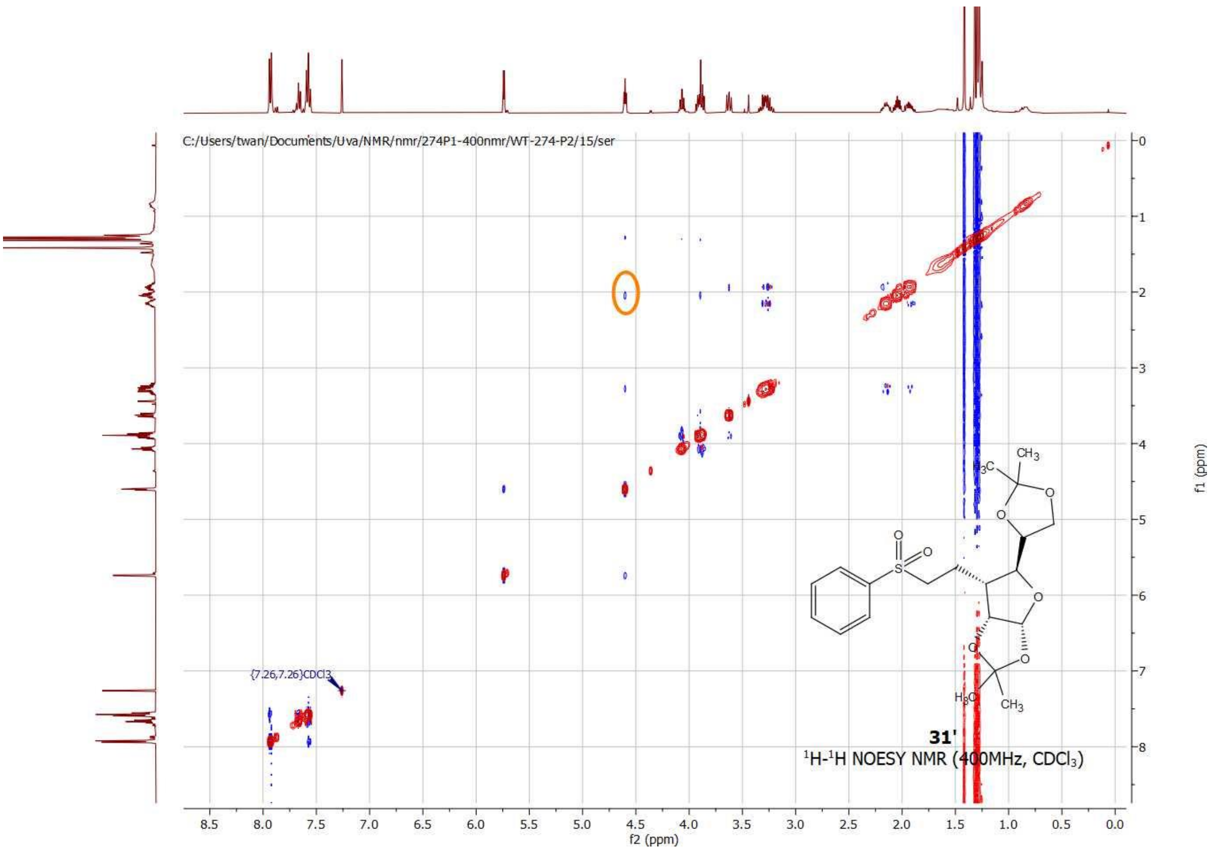

31'

^{1}H-^{1}H NOESY NMR (400MHz, $CDCl_3$)

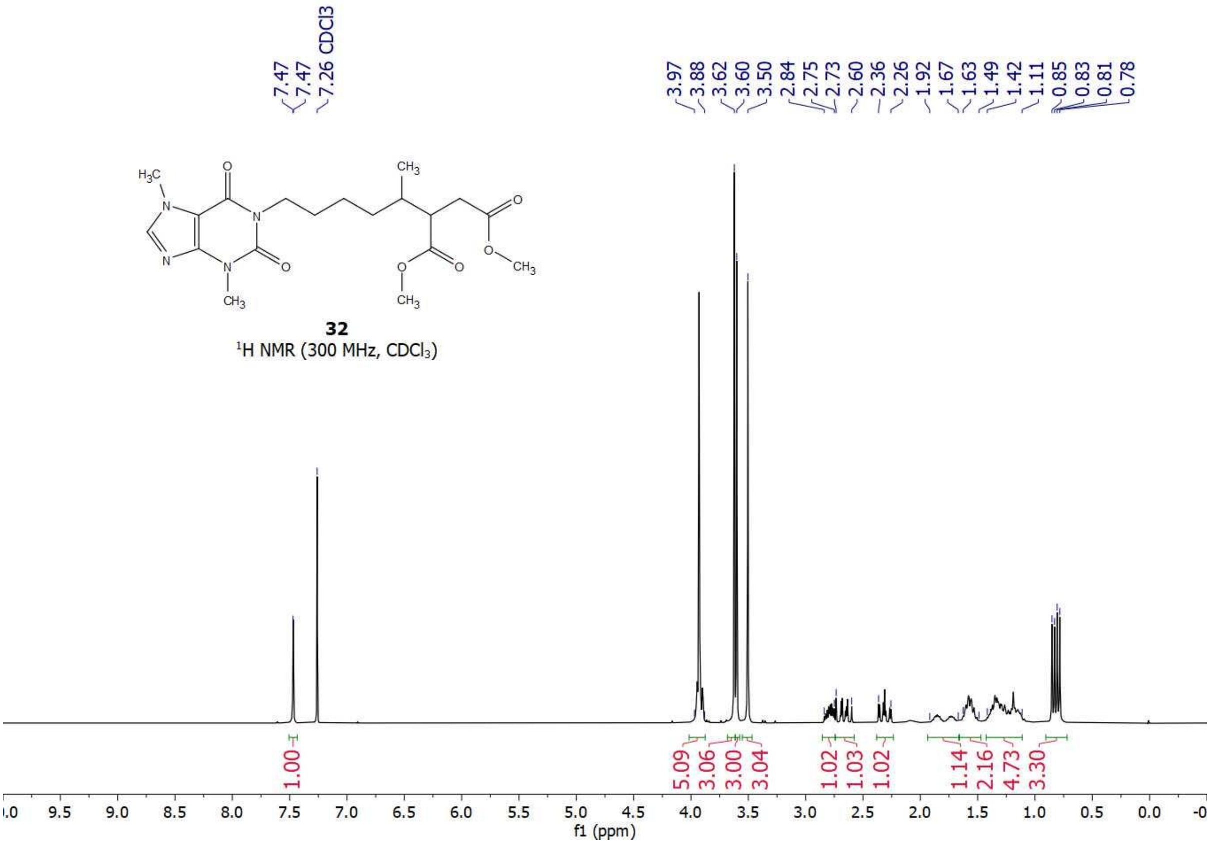

32

^{1}H NMR (300 MHz, $CDCl_3$)

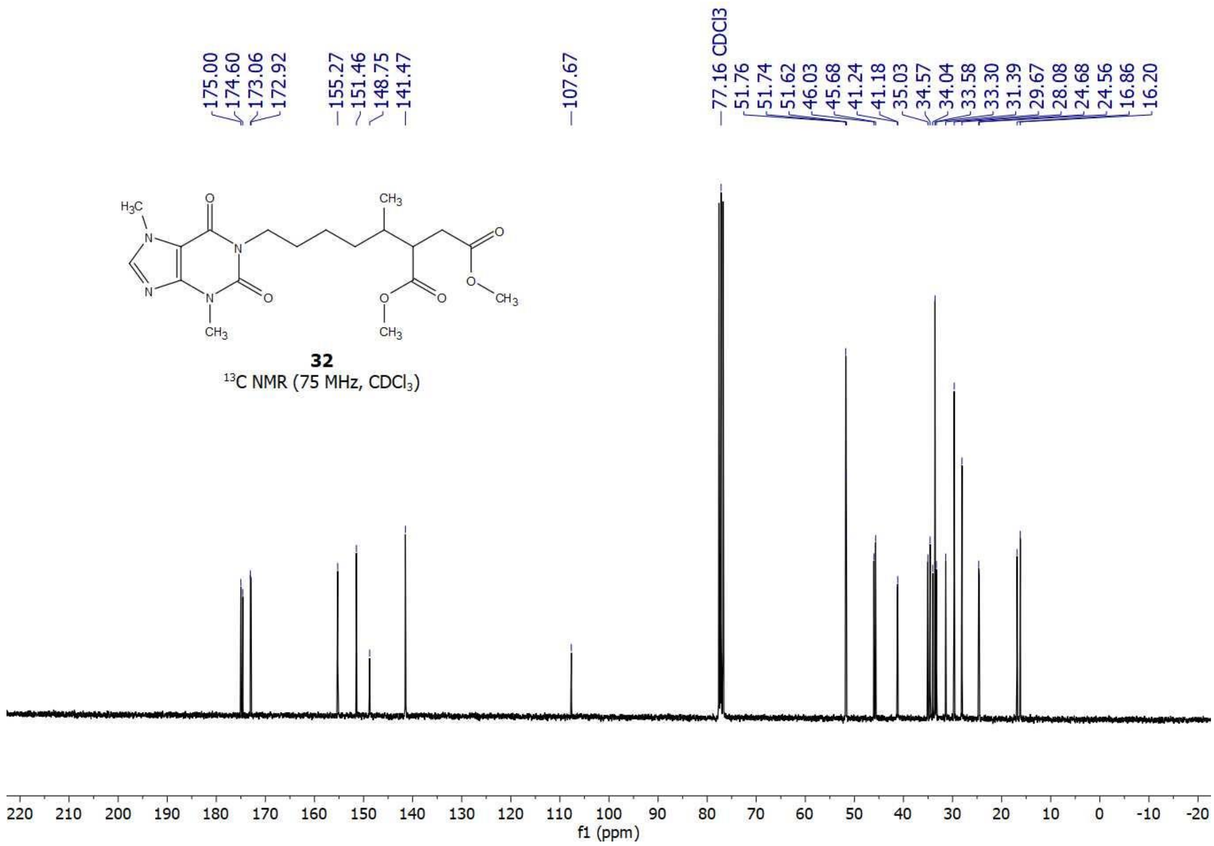

32
^{13}C NMR (75 MHz, CDCl$_3$)

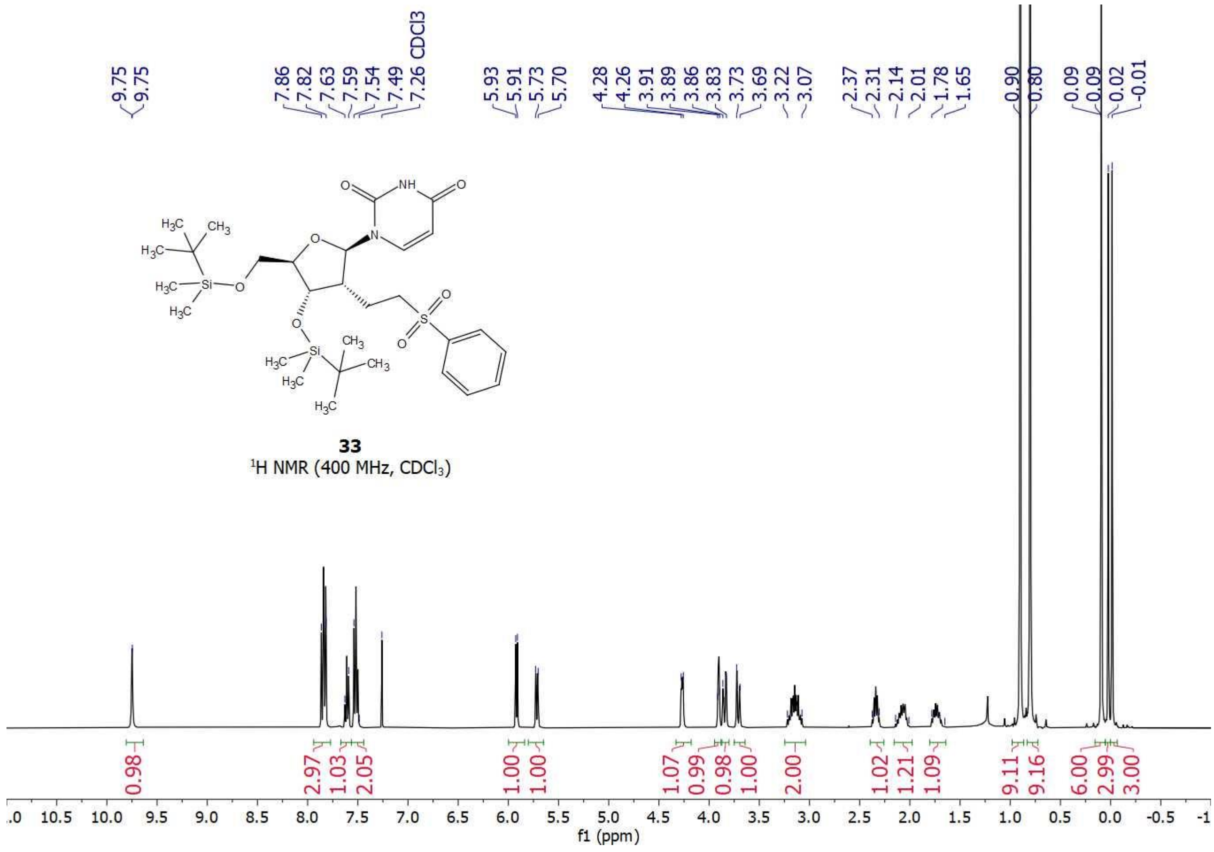

33
^{1}H NMR (400 MHz, CDCl$_3$)

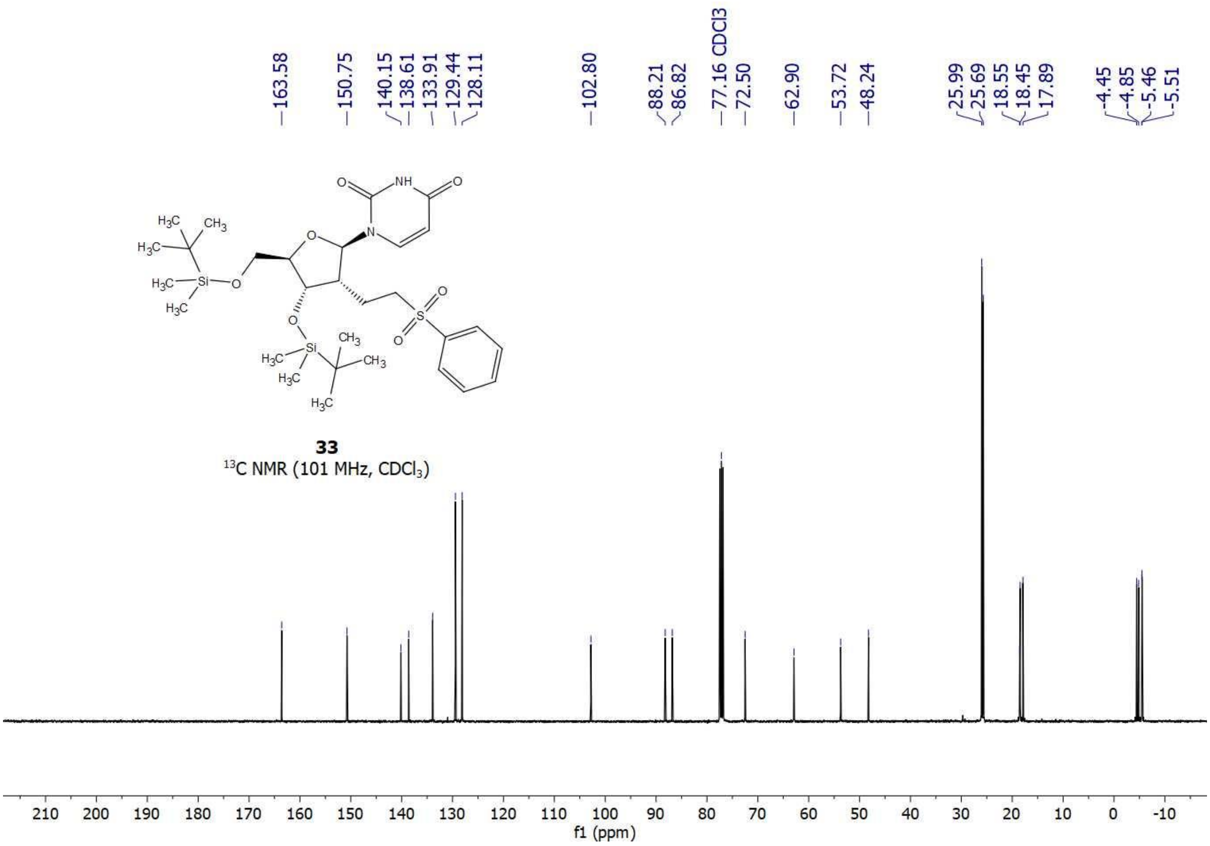
163.58
150.75
140.15
138.61
133.91
129.44
128.11
102.80
88.21
86.82
77.16 CDCl3
72.50
62.90
53.72
48.24
25.99
25.69
18.55
18.45
17.89
-4.45
-4.85
-5.46
-5.51
33
13C NMR (101 MHz, CDCl3)
f1 (ppm)

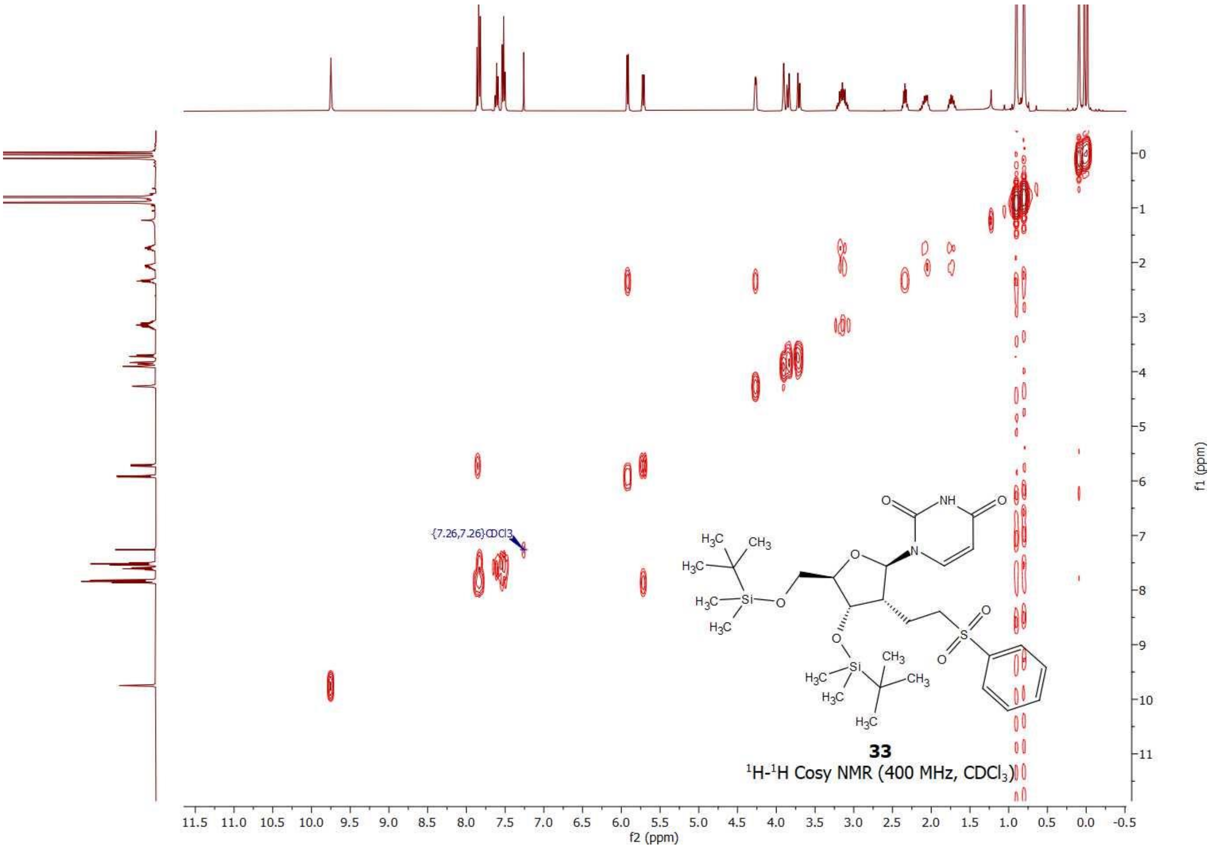
{7.26,7.26}CDCl3
33
1H-1H Cosy NMR (400 MHz, CDCl3)
f2 (ppm)
f1 (ppm)

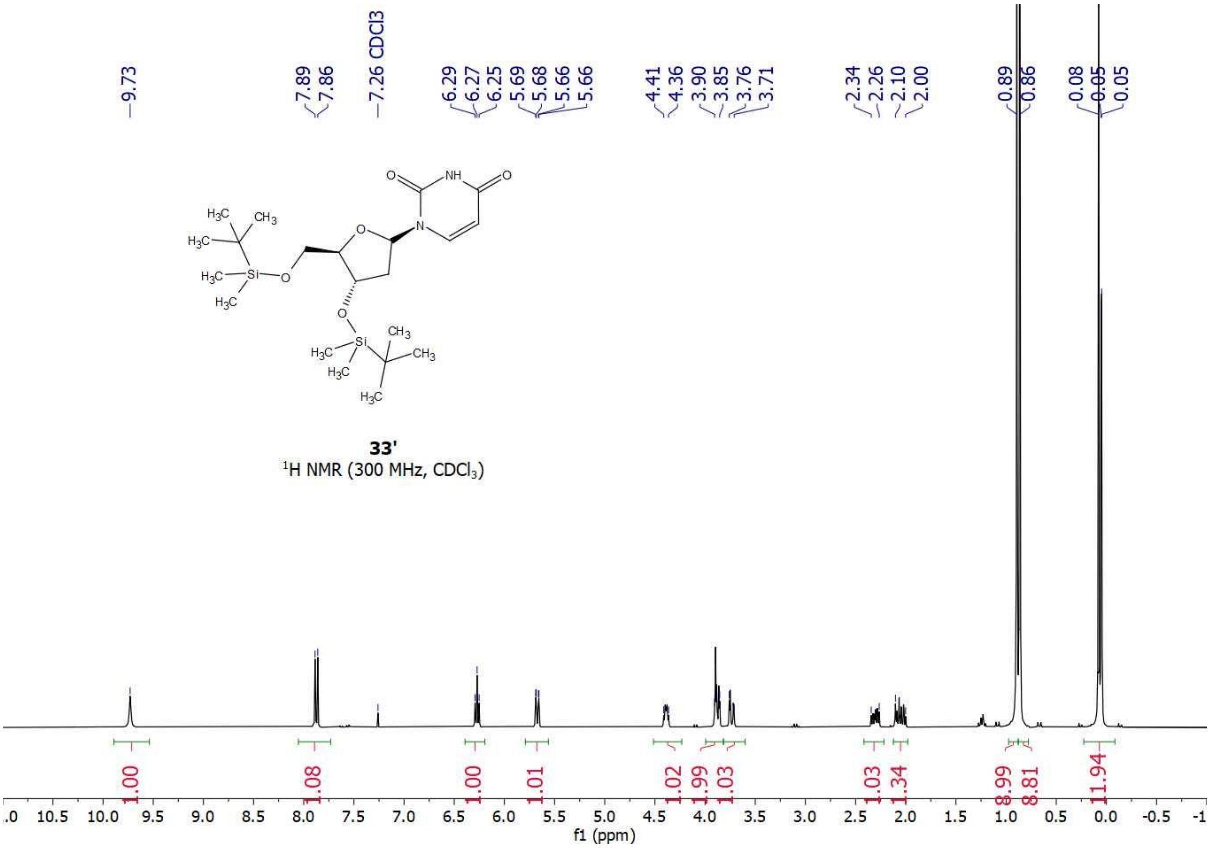

33'
^{1}H NMR (300 MHz, $CDCl_3$)

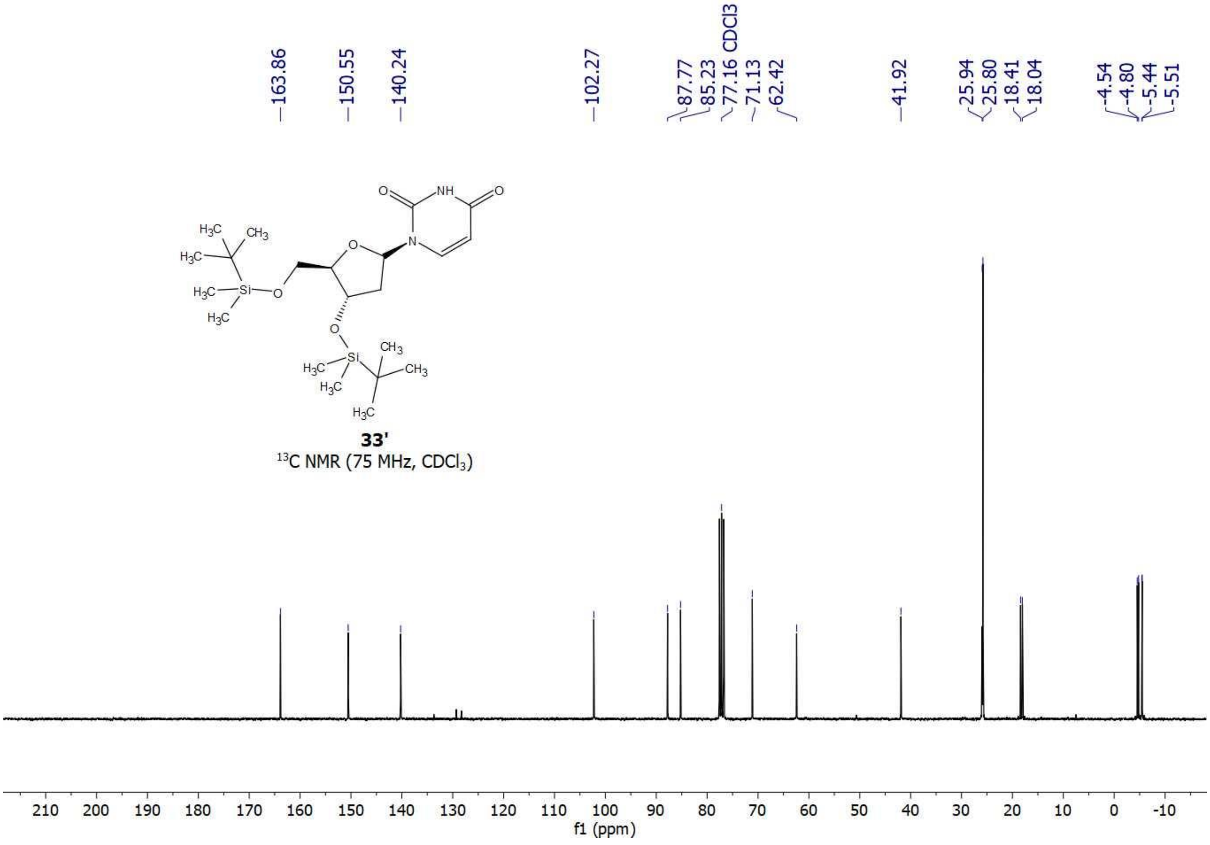

33'
^{13}C NMR (75 MHz, $CDCl_3$)

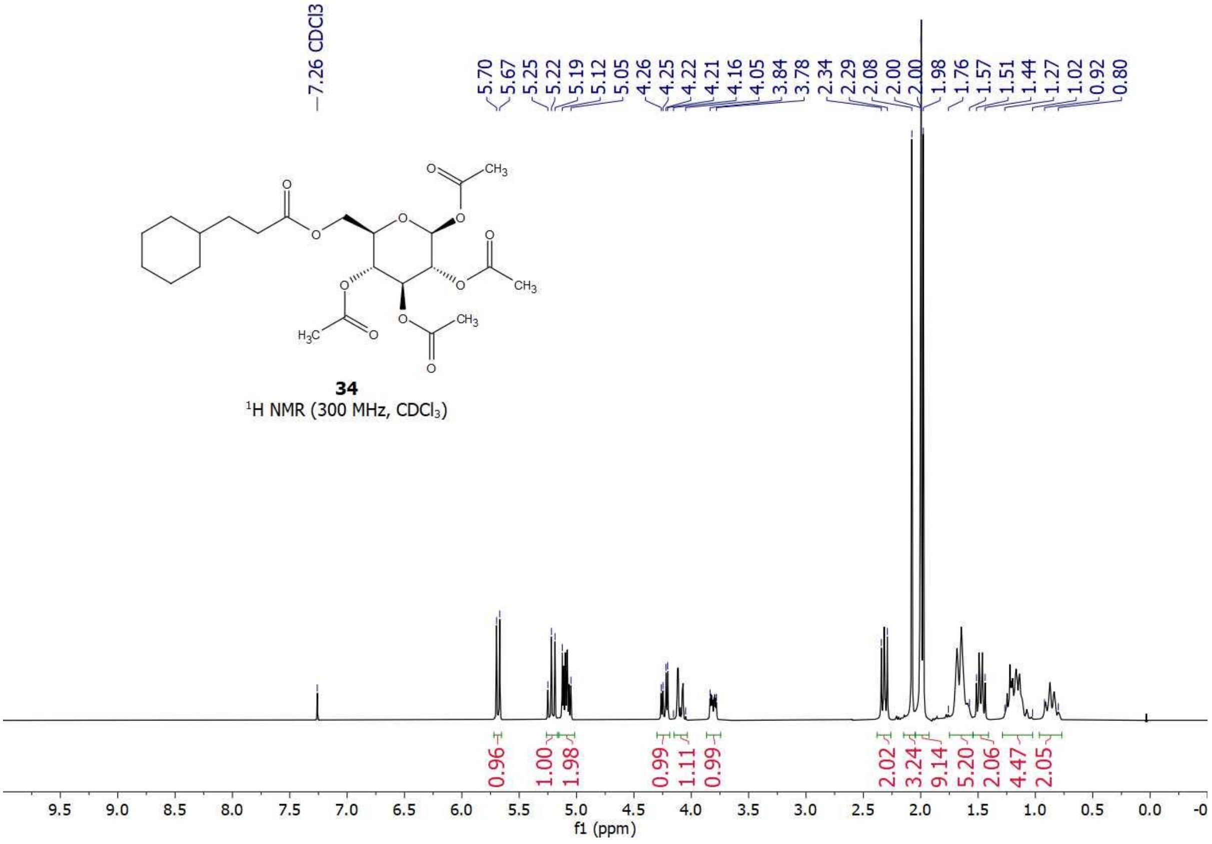

34

^{1}H NMR (300 MHz, $CDCl_3$)

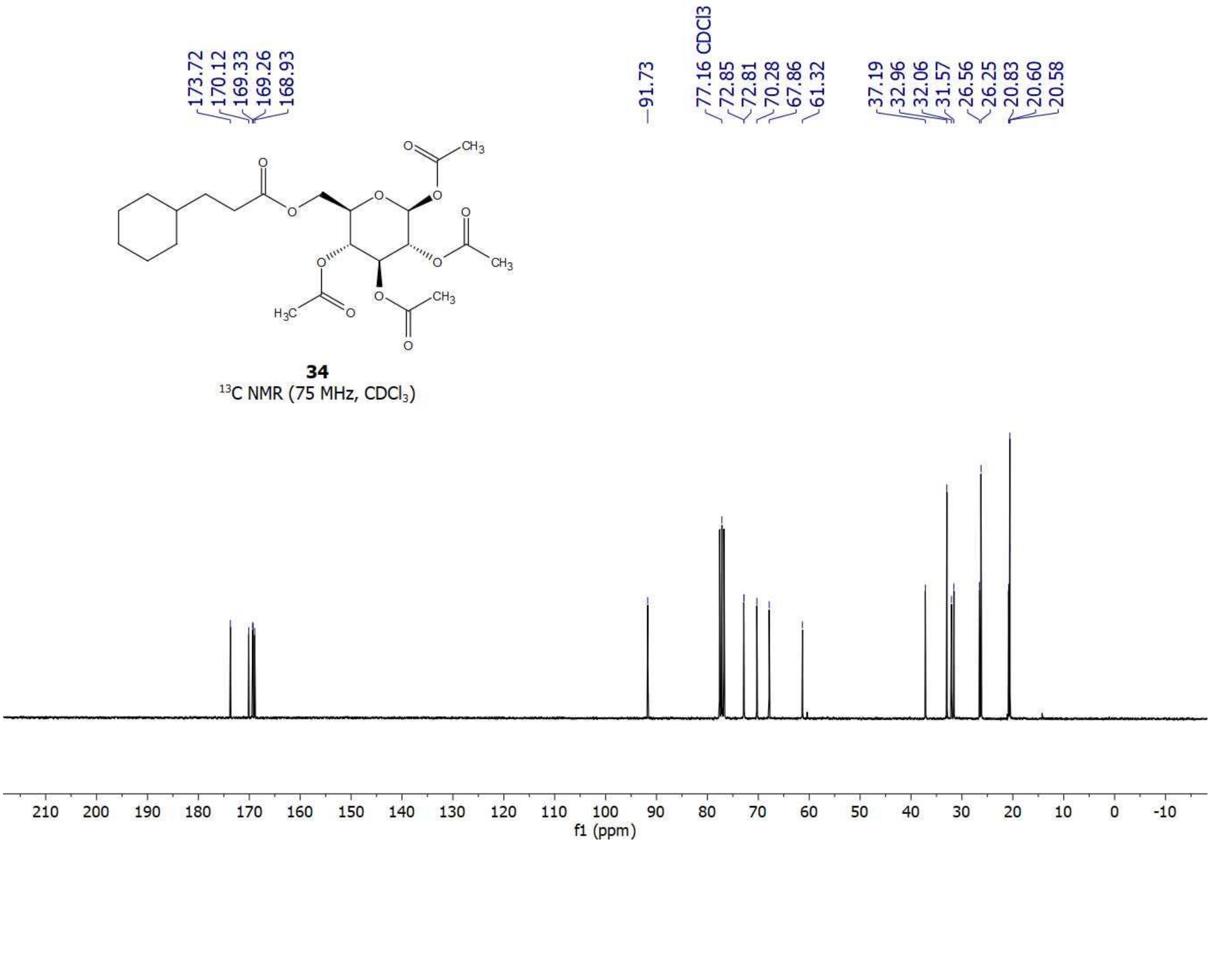

34

^{13}C NMR (75 MHz, $CDCl_3$)

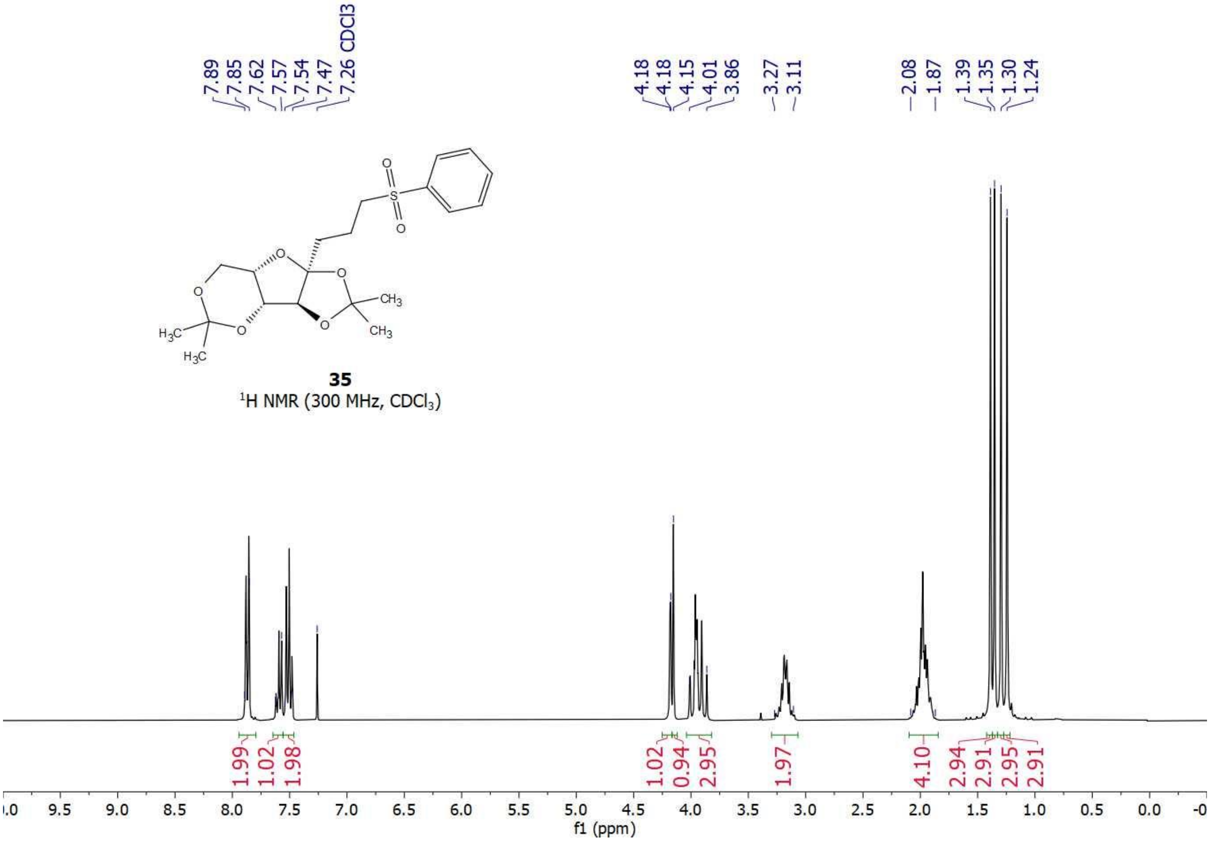

35
^1H NMR (300 MHz, $CDCl_3$)

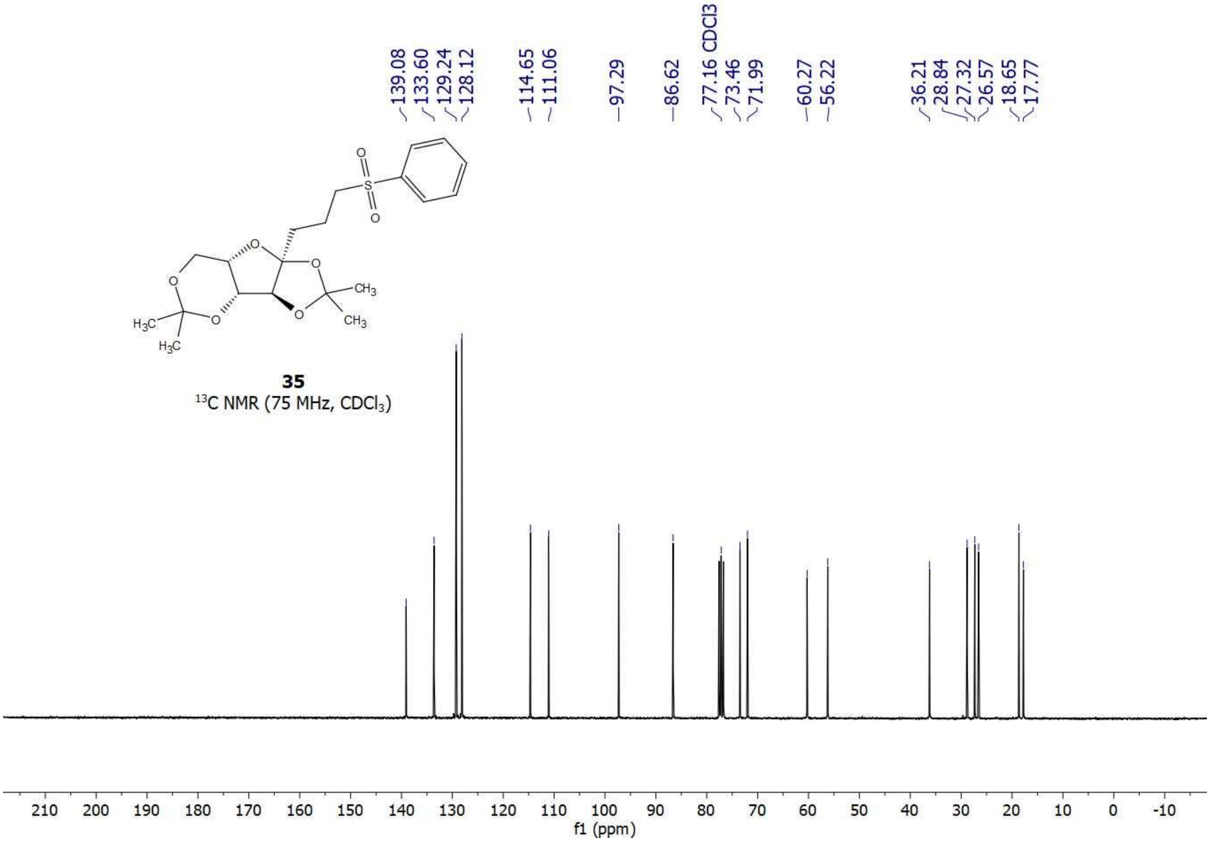

35
^{13}C NMR (75 MHz, $CDCl_3$)

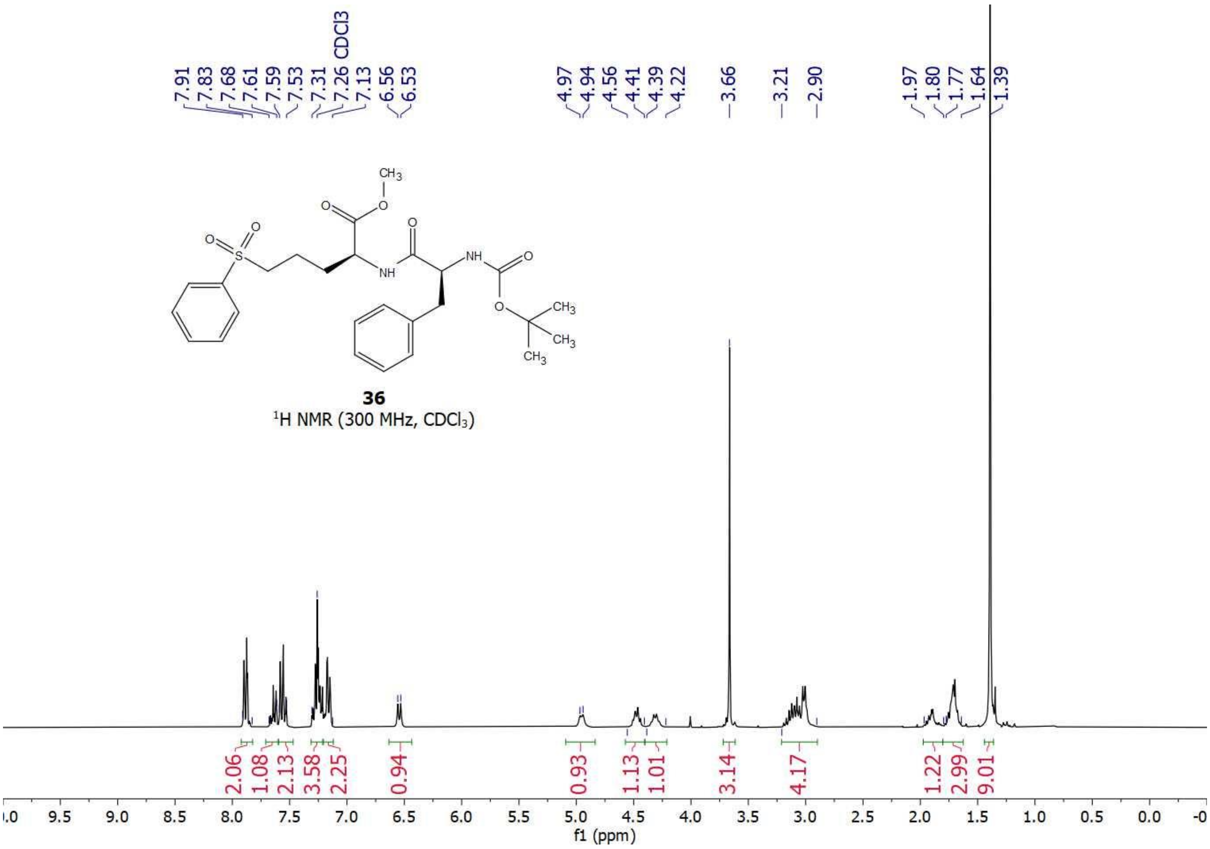

7.91
7.83
7.68
7.61
7.59
7.53
7.31
7.26 CDCl3
7.13
6.56
6.53
4.97
4.94
4.56
4.41
4.39
4.22
3.66
3.21
2.90
1.97
1.80
1.77
1.64
1.39
CH3
O
O
O
S
O
NH
O
NH
O
O
CH3
CH3
CH3
36
1H NMR (300 MHz, CDCl3)
2.06
1.08
2.13
3.58
2.25
0.94
0.93
1.13
1.01
3.14
4.17
1.22
2.99
9.01
f1 (ppm)

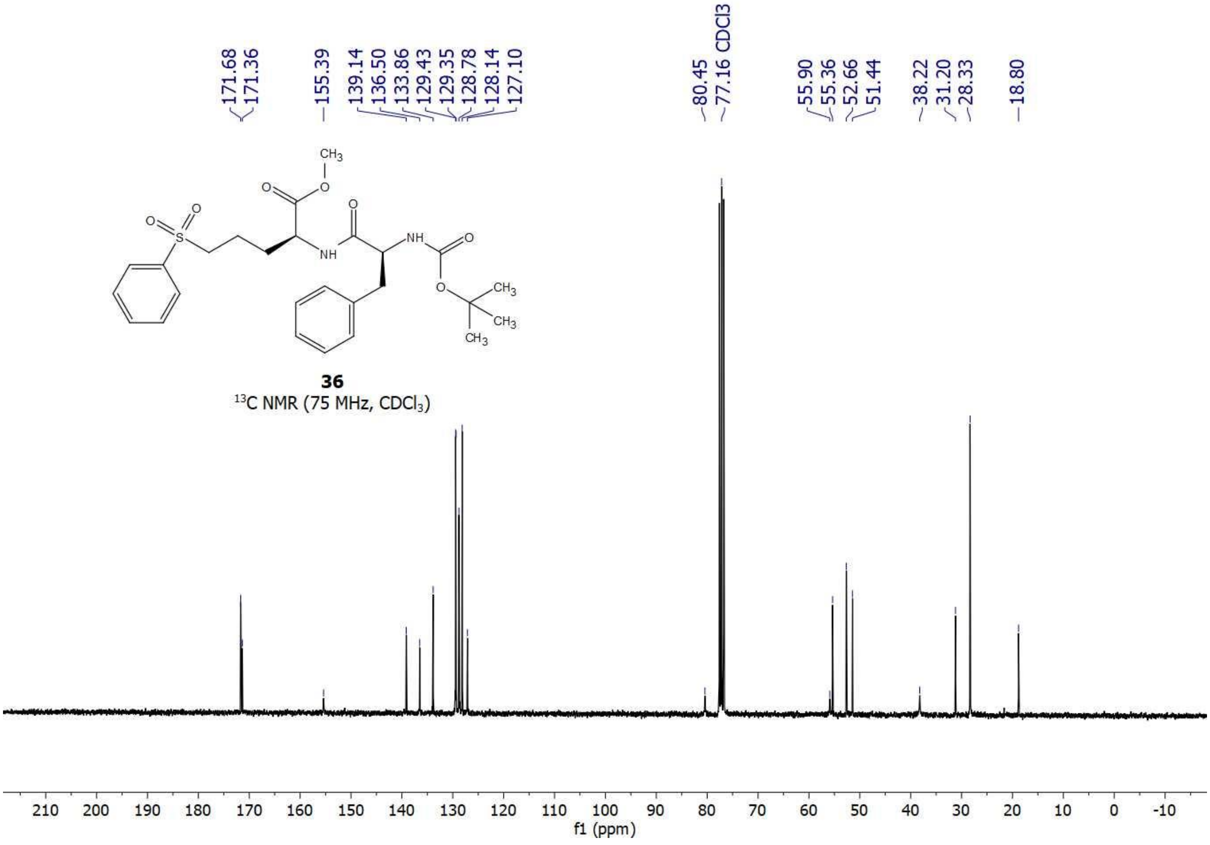

171.68
171.36
155.39
139.14
136.50
133.86
129.43
129.35
128.78
128.14
127.10
80.45
77.16 CDCl3
55.90
55.36
52.66
51.44
38.22
31.20
28.33
18.80
36
13C NMR (75 MHz, CDCl3)
f1 (ppm)

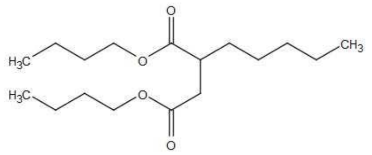

37

^{1}H NMR (300 MHz, $CDCl_3$)

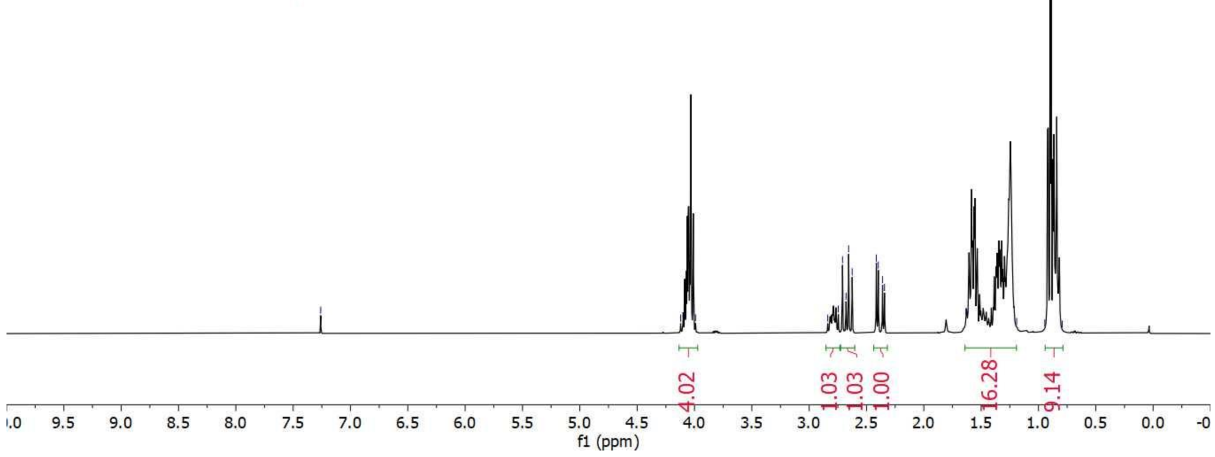

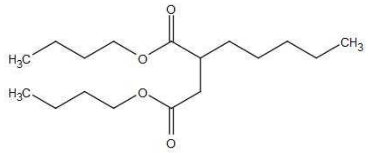

37

^{13}C NMR (75 MHz, $CDCl_3$)

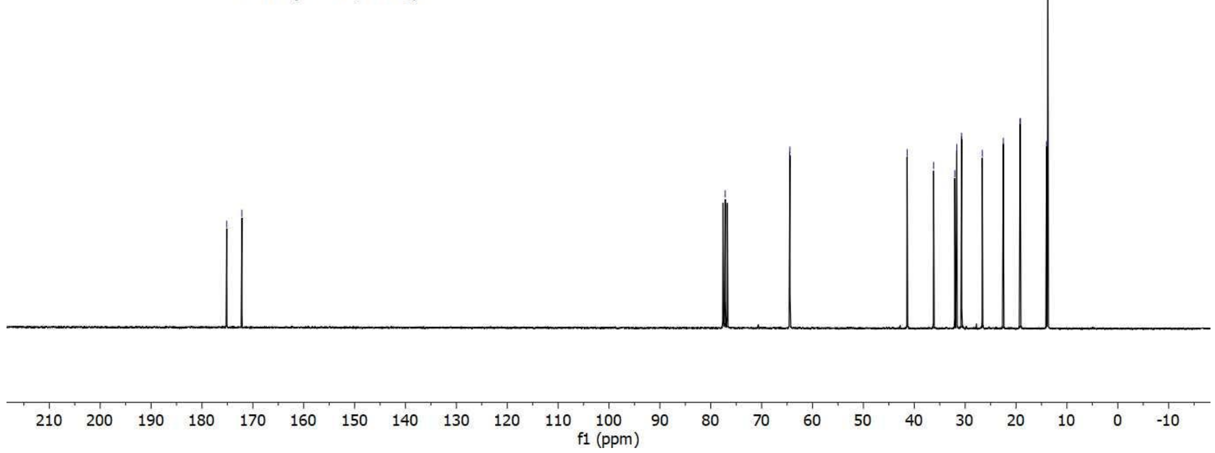

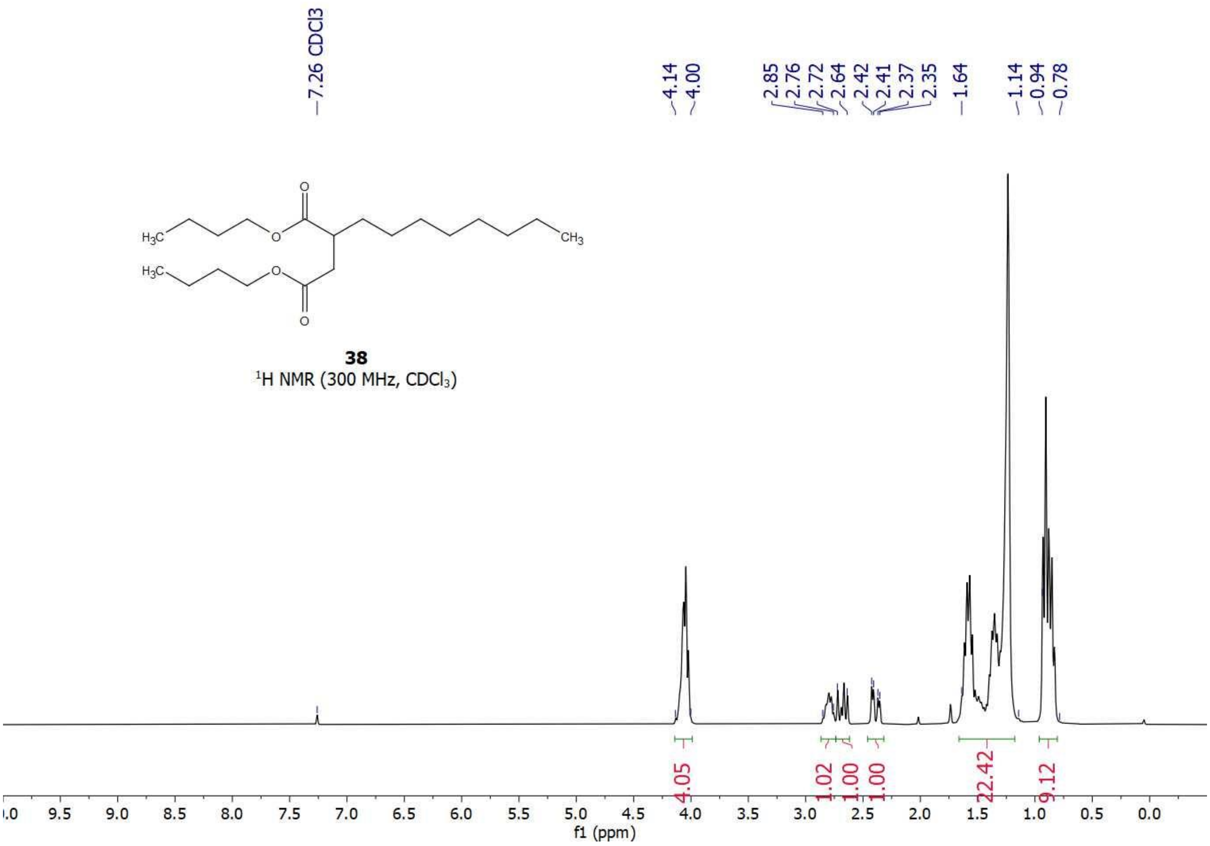

38
^{1}H NMR (300 MHz, $CDCl_3$)

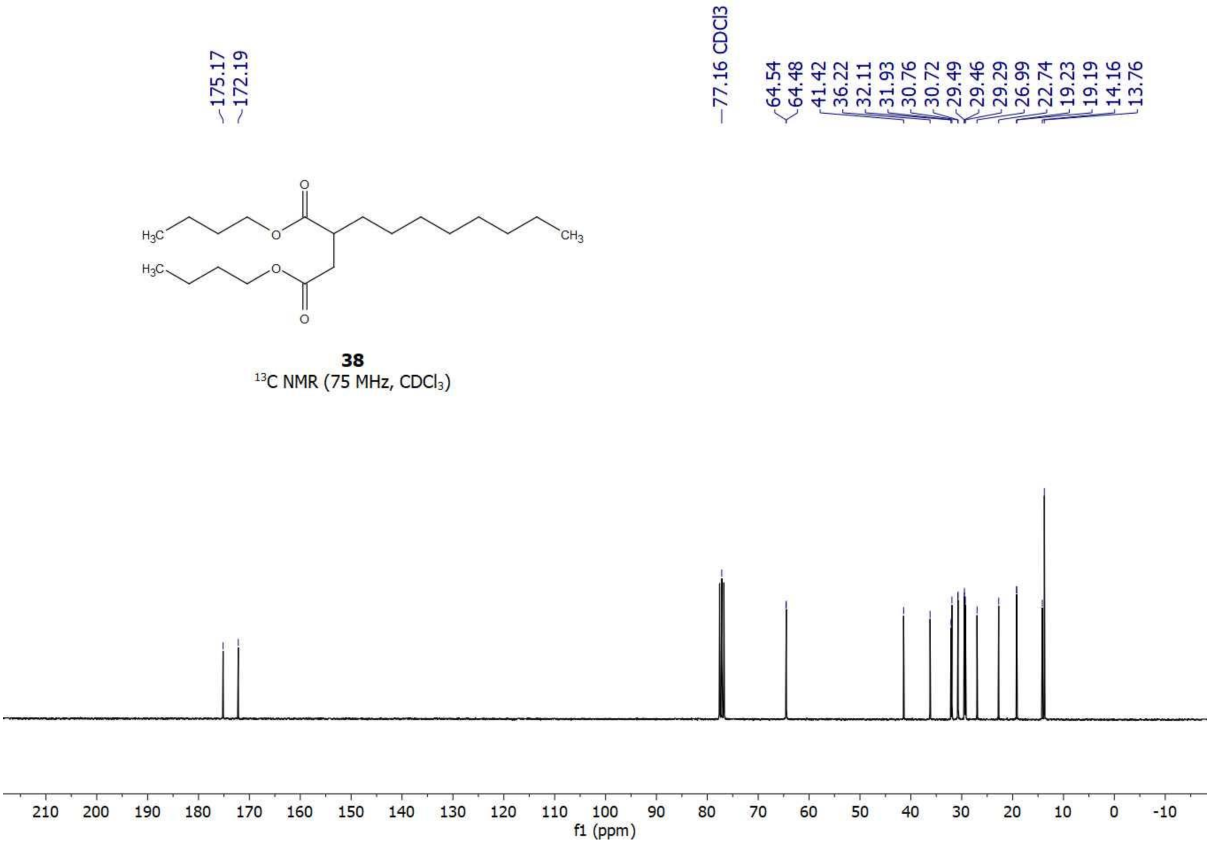

38
^{13}C NMR (75 MHz, $CDCl_3$)

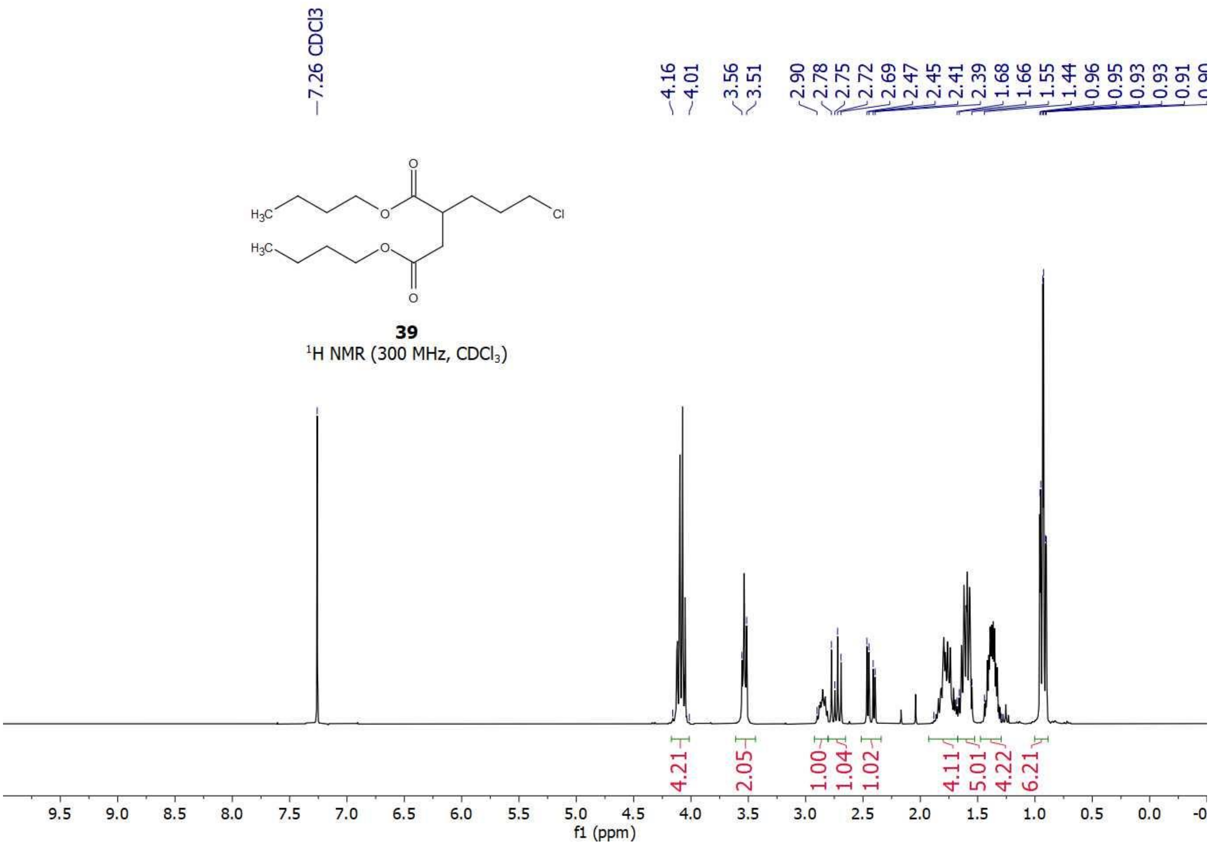
7.26 CDCl3
4.16
4.01
3.56
3.51
2.90
2.78
2.75
2.72
2.69
2.47
2.45
2.41
2.39
1.68
1.66
1.55
1.44
0.96
0.95
0.93
0.93
0.91
39
^{1}H NMR (300 MHz, $CDCl_3$)
4.21
2.05
1.00
1.04
1.02
4.11
5.01
4.22
6.21
f1 (ppm)

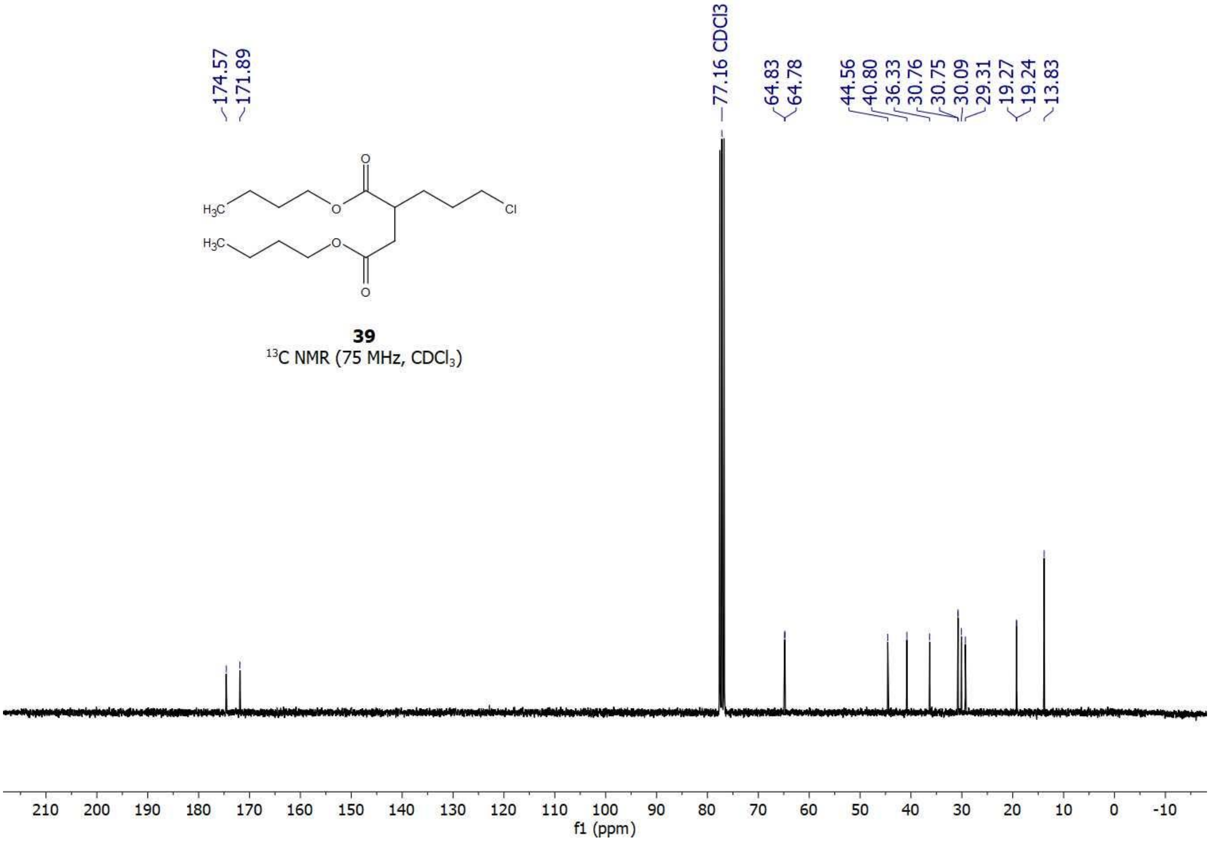
174.57
171.89
77.16 CDCl3
64.83
64.78
44.56
40.80
36.33
30.76
30.75
30.09
29.31
19.27
19.24
13.83
39
^{13}C NMR (75 MHz, $CDCl_3$)
f1 (ppm)

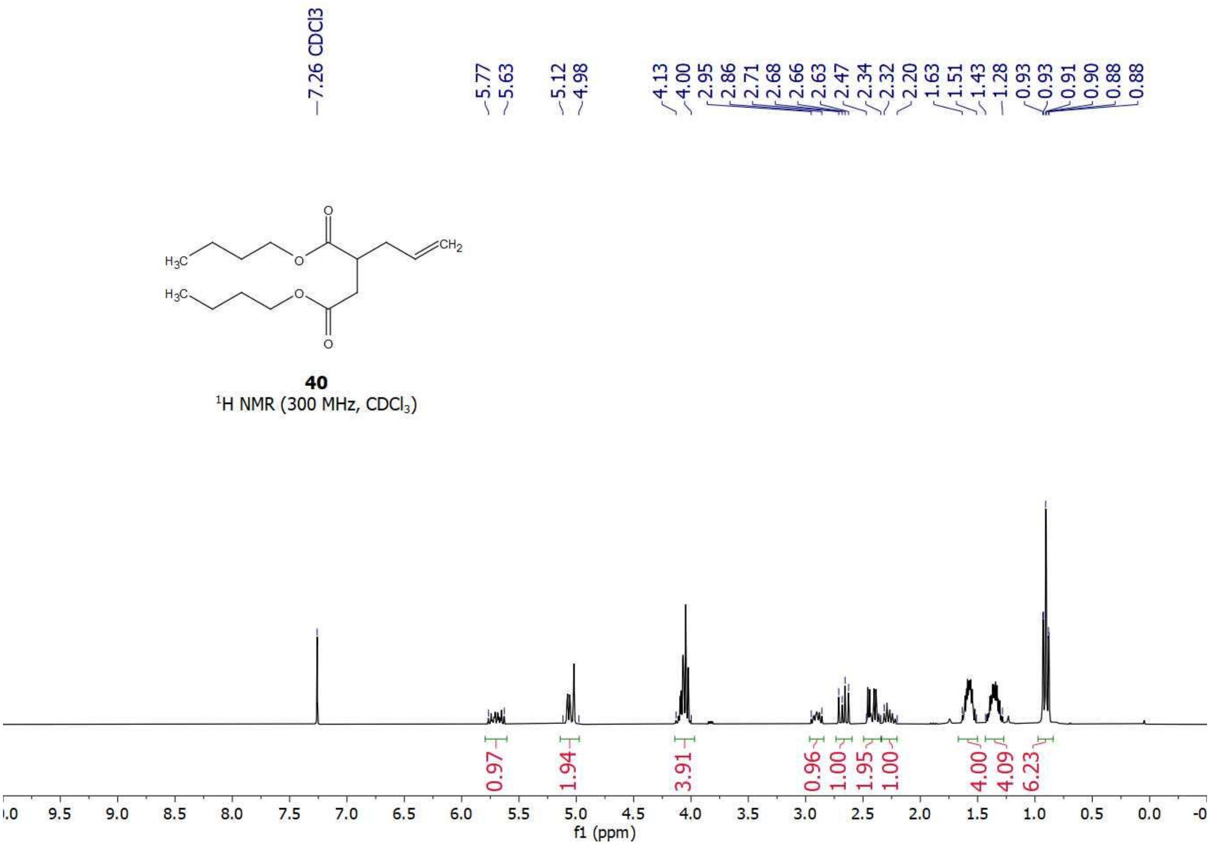

40

^{1}H NMR (300 MHz, $CDCl_3$)

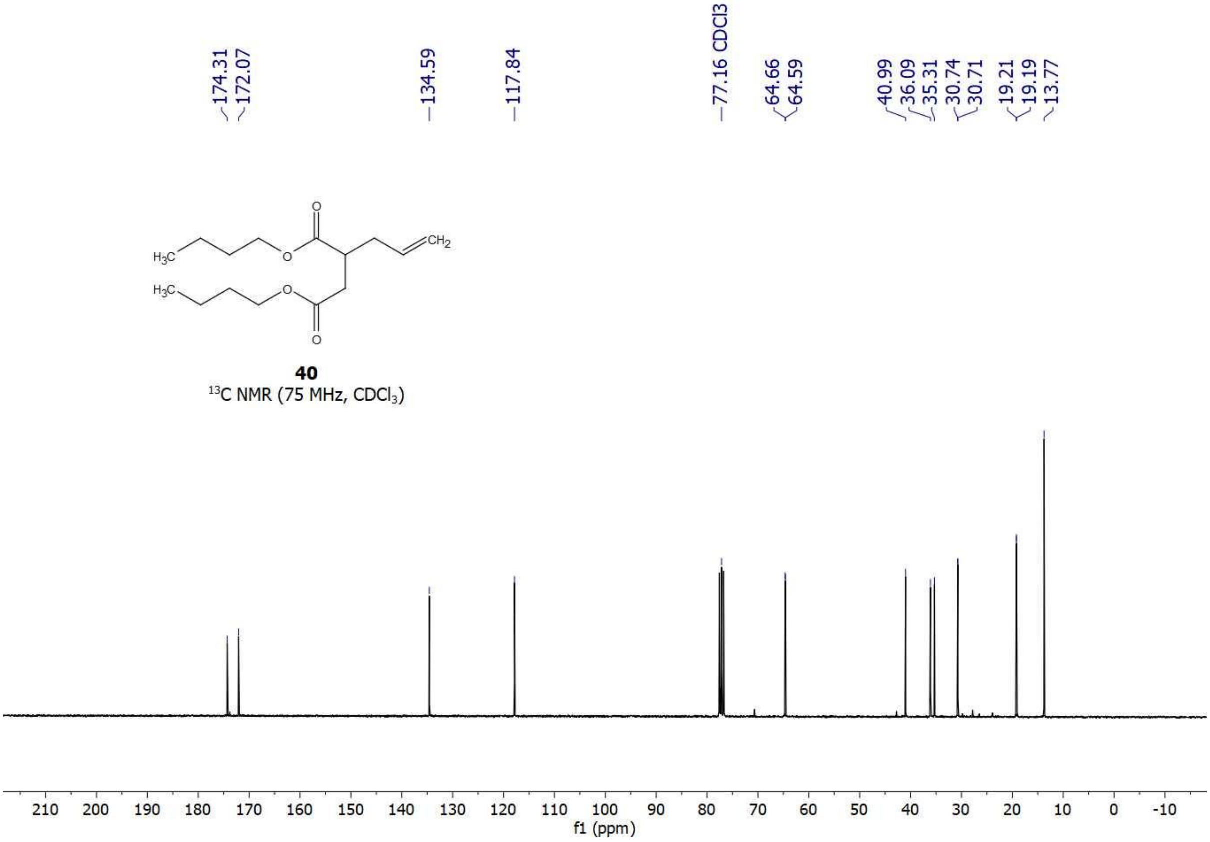

40

^{13}C NMR (75 MHz, $CDCl_3$)
